# A Comprehensive Phylogenetic Analysis of the MAP4K Family in the Green Lineage

**DOI:** 10.3389/fpls.2021.650171

**Published:** 2021-08-13

**Authors:** Lixia Pan, Cassio Flavio Fonseca De Lima, Lam Dai Vu, Ive De Smet

**Affiliations:** ^1^Department of Plant Biotechnology and Bioinformatics, Ghent University, Ghent, Belgium; ^2^VIB Center for Plant Systems Biology, Ghent, Belgium

**Keywords:** signaling, MAP4K, phylogenetic analysis, evolution, motifs

## Abstract

The kinase-mediated phosphorylation impacts every basic cellular process. While mitogen-activated protein kinase technology kinase kinases (MAP4Ks) are evolutionarily conserved, there is no comprehensive overview of the MAP4K family in the green lineage (Viridiplantae). In this study, we identified putative MAP4K members from representative species of the two core groups in the green lineage: Chlorophyta, which is a diverse group of green algae, and Streptophyta, which is mostly freshwater green algae and land plants. From that, we inferred the evolutionary relationships of MAP4K proteins through a phylogenetic reconstruction. Furthermore, we provided a classification of the MAP4Ks in the green lineage into three distinct.

## Introduction

Post-translational modifications (PTMs) are vital for plants to sense and respond to environmental changes (Hashiguchi and Komatsu, 2016; Vu et al., 2018). Among these, reversible protein phosphorylation is one of the most widespread and pivotal PTMs that affect every basic cellular process (Ardito et al., 2017; Vu et al., 2018). Phosphorylation is catalyzed by protein kinases, which predominantly phosphorylate substrate proteins on serine, threonine, or tyrosine residues, and protein phosphatases, which mediate the reverse reaction. One of the well-explored, highly conserved kinase subfamilies are components of the mitogen-activated protein kinase (MAPK) cascades, which act through linear sequential serine/threonine and/or tyrosine phosphorylation (Xu and Zhang, 2015). An MAPK signaling module is composed of an MAPK kinase kinase (MAP3K), a MAPK kinase (MAP2K), and a MAPK (Jagodzik et al., 2018). Typically, a MAPK cascade regulates signal transduction through a MAP3K that phosphorylates a MAP2K, which then activates a MAPK by phosphorylation (Xu and Zhang, 2015; Krysan and Colcombet, 2018). Plant MAPK cascades act downstream of receptor-like protein kinases or G-proteins and play important roles in immunity, abiotic stress, and plant growth and development (Cho et al., 2008; Cheng et al., 2015; Xu and Zhang, 2015; Liu and Zhou, 2018; Yan et al., 2018; Zhu et al., 2019).

However, the yeast Ste20 acts as a MAPK kinase kinase kinase (MAP4K) that activates Ste11, a MAP3K, which, in turn, activates downstream components in the mating signaling pathway (Leberer et al., 1992). Interestingly, MAP4Ks are conserved not only in yeast but also in mammals and plants (Dan et al., 2001; Champion et al., 2004; Chuang et al., 2016). The MAP4Ks in plants were first identified in *Brassica* (Leprince et al., 1999), but, so far, plant MAP4Ks have not been extensively characterized (Pan and De Smet, 2020). In *Arabidopsis thaliana*, 10 MAP4Ks were described (Jonak et al., 2002), but not all MAP4Ks have been functionally characterized: BLUE LIGHT SIGNALING 1 (BLUS1)/MAP4K10 is essential for stomatal opening in response to blue light (Takemiya et al., 2013; Schnabel et al., 2018), SALT INDUCIBLE KINASE 1 (SIK1)/MAP4K3 is critical for cell proliferation and expansion during organ growth and development (Xiong et al., 2016), and both SIK1 and MAP4K4 regulate the flg22-triggered immunity response (Jiang et al., 2019). In addition, a TARGET OF TEMPERATURE 3 (TOT3)/MAP4K4 interacts with TOT3-INTERACTING PROTEIN 4 (TOI4)/MAP4K6 and TOI5/MAP5K5 and controls thermomorphogenesis in Arabidopsis and wheat (Vu et al., 2021).

Although that MAP4Ks are so conserved throughout multiple clades, there is no comprehensive overview of the MAP4K family in the green lineage (Viridiplantae). Viridiplantae is a monophyletic clade of photosynthetic eukaryotic organisms that play important roles in both terrestrial and aquatic ecologies (Leliaert et al., 2012). The group separated around 1 billion years ago into Chlorophyta and Streptophyta, the latter including Embryophyta (the clade of the land plants) (Morris et al., 2018; Fürst-Jansen et al., 2020). One major evolutionary milestone was the conquest of land by the Embryophyta, which is from a freshwater streptophyte algal common ancestor, followed by an expansion in biodiversity (Becker and Marin, 2009). The habitat shift was accompanied by the modification of a series of traits that granted success in land colonization (e.g., the development of specialized organs and tissues such as stomata, roots, leaves, seeds, and flowers), but also a set of molecular innovations (Pires and Dolan, 2012; Ishizaki, 2017).

In this study, we identify putative MAP4K members from representative species of Chlorophyta and Streptophyta, the two clades in the Viridiplantae, and infer the phylogenetic relationships in the green lineage underpinning MAP4K evolution and diversification.

## Materials and Methods

### Genome-Wide Identification of (Putative) MAP4Ks

To identify and reconstruct the evolution of putative MAP4Ks throughout the green lineage (Viridiplantae), 6 Chlorophytes, and 29 Streptophytes (including 24 Embryophytes) were chosen depending on the genome availability and annotation status (Supplementary Table 1). The MAP4K sequences were identified using a hidden Markov model (HMM) profile generated and calibrated using the HMMER software v3.3 (Eddy, 1998) that was trained based on an alignment of the 10 MAP4Ks previously identified in *A. thaliana* (Supplementary Figure 1; Jonak et al., 2002). Local searches were performed on primary protein libraries (Leebens-Mack et al., 2019). To maximize the balance between sensitivity and selectivity, only hits with both per-sequence and per-domain *E*-values lower than 2e-100 and primary protein isoforms were considered for downstream analysis. The sequences retrieved were then further confirmed for the presence of the protein kinases domain (PF00069) in the Pfam database (El-Gebali et al., 2019) and InterPro Scan v5 (Jones et al., 2014) and inspected considering the annotation status for some of the libraries; the 5% longest and shortest were reviewed using a homology-based method (Korf et al., 2001). For this process, the canonical genomic structure [(e.g., evolutionarily conserved exons, introns, and untranslated regions (UTRs)] of the best reciprocal blast hits among *A. thaliana* (Streptophyta-dicotyledon), *Oryza sativa* (Streptophyta-monocotyledon), and *Chlamydomonas reinhardtii* (Chlorophyta) were compared for validation (Altschul et al., 1990, 1997; Moreno-Hagelsieb and Latimer, 2008).

### Alignment and Phylogenetic Analyses

To build a phylogenetic reconstruction for the MAP4K group, a MAPK family member (MPK3, AT3G45640.1) was first added to the list of putative MAP4Ks as a possible outgroup. Following this, full-length sequences were aligned using the GISMO software, which applies a top-down sequence alignment and is optimized for protein sampling (Neuwald and Altschul, 2016) (Supplementary Data Sheet 1). Subsequently, we inferred the maximum likelihood phylogenetic tree using the concatenated alignment under the best fitting model JTT + I + G4 in IQ-TREE v2.0.6 (Neuwald and Altschul, 2016), executing 1,000 ultrafast replicates. The resulting Newick consensus tree was visualized using the Interactive Tree Of Live version 4 (iTOL) (Letunic and Bork, 2019). The taxonomy tree was generated with the phyloT online tool (phylot.biobyte.de). In addition, a Bayesian Markov chain Monte Carlo (MCMC) sampling method for phylogenetic reconstruction was used. The alignment was submitted to the best fitting model JTT + G4 implemented in PhyloBayes with 5,000 generations and, after the convergence of the two chains, a burn-in of 25% was applied. The resulting consensus tree (data not shown) was inspected and compared with the previous tree, revealing a high conservation of the three clades (100%, 100%, and 99.41% for clade I, II, and II, respectively) and high posterior probability support (1,0.83, and 1 for Clade I, II, and III, respectively), but several unresolved polytomies.

### Feature Search and Characterization

To gain insight into the conserved motifs among plant MAP4K sequences, the MEME suite 5.3.3^1^ (Bailey and Gribskov, 1998) was used. The number of discoverable motifs was set to 15 and the width of motifs was set to an interval of a minimum of 3 and maximum of 50 amino acids. To create sequence logos, WEBLOGO^2^ was used with the default settings (Crooks et al., 2004). The analysis of disorder was performed by the web-based IUPred2A tool^3^ (Mészáros et al., 2018) utilizing the *A. thaliana* MAP4K protein sequences. The resulting disorder probability was used to categorize each residue as either ordered (<0.4), intermediate (0.4–0.6), or disordered (>0.6). Analyses of the biotic and abiotic influences on *A. thaliana MAP4K* expression was done through analyses of available transcriptome data on the Genevestigator platform^4^ (Zimmermann et al., 2004, 2005; Hruz et al., 2008) on August 25, 2020. The eFP browser^5^ (Hayes et al., 2008) with standard settings was used for the cell, tissue, and organ *MAP4K* expression analyses.

## Results and Discussion

### The MAP4K Family Members in *A. thaliana*

In *A. thaliana*, the MAP4K family consists of 10 members, namely, MAP4K1 (At1G53165), MAP4K2 (At3G15220), MAP4K3/SIK1 (At1G69220), MAP4K4/TOT3 (At5G14720), MAP4K5/TOI5 (At4G24100), MAP4K6/TOI4 (At4G10730), MAP4K7 (At1G70430), MAP4K8 (At1G79640), MAP4K9 (At1G23700), and MAP4K10/BLUS1 (At4G14480) (Pan and De Smet, 2020; Vu et al., 2021). Most *A. thaliana* MAP4K family members share common features, such as a MAP4K signature motif and several conserved residues in the kinase domain, and display a C-terminal half from the end of the kinase domain to the stop codon that is largely intrinsically disordered (Supplementary Figure 1). However, some differences can be observed. For example, SIK1 contains a long intrinsically disordered N-terminal part before the kinase domain, which is important for interaction with MOB1 to control cell expansion (Xiong et al., 2016; Supplementary Figure 1). Furthermore, MAP4K9 lacks the HRD and DFG motifs in the kinase domain that are vital for catalytic activity, does not contain an intrinsically disordered C-terminal part, and does not display the conserved MAP4K signature and GXGXXG/A motifs (Supplementary Figure 1). In *Homo sapiens*, MAP4Ks contain a characteristic, highly conserved C-terminal citron-homology domain (CNH) in all seven known members of the group (Chuang et al., 2016; Seo et al., 2020), which is also present in MAP4K orthologs in *Drosophila melanogaster* and *Caenorhabditis elegans* (Fiedler et al., 2014), which is important for protein–protein interactions (Poinat et al., 2002; Machida et al., 2004; Taira et al., 2004). Strikingly, a fully conserved CNH domain is absent in *A. thaliana* MAP4Ks.

To gain insight into the expression patterns of the *A. thaliana MAP4K* family during development, the *MAP4K* expression data were compiled and visualized from online repositories, namely, eFP browser (Winter et al., 2007) and Genevestigator v3 (Hruz et al., 2008). These *in silico* expression patterns suggested that most *MAP4Ks* are broadly expressed throughout the plant, with some *MAP4Ks* expressed stronger or specifically in one organ, such as in pollen (Supplementary Figure 2). This indicates distinct roles for some of these kinases. Furthermore, the *MAP4K* expression was hardly affected by hormones or nutrients and was mainly regulated by abiotic and biotic stresses (Supplementary Table 2).

### Identification of Putative MAP4K Family Members in the Green Lineage

**FIGURE 1:**
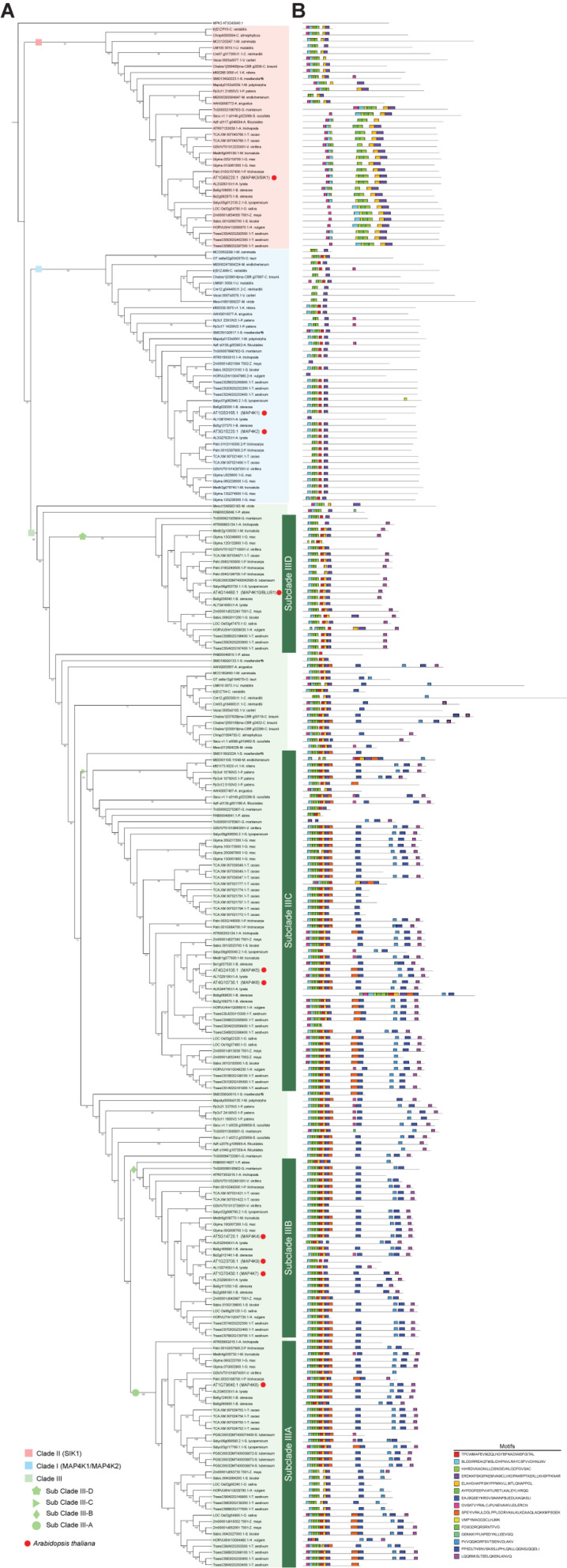
Combined phylogenetic tree and motif locations of the green lineage MAP4Ks. **(A)** Inferred phylogenetic tree of putative green lineage mitogen-activated protein kinase MAP4Ks among representative species. The maximum likelihood tree was inferred from the multiple alignments of 239 putative MAP4K sequences and 10 previously described *Arabidopsis thaliana* MAP4Ks sequences across 34 Viridiplantae primary proteomes using IQ-Tree. Three main clades are indicated: I, II, and III. Clade III contains additional subclades. IIIA, IIIB, IIIC, and IIID. Bootstrap values > 24% are indicated in the branches. Red dots indicate Arabidopsis MAP4Ks. The Arabidopsis MAP4Ks included in the (sub)clades are MAP4K1 (I), MAP4K2 (I), MAP4K3/SIK1 (II), MAP4K4 (IIIB), MAP4K5 (IIIC), MAP4K6 (IIIC), MAP4K7 (IIIB), MAP4K8 (IIIA), MAP4K9 (IIIB), and MAP4K10/BLUS1 (IIID). **(B)** Motif distribution along the putative MAP4K sequences identified by MEME tool. The height of motif “block” is proportional to -log (*p*-value), the taller the block, and the lower the probability of a wrong match. The motif sequence is indicated in the figure.

While there is some information on the MAP4K family members in a few plant species, such as *A. thaliana*, *Brassica napus*, *Zea mays*, *Solanum chacoense*, and *Vitis vinifera* (Leprince et al., 1999; Llompart et al., 2003; Major et al., 2009; Cakir and Kılıçkaya, 2015; Pan and De Smet, 2020), a comprehensive overview is lacking. Therefore, to reconstruct the evolutionary relationships of the MAP4K family in the green lineage, we analyzed 34 representative species with available genome or protein information (Tuskan et al., 2006; Jaillon et al., 2007; Merchant et al., 2007; Ouyang et al., 2007; Palenik et al., 2007; Worden et al., 2009; Blanc et al., 2010; Prochnik et al., 2010; Schmutz et al., 2010; Banks et al., 2011; Hu et al., 2011; Young et al., 2011; Lamesch et al., 2012; Sato et al., 2012; Albert et al., 2013; Motamayor et al., 2013; Nystedt et al., 2013; Sharma et al., 2013; Blanc-Mathieu et al., 2014; Hori et al., 2014; Liu et al., 2014; Hirsch et al., 2016; Beier et al., 2017; Bowman et al., 2017; Appels et al., 2018; De Clerck et al., 2018; Lang et al., 2018; Li et al., 2018; McCormick et al., 2018; Nishiyama et al., 2018; Wan et al., 2018; Cheng et al., 2019; Wang et al., 2020; Zhang et al., 2020; Supplementary Table 1). In this analysis, we included representative green algae from Chlorophyta [*Chlamydomonas reinhardtii* and *Volvox carteri* (Chlorophyceae), *Micromonas pusilla* (Mamiellophyceae), *Ostreococcus tauri* and *Chlorella variabilis* (Trebouxiophyceae), and *Ulva mutabilis* (Ulvophyceae)] and Streptophyta [*Klebsormidium nitens* (Klebsormidiophyceae), *Chlorokybus atmophyticus* (Chlorokybophyceae), *Mesostigma viride* (Mesostigmatophyceae), *Mesotaenium endlicherianum* (Zygnematophyceae), and *Chara braunii* (Charophyceae)]. In addition, several land plants from the Streptophyta were included to trace the expansion of MAP4Ks in land plants: the bryophyte *Physcomitrium patens*, the liverwort *Marchantia polymorpha*, the hornwort *Anthoceros agrestis*, the lycophyte *Selaginella moellendorffii*, the ferns *Azolla filiculoides* and *Salvinia cucullata*, the gymnosperms *Picea abies* and *Gnetum montanum*, and 16 angiosperms (including *Amborella trichopoda*, 10 eudicots, and 5 monocots). Through in-depth analyses of protein-containing databases (see section “Materials and Methods”), we generated a set of 249 putative MAP4K family members with a minimum and maximum amino acid sequence length of 225 and 2,251, respectively (Supplementary Table 3 and Supplementary Data Sheet 2). These results showed that putative members of the MAP4K family are present in both Chlorophyta and Streptophyta and all representative species used in this analysis (Figure 1 and Supplementary Tables 1, 3). The resulting phylogenetic consensus tree revealed three major clades (Figure 1). With respect to clade III, four subclades could be distinguished in angiosperms: one subclade with AtMAP4K8 (Subclade IIIA), a second subclade with AtMAP4K4, AtMAP4K7, and AtMAP4K9 (Subclade IIIB), the third subclade with AtMAP4K5 and AtMAP4K6 (Subclade IIIC), and the fourth clade with AtMAP4K10 (Subclade IIID) (Figure 1 and Supplementary Table 3).

### Conserved Features of the MAP4Ks in Plants

**FIGURE 2:**
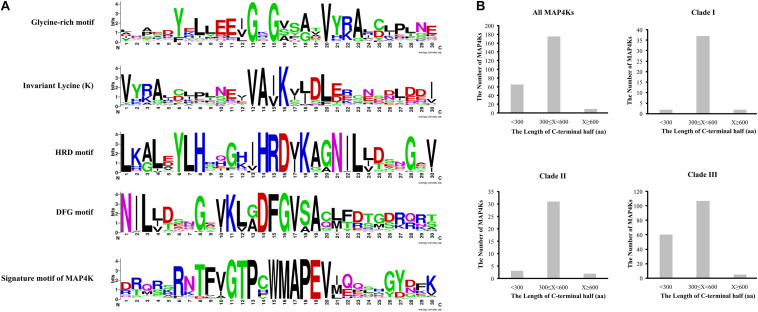
MAP4K features. **(A)** Sequence logos of main conserved motifs. **(B)** Length of C-terminal half for all MAP4Ks and indicated clades.

As proteins are generally composed of one or more functional regions or domains that can provide insight into their function and evolutionary relationships (Lee et al., 2007), we explored to what level the key domains and motifs are conserved in the putative MAP4K family members (Figures 1, 2A, Supplementary Figure 3, and Supplementary Table 3).

First, we assessed to what extent the identified sequences have a likely functional kinase domain based on some key features, such as a glycine-rich loop motif (GxGxxG) that is essential for nucleotide binding and is an integral part of the ATP-binding site. In general, in about 20% of kinases, the third G is substituted by A or S, which disrupts kinase activity (Hanks and Quinn, 1991; Hemmer et al., 1997; Torkamani et al., 2008; Chmielecki et al., 2010; Steinberg, 2018; Zhang et al., 2018). We also observed this in several MAP4Ks (Supplementary Table 3 and Supplementary Figure 3). In addition, we analyzed HRD and DFG motifs and an invariant lysine (K), all of which are pivotal for catalytic activity (Hanks et al., 1988; Carrera et al., 1993; Hanks and Hunter, 1995; Xu et al., 2000; Roskoski, 2004; Kannan and Neuwald, 2005; Zhang et al., 2015; Modi and Dunbrack, 2019; Pan and De Smet, 2020; Supplementary Table 3 and Supplementary Figure 3). Due to changes in these amino acid triads, the putative MAP4Ks that lack all or some of the above-mentioned kinase features are possibly kinase-inactive MAP4Ks (Supplementary Table 3; Paul and Srinivasan, 2020).

Second, we evaluated the presence of the previously proposed signature motif for (mammalian) MAP4Ks (GTPyWMAPEv, with Y and V being less conserved) located in the kinase subdomain VIII (Sells and Chernoff, 1997; Dan et al., 2001). The majority (94%) of the putative MAP4Ks in the green lineage share this well-conserved signature motif (Supplementary Table 3 and Supplementary Figure 3). Interestingly, two *A. thaliana* and *A. lyrata* MAP4K9s from subclade IIIB do not contain this conserved signature motif and show severe alterations in the HRD, DFG, and glycine-rich loop motifs (GxGxxG) (Supplementary Table 3), in addition, which raises the question if these should still be considered as true (functional) MAP4Ks.

Third, we investigated the length of the C-terminal half of the MAP4Ks. Although the disordered C-terminal half is essential for the interaction with substrates, it is largely not conserved across sequences (Lee et al., 2017). For almost 70% of the putative MAP4Ks, the length of the C-terminal half was between 300 and 600 amino acids (Figure 2B, Supplementary Figure 4, and Supplementary Table 3). Putative MAP4Ks that (partially) lack such a C-terminal half, such as SMO169G0133.1 in *Selaginella*, TraesCS6A02G353400.1 in wheat, and Bo9g089620.1 in *Brassica*, will likely be affected in the interaction with substrates (Zhang et al., 2018; Jiang et al., 2019).

Strikingly, none out of the 249 sequences contained a fully conserved CNH domain, suggesting that this characteristic element for several non-plant MAP4Ks might not have been present in the ancestor of the Viridiplantae. However, we identified conserved but clade-specific motifs at a similar position where the CNH domain is found in other eukaryotes (Figure 1, Supplementary Figures 1–5, and Supplementary Table 3). Furthermore, an experimental analysis will be required to reveal whether these motifs are involved in similar functions as the CNH domain, such as protein–protein interaction.

Finally, the phototropin-mediated phosphorylation of BLUS1 Ser-348 alleviates the autoinhibitory activity of the C-terminal part on its kinase activity (Hosotani et al., 2021). This serine residue belongs to a conserved RRI**S**GWNF consensus motif (Takemiya et al., 2013). Therefore, we checked for the presence of such a motif in all the putative MAP4Ks. This revealed that an S(G/A)WNF motif is absent in Clade I and Clade II of MAP4Ks, but present in 64% of Clade III MAP4Ks (Supplementary Table 3 and Supplementary Figure 3).

### Evolutionary Insight in the MAP4K Family in the Green Lineage

**FIGURE 3:**
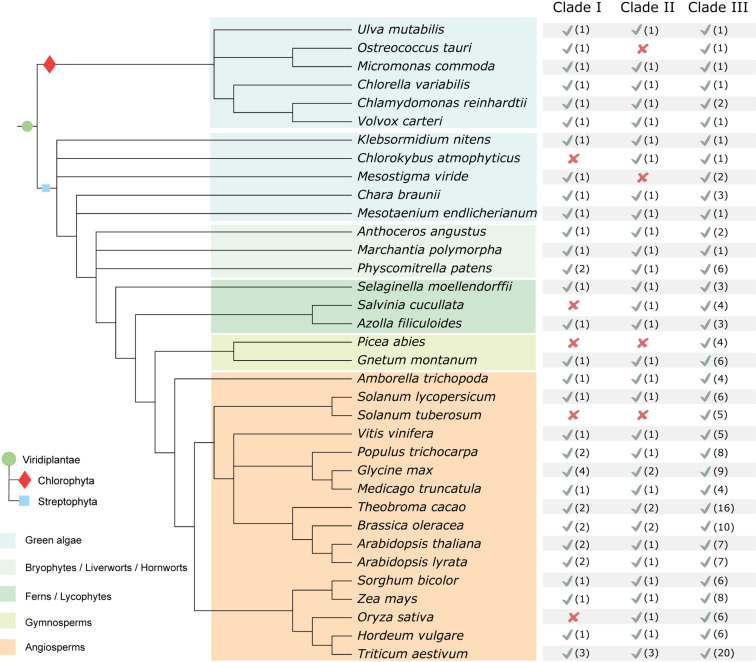
Summary of MAP4K family occurrence in the green lineage. Table based on the results of the inferred MAP4K phylogenetic tree within selected species. The presence (√) (and the number of MAP4Ks) or absence (x) of a likely functional ortholog are indicated. It should be noted that for those with missing clades, this can also be due to an incomplete genome.

Based on the tree topology of the inferred phylogenetic reconstruction of the MAP4K family, we divided the group into three major clades (I, II, and III). In addition, based on the protein motif structure and expansion in flowering plants, we defined four subclades in clade III (IIIA, IIIB, IIIC, and IIID) (Figure 1). While MAP4Ks are present in all the plant species we investigated, the absolute number of group members increased in land plants, especially in Clade III, and some species seemed to lack members for a particular clade (Figure 3). For example, *O. tauri* (Chlorophyta), *Mesostigma viride* (Streptophyta), *Picea abies*, and *Solanum tuberosum* lacked a clade II member, and clade I was absent in *C. atmophyticus* (Streptophyta), *Salvinia cucullate* (Fern), *P. abies*, *S. tuberosum*, and *O. sativa* (Figures 1, 3). Furthermore, Subclade IIIA (which contained angiosperm-exclusive MAP4Ks) appeared as a sister group of Subclade IIIB (which was exclusive to seed plant MAP4Ks) and orthologs for both clades were identified in ferns. Likely, the increase in the number of MAP4Ks in vascular plants (ferns, gymnosperms, and angiosperms) is associated with the increased rate of whole-genome duplications or polyploidization largely observed in land plants; thus, it is often invoked as one of the main causal agents for diversification and land colonization (Bowers et al., 2003; Qiao et al., 2019; Zhang et al., 2019). In addition, Subclade IIID (mainly defined by the presence of BLUS1) contained only members of gymnosperms and angiosperms (Figure 1), extending this beyond the angiosperm lineage (Harris et al., 2020). However, the single *Gnetum montanum* (TnS000821935t04) MAP4K sequence identified as belonging to this clade has retained only some of the described angiosperm motifs and lacks the LQQRMISLTEELQKEKLKNVQ motif in the disordered domain, which is likely essential for protein–protein interactions (Figure 1). Therefore, whether the gymnosperm MAP4K in subclade IIID retains a similar function as the other members of the clade remains unknown.

## Conclusion

In this study, we identified putative MAP4K members from representative plant species and inferred the phylogenetic relationships in the green lineage underpinning MAP4K evolution and diversification. As a whole, the presence and absence of typical sequence motifs in (putative) MAP4K sequences likely resulted in a functional diversification within the MAPK4 family. The next important and necessary step is thus to evaluate the functionality and activity of these MAP4Ks, not only in *A. thaliana*, but also in other members of the green lineage. Importantly, this also relates to some of the motifs that were identified and the large intrinsically disordered C-terminal half. Some of these analyses will be facilitated by the fewer MAP4K family members identified in this study in, for example, *Marchantia*, which is a valuable model for genetic studies (Ishizaki et al., 2016; Poveda, 2020).

## Data Availability Statement

The original contributions presented in the study are included in the article and Supplementary Material. Further inquiries can be directed to the corresponding author.

## Author Contributions

LP, CF, and LDV performed analyses. LP, CF, LDV, and ID interpreted the results and wrote the manuscript. ID coordinated the study. All authors contributed to the article and approved the submitted version.

## Conflict of Interest

The authors declare that the research was conducted in the absence of any commercial or financial relationships that could be construed as a potential conflict of interest.

## Publisher’s Note

All claims expressed in this article are solely those of the authors and do not necessarily represent those of their affiliated organizations, or those of the publisher, the editors and the reviewers. Any product that may be evaluated in this article, or claim that may be made by its manufacturer, is not guaranteed or endorsed by the publisher.

## Footnotes

1
http://meme-suite.org/tools/meme

2
https://weblogo.berkeley.edu/logo.cgi

3
https://iupred2a.elte.hu/

4
https://genevestigator.com/gv/start/start.jsp

5
http://www.bar.utoronto.ca/efp/cgi-bin/efpWeb.cgi
