# Supplementary material for: A Comprehensive Phylogenetic Analysis of the MAP4K Family in the Green Lineage
Source: Front Plant Sci. 2021 Aug 13;12:650171. doi: 10.3389/fpls.2021.650171 (PMC8415026; doi:10.3389/fpls.2021.650171)
Supplement: Supplementary file 1 [file Presentation_1.pdf]

Supplementary Material

1 Supplementary Data

Supplementary Table S1. List of Viridiplantae representative species used to search for putative MAP4K proteins

Supplementary Table S2. The effect of hormones, nutrients, and stress on MAP4K expression

Supplementary Table S3. Overview of (conserved) motifs and the length of C-terminal half in all putative MAP4Ks

Supplementary File S1. All Protein sequences of putative MAP4Ks

Supplementary File S2. Alignment file of putative MAP4Ks.

2 Supplementary Figures and Tables

2.1 Supplementary Figures

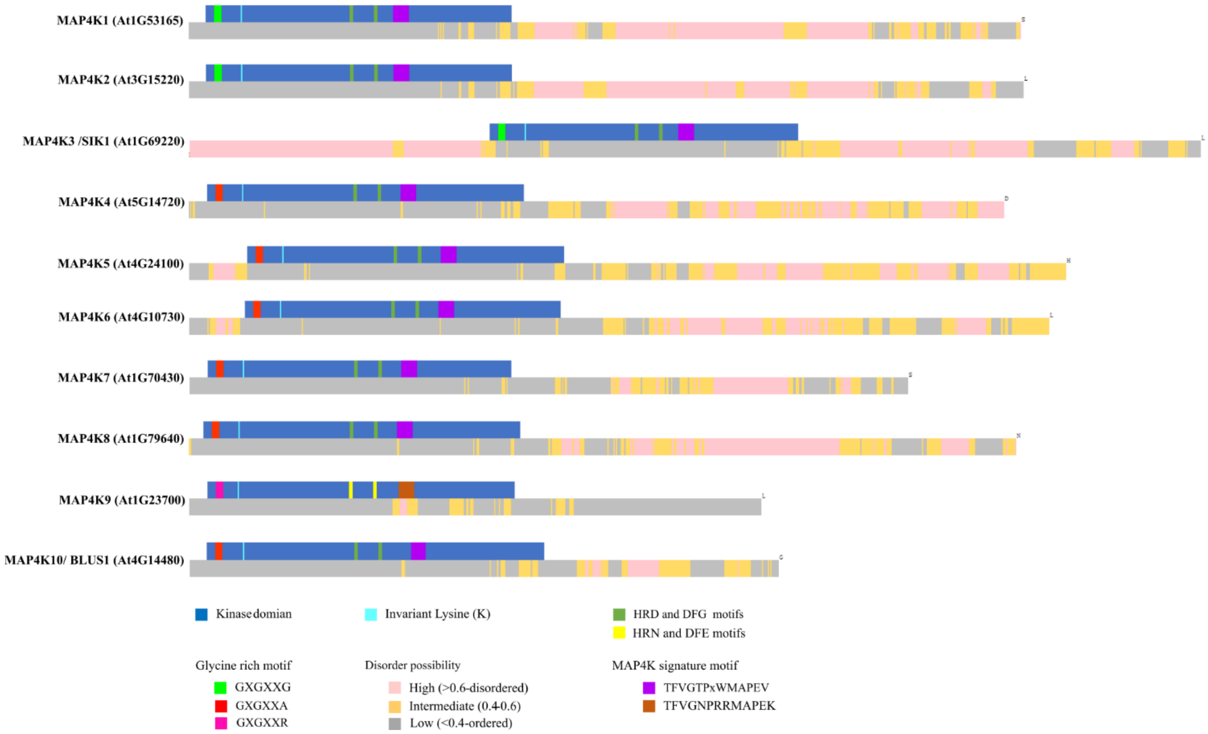

Supplementary Figure 1. Conserved domains, motifs and disorder possibility for Arabidopsis MAP4Ks are indicated.

MAP4K1 (At1G53165)

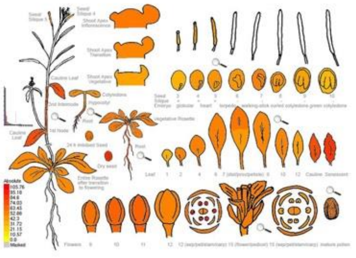

MAP4K2 (At3G15220)

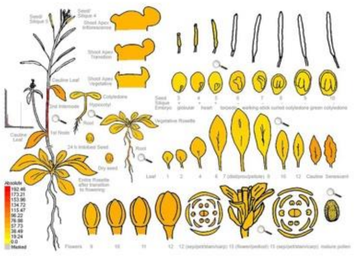

MAP4K3/SIK1 (At1G69220)

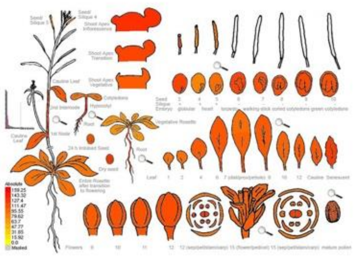

MAP4K4 (At5G14720)

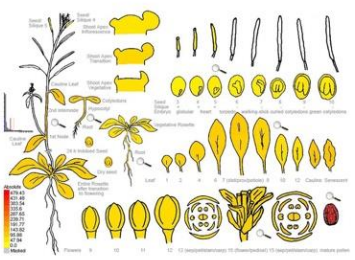

MAP4K5 (At4G24100)

No information available

MAP4K6 (At4G10730)

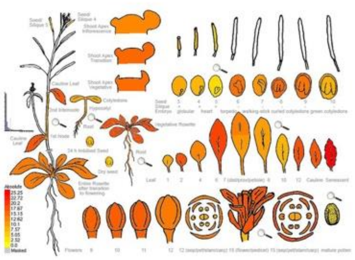

MAP4K7 (At1G70430)

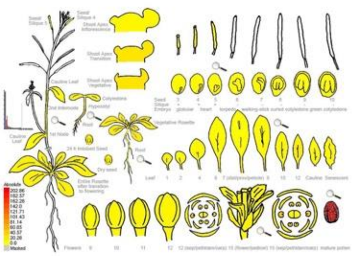

MAP4K8 (At1G79640)

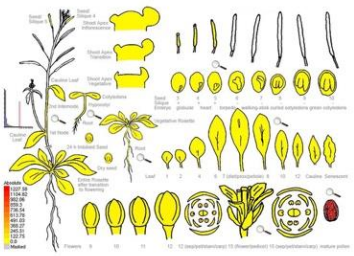

MAP4K9 (At1G23700)

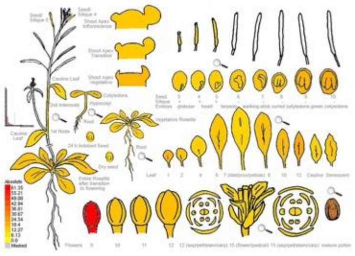

MAP4K10/BLUS1 (At4G14480)

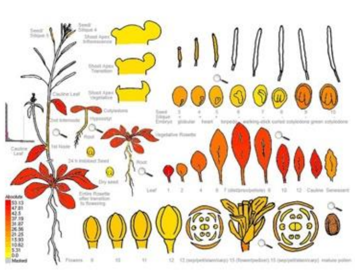

Supplementary Figure 2. The *in silico* expression patterns for *MAP4Ks*. Data from eFP browser are depicted. For *MAP4K5*, no data were available.

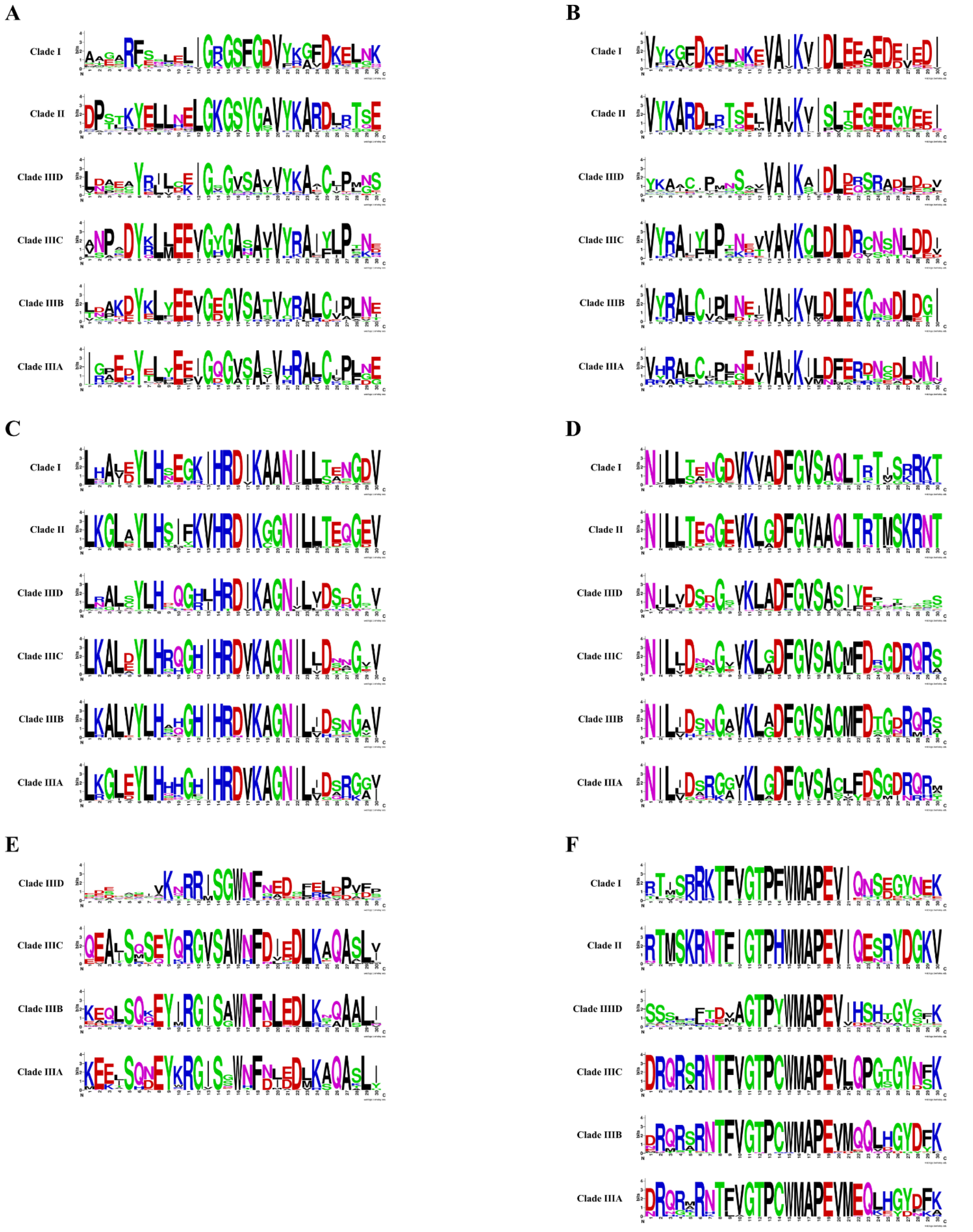

Supplementary Figure 3. Web Logo of featured motifs and signature motif per (sub)clade. **(A)** Glycine-rich motif. **(B)** Invariant lysine (K). **(C)** HRD motif. **(D)** DFG motif. **(E)** S(G/A)WNF motif. **(F)** MAP4K signature motif.

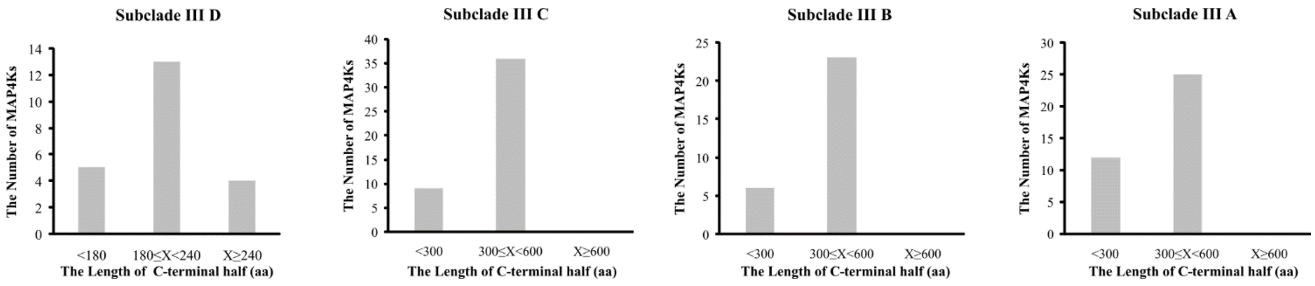

Supplementary Figure 4. Length of the C-terminal half for MAP4Ks subclades.

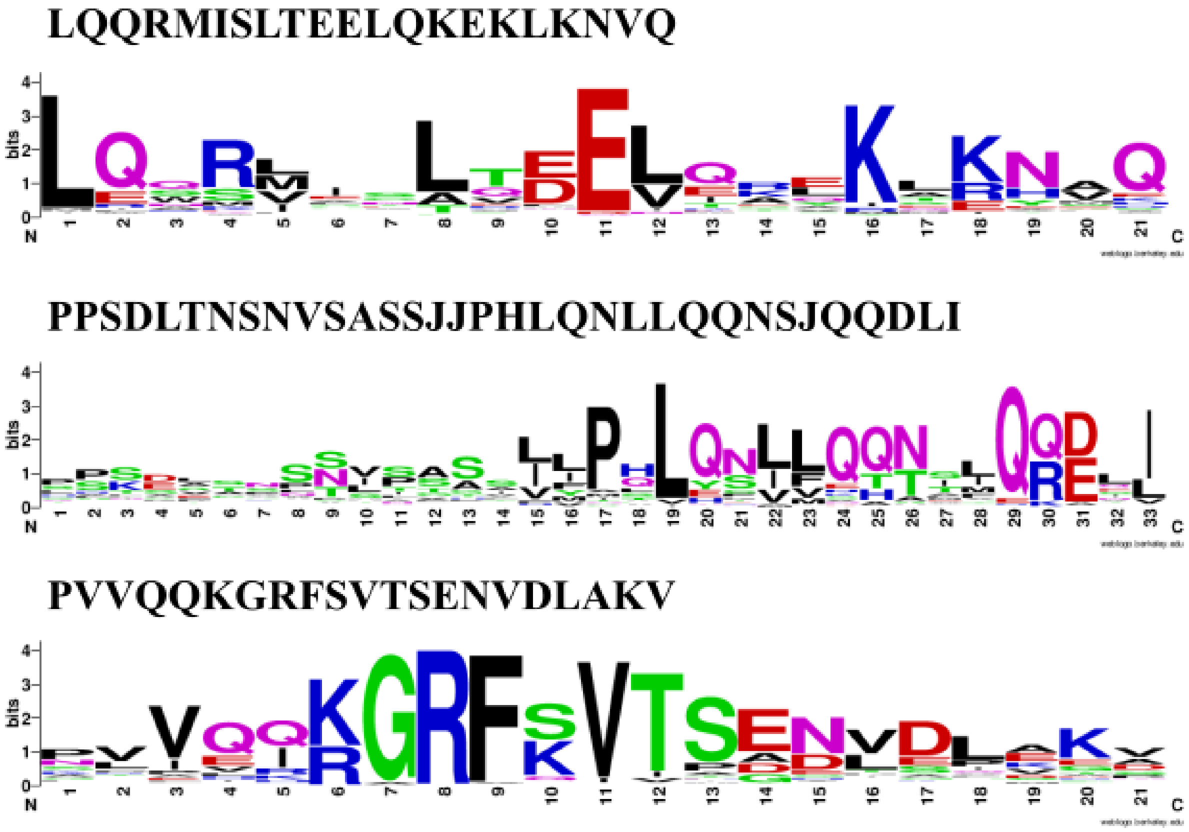

Supplementary Figure 5. Web logo of selected conserved motifs only present in Clade III from MEME suite.
