# Supplementary material for: A Comprehensive Phylogenetic Analysis of the MAP4K Family in the Green Lineage
Source: Front Plant Sci. 2021 Aug 13;12:650171. doi: 10.3389/fpls.2021.650171 (PMC8415026; doi:10.3389/fpls.2021.650171)
Supplement: Supplementary file 2 [file Data_Sheet_2.docx]

>kfl00266_0050_v1.1

MAESLEEERSGVVVEEPGAQQEAGGVTSYEDPSLQYDLLDELGKGSYGAVYKARSLRTNELVAIKVISLSEAEEGYEEIRGEIEMLQQCNHPNVVRYLGSHQGEDYLWIVMEYCGGGSVADLMHLSDEPLEEAQIAYICRESLKGLAYLHSIFKVHRDIKGGNILLTDKGEVKLGDFGVAAQLTRTMSKRNTFIGTPHWMAPEVIQESRYDGKVDVWALGISAIEMAEGLPPRSNIHPMRVIFMISREPAPMLEDKEKWSLLFHDWVAKCLAKDPRVRPTAQALLQHKFIEKSKAGASVLLPLIERCKILKAQLLEQQRKEEEAGASTSVGEWSWGRGSTVRVGGGTVVVRSDQETLPAGGGAALASAMAAGKAGSGEFGTFVVKDGDGTGTVKSKVRLQADGAPSDDRVSGALPSPLRPSQLPSFNASGRPLPGFSLRTTSPGVTSPGYFSPSWRGKISPAETFTGGTSPESNAGRQPALPDKLLSIYAAGNTVPIPFLRATDVAPLALLERPADPLTSSQTSDAALLEVLQELYSGSATGEGGLRKQRKKGGEELALPAGVYKLLASSGTLPNLVRALAYHKQRLEETPLQPWQAADQQRVVDNLSDTLRTILRL*

>kfl00339_0070_v1.1

MMATDNFEATKIIGKGSFGDVYRGVDKATGKDVAIKIIDLEEAEDEVDDIQKEISVLSQCRSPYVTEYYGAYLQGTKLWIVMELMACSVADMLETGPPLDEAAIACVLRDLLKALDYLHGEGKIHRDIKAANILLTSEGEVKVADFGVSAQLTRTVSKRKTFVGTPFWMAPEVIQHSEGYNEKADIWSLGITAIEMAKGEPPLADLHPMRVLFLIPKSPSPQLDDHFSRPFKEFVALCLKKDPAERPTAKELLKHRFIRSAKRPAKLLEKISEHVSQREAQGLAAGSPVQTRIAQATLPRRPATKDASWDFGSLVPPPRAAAASPGTSQPSTPTLGFGTGTVRARQPPVAPGGWQEEAGTPSAGPGEGPYREAGSGEEHLERAPSFSDTVGGSTTEAGGPTGGSVFSGTWARLRESDDDGAAADSISGTVVVRTGGRETTGREAPEALGPARSRSPAPFSRSISERPPTPPLGDLRRASSDAPGRQPPPLQREDSATNLAEASAALGRRKPGLSWKGDSDERDRRSPSPAASPRPYENGASAARSPSPAKPANRGRGSAPGSSSSLLNLVLVPAIREAGEAVKGDAAARRAAAAVSEAVVDFERAAPGGSRALLHSVLHKVARSDDPAVRDLRTRAFEGLHVAPAPPLAKEDLAGLPAIASFLLRRWQAQTAAEFSSTPVQ*

>kfl01173_0020_v1.1

MAELQRAYPTTAADYKLHEEIGQGVSAVVYRATCLPYMEVVAVKVLDLEKCNNSLDEIRKEAQTMSLINHPNVVKAYCSFVVDVNLWVVMPFLAGGSCLHIMKSAYPEGFDEPVIATVLKETLKALDYLHRHGHIHRDVKAGNILIDTNGTVKLADYGVSAFMFDSGDRQRSRQTFVGTPCWMAPEVMEQVHGYDFRADIWSFGITALELAHGHAPFSKYPPMKVLLMTLQNAPPGLDYPQDKRFSKSFKELIGLCLVKEPGKRPTAEKLLKHSFFKHAKTADYIQRHILDALPPLGERVKNLKAKDAARRAQAKMPMIEQEERSNNEYKRGVSGWNFDLDDLKAQAALMEDDEPPARDDRSRRTSAASVDEYERESPPVSAVPTPSAEREGARFTDVPLPSALSGKSGPSGPSLRGSASDKEMKPLSSTSQREKPKNFSGPLPMGRKEPKHVGRFDVFEDDNDTSPDYRARDRDRLAATIGPTDRLGGSSKDRSGDRGSAVVGPSLSMSRDRDRSGNDSRRDTEYRASEGERRQGGNGGTSSRSTSKDGDGKPAPVVQKKGRFSVSEEVEEPHMHKSASVPDWPADREKEKPAVRFAQTVAGPQSRQELNVLGVSTGGETVPVSAVLPHLQSLMHHAAVQQDVVLALMNSLSPADLRQSYTSRRDLSRTVSAVSGEFSPLDLPTERERELQQQVIELQTKVASLLDDIQTLKGRNAGLERQLNQIHNEEEERRIKAEEEADADHQ*

>AANG006772

MQRSSVEEHVDEMENDHSKDIRRRSGATNDNVTREDPSLKYELLNELGKGSYGAVYKARDLRTSELVAIKVISLSEGEEGYEEIRGEIEMLQQCNHPNVVRYLGSYQGEDYLWIVMEYCGGGSVADLMSITDEALEEQQIAYICREALKGLAYLHSIFKVHRDIKGGNILLTEQGEVKLGDFGVAAQLTRTMSKRNTFIGTPHWMAPEVIQESRYDGKVDVWALGVSAIEMAEGLPPRSNVHPMRVLFMISSEPAPMLEDKEKWSLVFHDFVAKCLSKEPRLRPTATEMLQHKFIEKCKVNAAAMLPRIDKARIIRTEMIAREAALEKKQGTVSSGGGQWSWEKGQTVKMNETLGGTVLVRQLISVYAAGNIVPIPFLKATDISPIALLSDSSGDGRPDQSGGIALEAIQELCNGVSFADAQLRKGRKVTNTEAPLPPGVYQRLATSTTLPNLARALAYHNMCYEEMPLKGAQAAQEQQIIENLSDTLRTILRL*

>AANG007497

MTSFKEIVDSIAPESRVDLKQMLCCCVAPKSSIDSIRKFSEGTNFYHLEDKIGEGGTATVWRATYLETDEIVAIKVVYLDRDKLDLQKLHNETKIMQLVHHPNVLGCYASFAVERTLWFVLPFMAGGSCSLILKENDNKGFPEPIIATILRDVLQALEYLHLKGIIHRDVKGGNILLGGRGEVKLADFGSAALAKPGEGAFSEFTGSLCWVAPEVILQPKVPAYDYKADLWSFGITAIELAQGKPPGINKAPTDLLQERVINSPPRLEETELSKFSSEFQDMLQQCLIKEPQNRCTASALLRHPFFKKARSHDYLVSHILKDLPPLEQRFLRSQKSKVIEKREKVSKIIRKAKRK*

>AANG002657

MEEKKTYPVVASGYKILEEVGQGVSATVYRAMCLPLSEIVAIKSLDLEKCNSNLDEIRREAQTMSLISHANVVKAHCSFVVDQSLWVVMPYMSGGSCLHIMKAAQPNGFEEAVIATVLRETLKALEYLHRHGHIHRDVKAGNILVDSNGSVKLGDFGVSACMFDTGDRQRSRNTFVGTPCWMAPEVMEQVHGYDFKADIWSFGITALELARGHAPFSKFPPMKVLLMTLQNAPPGLDCEGDKRFSKSFKEMIAMCLVKDPTRRPSAEKLLKHSFFKHARSADYIAKHLLEGLPPLGERVKSLKDKDAVRLAQKKMPYGEQEEKSQKEYQRGVSSWNFNVEDLKAQAALIQDDDEPAISKVPEEEEQSKPRSSRESDDSLEAASQAASSSPQPSVTRDSQMTTSASTLKPETATDVGDRASRNRTGQLSTALQSKSFPRSGPQLLGRKEPKHIGRFDVFEDDGSPRWHEPAKAQELRRTESSRDVRDASEVRSLSDKDRRDDLKRDDRDELRRPASGPLIPDHVAGKSVDAVLGNESFDVLIPTGIGTTGRQVGPAMRLQACSKWVFKTSSKYVPANHDCSWGMLTSFAVGIKEGPDEKPKGPVIQKKGRFSVVSDDVAMAEVTTLQIPFWVRKSIIQVIDQRGALRPGRFTSLPVLVTGHQNEGSLYATSPCNVLYVYGCQFPSFEGKRGSVPGTGLRQQLAQNSSFSQPPPSIPTNSSTVPVTAMITQLQNIYNQGAVQQDSLLNMINSLSTGDAAAGQRLNHSSSKHLPRSSSISAGEYPMEVPTERERDLWHQFTELQNKYANSLVEMHNLKSRNGQLERQLNAIYNKEEEERIRREEAAKEDG*

>AANG014577

MEARFQPLQLIGKGSFGDVYRGFDKELGKDVAIKVIDLEEAEDEVDDIQKEISVLSQCRCAFITEYYGSYLHKTRLWIVMEYMAGGSVSDLLEMGPPLDEASIACILKDLLQALDYLHSEGRIHRDIKAANILLTANGDVKVADFGVSAQLTRTMSKRKTFVGTPFWMAPEVIQNSEGYNEKADIWSLGITAIEMAKGEPPYSDLHPMRVLFLIPKNGPPQLDEHFSRPMKEFVALCLKKNPAERPTAKELLKHRFVKNARKSQRLLERIKERPVNYVGKGRDMGGLDDETKEKMKAMKGLGDRDSAGGRKSRYASWDFGTGTIQTTGTIRSAAVKPPAKGRSDAAPSPRLTTILSQAESEWQMEQPDLTPLGREGLRGTQEGEGSEGASTPEDESNVADADADIYGTVVRNSSEHSSDSGTPKLIQEDSATNLHEAMAAFRKAHMRPGRPPVRDRDHGYEAASPARTPDRSGRFLGRPASRPSSRPTSEEDFAQVSGAGPSSALSLVLLPALSESVPEQSENAAFRLAAELAELLIEFEHLVPGASEALIRKLFHKLGSTDDPSLKGLQSVARGVFAPEEGLSQRRDSIQSEFGKSRSGRLRSTLDASENSGLSPVAAFLLSRWQGQVNRDVAPCP*

>Azfi_s0117.g046584

LRREGHEEEEEEEEEEEEEEEEEEEEPPLLLQSLAKRFAGTDAFDVTEDPSDIIPIFPARDDDESSVVLHSATTSTEDAGAVLRGDDFSAAIRYMQKDAHISRIRARHQKEKENLLEEDEDDYDNGDDSFSTFVVKRPDKKQVPISGPRLGTSRNDSSDVYSTILHRTGGENAFSTVVTKNGDSTNSISRAVESFRREKETSERKRGLKDARRDAHEKSQHAGRRGSSPAQDNVIREDPTLKYELLDELGKGSYGSVYKARDLKTSELVAIKVISLSEGEEGYEDICGEIEMLQECNHPNVVRYLGSYQAEEYLWIVMEYCGGGSVADLLQVTEETLEERQIAYVCAEALKGLAYLHSIYKVHRDIKGGNILLTEQGEVKLGDFGVAAQLTRTLSKRNTSIGTPHWMAPEVIQGTQYDGKVDVWSLGISAIEMAEGLPPRCHVHPLRVLFMILKEPAPMLEDKEKWSLIFHDFVAKCLTKETRIRQSATDMLEHKFIARCNGNASCMLPRIEKARLIMLQTVPQRTLQNLDTSVGTTLPQQRSWERGNTVKMGEVYGATVLVKGEKTTTQESLRKSFQVQGTTIVHGDEEFAEFNTMVVHLEDSSNEVELQQADNYSEVKKTEDNSDSLAEGFSDSKIGHVRNHEGALAEVEVHGVDPVATQTQSSIPVQQSSLNDTKHIKGVIESGEMYTAPALSTPRGLLAGGRQTFSLQDKLLSFYCGGNTVPIPFLRATDISPLALISNSIFGEEQVDKGNSVALEAVQELYNGGGLGDAPPKKGRKPLTSELPLPSSVHKRLATSTTLPNLARALAYHKLCYEEMPLQGWQAGQEELKVQNLSDTLRTILHL*

>Azfi_s0139.g051166

MERKASFSSDPRAEKKMYPLAASEYKLLEEIGKGVSAIVYRAKCIPLNEFVAIKALDLERCNSSLDDIRREAQTMSLINHPNVVKAFCSFVVEQFLWVVMPFMAGGSCLHIMKAAYPDGFEEPIIASFLKETLKALEYLHNQGHIHRDVKAGNILVDGNGTIKLGDFGVSACMFDTGDRQRSRITFVGTPCWMAPEVMEQVHGYDFKADIWSFGITALELAHGHAPFSKYPPMKVLLMTLQNAPPGLDYERDKRFSKSFKEMVAMCLVKDPSKRPSAEKLLKHSFFKHVKSADYIARTILDPLPPLWQRVRDVKMRDEARLAEKKMPYGEQEARSQFKSDMEANFGCHFHREPKCLLSELDDKGLKVMLIIGIMKNEYKRGVSSWNFDLEDLKAQAALNEAEPPVKDDVKKDSDVTEINCASSPPLTSSQRAVSESNSCQPAGAVKAENATDESEHVTRIRPGALPPKPSNDLNRLGHPPLGKKEPKHFGRFDVFDDDPEQDILDSCNGTRDIKQKHYDEEKEILRKDDQKTGRDYDIVADTDQIIEPDNVEEKVSEQEYANQKSNQISIMEFNDDAQEDRGIQPTGKYVDEDTGISQPSKPPKSSNGPSTSSDHKPQKELSEEKLKGQAHETSSRKNASIQTSTSLLHKSASVSDWLLERKNTGPPLSSQTPTMPSGLNAISVVPQLQTLFSQTVIQQELIVNLMNSLNSSEQPTFQKSSSFSSKSSSRNMDSTVEISIERERDLLSQVAELQCRITVLVEEVQAVKWKNVQIELPPIGALGVKAPLAKGALE*

>Azfi_s0158.g053902

MTTGVLDATSAPAPASADAPLADIEARFESLKTIGKGSFGDVKKGFDKELGRDVAIKVIDLEEAEDEVEEIQKEISVLSQCRSPYITEYYGSYLHQTKLWIIMEFMAGGSVADLLHTGPPLDEGSIACILRDLLHALDYLHSEGKIHRDIKAANILLTLNGDVKVADFGVSAQLTRTMSKRKTFVGTPFWMAPEVIQSSDGYNEKADIWSLGITAIEMAKGEPPYADLYPMRVLFLIPKNNPPQLDDHFSKPMKEFVSLCLKKNPAERPSAKELLKHRFVKNVRKSSRLLERIRERPDRQFESSEDLSTSERTPFEEATIKVIPRKPPISSTWDFGTGTGTFQSTGTVRSMKPAMPELDSESASPQLTRVLTDNKQEWQLPSSSLSPLRISNGNGNINEYIVDEQGDGLKMVQDVHVSKSISNAYSSSLQSSSGHSSPIASADSPGQETGLIASTEEILGTMFLRNTHDGISSGPETPRSQDTLQEKPSQTMFREDSATNLADAKAALEAGSRSRRHPPSKLRVEGSFKTVEQSSNSEIPRDIQDARTPPKLASRLAVRRGSEDEDAARTAAAGSSPTLALLLIPALKETAAEQSEGPALRAAADAADSLMDLERLAPGACEVLVSKLLHRLGSSKEPAVKALQDTAEKVLSQKQSSQTKEEAGVNHTSKQTKGHPGSDPVEKSGLSPLAGFLLTRWQANVSRDMNTQR*

>Azfi_s1840.g107359

MEQKVNKKKYPLSASSYTLLEEVGQGASALVYRAKCITLNEIVAIKVLDMERCNSSLDDVRREAQTMSLISHPNVLSAFCSFVVDHNLWVVMPYMAGGSCLHIMKSSYPDGFEEPIIATVLKECLKALEYLHAHGHIHRDVKAGNILIDAHGNVKLGDFGVSACMFDTGDRQRARNTFVGTPCWMAPEVLEQVHGYDFKADIWSFGITALELAHGHAPFSKYPPMKVLLMTLQNAPPGLDYERDKRFSKSFKEMIAMCLVKNPARRPTAEKLLKHSFFKHTKSPEYLVRTVLDGLAPLGQRFQDLKVKDAERLAQKQMPFGEKEERSQTEYKRGVSSWNFDIEDLKAQAAAMQDDDGCFATGKEDAEQMEASTTMSDTVDDGHGPITLHSLSSENVPDAGGPFTRVRSGPLPLGLQSGSEELIESCAARDDEELINKGSLLTDTLSIQQLAGDPKISEADSPLRENKGESKKVDKSLPYSGDVKREDKPFQNMEERDLRSEKVVQEGRRASSGPLVPEHVLAMHRGREEEKDSGRLSFRGGGDDDTPRARFQRERAYSGPLTMHGTSFDHRFMNGFSSTSKQIAGIIGSKDVPDKGLYVQKKGRFSVTSADVELMEDHPATMPTRRSASMQVLQSSPASPNPTNPIPNTAVLACLQNLLSHTSSQQELLVSMINNISRGEPLQVHGVPDSSAMKQNYSGSGARSSDSLEVEISSDREHELLQQVSELQNRLVSLVDELQTQKLKNISLERQLNALYNKKEEERIRREDEADED*

>Azfi_s2076.g108990

MEQKVNKKKYPLSASSYTLLEEVGQGASALVYRAKCITLNEIVAIKVLDMERCNSSLDDVRREAQTMSLISHPNVLSAFCSFVVDHNLWVVMPYMAGGSCLHIMKSSYPDGFEEPIIATVLKECLKALEYLHAHGHIHRDVKAGNILIDAHGNVKLGDFGVSACMFDTGDRQRARNTFVGTPCWMAPEVLEQVHGYDFKADIWSFGITALELAHGHAPFSKYPPMKVLLMTLQNAPPGLDYERDKRFSKSFKEMIAMCLVKNPARRPTAEKLLKHSFFKHTKSPEYLVRTVLDGLAPLGQRFQDLKVKDAERLAQKQMPFGEKEERSQTEYKRGVSSWNFDIEDLKAQAAAMQDDDGCFATGKEDAEQMEASTTMSDTVDDGHGPITLHSLSSENVPDAGGPFTRVRSGPLPLGLQSGSEELIESCAARDDEELINKGSLLTDTLSIQQLAGDPKISEADSPLRENKGESKKVDKSLPYSGDVKREDKPFQNMEERDLRSEKVVQEGRRASSGPLVPEHVLAMHRGREEEKDSGRLSFRGGGDDDTPRARFQRERAYSGPLTMHGTSFDHRSKDVPDKGLYVQKKGRFSVTSADVELMEDHPATMPTRRSASMQVLQSSPASPNPTNPIPNTAVLACLQNLLSHTSSQQELLVSMINNISRGEPLQVHGVPDSSAMKQNYSGSGARSSDSLEVEISSDREHELLQQVSELQNRLVSLVDELQTQKLKNISLERQLNALYNKKEEERIRREDEADED*

>Chabra1|335919|rna-CBR_g32298

MDKSAAVKEYPTRVEDYDLQEDCGRGEGAIVYRALCLPLNEAVAVKVIDLESCSSNLDDIRKETQTMRLIDHPNVVKAHCSFVEGHYLWVVMPFLSGGSCLQVMKAGFRDGFDEAVIATILKETLKALDYLHKKGLIHRDVKAGNILIDGNGAVKLADFGVSAYMLDSTGTKLKPRQTFVGTPCWMAPEVMEQRQGYDFKADIWSFGITAIELARGRAPLAMFPAIKILLMTLEHEPPTLDDDDERKYSKAFKEMVASCLVKDPQKRPTAEKLLKNPFFKQARSADYLVRHVLQAVPPFSLRSMSTTTLKAPPPKDAGCTADPNQKAKRDSMATKMECKNWNFDLPPSTSSSANQKSPISLSPPSPRGRDATNATSSGTPRGGSSGRTTTTTGSNSANQCDCGGSGIISRNGSSSTTSSTTTNPCNVCGRSRSRAGLAASGAGGGPPEYGTAAGAGHDAANCGGAGAGTGTRAPAAAADGYSGRVATTGSGSGSCSSCTTYGSCRSCSKSSDCSSGSTNSREKIDKSPAPSPRRDQGAPMSLSVPKHGGHRNPSPAPGPSSVVSDGLNSNCDSRPSSARGQNKGEKRISSGGGRGLSRRGSL*

>Chabra1|337629|rna-CBR_g30118

MEEKKGLPTNAKDYRLLQECGSGVSATVYRSLCIPLNEIVAIKVLDLEKCQSTLDEIRKEAQTMSLINHPNVVRAFCSFVVDRHLWVVMPFLAGGSCLHVMKSAFPEGFEEVVIATILKETLKALEYLHRHGHIHRDVKAGNILIDNSGQVKLADFGVSACMFETGDRQKSRQTFVGTPCWMAPEVMEQIHGYDFKADIWSFGITALELAHGNAPFSKYPPMKILLMTLQHAPPTLDLERDKRFTKSFKEMIASCLVKDPSKRPTADKLLKHPFFRQARSPEYLVRHVMQGLPPLVDRVRALNEKKAKAAAQKMPMEREEEESQEAYVKGVSGWNFSLEDLKREASLLKDDDDEGPGTPSSDEGGRPGSPGVRGGSSPRRGAGMGSSGGGGGGASAGSAAAGGTGATRGTAGTGVGANEPGGEGGSDVIPYSISTPVGAISSTSAVSVQAQASFPSSAASSSSSSSSTSSSLAQRRSSLSPTYDEDKDWEALGLSSEFLASKQERDRERERERERERERERERERERERERERERERERERERERERERDREREREIELRQPRSSAIAIMNGPTKFASRGPGGGVGGVGGGEPMYLPGGTPPPPGALLRSSKEPSGSGRHQGLLSSRYDSGELMNGGGEYSSSSGLRVERERSSFDRERNSFERERDALPSPKAPSPQPIGGKFSRSSSLIFDRERDRESMRGGGGGGLGGYERNTESLRSSSFGERERERERDRERPSSANGNYAPVSRPVGGGGATSASGMTTSSSSSSLNYRGRFVEELVDAGPTNIISSPPNSSSSKRQAAAPAAVMGGPVAGKSQLMRKSTSVGDWSDRRLLQQSGQSVTAAAAVSYSPPNAGQPLAPASAMNLHSCLPSSTTSASAMAGGSSGGSASSSISSSSSSSSSSSSTLPFNSAYGATVPVIAIMPHLQEVMHRVLTQQDILLHVMSCLGSNDSTTWKNKIATREPDQVDVTTEREKDLIRHIADLHNRIAALSDQLEMYKSKNALLDQQLNAIRNEEEEKRIQAADMM*

>Chabra1|339814|rna-CBR_g37697

MEVEVSTFVVLGERPRELGSVFRCFLGLDKGVGGLLSSVEFAKPVYRVPPPQEGRYEIVGMIGKGSFGDVFKGVDKETGQEVAIKIIDLEEAEDDVEDIQKEIAVLSQCRSQYVTEYYGSYLHNTKLWIVMEYMAGGSVSDLVKTGPPLDEFSIASILKELLQALEYLHGEGKIHRDIKAANILLTANGDVKVADFGVSAQLTRTISKRKTFVGTPFWMAPEVIQHSDGYNEKADIWSLGITAIEMAKGEPPYADLHPMRVLFLIPKSNPPQLDDHFSQPFKQFVAACLKKNPAERPSARELQKYRFVKNARKSPRLLERLRERMEGGLTLENRELPGTVSMGGTISEVMMRSEGSLTKPVAEAVQSLAHSHPSSDSTWDFGTGTWKIGGRQQAVGGESEEGLGAGGVLGGRGGGSDTRERSGSQTSTDSGSTADSVSDNSKGSDQSVVVGGAASGQDGSPSSKGEGIDYNVSDDDGGERVAVSEMGTMVIVKSLKSKTSAAEALAHLKNQPASRGMLPPVGPAKFSESQSMGRIPRTSSSMSDEDPAGSSFDFSSDIGDTVVVKKNESPFVRRSMSANVRVEPQGPPAPFVQEDSATNLAEARAAMAAAGAKGGKKMGKASSMKPLLPRKPSEESAPKPDASAVTSPALSSLLIPALKEIAADRAEGGAAIRAASDALEALTGLERVAPGACEALVGRLVDKLASTEDHAIEELRQQARLALLARGSRGAGSSLAGENGEGNGRGCDMLWMGVDRGAGRKLDTAGLSPVASFLLKRWQLQMSVRMTGDALRL*

>Chabra1|355109|rna-CBR_g3422

MDNAGGMKAYPTRAGDYKFLEECGHGVSAIVYRALCVPLNEIVAIKVLDLEKCSSNLDEIRREATTMSLINHPNVVRAYCSFVVNQNLWVVMPFLSGGSCLHVMKAAFPEGFEELVIATILKETLKALEYLHRHGHIHRDVKAGNILIDSNGAVKLADFGVSACMFDTGDRQKSRQTFVGTPCWMAPEVMEQIHGYDFKADIWSFGITALELAHGHAPFSKYPPMKVLLMTLQHEPPGLNLDRDKRFTKSFKEMIASCLVKDPSKRPPVEKLLKHPFFKQAKSADYLVRHVLQGLPPLGERVKALKIKDQARMAAKMMPLGEQEEVSHNEYKRGVSSWNFSIEDLKQQAKDEEEDGGSSQAPSSDEGSFGRGAGGGGMVSSKGGTSAGGGSSRGTRGSHSDAESDPSPSMEGSPRQAQHRTHAMMSSTVHPAPLSTASAAAASALDGDKGEKGLMAGAMDSRDGGGAGAAAPAGGGGGYAGAGPMRGRTSRGPTPPPMQNVQMLIIGGESQNGHKFLSGNGGGGGGLFSPKTGEVQQQHQSMYGGSGGGGGGGSSTTGVIVVRKEGVVKHVGRFDVEIAERTSPPQQQHQPGGAAGAQRSIVMERLEAVTVTSAVPIGGEVGERSSRHSSGLERVAIENRTALEQREREHRGSLGNLSFFDKDRDRPLANGVPSSSAAGAGGAPRGAAASAGATPSKDAEQSQVVRKGRFSVTPGNVEDVESTLPIPPKRPNAGAAVKAAQQAPQSSQVHRSPSINDWSAQSGGGADRRSSLNPSSSPSPPIVNHPAATTASAGGGGGLLVPVVNVLPHLQNMMHHVICEQDLLLNLMNCLSANDPSALCRSKVSSMGSRESAEHGGDVTDRERDLLRQVMELQSRVAALTEQLELVKGKNVQLERKLNTIFNEEEERRIHAEERDRSPDND*

>Chabra1|356485|rna-CBR_g2836

MKSPTGSEKDVEFEDPTMRYELLNELGKGSYGAVYKARDLHTSELVAIKVISLSEAEEGYEEIRGEIEMLQQCNHPNVVRYLGSYQGDDYLWIVMEYCGGSSVADLINVTDEPLEEVQIAYICREALKGLAYLHSIFKVHRDIKCGNILMTEQGEIKLGDFGVAAQLTRTMSKRNTFIGTPHWMAPEVIQESRYDGKVDVWALGISGIEMAEGLPPRSNIHPMRVIFMISREPPPMLEDKEKWSLVFHDFVAKSLTKEPRSRPTATQMLQHKFIDKCKATAACMLPNGGGGGGSNGLAGGTLRAAQTSISGLQDKMISVYAAGNTIPIPFLRATDVSPIALLSPGNNPRAFGALAGNAIKTPEHSGRIALDALQQLYSSGGGIEGGGLRRGKKPMGNEPPLPPSTYRRLATSSTLGNLARAMAYHKHCADELPLLNWQLQQEEQIINNLSDILRTILRL*

>Chrsp31S04792

MALHPMPSAGSLTLGSASDGRKMYPTTADAYVLLEESGTGVSSVVWKAKCVPLNETVAVKVMDLERCLGSLDGVRKEALTMSMINHPNVVRALASFITGHLLYIVMPYLAAGSVLHIMKYAYPQACHRCHGLKEPVIATILKEALKGLDYLHRSGNIHRDIKAGNILVDLDGTVKLADFGVSAALAEGADRKMQRQTFVGTPHWMAPEVMEQVHGYDWRADIWSFGITCLELAHGHAPFSKYPPMKVLLMTLQGAPPTLQQEGHKGRFSKDFREVVDKCLVKDPAKRPSAEKLLKMSFFKHAHSSDYLVKALLEGLPSLGERVRRQKELVAAMRGGPPEDEQQKSLENYQKGISGWNFDLDDLKKEAANLDESGELIERHPATIPEEPADELQAAQSAQLAQLAPPPQAFDAARMLPPGVLQGVPQGMAPRRAPTAPSPPVQVAKAPQHRGRFDIYDGPLLSPESHDDRAEVAMQAMSQAASQGVRRGRFSVTSDDQPSPSSPTLKSSTSLHKSLSVNDVPSRAPLPQARSTTPPLVASSSVRQAEYDSQAQAAANLSHQSSMPPEQIMPQLQSLLEASAQQQQTLNTLIGALTSGTGVAPGADGHPIRQLPPLHFDTQHMLAMRGRAGMFDVHNELELLQRIQELQQQVASLMEENQSMKQRNVELERRLNAMLNEKLAQEDANND*

>Chrsp6S00594

MGSADDGGGGGGGGSVVGAPLSDQDPTRLYELLQPLGKGSYGAVYKARDLRSSELVAVKIINLGEGEESLDEIRGEIEMLQQCNHPNIVRYLGSYRGPDYLWIVMEYCGGGSIADIMHVREAPLDEAQIAYVCRETLKGLAYLHSIGKVHRDIKCGNILMTEQGEVKLADFGVAAQLTRTMSKRNTFIGTPHWMAPEVIQESRYDGTVDVWALGISAIEMAEVAPPRSNVHPMRVIFMISREPPPMLQEKEKWSLTFHDFVAQCLVKDSRLRPSSAQLLHHKLIERCKATAACLQPQIDALRQAMAEHAARLLQERGTSSSTGQLSWVGTSNQMTVDGRGGTVRMTPGGTSASGGSYVDEEYATFVMHRDSGTSTGTVRIMSAEQRERLRNDAPMFPHELMLDRALPPPRYTITARPSPAPSPHPAANADRADVPKSDKGMSEVAARLFGGNKPTHAASAAPPPPLRQMDMNGTVKVLDSYTATQLHSYDNSRVPSREQEMGSLRALNTALYDKLASVHAAGSTIPIPFLKASSIPSAAMLRQDPNVGEDSSAIAAVKELYYSAYGSTGKKGPNPELPQSVKQLLSRGTLPNLARALSYHKQCIDDLVLTPAQLAEHQHVVSDLSDTLKTILRL*

>tr|E1ZJW9

MAGSDGGPAASPASRYELGELIGKGSFGTVHRGYDTVAQMEVAIKLIDLEDIEEDIAEIQREISMLKQCQSPNITAYYGSAVVPGTSQLMIVMELLAASAADLVSEETGGEPLGEACIAFVVREVLRALAYLHSQHRIHRDVKAANILLSPEGAVKVSDFGVSAQLGGTVGFKRRTFVGSPLWMAPEVIEQSPDTEGFRGDAGGAPPDGYDEAADIWSLGITAIELAEGEPPRAHLASFRLLFMIVRDDPPQLEGGQWSTELKDFVWQCLRKDPSARPSAEDLLEHPFVAGAQQPPELRERVAAWLQRRPALLERQQARVSGGGGFGTVGATMPRWDFAAGGGGGGGNGTLAAARPVTAPGAGGMQRTAAGAADLGGTLVRRHTDAELAAADYGGTVQVRPAGDAEFGMPVAAAAPGGLAAAAPPSRLFIPNDPTQPDQPLAAAAGGYLAGPASAVGGGGGGGGGDGGATRQLLQAGLQAAAGGPGAGPGAAAAAETASAALGQLEAARPGAVRDALTEVLTQLSLASSPSLAALQGSASALFGDGGGAAGVGGQPGDVPELGPLGRFLMSRWREAVARERVLHTQQWQAS

>tr|E1ZPY0

MAPSQQSSSPARRRQGSEADAYSITDPFKIFELIHPLGRGSYGAVHKARILSSGDIVAVKIIPVTEQDEIASIQKEIAMLRECSHPNIVKYYGSWRTRDALWICMEYCAGGSVSDVMHTRGGGLDEDMIAYICAQTLAGLVYLHSMGKVHRDIKCGNILLSEAGEVKLADFGVAAQLTNTMSKRNTFIGTPHWMAPEVIQVSQYDGKVDMWALGVSAIEMAEQFPPRWRINPNRVIFMIVKDPPPRLADKERWSLNFQDFVAQCLQKDPRTRPTAKYLQQHKFTSRDAVTTVAVRALLPLVQQARTHMAEMALMAGIEAQQAVPAAGNAA

>tr|E1ZTI4

NYDLVEECGRGVSATVYRAICKTYNEEVAVKLLDLENMNCSLDEIVREAQTMRQLNHPNLLPLYCSFVHKEHLWMVMPYVQGGSVLNIMRFAYPDGLEEPVIATIMKDVLKALEYLHRQGIIHRDIKAGNILLDNGGQVLLADFGVAATLERGGSWGNRMMSRNTFVGTPCWMAPEVMEQSQGYDARADIWSFGITLLELAHGHAPFARLPPMKVLLMTIQNPPPTLDTETSKKHFSKAMRELVAKCLVKDPTKRPTAAQLLDNKFFKTAHDNQYLQKHLLVGLPPAPERVQLMRQGHAARQEVQDKDILASQQEYRKGVSSWNFDVAALKAAAAAAREDEEDRLPPISEHSGAPPGWLAGQLGGWCAGWLGHGRGTG

>TnS000331967t03

MDWRSGKKKQQQDEQQQASSDLYSTFVYKADEKKPYIYSTVVIHGDSDEDAAINSRKKKKSSTAAAKGVDDIYSTMILNDHKDDADAEDGDVEDDDEPSLPPLLRSLPKDFGGASEDADDDMYATSIAKPTSSAPPPLPRPVPSLAFGRFGKPRRYPIKEEKKKEVEDPYTSFRNRSPRGGVDSAYATMIQKDDVYSTMIMKDDIYSTMIRKEETFNSVAEKAEEDEDAFSSVVHSRGKQDDDDVSTNFRKAVESMQSGLAAEDAARRHALERKRENKKRSGSSGVLDDVIREDPSTKYELLDELGKGSYGSVFKARDLRTSELVAIKMISLSEGEEGYDEIRGEIEMLKQCNHPNVVRYLASYQSEDYLWIVMEYCGGGSVADLMKFTDEPLEEDQIGYICREALKGLSYLHSIYKVHRDIKGGNILLSEQGEVKLGDFGVAAQLTRTMSKRNTFIGTPHWMAPEVIQESRYDGKVDVWALGVSAVEMAEGLPPRWDVHPMRVLFMISSEPAPMLEDKEKWSLVFHDFVAKCLTKEPQKRPTARDMLRHKFIEKCKSTASSMLPKIEKARQIKLDMANQEYNIGRGTSASGGGTVKMNEPYVGTTPSKVNKGAHPEIHAGITDSSGAAKEEEFGTVVVHQTASDKIISNIQTKSIESAAVVTGPMKSDATAHTPSTVLHQGMANKADSSLSSSSFTGSPFQNRTLASKKLQGDDTEWQSAPTTVGGTLRSSGTISRQGFALQDKLLSIYATGNTVPIPFLKATDISPIALISDNLDGEPDHSGAIALEAIQELYNGGILGDSQLQKGRKPANNEKPLPPNVCQRLATSPTLLNLARALAYHKMCYEEMPLQEWQVTQEQKTIQNLSDTLKTILRL*

>TnS000076667t02

MSNSSGISGIESRFLNLELIGKGSFGDVFKGYDKELNKEVAVKVIDLEEAEDEVEDIQKEISVLSQCRSPYITEYYGSYLHETKLYIVMEYMAGGSVSDLLEIGPPLDEGSIACILRDLLHAIEYLHTEGKIHRDIKAANILLTANGDVKVADFGVSAQLTRTVSKRKTFVGTPFWMAPEVIQNSDGYNEKADIWSLGITAIEMAKGEPPYADLHPMRVLFLIPKNNPPQLDDHFSRLMKEFVSLCLKKNPTERPSAKDLLKHRFIKNARKSPKLLERIRERPKVHIRKVRETQKLETLNAETVSEKVNSSKYASETESLPYSHRNIPKNASWDFSTTGAPATGTVRAVKPPNISSRDRKLDSVAGQEARRSLSGQENEWHGVSTSASLIGDASSDGDVLDGLSSIHERRRTPSSEDEKLSVSSSGTVIVRSPRDSRAASGPQSQSTSLFSGNISGEDVSIVGTVLVHGKQDDVDSMPRTPKSRFGHQEKLSNAFPEDSATNLAEAKAALQAGMRKFNQRDRYAPPKSHMNGHESKLAERPSLAEPTRETRDPWEGVKVPVKPFSRRASDDEDARSAAATSSPALSLLLIPALKETAAEQSEGPALRAAADAADSLMDLERLAPGACEVLVSRLLQRLGSTKDPSVKELQEVAKTSVTKDASQLNGSGKSTQLKNEKNPENPENSGLSPLATFLLSRWQSNIIGGTNSV*

>TnS000022753t07

MADGREYPTSASGYRLLEVIGRGASATVFRAKCLPFDEIVAIKSIDLDRSNINLDAISLEARTMRLIDHPNLITAHCSFVVDNFLWVVMPFMAHGSCQHIMEAAYPQGFSEPVIGSILKQVLNALNYLHEHGHIHRDVKAGNILVDTVGEVKLCDFGVAAYLFDKGNLQHSRYTVVGTPCWMAPDVLPDGKGYNFKADIWSFGITAIELAHGHPPYSNETPQKAFKEIVDICLERDPRKRPTVKQLLKHPFFKKAKSLEHFSMHLFTTLPPLWERVKAMEEGSCIRISAHPIVILEDKEHEYET*

>TnS000947333t01

MEEIFPLEPSGYEVHEQVGHGVSATVFRAFCKSLKKIVAIKVLDLDRCNSNLDDIRREAQTMNLIDHPNVVRAYCSFVVDQHLWVVMPYMGGGSCLHIMKLAYPNGFEESVIASILREVLKALQYLHGEGHIHRDVKAGNILVDINGIVKLADFGVSACMFDTGDRQRSRKTFVGTPCWMAPEVMEQKNGYDFKADIWSFGITALELAHGHAPFSKHSPAKVFLETLRNAPPGLDYARDKKFSRSFREMISMCLVKDPNRRPSAEKLLKHSFFKQARSNSYIANEILHGLPPLVERIKILKEKDDDRLAQEKTPYGIEEEKSQNEYKRGISGWNFDLQELKSQAALIKDDDLVDSKMMEHKSLTQNSQEHKQTDSSLLVTDISYKGNSQEKEMNQSSESQQDPLSTSFSLNILNDSRKQFDLGKDDIHQKCSMIIEVNEKGNVIMENTVSSSIAGTIDDIRKTQQCLPPRWIAYSGPLISSVAKESSIIENNRGHKAPTNDIDDKAKKLSIQQKGRFKVTADSAEAETNGNANLHRRKSFKNVSQQLPPHPTASLNLERPTIPVSDILPFLQTLLQTNLLQKETIDHATRLCYDEALLGDASAKYLPDGGAGGSMDPTNREIELMLQIVDLQNTISDLTADLQSYQVKNEQLEKQLNSHTRKKNEE*

>TnS000060165t02

MEHRYEKRYPTSKDDYELYEEVGQGVSAVVHRALCRPFNEIVAVKILDLERCNSNLDGIRQEVQTMSLIDHPNLLRAHCSFIVGQHLWVIMPYMSGGSCLHIIKSCFPDGFEESVIATFLRETLKALEYLHSQGHIHRDVKAGNILVDRSGAVKLADFGVSACMFDSGDRQRSRNTFVGTPCWMAPEVMEQLNGYDFKADIWSFGITALELAHGHAPFSKYPPMKVLLMTLQNAPPGLDYERDKRFSKSFKEMVAQCLVKDPSKRPSSTKLLKNHFFKHVRSNDYIVRTVLHGLAPLGQRFEELKKKEKERMEQNKMAYEEKEERSQSEYIRGISAWNFDLEDLKAQAALIQDDDEVPAPKEHLSDSLIPRGNKESAPMTPNLDFFGSVHLGRKQSLEKDGDDIMPVLGNSAALPNQIRNGPIQLLDDDDVNSDGPHWKEYMKSQFNECVASQHRKDDNQYVKDKRHELERDPRLMEDYRGRRVLSGPLNPETVLASSRGVSGDNERDKLQRRLQQRNFSGPLVATGQKEIDSFCDPSRILGNLDDKMKGMVIQQKGRFKVTSDEIDPKETIGKRRPSLTLLNSSATVSAASILPTLQCILEQNKMQRDQIINLIKHVTQGESLFACSSSAADLPWSNTLEKVEAASERELELLQIIADLQQRYSVYHFYYWQFYVHDNFCVVLYIYIFLTESQVLLMGCKMRR*

>TnS000821935t04

MDQISGSSSSKRYSLNEGGYRLLAEIGRGAHATVYEAECSSGEGVARGEHVAIKCVNLDGAATVLERVHTESRTMSLLSHPNIVRAHCSFAASQSQSLWVVMPLMAGGSAESALRCRRAPLDEPLAGAVLAQALRGLSYLHAQGHIHRDIKAGNLLLDSSGTVQIADFGVSASVFQAGDRLLRRTFTGTPLWMAPEVVDQAGAYDMKADIWSLGITAAELVQALPNKKAPAAMFLQARATGVAEMINAGLSSRKFSKAFREMVSLCLQRDPARRPSAEKLLKHAFFRHVKGREFVVKHLLENLPPVAERARLCRPWPLLGCPADAEDEEEEEDEEDKASMQRCRRISGWNFDEDDQRCNALDRSHGSADAAAAESPNVLKFFPQQRKHPRPVH*

>TnS000010783t01

MMAPEVLQPGGGYDFKADIWSLGITALELAHGHAPFSKYPPMKVLLMTIQNAPPGLDYDRDKKFSKSFKEMVAMCLVKDQTKRPTAEKLLKHSFFKQAKPPEHVVRSLFQGLPPLWERVKMLKNKDAQQPLKKMSFTDEEAKSQEEYKRGVSAWNFDLEDLKTQAALIQDDDDSLGSKEEDEAHQNIEGNQSLAQNLQKNKYEKNHMEKIKSSSTNDLRGLADADNHSKQETNKIEHFDNGNGKVDTLVDENLQDIGHQVPSKVSEKEQRVLNDRQKNDGMNLPRPQDVEQIISKSKSPQRQCFSGPLVVDRMVSVRNSERDRNGRKLDETFKNDLSKDPIFAGPLLLPKHATANSLSAPVKSPGGFKEYQDDKSKGNVIQKKGRFSVTSEDVDIAENVPLPSAIRRSQSISLRKSASVGEWLSEQRQGAAAQLPKDSGNSTIPVGVLSACLQNLLQQNVTQQEQIVNLMTSVYQAEGNSSCQNLKKICSTGRNGALECLAETPEEREQILLTKIEELQFKVQSLTEELNAVKAKSLELQQQLNAAVLSSEEEKIPKSQKR*

>TnS000113885t01

MEFYRVNQKCFGKGLSPWKVASRSLKKKYMDTLINLSSCPKKHYPVDVSGYVLGEEVGYNFATNVYKARCIPFNETVAIKIIDLEKCGADLDEISLGIRIAPLLNHPNIMKVHCSFVVEHYVWIVMPYMTRGSCLHLMKASFQTGFEEAFIAFYLQEVLKGLMYLHEQGQVHRNIKATNILVDSSGLVGLGDIWFSTRNRNTFWTAPELNQQTNVHNPKVDIWSIGVTALELSHGPEALKENSPIKVLDIAMHKGSQGNSDGRRKKLSKSFVEMITQCLAKDPMRRPSAKRLLRHTFFSKYSLSSEHVSGALMCLPLLGDQIKAIKLKEGIMHGSQVFLNGEKEALSQNGCSRINSGWNFELEGSDIVSFREEKLSADAVSEYAETTDNILLESLEPLSSQKVADAYIEPANCIANEAGHYIQRKNNAEKDNLVSSKVIKVQGMGEKNTAQYDHQPSLYTDVNASSKYLREDMSNSLGNDDRSYSYSIDAEVGSSSMQSTSKIDDHKIELSKIKELEHPEKHFEIQEDIQFGPLQGENCESKERLLKDQPEHLEKQLDIQEVDQSISVNDEIIENGDYPSEDINQTDINLGIQEVNRPVSVYGDSMDKGHSMENQQSHTENMISDREKLMNDNDLNGYSSMENLRRSSSIGILEGQDSNQKLKAPVVEQKGRFKVTTGDADAEKICSAQNTSVSCSTSSSSIPMFSILPFMQKIAEHNDTQRDQLMKLIKTLGQSNSSPGLQNYMYPAKSSVRNSHQNSTSRVTSERERELQQNLEELENRALFLASEIEKGKLRNMQLEKELKTIYEKEENKHMITSVPQDSV*

>ME000247S04224

MEEQYELNELIGRGSFGDVYRAVDRKTGKEVAIKIIDLDDTEDDVEDIQKEIGVLGETRSPYITEYYGSLVSETKLWISMEYMAGGSVFDLLDDGPPLDEVSISYVLRDLLMAVEYLHTEKKIHRDIKAANILLTAGGDVKVADFGVSAKLTKTVSKRKTFVGTPFWMAPEVIQNTDGYDEKADIWSLGITAMEMANGEPPFADLHPMRALFLIPKNPPPELEDHFSKPFKEFVGLCLRKSPTERPSAKELLKHRFVKYARKSAKLVDRISTDGSGPTAGGGKGRHARNYSWDFGSGTIRGSGGTVKGPPPGIPSMPEAGGADVASQPGAGKGGSSIDGRGDTGASSPLASPHSFASAPSSFSSSSHAGGEAGGHENGQQPPSQQQQVGAGTGTVRGPRGGAGSASADSTPYGTLERDRDRERGGRGVESGMGTVKAGSLGLPPGAQSPAAGSVRSVRSVRTIGRRPFSQAAGQGGEAGVMYGKGWGNEAEAAGSSADEPSDPGNADGKEMDGDRGLVDDPALQPEDSSTNLAEAKAAMAAERSKRPPRREESSADGSHSRTNGVEPPRSGHRAPPKAPPPISRRQSDEDRLAKMAQLQQAASPASPALSLLLLPVLKEMATEQAEGAALRGAAQVADALADLEHAMPGAADVLLTRLIHRIASTEDPALRGVRLQAAKKLSPLSSELPSSGFKKGEEGSSRPDAADNSGLSPFASFLLHRWKAQSMDS*

>ME000293S04947

MGNSSEDGYDEIRSEIEMLQECNHPNVVRYFGSFQGEDCFWIVMEYCGGGSVADLMNSTDEPLDEPLIAYVCRESIKGLAYLHSIFKVHRDIKGGNILLTDSGEVKLGDFGVAAQLTRTMSKRNTFIGTPHWMAPEVIQENHYDGKVDIWALGVSAIEMAEGLPPRSNVHPMRVLFMISREPAPTLEDPEKWSLLFHDFVAKCLSKEPRARPLAPELLEHKFIAKGKGTAALMMKRIEASKSMREELRKEALAEQLAADLAATSSTSPTLSGRPFNRPFFLLGERVSRVQAAAAVAVGVAAAGKQWGPHWRPLLLPLALPLILLVVLVVMVAVVVDAVWEMGGTLPRGGAPEKPSRQSGAGGTLKARPSAAAAASVSDDDGDAGSEFGTMVVRGTGTLKITKNKDTGAGAGDRGGGGGGEAAPPLGAAAASGGAGRSDGTASTRGADEDGDRDADRSGVLPPKRQTSLPSVKPPLPRLKSSLSGLPPLFEGQVLRSPESASGVVPGVALHDKLLAVYAAGNTMPIPFLRASEISPLAFLGGLSDDGRSGGVSPNQAALEAIQELYRGRGPQDVSRRKLRLSTTSDVPLPPSVYKRLGGSSTLPNLARALAFHKQ*

>ME000110S_11048

MPHSQVTHPEPLDTRKWPTNAADYMLLEEIGQGGSAIVYRAVCSPFKEVVAIKCLDLEKCNSSLEDIRREAQMMSTVNHPNVVRAHCSFTKDQFLWVVMPFMAGGSCLHIMKSHHPDGLEEAVIATILRETLKALEYLHRQGHIHRDVKMMSTVNHPNVVRAHCSFTKDQFLWVVMPFMAGGSCLHIMKSHHPDGLEEAVIATILRETLKALEYLHRQGHIHRDVKAGNILIDTNGSIKLADFGVSAGMFDMGDRQRARNTFVGTPCWVPATPFLAGNILIDTNGSIKLADFGVSAGMFDMGDRQRARNTFVGTPCWMAPEVMEQLHGYDFKADIWSFGITALELAHGHAPFSRYPPMKVLLMTLQNAPPGLDYDRDKKFSKAFKEMIAVCLVKDPAKRPTADKLLKHSFFKQARSPDFIARSILTGLPPIGDRVRLLKDKDAQRLAAKKMLESEKEEISQVEYKRGVSSWNFNVDDLKKQAALIDDDEGEQKGPQRLPTVIDSEPTPAATPRPSEEDRSAATPVPGSQPSTRAASRRESDDSGWAAAAALPAAGGASTSSSTALPDGSGHGGTAAAAQLLASAAAAVVAAAAGGTDASDRSSSKIRSGPLPQGNQKPAPSKEKDQQQQQQQQYKSSSKSGPLDASKVVDDAGGGGGGHTLSAARDSRERESRRSQTGISAGAGPSGGSSSSQQDRATANGPLKEGTGGEEKKNAPPVLYKGRFHVTSGDEDVVFNLMNSLTPSDGALRVGAPGSKPGLFSRISSGSTDFLVLERQLNVFYNKEEEERIRREEAAKEDG*

>Mesvi1549S03163

MAFMNLAGLAMGKDAKDGVHSIISRRPDMDSGQNSPMHILAPSPSGVEVASSCSGDDRNEAEAQLQMYPSQVLQSPGGPSSDEDPSLLFEIMDELGRGSYGSVYRGRCLRTQEQVAVKIIPITEGEDGFDEIEVEIEMLQQCNHPNIVHYLGSYKAKDALWIVMEYCGGGSVNDLLHVDMQPLTEEMIAFICLEALKGLTYLHSINRLHRDIKCGNILLTDRGEVKLADFGVAAQLTHTMSKRNTFIGTPHWMAPEVIQESRYDGKVDIWALGISAIEMAEVLPPRSNVHPMRVIFMISRDPPPRLQDKDRWSLLFHDFVAKCLVKDSQGRPTAKDLLSHKFITKCKGPVPALLPIIARYRAAAEKARVAAALQRQQQEAADMSDHLEGCWGTGSDTLERGATVRRAALASDTYKAAGDTLERGGTVRTSAPRNVQTTFRGGGTVKAIQESDEDGVFGTMVVRPEFGTAKSKDDAGDFGTTAVRPESSGEGAGKDMSDVAARLFGGGGGGGSAKKEGSSAAPSSRAASQSGSVTLGGGSSYSGTISGTVGVRSNDEDISGTVVVRGREGGDTISGSVLRRGTVADRGTARDGFAMAVQAMKRSVDDGGGAAAAGGGAGAKKDTAGYQSAVRQAALHEKLMSVFEAGCTVPIPFLRARDIPPLALLAGPPASLVSPSLSLGSGMSLSSSASSEHGDGGRPTTLHGPWVALQAVRDMYRATSATGMPAPATGSLNSTRAWAERTGEDPLPSNVRQRLESNPTLSNLARAIAYHKSRTNEMPLPPMQLRQQYEVINDLTDTMRTILRL*

>Mesvi1691S09237

MAAAPPGAPVIAKDGTTLTQRYELHEIVGKGSFGAVYRGVDRETKQEVAIKIIDLESAVATWQNVEIRVLSQCRSPYVTTYYGSYLHQSKLWIIMEYMAGGSVADLLESGPPLDEASVAGILRDMLCALDYLHTEGKIHRDIKAANILLTENGDVKVADFGVSGQLTYTMSKRKTFVGTPFWMAPEVIQHSDGHNEKADIWPLGIMAIIFNKLADIWSLGITAIEMANGQPPFADLHPMRVLFLIPKNEPPELDAHFSKPFREFVAMCLKKNPEERLPARELLKHKFLKNARKTQVILDRLKESASKKRANLETRAASLRQHPEGGPGRDNQLWDFDFHPAATIASRDMPRPKLPSDTNGNTPAATHTNSTDGTSSPLPPRHGESGGGASAASEQDLERGSTVAVRGEAAVKLAAAVSMGRALGGGPPVMAQPAAPSLATRELAEPQQQATVDGLSPGRAPVSKKLAAPPRWATRGEDLASPAAVGSAASSGRGIERSNSSSSDHLEAEATTSSVGSGVFPPSAATRTASFGTFLKSLELASDGDGSKRPSGAEPSASPRSLMAAIELESGSDASSPIAAVAGTAPGSGTPYKGDGDGGRPTLAWGEGGYGEGDGDMGLEDEEPGSLTEVFGPALDEMRRKNVQDGDYYRSLLATLRELEHVAPGSCDALMEALLTFLASSNAGCVQNLKELASSLLPSATREGNRRRTSVSGGTGNDGGGGSQPSSLRRGLLTVQNRAPRLDALALGGGGEEGSSGAAGGNVGSSVDGGVAGVGFNRGRPGQGAAVPLMPSAAVATGDTTALGPMGAFLLARWQAQVASNLEQ*

>Mesvi213S04226

MADAKGTPGASVHGGDLVFPTNPDDYQLLEECGHGASAVVWRAMCKPLNQIVSVKVLDLERAGANLEEVRKETATMSMINHPNLVRAHASFVHQSNLWVIMPYLSGGSCLNIMRTAYPNGFPEIIIVTILKETLKALDYLHRHNYIHRDVKVKGRWRKRGGGEARRSSAQAGNILVDDDGTVKVTDFGVSATMQDSGDRTKGRQTFVGTPCWMAPEVMEQLHAYDTRADIWSFGITCLELAHGHAPFSKYPPMKVLLMTLQNAPPSLDMEPGDKDRFSKAFREVVGMCLQKNPAKRPSAEKLLKHSFFKASAGQSPNRPGKAMAAFRWSLDDYLAWNSKHMCPAAIVGAKSPDYLKKHLLEGLPPLGDRVKQQQSKDPSKREEAQEATSVEEYQKGVSGWNFDVGDLKKEAEAISLESIEEEAKKLALQTDAATAKPTEKKGRFVIT*

>OT_ostta13g01940T0

MARGGASAAEESDSASDAQSPPMVYPTEAAAYELIEEIGRGVSATVWRAKCLKNDETVAVKVLDLEEHEPEHLDEIRREAQTMSMLSHPNLVKYHCSFVNETSLWVVMPYLAGGSALNLMKWSHPSGFDEPIIACIVKAACKALDYFHRNGNIHRDVKAGNILVDEDGTVKLADFGVSASCWGSGGRPRSHQTFVGTPCWMAPEVMEQEHGYDFHADIWSLGITCLELCHGHAPFSKYPPMKVLLMTLQNPAPTLEESAEDGRSFSRHLREFVGLCLQKDPSKRPTASKLLEHKFLKDAKKADWLAKTLLDNIPSLGERTQRLAERDARRRAESKVGEEQREAASAEAYKHGVSNWNFDMADLKAQAAALDDPVVAESEAVAAANALENMQLGDKPNVERKGRFEIITEDVAPERSEASQTVQRRGRFDIIDEGGARGSRSTSPTRASSPAGGERRGTAKPVQKGRFTVHSEAPAGMHLNTFADVVQSIGSVHGVLRDLYAQAQTQSNVVEELREENARLRARIAELER*

>OT_ostta02g03420T0

MSDADVLVDGRYRLLTRVGRGATGEVYRAIDVTKDVTTGLNQNQDPNAHVAVKLVDLEDAEDEVADIQREIGILAQCASPYVTKYLGSALLRGTSKLAIVMEYMAGGSARHLVEEIEHSGDSSSKGLGEGEISFITRDVLRALEYLHAEGKIHRDVKAANVLLTASAEARLADFGVSGQMTHTLGARRKTFTGTPFWMAPEVIQGGEGYDEKADIWSLGITCYELATGSAPHSDLHPMRVLFVIPKEDPPSLPPDGDFSDDFRDFVSQCLRKDPLERPSAEELLKHPFVARFDVAAIQTLQARVDKHIALTDDEEGAHDSASALAQKEANTGSSAPAWDFGDDSLGRKFTPPEAAVVLAAASAEVRSEHGGSAPSVYQSMGGTIRMAEPPSFSDRHTEDGVSDDLVSMDAATFGTTSSHSAHQRDKLDALLENTLHTPLMRSLIGPALETIQTRDDEDAHAALQRAIEALDELEIVVPGSLMTLVQSIVRELARSDDESLRTLKSRARNYFGATRGDSVNGLSTGGRSVVANYLIARWQRDVRSDL*

>Sacu_v1.1_s0028.g009859

MEAKLEKRSVTDARRTYPVSASGYQLLEEVGRGVSAVVYRAKCLPFSDDVAIKALDLDRCNSNLDDIRREAQTMSLINHPNVIKAYCSFVVDRSLWVVMPYMAGGSCLHIMKSAYPEGFEEPIIASFLKETLKALEYLHHQGHIHRDVKAGNILVDSNGSIKLGDFGVSACMFESGDRQRSRNTFVGTPCWMAPEVMEQSHGYDFKADIWSFGITALELAHGHAPFSKYPPMKVLLMTLQNAPPGLDYERDRRFSKAFKEMIAMCLVKDPKKRPTAEKLLKHSFFKHARSAEYIARTIIAALPPLWERIKEAARLAQKKVPYDEQEERSQNEYKRGVSSWNFDMEVLKAEAALNEIESVKKVAEGLEVNISALTPSITSCSDDVENIELSGTSNLIKDENENIGGEQVTEVQARVLSTTNASKLEDTVAWKQGPKHYGRFDVFEADPDQDNIASSAMLQSANKEIRGGNDFIPVVSEGERLEMDGTMTGNTKGFKVELKSGDSRRYKEDESSQAFSRTDCSDVREDSESHAARKVFDNSVRMPRLGKTMSFNGITHSSSSATDASLFNGVSGFSRPNGQKDDDKLRVPLIQKKGRFSVTAHDMEFQEAILSSFKFWCCCQGLQSSGIRRNSGMQSSLHKSASVNDGLVERRNLVGSVVSSQSSHTFSNAATIMPQLQNLFTQTMIQQELIISLMSSINTSEQHHEKISVYGSNGFGNKSFGRSNTSNSINSTGELLSDKERELIVQIVELQNRITTVFDELQAARSRNVQLERQLNAIYNREEEERTQREDSMSDDG*

>Sacu_v1.1_s0098.g019462

MPEKVFPTQAADYDLLEEIGRGAFAVVRRALCKPMNLLVAIKRIDLESCNCSLESVGKEAHFMSIVDHPNVMKAYCSFNVDHFLWVVMPYMDGGSCLHIMKICCPDGLNESLTAIILKETLKGLDYLHRQQQIHRDIKAGNILIDSSGSVKLGDFGTSAYMFGQGGGERVRKTFTGTPCWMAPEVMEQKHGYDFSADIWSFGITALELAHGHAPFSKYPPMKVLLMTIQHAPPTLDQERAKHFSKGFREMVKLCLDKDPSKRPTTEKLLKHPFFRNAKNDSFKIQKLLHGLAPLWEREKILRDEYKRGISCWNFDVETLKDQAALEEDDVLSAGEDDDLLGPSSLRGNDSLKETSQLGKNIKQLTLDTNEISGRFGVFEGDLELESPGWHEIVRQTKYVEDQHEGIKLPPIAEHMSTSTVDVKGHEAHDLSTSLMFSRDMDDEAVEEDFEIHDLKSSGKYVACILDEHEPISDVSKNMKVNFEHVEGKSKEATLLRKNSSSVITREMTHIQASSDISTRTTSFLVPQLKNLLNQTVKQYETLADMLTLISPEEAINGHSVSAVEGSLTKIELSAKDPESHDVVTELQNTVSSLTKELIFVKYKNVQLERQLNAIYNQHEEERMRHEEAEKENLYEYQSSQHGTLYMP*

>Sacu_v1.1_s0146.g023088

MASSRDRASAHYAYRDDDAESGIYSTVVIHRGPDSGSDNSLTGADGDEVDDGVAGPGHIEPGDIYATMLRRDGDDDVDSPPPLLQSLAKRFAGTDAFDVTDDPSDIIPNFPPRDDDDSSVVLHSAATSTEDATALFKGDDFSAALRYMQKEAFSRMRVRQDEQEETDDSFSTFVVKKEDERGRKSTLGLGSSHGEASELYSTMLHRTGDASATVVTKHGDNSSSISKAVESFKNENEAFDKKRGFHDSPRSVHEKSQRSRRRASSPAQDNVIREDPTLKYELLDELGKGSYGAVYKARDRKTSDLVAIKVISLSEGEEGYDDICGEIEMLQECNHPNVVRYLGSYQAEEYLWIVMEYCGGGSVADLLQVTEETLEERQIAYICAEALKGLAYLHSIYKVHRDIKGGNILLTEQGEVKLGDFGVAAQLTRTLSKRNTSIGTPHWMAPEVIQGTRYDGKVDVWSLGISAIEMAEGLPPRWNVHPLRVLFMIFREPAPMLEGKEKWSLIFHDFVAKCLTKETRLRPSATDILEHKFITKCNGNASCMLPRIEKARLIRLQTAPQRIPQDLDATVGVSLPQQWSWEKGNTVKMGEIYGATVLVKSDKTINSSDMEGTTIIRHDEVEYGVLSFHGNLLLLDSADFNTMVVHLDHPIDQTKEQVPSTQNDQSNLAEMPDSATREIIDSKVGSSSITTHLPNELSTPADLSESSVPQGTYASSSTSIQQNSVPEAREQVERGEPSIVPVPIVTPRGLHAGGRQVFALRDKLLSIYSGGTTVPIPFLRATDISPLALISNRIFGEEQVDKGNAVALGAVQELYNGGGLGDAPPKRGRKPHTSEQPLPASVHKRLSTSNTLANLARALAYHKLCYEEMPLQGQDVQECCHSAMQDLSVSRRWRISRQDRHSGCDIFVTKGNRNASCKHPRIEKTRLILSQTAAQRIPQDLDAAVGVTLLILKVSGLY*

>Sacu_v1.1_s0149.g023298

MERKASFSSDSRTEKKVYPLVASDYKLLEEIGKGFSAIVFRAKCIPLGEFVAIKTVDLERCNGNLAYCSFVVEQSLWVVMPFMAGGSCLHIMKAAYPEGFEETIIASFLKETLKALEYLHQQGHIHRDVKAGNILVDGNGAVKLGDFGVSACMFDTGDRQRSRNTFVGTPCWMAPEVMEQVHGYDFKADIWSFGITALELAHGHAPFSKYPPMKVLLMTLQNAPPGLDYERDKKFSKSFKEMVAMCLVKDPSKRPSAEKLLKHSFFKHAKSADYIARTILDPLPPLWERVREVKMRDEARLAEKKMPYGEQEARSQNEYKRGVSSWNFDLEDLKAQAALNEVEPPMKDDIRKEFYAAEIHRASSPPCATSLPTTQDNNSCQQSDVPDHVSRVKSASLPPKPGNDSKFGHPPGKKEPKHFGRFDVFDDDPEQDALDSCNGNGDTKQQRKEEEKDNLTKEDDQGNGKECSFLLTTEKCMESSHAEEKTCEQDHTNQKYNHCSVVESADEGQEDGGIKSSGKQMDENATVSQPSKPPIPSSGPSVSSGAFYEGRCSNGLPTPSRLGDHKAHKESVEDKLKGQAVFQKKGRFSHGPSSRKNVSIQTSMQPVNKAPNVNDWHLERKNTGPPLSSQNPSLTTASGLNSAMVIPQLQTLFSQTALQQELIVNLMNSINPSEQSLSLKSSSFNAKNVSRSGGLSSVDSTMVEIITERERDLLSQVADLQYRVAALAEELQAVKSRNVQLERQLNAIFNKEEEERIRKEEAARDDG*

>Sacu_v1.1_s0212.g025959

MDKKKYPLSAPYYTLLEEVGQGASAAVYRAKCIPTNEIVAIKILDMERCNSNLDDIRREAQTMSLINHPNVLSAYCSFVVEHSLWVVMPYMAGGSCLHIMKSAYPDGFEEPVIATVLKECLKALEYLHSHGHIHRDVKAGNILIDSVGNIKLGDFGVSACMFDTGDRQRTRNTFVGTPCWMAPEVLEQVHGYDFKADIWSFGITALELAHGHAPFSKYPPMKVLLMTLQNAPPGLDYERDKRFSKSFKEMIAMCLVKNPAKRPTAEKLLRHSFFKHSRSPDYIARTLLDNLDPLGQRFRDLKLKDAERLAQKKMPFDEKEERSQTEYKRGVSSWNFDIEDLKAQAASIEDDDGCYATGKEELEHMDTSNIASAMQTSASQDSINDIHDAVMVQSPSAEKTGRAFPRVLSGPSVAPLRSSSEEYTQPSGVQDVGCSDESSLATDAINVQLQTETPKGTEALYGRETKGELKKADKSFQQRGDLKKEDRLFQNIDEKDLRHERMQQEGRRASSGPLVAEHVLSAYRTRDEEKDNGRVSSRGDDDVPRARVNHRERAFSGPLSVHGTPFEHRVTNGFSSTAKQVAGSKDVPDKGLYVQKKGRFSVTSADVELMEDNATSTAPRRGASMQTLPQLSQSSASLQYPVAHSPNMIPTTAVVACLQNLLQHASSQQELLVSMMNSINRGDALQGVSENISTKLYYGNPSARPLESLELEISSDREHELLQQIADLQNRLAIATDELQTQKNKNSVLERQLNALCNRKEEERIRREDEAEES*

>UM010_0073.1

MKGEHRKPSGLLKGFSRLGHSKSRKQLAPEPSKWDDMDFPTTPDEYEIQQECGRGVSATVYQGICKTNNEKVAIKKLDLDEMGWGAHWEEVVREAHTMAAHHHPNLLPLLCSFTDGNQLWMVEPYITHGSMLNIMKYAHPHGLSEELIAVIAHETLRGLDYLHHHGMIHRDVKCGNILVDADGRVYLADFGVAAPLEVRGAWGEKPRNTFVGTPCWMAPEVMQESQYDFSADIWSLGITVLELAHGHAPFAKYPPFKVVMMTVQNPPPSLDSEPNSQRRFSKELHDLVASCLQKDPAKRPTARALLEHRFFKHVPKDRDYMRKHLMAGLPDVITRVRHMKDGVKGLPFEPALQEESRSNDQYVRGLSAWNFDVAELKRQAQLEPMEPLLEEEETGHADRVASTSFTSRTAAGDSAPMAAADGAAGLPRSSADSGVSAALPVAAPPPASVVSMDTVSGALASVGVPDVAGGASSVGAPLSEAGDAISRGASHTGEFALPSTALSSSPGQLGPTDHAGLPHSPYSNPPSQPPSNPPTSNAASDMHEEDDLAQATIGHSIAQASPSTCLPHSLAMSSSTGAPAAAPADSAAASSAAALSDALGGQPLPPGSVVAVTTSVTVATTALPVPAGSPVPAGAAVPPAEQVLPPTEVNIARHTHALAATVGPPPPQQKVGRFNIKKKMDLTASDNGSAATMPEWRHGVMAGEDAVPRPGPSNSPQDNPLPMFDPVADRAIVEGAKAASVAGMPIGDKDETSSRKSSKKGRFNVTAATMPLQRSSVNLLPAQPESERSLTVEALTALDTDPAAVAASHVARVTCDSDIAGRLTSDGAWASPAHTSSTHGEADAPAAAAAAAAAANAAAASVAVSTADAVLEAALTAPLGKDLSKSRKESRFKVVKVVDGRLPSEPGGVVQGARTTSESAAPQPGAMQQGVSQGGISVNGAQQDALGIVNKKLQELMQANQAQQEALRMVTAAVSEGAKGKDLLLSDLSRNPLVAVHLLGSESQHNALLEENARLRAELEDLKRKTRRDSEKNKALTKEKELLRSHVAALREQLEAARGVPPPIAALPPDLRTSAPLLTTPTPGPGDSRTSSPSGSILAAAAGGGGGGVRLLPLDMAIIGSGGVVAGAGAPGDMTACSGSSSTTPQELSPWPGGSSQSGEVGGGARYMPPVCPSTSMAMPTPVPAAAIRSPFAGTQHMMVAGDAPAAAAHSAQHAHLRPGTLPSDLSSLAQMTKSTAAQTAPAALDAGVPADAGPAAAPAATAAAAVDVDSAPATRPLSPVAEGAAEGSASSATGQADSMPGLPMVHSADHVAVLPSCGAAAAPAVAGHDRPAEPAS*

>UM061_0059.1

MGLSDPLADAISVHGRFDAGALIGKGSYGSVYEGVDTKNGNKIALKVIDLDELEDDISEVMQEITALANCDCPQITKYYDSMLLPDSSKLVIAMELMSCSGADLLELAPWPEAGIAYAMKQVLQALDYLHSQRRMHRDIKAANVLLSAEGVVKVADFGVSGQLTATLGYKRRTFVGTPYWMAPEAIESSEEGYTCSADIWSLGITAIEMAQQRPPHSELHPMRVLFVIPKAEQPVLAGGQHSAHFKDFVALCTAKDPQQRPSASALLAHPFLKAQEKPPAELLGLLKQAEAKRAAVAPTRSAATLSRRTTAQRTESSGPFWDFPDDDRGSGMHGRPAVGEPEAPPGSEQGTVRLQRLPPSSWQDTGDGTVRIMRPDSRISGGGGGGGEGPQFRTVTAADMAAYADESDVPLITAAAAKSNARMQQAPLRPPSLRAPSTRDASEGFAPVDTATILNQASTPATLKHVIGPALTAAGRPAEGAMPAAQAVGDAVDAFARLEAASPGACLAFVAQVMSEAVAAPIAGGGELVQLRKFATRMAAAVQSAPDEPEEEKPPMLEFLKQRWRSNLARDELRAAGLAGSIGS*

>UM105_0010.1

MRTRQSSAQQHPKKRYDLREQLGRGSYGSVYKAIDTVTSEIVAVKIIPLSVTEQDGFAEVKKEIEMLQMCNHPNIVNYISSIQAEDALWIIMEYCGGGSITDVIASNGLPLEESTIAYICSEALKGLSYLHSMNKVHRDIKCGNILLTETGEVKLADFGVAAQLTNTLSKRNTFIGTPHWMAPEVIQESRYDGKVDIWALGISAIEMAETIPPRWTVHPMRVIFLISREEPPRLSEWEKWTLTFHDFVRLCLTKDTTARPTGAQLENHKFVTQSRAAPRPNLQPAIHRAKLFLSAKAAAASEAFGLSLTSTVPEEGGRQSIAATESYQGTVLQRGTVGAGGTWGTVLMHDGDPPATPSPDFQDTVVHQGSVRRPAAPRAFGRGAAVPSTPERYGTTVIHEDTTEGGEDADAYGTVVVAGEPPAGGGGYLDAVRMAGEQYGSREPSPMGRSGGGGAVGGSPQSGYWAAVASASEGGPASAAVSPSPAREKTPTQKMLSRLNSVYESGNVMPVAFLKASHAAPLALLDPDPAPAAAAPLAPPPAPGGPTAGATSGGGGSGASRGGEPGSELSRRALRMVSDLAVDSMLAAVPQQMRDNYKKQARPFDAAIVKQLQEAPVLMNLARALAFHHATLDDAPLPPAEAAAQKEVVDDLTEMLRTIMCL*

>Vocar.0005s0155.1

MERALTGLTARLTSRKSTKDLATLATGKEWPTDASAFKILDDCGRGVSATVHRAICLDNGAEVAVKLMNLESMNCDLEEIIHEAQTMRKYNHPNILPLYCSFVHEQELWMVMPFVAGGSVLHIMKYGHPEGLNETIIATIGREVLKGLDYVHKNGSIHRDVKAGNILVDGDGNVKLGDFGVAASLERGGSWGHDKQARMTFVGTPCWMAPEVMEQTSGYDFSADIWSFGITLLEMCHGHAPFAKFPPMKVLLMTLQNPAPTLEDKGQKHFSKALKDLVTRCLQKEKDKRPTAAQLLEHRFFKIAKDAKYLKDNLVGELPPLSDRVNRIRTGMAATNKQDNDRNLEKSQEEYRKGVSSWNFDLAALKAQAALEPDDETTHGANMLPTITESDEREETMTGTSAAVATPVEAERTAPQPTGAVAGPYTSTSTASVFATMNGQDATLRTNAIVPLLTSAASDGAPPQDQAGVSAGPSAERCNTQGELPPPSPGGGVSPATGLSREGSLGGNLADARAGQAATPTGGVLKQKKGRFEVSVHADVPASSAASAAAQALAQVPTMPASVPLAAAHPSVQLAHAPNSIGSNTLASMSTGSGVLISGGTVPDLQLLRGSTPGLDAASESRDEFPGTLLPAKTEVEESVTSVEPKQRGRFKIVAEQGTTSDSRPLSKASSMANLSDSNKDPSKQRSDGGNLSGKPPTAPTSMTTSVSVVLPKLQELMDHASAHQAALQKLIGAVQECEKGRVPLLLSRTQSTKSLFETSPSEAADDLRLQVVELRAKLSALEDENTRLRERNKVLETLHSAAHTVRFTLPAEAASAPGGGATSPKFEGAPTQQQLATSQPSLA*

>Vocar.0007s0076.1

MASERYERRHCIGRGSFGDVYEGVDTETGNPVAIKVIDLENVEDDIQDIHKEIQALAGCKCKNITEYYGSVAPPGTAELCIIMELMACSVSDLVQHGPLDEPCIAYILSQVLNALVYLHSERRIHRDIKAANLLLSRTAEVKITDFGVSGQLTGTLGYRRKTFVGTPFWMAPEVIETSEEGYSEKADVWSLGITTIEMATGAPPHAALHPMRVLFLIPTRPPPQLEGDFSPEMKDFVTKCLQRDPGARPAAKDLLSHPFLVGAVEPPDHLPAMVQELVRHKKPLTSRRDVEDSMAACGTMPAWDFGTIGRKAAAAAAAAAVPTGTVRAVAAAMAAVGSGATIRNGDPLREALKLQQQQQQQQQQQQQQQLATAATVAAAPRLESTSRKAVGTAFTEGLASPNGAIAAVVPVIAAAAAPPAVTAPPSPGPGTAAAAAATTAPSTPGGHTSSSSLLQLAQQAEQQQQQPPQGIAVEAVKSPLASPGARTNGVADGIGGGSGVGGALGLGGALSPSAKTRGAAHALERHSSLPTAGQQPQQQPQQQLSQPQLPQQQQQPVQQEPAPQQPQRSAPILSEKFRTMSSAERRMYGDLISKGSPWAGSGVIGGGSGSATGSGGGSGSATGSGGGGGSEGTATTTEGLVRNSLSFGAPLPAGTPPSGIAAAAVATVSAGAKNAVHRYSATSDDSSEASSGRRRPAEHSVDGTAGDADKYGTMQSRASGTASGGASSGSPSHNSGSQRSSIATHSGGAGGPSTVIGSSSGGGVYGTVQSRKQVTPLVDGNHSGAPTAAAAGGLPPRGTSTTADGLAGNAGGGGAAAAAAAASGGDEIFKGLEPLWVHGVSLVVGVIVVVVITAGYQVPTPCSVYPTCPRSSCVKRIGAFLHACRDFCVMCSVQMPGASYRLVQELLVRLSCSSDPALQPLCASAIGLYATQLATSSAAGAIGIGGTSGPGLASTAAAAAAGGGGGSSSGRGSASSSGNGISMPAAAVAAVAAAAPHSHGKNRSLPDLGPLGEFLLGRWREEEAHELALLARSMSLSVAPRR*

>Vocar.0003s0077.1

MMSKDVAAGALCATWQRDQHPTDEMESEVASSRFQLQNVLGKGAYGTVYSAVDGQTGETVAIKVIPVTDQDREELTQIQKEIRFLADCNHPNVVRYLGSYRHPNELWIVMEYCGGGSVSDLLSATSEPLSEDLIAYVCGEALKGLAYLHGLGKVHRDIKCGNILLTTGGEVKIADFGVSAQLTATMSKRNTFIGTPHWMAPEVIQESRYDGKVDVWALGISAIEMAELRPPRWNVHPLRVIFMIGRDPPPRLSQLDKWSPVFQDFVSQALLKVKVSFNRVWFVFPPAPTSALSLLSPPFAFSPPPGSDPRFAPFRATHPVVRLSPRWHTQDAAAAAQKRQSVVEVTSLEGLATGRFTWRSGTARGHGAVGAVPETEDVALAAAATMRAGARNGGGGGGGATAAVSGAGFGAIVQPEAEPASVPRSAAARGGRTPAAAAGAQPPASSPLPATPLSAVRRISSGAGFDSTMVDKEDYYGTVVVHNEYDAAAVHSDGGAGGNGLAVALAAYRTPATPAGGPAGAAEYGSAMVVGGTMIERSGTVRRSNEMADASGDGADGCGGGGGGYLAAVRAAAAEAQNGDRHAKPLDSPLGSRPAAAHHLDEVERVRERLHSLYDGGLVVPLPFFKASQAQPLALLCPSEPFAGSCSHAVQGPLLPPPLPPPLAGSSRQEGAAAAVAAAGAPYRRVQHGGGGGGGGALTRSGGGVAAVPPPPASAAAAAAAGGTSWVGALQKTSQPSLPPLPPLQQQQQQLSSSPQQQSQPHVLQSNAAPPASLDLHGVDPEAYGVVLELVQQSAAMARQRGEVVSEPGSAAVDPLPPTVVTQLLFHPALQNLARTLTYNRRCLASLPLDRRAQEELQESCNQLSAALQCVLSL*

>AL1G37450.t1

MTSSSGTKFPLDAKDYELLERIGDGVYRARCILLDEIVAIKIWNLEKCTKDLETIKKEVHRLSSIDHPNLLRSHCSFIDGTSLWIVMPYMSCGSCLNIMKSVYPNGLEEPVVAIFLREILKALVYLHGLGYIHGNVKAGNILVDSEGTVKLGVERMPTSSGNTFVGTPCWIAPEEDTQQVDGYGFKLDIWSFGMTALELAHGHSPFSKYPHTVAPPLTLQNSPCPVYEEDNKFSKSFRELVAACLIKDLEKRPTASKLLEYPFLQHTLSTEYLASTILDSLYPLGERFRKLKEEEAKLVKRIDGNSEKLSQVTIEGLLTSGKPASPVNPVSCNAAQILPMLQNLLIQNDIQRERVISLMQLYDPTAEIRIPVVNTEGGQISTPETDLLSEVHVLQQSVNKLEEEVEKQKTENAKLEGQINRLTRCTDPYRQNCFVLHLEGEAPLL*

>AL1G61540.t1

MAPEVIQNSEGYNEKADIWSLGITMIEMAKGEPPLADLHPMRVLFIIPRESPPQLDEHFSRPLKEFVSFCLKKAPAERPSAKELLKHRFIKNARKSPKLLERIRERPKYQVKEDEEIPTNGPKSPVESSGTVRVAGDERGQGTSGTSFQVKTVRNAGWDFSFGGSQGAGTVRALKPPQARERRQEVSSNQTSQKTSRASGSQLSSTFGVPEISEGGFNKRDSYQNDNQEEDDSSGSGTVVIRSPRSSQPSSMYRDQSSGSANRYTSFDDASTSGTVVVRGQNDDSGSPRTPRSRLGLQERSSSASEDSISNLAEAKLALEAGFRRGNAREKLGNGKVNKRREQATDNSDYLRNSRDHSDKQKPVIRSQQVSDDEDDSKLASLSASLSLLLLPSLKEAVGGDDSKGSVGHRVSRSLVNMEREKPGSSEAFIAKLIEQLGSTKEVSVKEVQDMAIRVFAKTMNKDAENKRKQASKEFSSNANFSPLARFLFSRWLGQTSRDLNQS*

>AL2G28310.t1

MDHNSPRSRRSRKPEPKSDIYSTFVVHSDSDSDQGRNRDKRKAKPEEDENVDLYATMVYKGDSDGEGEEDDEDDSMLPPLLKRLPKDFGGGASLDYDDDDGDETGDFGTMIVKTDRSSHSKNSPYSSKPRMGVSPRRRARGGDEESSDEEDEEEEDDDDDGEYGTFVVKSSSKKGKNKEKEIDMSTMGRAVASMQKSNFGGKTRKLDPSSSSSKLQGEDNRKMQQQNSKMSTTSLPDSITREDPTTKYEFLNELGKGSYGSVYKARDLKTSEIVAVKVISLTEGEEGYEEIRGEIEMLQQCNHPNVVRYLGSYQGEDYLWIVMEYCGGGSVADLMNVTEEALEEYQIAYICREALKGLAYLHSIYKVHRDIKGGNILLTEQGEVKLGDFGVAAQLTRTMSKRNTFIGTPHWMAPEVIQENRYDGKVDVWALGVSAIEMAEGLPPRSAVHPMRVLFMISIEPAPMLEDKEKWSLVFHDFVAKCLTKEPRLRPTAAEMLKHKFVERCKTGASAMSPKIEKSRQIRAVMALQAQSVVAPSSEDTSTLGPKSNEEVGITVPAKPPQNSTEAPLTGTLNRQHITGNTVLAGEGGDFGTMIVHGEDEAEESDSRSQLVKEKEESSSSQVEGVSREVSGEELPDSWIHDKKNPPGIDLPIEASTSQSIQASSSHEHRTKLNNIAGTQIEGGSDASGSTLKNETVGRKAFALQDKLWSIYAAGNTVPIPFLRATDISPIALLSENMIGGMQQDGNGTVAVEALQELFTSDPQSKKGRRGQNEMPLPPSVYQRLTTSSSLMNLAQVLAYHRACYEEMPLQELQATQEQQTIQNLCDTLRTILRL*

>AL2G29800.t1

MAGSSTKRFPLYAKDYELFEEVGEGVSATVYKARCIALNEIVAVKILDLEKCRNDLETIRKEVHIMSLIDHPNLLKAHCSFIDRSSLWIVMPYMSGGSCFHLMKSVYPEGLEQPIIATLLREVLRALVYLHRQGHIHRDVKAGNILIHSKGVVKLGDFGVSACMFDSGERMRTRNTFVGTPCWMAPEVMQQVDGYDFKADIWSFGITALELAHGHAPFSKYPPMKVLLMTLQNAPPRLDYDRDKKFSKSFRELIAACLVKDPKKRPTAAKLLKHPFFKHARSTDYLSRKILHGLSPLGERFKKLKEAEAELFKGINGDKEQLSQHEYMRGISAWNFDLEDLRRQAAIVPNDEMCNSEIQELNTNGDVPKGKPVMQRSQTMPLEFFSEKDMMSESYSQLTGSLLPSFHRKFLPTIGYQVGILSDERNACCSSDGVAEKLALGKPHQLEPLADTEQIGKAGSEQEKPKNGYAVSPVNRESSTSKEPLADTKQIRKAGNEQEKPKNGYIVSPVNGESSPSKEILPLLQSLLVQNDIQRAKVIRLIRFFDRTAGTENPISKTEGVQVYPSKEKDLQSQVQFLEQSVEKLVEEVQRRKEINSQLEQQISSLISSSSSSNIP*

>AL2G40330.t1

MEKKKYPIGPEHYSLYEVIGQGVSALVHRALCIPFDEVVAIKILDFERDNCDLNNISREAQTMMLVDHPNVLKSHCSFVSDHNLWVIMPYMSGGSCLHILKAAYPDGFEEAIIATILREALKGLDYLHQHGHIHRDVKAGNILLGARGAVKLGDFGVSACLFDSGDRQRTRNTFVGTPCWMAPEVMEQLHGYDFKADIWSFGITGLELAHGHAPFSKYPPMKVLLMTLQNAPPGLDYERDKKFSRSFKQMIASCLVKDPSKRPSAKKLLKHSFFKQARSSDYIARKLLDGLPDLVNRVQAIKKKEEDMLAQEKMADGEKEELSQNEYKRGISGWNFNLDDMKAQASLIQDMDCGFSDSLSGSTTSLQALDSQDTQSETQEDVGQITNKYLQPLIHRSLSIARDKSDDDSSLASPSYDSYVYSSPRHEDLSLNNTTVGSNHAINGKPTDSTSIPTNQPTEILAGNSALADRNGIPNKGESDKTQDHLQNGSNCNGAHPTVGGDEVPTELAVKPPKAASSLDESDDKSKPPVVQQRGRFKVTSENLDIEKVVAPSPILQKSHSMQVLCQHSSAPLPHSDVTLPNLTSSYVYPLVYPVLQTNILERDNILHMMKVLTNRELTDGRAVEQGSVQQPTVPPTEKSMLEAAHEREKELLHDITDLQWRLICAEEELQKYKTEHA*

>AL3G27620.t1

MDDVAGLREAAGARFSHIELIGRGSFGDVYKAFDKDLNKEVAIKVIDLEESEDEIEDIQKEISVLSQCRCPYITEYYGSYLHQTKLWIIMEYMAGGSVADLLQSSNPLDETSIACITRDLLLAVEYLHNEGKIHRDIKAANILLTENGDVKVADFGVSAQLTRTISRRKTFVGTPFWMAPEVIQNSEGYNEKADIWSLGITVIEMAKGEPPLADLHPMRVLFIIPRENPPQLDEHFSRPVKEFVSLCLKKVPAERPSAKELIKHRFIKNARKSPKLLERIRERPKYQVKEDEETPRNGPKAPVDSSGTVRVAKDERSQGTPGSSFQGNTVKNAGWDFSVEGSQSIGAVRALKPPQARERRHEVSSNQISQQTTRDSGNQWSSSTGSTISEASEGGFVRRHPFQNDHEDGFHEEDDSSLSGSGTVVIRASRSSQSSSKFREQSSVSSGRYAPFDDASASGTVIVRGQYDDSGSPRTPKSRLGIQERTSSASEDSNANLAEAKAALDAGFRKGNARERLGMGKNNNDGKVNRRRDQMADDSDYSRNSGDKSSKQKAVPRSEQVSDEEDDSIWESLPASLSILLIPSLKEALGDDSKESIGRTVSRSLVMMEREKPGSCEAFVAKLIELLGSSKEASVKELHDMAVRVFAKTTPDAAENKRKQANKEFSSNTNVSPLGRFLLSRWLGQSSRDL*

>AL6G25490.t1

MSLINHPNVLQAHCSFTAGHQLWVVMPYMAGGSCLHIIKSSYPDGFEEPVIATLLRETLKALVYLHAHGHIHRDVKAGNILLDSNGAVKLADFGVSACMFDTGDRQRSRNTFVGTPCWMAPEVMQQLHGYDFKADVWSFGITALELAHGHAPFSKYPPMKVLLMTLQNAPPGLDYERDKRFSKAFKEMVGTCLVKDPKKRPTSEKLLKHPFFKHARPADYLVKTILNGLPPLGDRYRQIKSKEADLLMQNKSEYEAHLSQQEYIRGISAWNFNLEDLKTQAALISDDDTSHAEEPDFNQKQCERQDESALSPERASSSATAPSQDDELNDIHDLESSFASFPIKPLQALKGCFDISEDEDNATTPDWKDANLNSGQQHLTKASIGSLADTTKEEDTAAQNTSLPRHVISEQKKYLSGSIIPESTFSPKRITSEADREFQQRRYQTERSYSGSLYRTKRDSVDETSEVPHVEHKGRFKVTSADLSPKGSTNSTFTPFSGGSSSPSSLNATTASILPSIQSILQQNAMQREEILRLIKYVEQTSAKQPGSPETNVDELLQTPPATPRERELQSQVMLLQQSFSSLTEELKKQKQKNGQLENQLNALTHRND*

>AL6G44780.t1

MVSRLRLALEAVFGSRRRKKKMASTSSGGDKKKKKGFSVNPKDYKLMEEVGYGASAVVHRAIYLPTNEVVAIKCLDLDRCNSNLDDIRREAQTMTLIDHPNVIKSFCSFAVDHHLWVVMPFMAQGSCLHLMKAAYPDGFEEAAICSMLKETLKALDYLHRQGHIHRDVKAGNILLDDTGEIKLGDFGVSACLFDNGDRQRARNTFVGTPCWMAPEVLQPGSGYDSKADIWSFGITALELAHGHAPFSKYPPMKVLLMTIQNAPPGLDYDRDKKFSKSFKELVALCLVKDQTKRPTAEKLLKHSFFKNAKPPEICVKKLFVDLPPLWTRVKALQAKDAAQLALKGMASADQDAISQSEYQRGVSAWNFNIEDLKEQASLLDDDDILTESREEEESPGEQLHNKVNDRGQVSCSQLLSENMNGKEKVSDTEVVEPICEEKSTLNSTASSVEQAAPSSEQDVPQAKGKSVRRQTHSGPLSSGVVLINSDSEKGPGYERSESERQLKSSVRRAPSFSGPLNLPNRASANSFSAPIKSSGGFRDSIDDKSKTNVVQIKGRFSVTSENLDLARSSPLRKSASVGNWILDSKMPTGQPSKESSSHHASPSFILPQLQNLFQHNSIVQDLTLNLVNSLQQAAEATDGSQNGKLPPLPRGSDSNGTVVELTASERERLLLTKITELRARMKELTEELEKEKSKETQLQQKLKSVTGREQL*

>AL7G29190.t1

MVGGGSSSGGGGVFRGGGSGKQQRGFSLNPKDYKLLEEIGHGASAVVYRAIYLPTNEVVAIKCLDLDRCNSNLDDIRRESQTMSLIDHPNVIKSFCSFSVDHSLWVVMPFMAQGSCLHLMKTAYSDGFEESAICCVLKETLKALDYLHKQGHIHRDVKAGNILLDDSGEIKLGDFGVSACLFDNGDRRRARNTFVGTPCWMAPEVLQPGEGYNSKADIWSFGITALELAHGHAPFSKYPPMKVLLMTIQNAPPGLDYDRDKKFSKSFKEMVAMCLVKDQTKRPTAEKLLKHSCFKHTKPPEFYVKKLFSDLPPLWTRVKSLQDKDAQQLALKRMATADEEAISQSEYQRGVSAWNFDVRDLKTQASLLIDDDDLEESKEDDEILCAQFNKVNDRVQVFDSLQLYETMNEKEKVSNTEVEEPTCEEKFTFITTASSLERMSPNSEHDIPEAKVKPVRRQSQSGPLTSKTVLCHSASEKGHIFERSESEQQTASTVRRAPSFSGPLNLPTRASSNSLSAPIKYSGGFRDSLDDKSKANLVQKGRFSVTSGNVDLAKDVPLSIVPRRSPQATPLRKSASVGNWILEPKMPTAQPQTIKEHSSHPTSSSSSLIVPQLQHLFQQNSIQQDLIMNLLNSIQPGEATEGSQSGKLPPLPRSDSNGNVEPVASERERLLLSSISDLRARLDDLTEELDIEKSKYSQLQQKLKAFTGRKHV*

>AL7G41660.t1

MARNKIEFPLDAEAYEIICKIGVGVSASVYKAICIPMNSMVVAIKAIDLDQSRADFDSLRRETKTMSLLSHPNILNAYCSFTVDRCLWVVMPFMSCGSLHSIVSSSFPNGLPENCISVFLKETLSAISYLHDQGHLHRDIKAGNILVDSDGSVKLADFGVSASIYEPVTSSSGTTSSSLRLTDIAGTPYWMAPEVVHSHTGYGFKADIWSFGITALELAHGRPPLSHLPPLKSLLMKITKRFHFADYEINTSGSSKKGNKKFSKAFREMVGLCLEQDPAKRPSAEKLLKHPFFKNCKGLDFVVKNVLHSLSNAEQMFMESQILMKSVGDDEEDEEEDEKIAKNRRISGWNFREDDLQLSPVFPATDSDTSESSPREEDHIQDKQEDDNVTITGSELGLGLSNEEAKNQEGEVVGFDKDLVLEKLKVLKKSLEHQRARVSIIIEALSGDKEEKSREEELLDMVEKLKIELEAEKLRS*

>AT1G53165.1

MDDVAGLQEAAGTRFSQFELIGRGSFGDVYKAFDTELNKDVAIKVIDLEESEDEIEDIQKEISVLSQCRCPYITEYYGSYLHQTKLWIIMEYMAGGSVADLLQPGNPLDEISIACITRDLLHAVEYLHAEGKIHRDIKAANILLSENGDVKVADFGVSAQLTRTISRRKTFVGTPFWMAPEVIQNSEGYNEKADIWSLGITMIEMAKGEPPLADLHPMRVLFIIPRESPPQLDEHFSRPLKEFVSFCLKKAPAERPNAKELLKHRFIKNARKSPKLLERIRERPKYQVKEDEEIPTNGPKAPAESSGTVRVAKDERGQGTSGTSFQVKTVRNAGWDFSIGGSQGAGTVRALKPPQARERRQEVNSNQTSQKTSRTSGSQLSSTFGVPEISEGGFNKRDSYQNDYQEEDDSSGSGTVVIRSPRSSQSSSMFRDQSSGSTRYTSFDDASTSGTVVVRGQNDDSGSPRTPRSRLGLQERSSSASEDSISNLAEAKLALEAGFRRGNARERLGNGKVNKRREQATDNSDYLRNSRDHSDKQRPVMRSQQVSDDEEDDSKLASLSASLSLLLLPSLKEAVGGDDSKGTIGHRVSRSLVKMEREKPGSSEAFIAKLIEQLGSTKEVSVKEVQDMAIRVFAKTMNNDAENKRKQASKEFSSNANFSPLARFLFSRWLGQTSRDLNQS*

>AT1G69220.1

MDHNSPKSRRSRKPEPKPDIYSTFVVHSDSDSDQGRDRDKRKAKPEEDENVDLYATMVYKGDSDGEGEEDDDDDSMLPPLLKRLPKDFGGGASLDYDDDDGDESGDFGTMIVKTDRSSHSKKNSPYSSKPRMGVSPRRRARGGDEESSDEEDEEEDDDDDDGDYGTFVVKSKDKKGKKKDKEIDMTTMGRAVASMQKSNFGGKTRKLDPSSSSSKLHGEDNRKMQQQNSKMSTTSLPDSITREDPTTKYEFLNELGKGSYGSVYKARDLKTSEIVAVKVISLTEGEEGYEEIRGEIEMLQQCNHPNVVRYLGSYQGEDYLWIVMEYCGGGSVADLMNVTEEALEEYQIAYICREALKGLAYLHSIYKVHRDIKGGNILLTEQGEVKLGDFGVAAQLTRTMSKRNTFIGTPHWMAPEVIQENRYDGKVDVWALGVSAIEMAEGLPPRSSVHPMRVLFMISIEPAPMLEDKEKWSLVFHDFVAKCLTKEPRLRPTAAEMLKHKFVERCKTGASAMSPKIEKSRQIRATMALQAQSVVAPSLEDTSTLGPKSSEELGITVPSKPPQNSTEAPLTSTLNRQHITGNTVLAGEGGDFGTMIVHGEDETEESDSRSQLVREKESSSSQFEGVPREFPGEELPDSWIHDKKKPPAIDLPVEASISQSMQASSSHEHRTKLHNIAGTQMEGGSDASGSTLKNETVGRKAFALQDKLWSIYAAGNTVPIPFLRATDISPIALLSENMIGGMQQDGNGTVAVEALQELFTSSDPQSKKGRRGQNEMPLPPSVYQRLTTSSSLMNLAQVLAYHRACYEEMPLQELQATQEQQTIQNLCDTLRTILRL*

>AT1G70430.1

MAGSSTKRFPLYAKDYELFEEVGEGVSATVYRARCIALNEIVAVKILDLEKCRNDLETIRKEVHIMSLIDHPNLLKAHCSFIDSSSLWIVMPYMSGGSCFHLMKSVYPEGLEQPIIATLLREVLKALVYLHRQGHIHRDVKAGNILIHSKGVVKLGDFGVSACMFDSGERMQTRNTFVGTPCWMAPEVMQQLDGYDFKYLAHGHAPFSKYPPMKVLLMTLQNAPPRLDYDRDKKFSKSFRELIAACLVKDPKKRPTAAKLLKHPFFKHARSTDYLSRKILHGLSPLGERFKKLKEAEAELFKGINGDKEQLSQHEYMRGISAWNFDLEALRRQASLVIIPNEEIYNSEIQELNRNGDVPKGKPVIQRSQTMPLEYFSEKASDMVSESSSQLTGSLLPSFHRKFLPALGNACNSSDRAAEKLAFEEPRQVLHPLADTKKIRKAGSDQQEKPKNGYADSPVNRESSTLSKEPLADTKQVRKPGNEQEKPKNGYIVSHVNRESSTSEEILPLLQSLLVQNDIQRAQVIRLIRFFDRTAKTENPISKTEGVQEKDLQSQVQFLEQSVEKLVEEVQRRKDINSQLEQQISSLISSNNIS*

>AT3G15220.1

MDDVAGLQEAAGARFSQIELIGRGSFGDVYKAFDKDLNKEVAIKVIDLEESEDEIEDIQKEISVLSQCRCPYITEYYGSYLHQTKLWIIMEYMAGGSVADLLQSNNPLDETSIACITRDLLHAVEYLHNEGKIHRDIKAANILLSENGDVKVADFGVSAQLTRTISRRKTFVGTPFWMAPEVIQNSEGYNEKADIWSLGITVIEMAKGEPPLADLHPMRVLFIIPRETPPQLDEHFSRQVKEFVSLCLKKAPAERPSAKELIKHRFIKNARKSPKLLERIRERPKYQVKEDEETPRNGAKAPVESSGTVRIARDERSQGAPGYSFQGNTVKNAGWDFTVGGSQSIGTVRALKPPQARERRQEVSPNRISQRTTRPSGNQWSSATGSTISEASEGGFVRRHPFQNDHEDGFHEEDDSSLSGSGTVVIRTPRSSQSSSVFREPSSGSSGRYAAFDDASASGTVVVRGQYDDSGSPRTPKSRLGIQERTSSASEDSNANLAEAKAALDAGFRRGKARERLGMGNNNNDGKVNRRREQMADDSDYSRNSGDKSSKQKVVPRSEQVSDEEDDSIWESLPASLSVLLIPSLKEALGDDSKESTVRTVSRSLVMMEREKPGSCEAFVAKLIELLGSSKEASVKELHDMAVCVFAKTTPDNAENKMKQANKEFSSNTNVSPLGRFLLSRWLGQSSRDL*

>AT4G10730.1

MVSRFRLALEAVLGSRRRKKMASTSSGGGGGGDKKKKKGFSVNPKDYKLMEEVGYGASAVVHRAIYLPTNEVVAIKSLDLDRCNSNLDDIRREAQTMTLIDHPNVIKSFCSFAVDHHLWVVMPFMAQGSCLHLMKAAYPDGFEEAAICSMLKETLKALDYLHRQGHIHRDVKAGNILLDDTGEIKLGDFGVSACLFDNGDRQRARNTFVGTPCWMAPEVLQPGSGYNSKADIWSFGITALELAHGHAPFSKYPPMKVLLMTIQNAPPGLDYDRDKKFSKSFKELVALCLVKDQTKRPTAEKLLKHSFFKNVKPPEICVKKLFVDLPPLWTRVKALQAKDAAQLALKGMASADQDAISQSEYQRGVSAWNFNIEDLKEQASLLDDDDILTESREEEESFGEQLHNKVNDRGQVSGSQLLSENMNGKEKASDTEVVEPICEEKSTLNSTTSSVEQPASSSEQDVPQAKGKPVRLQTHSGPLSSGVVLINSDSEKVHGYERSESERQLKSSVRRAPSFSGPLNLPNRASANSLSAPIKSSGGFRDSIDDKSKANVVQIKGRFSVTSENLDLARASPLRKSASVGNWILDSKMPTGQAIKESSSHLSFIIPQLQNLFQQNSMQQDLIMNLVNTLQQAAETTDGSQNGKLPPLPRGSDSNGTVVELTAAERERLLLTKITELRARMKELTEELEVEKSKQTQLQQKLKSVTGREQL*

>AT4G14480.1

MARNKLEFPLDAEAYEIICKIGVGVSASVYKAICIPMNSMVVAIKAIDLDQSRADFDSLRRETKTMSLLSHPNILNAYCSFTVDRCLWVVMPFMSCGSLHSIVSSSFPSGLPENCISVFLKETLNAISYLHDQGHLHRDIKAGNILVDSDGSVKLADFGVSASIYEPVTSSSGTTSSSLRLTDIAGTPYWMAPEVVHSHTGYGFKADIWSFGITALELAHGRPPLSHLPPLKSLLMKITKRFHFSDYEINTSGSSKKGNKKFSKAFREMVGLCLEQDPTKRPSAEKLLKHPFFKNCKGLDFVVKNVLHSLSNAEQMFMESQILIKSVGDDDEEEEEEDEEIVKNRRISGWNFREDDLQLSPVFPATESDSSESSPREEDQSKDKKEDDNVTITGYELGLGLSNEEAKNQEGEVVGFDKDLVLEKLKVLKKSLEHQRARVSIIIEALSGDKEEKSREEELLEMVEKLKIELETEKLKTLRADKDSVLG*

>AT5G14720.1

MESGSEKKFPLNAKDYKLYEEIGDGVSATVHRALCIPLNVVVAIKVLDLEKCNNDLDGIRREVQTMSLINHPNVLQAHCSFTTGHQLWVVMPYMAGGSCLHIIKSSYPDGFEEPVIATLLRETLKALVYLHAHGHIHRDVKAGNILLDSNGAVKLADFGVSACMFDTGDRQRSRNTFVGTPCWMAPEVMQQLHGYDFKADVWSFGITALELAHGHAPFSKYPPMKVLLMTLQNAPPGLDYERDKRFSKAFKEMVGTCLVKDPKKRPTSEKLLKHPFFKHARPADYLVKTILNGLPPLGDRYRQIKSKEADLLMQNKSEYEAHLSQQEYIRGISAWNFNLEDLKTQAALISDDDTSHAEEPDFNQKQCERQDESALSPERASSSATAPSQDDELNDIHDLESSFASFPIKPLQALKGCFDISEDEDNATTPDWKDANVNSGQQLLTKASIGSLAETTKEEDTAAQNTSLPRHVISEQKKYLSGSIIPESTFSPKRITSDADREFQQRRYQTERSYSGSLYRTKRDSVDETSEVPHVEHKGRFKVTSADLSPKGSTNSTFTPFSGGTSSPSCLNATTASILPSIQSILQQNAMQREEILRLIKYLEQTSAKQPGSPETNVDDLLQTPPATSRERELQSQVMLLQQSFSSLTEELKKQKQKNGQLENQLNALTHRND*

>AT1G23700.1

MTSSPETRFPLVAKDYEILEEIGDGVYRARCILLDEIVAIKIWNLEKCTNDLETIRKEVHRLSLIDHPNLLRVHCSFIDSSSLWIVMPFMSCGSSLNIMKSVYPNGLEEPVIAILLREILKALVYLHGLGHIHRNVKAGNVLVDSEGTVKLGDFEVSASMFDSVERMRTSSENTFVGNPRRMAPEKDMQQVDGYDFKVDIWSFGMTALELAHGHSPTTVLPLNLQNSPFPNYEEDTKFSKSFRELVAACLIEDPEKRPTASQLLEYPFLQQTLSTEYLASTFLDGLSPLGERYRKLKEEKAKLVKGVDGNKEKVSQENVEALLMEPASLVNPVSCDTAQVLPILQNILIQNDIQRENVEALLTEPAILVNPVSCDTAQVLPIVQNILIQNDIQRKRLIGLMQLCDPTAGKFAVLSLEFASSLCYKFHDLILIFVQKSEFRLAIQKLGRYQQQRQIYCLRFTFCSRGNDKFHKL*

>AT1G79640.1

MEKKKYPIGPEHYTLYEFIGQGVSALVHRALCIPFDEVVAIKILDFERDNCDLNNISREAQTMMLVDHPNVLKSHCSFVSDHNLWVIMPYMSGGSCLHILKAAYPDGFEEAIIATILREALKGLDYLHQHGHIHRDVKAGNILLGARGAVKLGDFGVSACLFDSGDRQRTRNTFVGTPCWMAPEVMEQLHGYDFKADIWSFGITGLELAHGHAPFSKYPPMKVLLMTLQNAPPGLDYERDKKFSRSFKQMIASCLVKDPSKRPSAKKLLKHSFFKQARSSDYIARKLLDGLPDLVNRVQAIKRKEEDMLAQEKMADGEKEELSQNEYKRGISGWNFNLDDMKAQASLIQDMDCGFSDSLSGSATSLQALDSQDTQSEIQEDTGQITNKYLQPLIHRSLSIARDKSDDDSSLASPSYDSYVYSSPRHEDLSLNNTHVGSTHANNGKPTDATSIPTNQPTEIIAGSSVLADGNGAPNKGESDKTQEQLQNGSNCNGTHPTVGGDDVPTEMAVKPPKAASSLDESDDKSKPPVVQQRGRFKVTSENLDIEKVVAPSPILQKSHSMQVLCQHSSASLPHSDVTLPNLTSSYVYPLVYPVLQTNILERDNILHMMKVLTNRELTDGRAVEQGSIQQPTVPPTEKSMLEAAHEREKELLHDITDLQWRLICAEEELQKYKTEHAQVSMSN*

>AT4G24100.1

MVGGGGGSSGRGGGSGSGSSKQQRGFSMNPKDYKLMEEIGHGASAVVYRAIYLPTNEVVAIKCLDLDRCNSNLDDIRRESQTMSLIDHPNVIKSFCSFSVDHSLWVVMPFMAQGSCLHLMKTAYSDGFEESAICCVLKETLKALDYLHRQGHIHRDVKAGNILLDDNGEIKLGDFGVSACLFDNGDRQRARNTFVGTPCWMAPEVLQPGNGYNSKADIWSFGITALELAHGHAPFSKYPPMKVLLMTIQNAPPGLDYDRDKKFSKSFKEMVAMCLVKDQTKRPTAEKLLKHSCFKHTKPPEQTVKILFSDLPPLWTRVKSLQDKDAQQLALKRMATADEEAISQSEYQRGVSAWNFDVRDLKTQASLLIDDDDLEESKEDEEILCAQFNKVNDREQVFDSLQLYENMNGKEKVSNTEVEEPTCKEKFTFVTTTSSLERMSPNSEHDIPEAKVKPLRRQSQSGPLTSRTVLSHSASEKSHIFERSESEPQTAPTVRRAPSFSGPLNLSTRASSNSLSAPIKYSGGFRDSLDDKSKANLVQKGRFSVTSGNVDLAKDVPLSIVPRRSPQATPLRKSASVGNWILEPKMPTAQPQTIKEHSSHPTSSSPIMPQLQHLFQQNSIQQDLIMNLLNSLQPVEATEGSQSGKLPPLPRSDSNGNVEPVASERERLLLSSISDLRARLDDLTEELDIEKSKYSQLQQKLKAFTGREH*

>ATR0150G013.1

MSDSSINDIELRFSSLEQIGRGSFGDVYKGFDKVLNKDVAIKVIDLEEAEDEIEDIHKEISVLSQCRCPYITEYYGSYLHQTKLWILMEFMAGGSVADLIQTGPPLDEMSIACILRDLLHAIEYLHSEGKIHRDIKAANILLTANGDVKVADFGVSAQLTRTISRRKTFVGTPFWMAPEVIQNTDGYNEKADIWSLGITAIEMAKGEPPLADIHPMRVLFIIPRENPPQLDDHFSRPMKEFVSLCLKKNPSERPSAKELLKHRFIKNVRRSPRLLERIRERPKVLVRNPDTQRNGHTAFEDVSNPVRETKGLKDEETVRARNAGWDFSVRGNLGGGTISNAVSPPQVNATKEKFSDAQYTQVLPRVSSELDSRYSTLGNTPQDSTLNFTTEIKGRDQQTDEREEISLGDGKVSANSSGTVISRTPRVTQKSSFFTDQSTASSSSRRSSSGDLSPSGTIVVRGPLDEADPPSRTPRSRLGIQEKASRASQEDSAVNLAEAKAALQAGLRKGNTRERFQLGKLHIDGQESKVAERPTSSDSSRDSHEYLDAPNLLSRLGRSSDDESGARYAAAVSSPSLSLLLIPALKEASGNDSEGAVVRAVTNSLIEMEHALPSSCEVFVSRLLQQLGSTKEPSVKGLQELAARIFAKTSAPPNMESANNEATSSKPSNGKRQPNVDQTENSGLSPLARFLISRWQGQVSRDLNSV*

>ATR0580G015.1

MERKKYPIKAEDYRLHEEIGHGVSAKVFRAICIPLNEIIAIKVLDFEKTNSNLDNIRREAQTMILVDHPNVLKAHCSFVHEHTLWVVMPYMAGGSCLHILKHAYPDGFEETIIATVLREALKGIAYLHHHGHIHRDIKAGNILVEATGAIKLGDFGVSVCLYDSGDRQRSRNTFVGTPCWMAPEVMEQLHGYNFKADIWSFGITALELAHGHAPFSKYPPMKVLLMTLQNAPPGLDYERDKKFSKSFKQMIAMCLAKDPSKRPSAEKLLKHAFFKQARSNDYMARTILDGMPSLGDRIRHLKDKEADMLAQKKMPDGEKEERSQNEYNRGISAWNFNIEDVKAQASLIPDVDDILPDKDQGMLGFYDSLEKDSNCQPAVMDISDMVATDGGHATGPGDAHLPLPGQDPKIIRFKAHGFEDDFDALSLTGEGSARQSTSVMELQCNNADIEKFANEQEVVSNPFLPLATYKGERLDSVIYDK*

>ATR0620G134.1

CRDYGEDCGKFLVIMGRMGGQKSYSALPGDYKLLEEVGHGASAVVYRAIYLPFNEVVAVKCLDLDRCGSNLDDIRREAQTMSLIDHPNVIRAYSSFVVDHSLWVVMPFMAEGSCLHLMKIAYPEGFEEAVIGSVLKETLKAVEYLHRHGHIHRDVKAGNILLDSDGGVKLADFGVTACMFDRGDRQRSRNTFVGTPCWMAPEVLQPGSGYNFKADIWSFGITALELAHGHAPFSKYPPMKVLLMTIQSAPPGLDYDRDKRFSKSFKEMVAMCLVKDPMKRPTAEKLLKHSFFKHAKPPEATVPGLLVNLPPLWERVKALQLKDAAQLALKKMPSAEEEALSQDEYKRGVSAWNFDVEDLKSQAALVQDDDDPSVLKEEDEDLGPLVNDRVFSVSKSGAGKSISTNDLYCKGNIDGAGCLDSRCFGAREENSESESPAAENLEQRDGRENGSINDPIHSLSKRDPEQSNSRNQLFKHRQTHSGPLMPIRVLSMPSSERGRFSERDEGDYPSAMERGCKHDGRKEPNLSGPLMLPSRASANSLSAPIRSSGFREGLDDKTKANVIQIKGRFSITSENADRVKDIPLCSVSRRSSQGSPLRKSASVGDWLVDTKQTRPKEASNLPIPVSVLIPQLQNLFQQTIFQQDLVANLLRTVQQTELVEGGKWAPPTQSLGSDSSNDPAVSERERLLSVKVAELQNRMINLTDELTAAKLKHVQLQQQLTEAHGREEDRIRKEEKEYS*

>ATR0686G134.1

MATDPRLRVPYPLEASAYRIVHEIGQGLSSVVYLAECLPLGSTVAIKALDLDRAPTHRLDDIRCEAQTMTLLSHPNVLTAHCSFTVDHSLWVVMPFCSAGSLHSILSASFPSGFDETVIAIVLRDTVRALNYLHQQGHVHRDIKAGNILLDSMGLIKVADFGVSASLFDPSSLTKLQEMAGTPYWMAPEVIDSAARGGPGYDYKADVWSLGITALELAHGKLPRSDVPLGKAIVLSATKPFPASSRKKFSKAFREMVGLCLTHDPVKRPSASKLLKHSFFKHCKSSEYLVKHVLNDLPSVEVRFKAMAEAGRIPKQMVDDEEDEAHELQKTRRISGWNFDEDCFRFVPVFPFQEEQGAKNQENDERGAQKEQDQEINATGAQKEQDQAKNHEIDETGAQKEQDQANNQEIEAEEESEEAKNQEIRAGGVNKERESGDQEEREGETNQEGGEAKTVVSGGRMAAMIPELERILEHSEAQRQGLVRLVDMFVDGRREVELLDLLGSLQLEHDRLTSELEILTKRNEELQRQWEEATGVGRIEQPDMEGANPELQEP*

>ATR0712G038.1

MASPRPFSRKSKSSNQADMYSTVVIHGDTGDSDQRDPDPRKDEEEEEDIYATMITKDHEDPSLPPLLKRLPKDFGAAIDYEEEEHNYSGSVIVRSNRSSVSKYPRKPPGIDPRSRGSLRKKPEVEDPYSTFLVKSSVRSRGGTFRGSPFGTMLSRTGSNFSTSMSKAVESIRGGAGMEFTPQMRDFHVEEGRKAMRRSVSSLPDSVTKEDPTNKYELLNELGKGSYGAVYKARDIKTSEMVAIKVISLCEGEEGYEEICGEIEMLQQCNHPNVVRYLGSYQGEDYLWIVMEYCGGGSVADLMGTTEETFEEHHIAYICREALKGLSYLHSIFKVHRDIKGGNILLTEQGEVKLGDFGVAAQLTRTMSKRNTFIGTPHWMAPEVIQESRYDGKVDVWALGVSAIEMAEGLPPRSTVHPMRVIFMISSEPAPMLEDKEKWSLVFHDFVAKCLTKEPRLRPNATEMLKHKFIEKCKWNASVMLPKIEKARIIRAAMSAQALHQVPGTSFPGGSDINESHADTVPTEPSKIPTHEVRDETSKVHDEEPPREGESGTFIVHMQLEREAKEIAPAGTKDMTTSMDVRIPSSHALEDRQPKFEVDMERTSPGGVPSVASSHLPQDINDSFPSLDTFPEKSMQKEKAAPLQRDSGDPQMGGESTSGSTLKTATIGRQGFALQDKLWSIYAAGNTVPIPFLKATDISPLALISDNDNGDRDPDGNGTTALEAIQELYSGDGTARKGRRAQNNEVPLPSSVHKRLTTSSTLFNLAQALAYHKMCYDEMPLQGLQAAQEQRTIQNLGDTLRTILRL*

>ATR0730G015.1

MDHAYEKKYPLSSKDYRLYEEVGEGVSASVYRALCIPLNEIVAIKVLDLEKCNNDLDGIRREVQMMSLIDHPNLLRAHCSFTADHNLWVVMPYMAGGSCLHIMKTTYPEGFDEPVIATLLREVLKALVYLHGHGHIHRDVKAGNILVDGNGAVKLADFGVSACMFDTGDRQRSRNTFVGTPCWMAPEVMQQLHGYDFKADIWSFGITALELAHGHAPFSKYPPMKVLLMTLQNAPPGLDYERDRRFSRNFKEMVAACLVKDPKKRPSSEKLLKHHFFKHARSNEYIARTILDGLSPLGDRFRALKAKEADLLLQNKAMYGDKEQKSQHEYIRGISAWNFNLDDLKAQASLIQDYDENPGSNDINMNGKEKAGFNDAPDPLSPNNANHDTSEIVEEDGDHILKDRERPFPSSFPLHPLEALKGYFDVTEDDINSSSPKEGIQSEFEPQEDDQEPKPNENEGFERSFSSSKVIINPLPGRGNKFLSGSLIPERVTRSGNGDGDR*

>Bo1g037530.1

MQTLSPQSLRWKSNPLIVSESYVFNPKALNFQLSSLSSPHRRTRNWRISSSPEENAANSSDGGDLKKSLSGIVGNQVEELLSREENKSLLDGLEKASMRVETAKRELAEIERQELEAKLLQDYVDKLESRAAEIAECQQEIVAARTMVEEAERSLSLAETAATKSSENGYSIDKDKERLESAKAAAIAAAVGTVAEVPFALSQVSTIEQLVLPLGVAFASCALFGVTFRYVIRRDLDDSHLKSGAVAAFGFVKGLGMLSRGPPLELSWESLFSHGIDGAVLVSQSVLIFAFASISLDFCFKLKLLKPFPSSAQMAVFGGKRRKKMVRGSSSSGAVVRGGGGSSVKQKGFSMNPKDYKLMEEVGHGASAVVYRAIYLPTNEVIAVKCLDLDRCNSNLDDVRRESQTMSLIDHPNVIKSFCSFSVDHSLWVVMPFMAQGSCLHLMKTAYSDGFEESAICSILKETLKALDYLHKQGHIHRDVKAGNILLDDNGEIKLGDFGVSACLFDNGDRQRARNTFVGTPCWMAPEVLQPGNGYNSKADIWSFGITALELAHGHAPFSKYPPMKVLLMTIQNAPPGLDYDRDKKFSKRTFSCEQSFKEMVAMCLVKDQTKRPTAEKLLKHSCFKHTKPPELAVKSLFADLPPLWTRVKSLQAKDAAQLALKRMATADEEAISMSEYQRGVSAWNFDVKDLKTQASLVNDSPVLYENMNGKEKVSTTEVEEPNCEEKFTFITNASSVTPNSEHEVPEAKVNKPVRRQSQSGPLTSRAVVSHSASEKEKAFKSNPTFETLLRSESEHQAAPSVKRAPSFSGPLNLSTRASSNSLSAPIKYSGGFRDSLDEKSKGNLVQKGRFSVTSGNLDLAKDVPLSIVPRRSPQPTVQPQTIKELSSQTMSPSLIIPQLQHIFQQNSVQQDLLMNLLNSVQPAETTDGSQSGKLPPLPRSETNGTVDSVPSERERMLLSSISELRANKSLLSAYRCQNFQAERLNGGTRFREIKIQPTTAETESIHWSRTSVSRSGKRGRKKLSCTEPVGERVEKSYQ*

>Bo2g012140.1

MLLKYIYIIMESGSEKKFPLDAKDYKLHEEIGDGVSATVYRALCIPLNEVVAIKVLDLEKCNNDLDGIRREVQTMSLINHPNVLQAHCSFTARHQLWVVMPYMAAGSCLHIIKSSYQDGFEEPVIATLLRETLRALVYLHAHGHIHRDVKAGNILLDSSGTVKLADFGVSACMFDTGDRQRSRNTFVGTPCWMAPEVMQQLHGYDFKADVWSFGITALELAHGHAPFSKYPPMKVLLMTLQNAPPGLDYERDKRFSKAFKEMVGICLVKDPKKRPTSEKLLKHPFFKQARAPDYMAKAILNGLPPLGERYRTIKSKEADLLMQNKSEYEAHLSQQEYIRGVSAWNFNLEDLKNQAALISDDDSSHAEEPDFNRKQCEIQDESAISPERASSSETAPSQDDELNDIHDLESSFASFPSKPLQALKGCFDIDEDEDNETIGTQAHTTKEEDTAAQISSLPRHVISEQKKYSSGSILPEGTFSPKRTSSDAERDFQQRKYQTERSYSGSLYRTKRDTVDETSEAPYVEHKGRFKVTSADLSPKGSTNSTFTPFGGGSSSPSSLNATAVSILQSILQQNAMQREEILRLIKYVEQTSVKQSGSPETNLNELLQTPPATLRERELQTQLMLLQQSFLSLTEEVKKHKQRNGQLENQLNVLTQRND*

>Bo2g062870.1

MDQNSPRSRRTRQTDPKPDIYSTFVVHSDSDSDQDNKRNAKPEDDDDENVDLYATTVYKGDSDGGGDEDDDDDSFLPPLLKRLPKDFGGGASLDYDDDDNGDFGTMIVKKDRDSHSSSKPRVAASPPRRIADEESSEEEEFGTFVVKPSSKKGKEKEKEKEMDLSTMGRAVASMQESSFGGKKNRKSRPSSPSSHRRMQQQNSKMSTTSLPDSITREDPTTKYEFLNELGKGSYGSVYKARDLKTSEIVAVKVISLTEGEEGYEEIRGEIEMLQQCNHPNVVRYLGSYQGEDYLWIVMEYCGGGSVVDVMNVTEEALEEYQIAYICREALKGLAYLHSIFKVHRDIKGGNILLTEQGEVKLGDFGVAAQLTRTMSKRNTFIGTPHWMAPEVIQENRYDGKVDVWALGVSAIEMAEGLPPRSAVHPMRVLFMISIEPAPMLEDKEKWSLVFHDFVAKCLTKEPRLRPTADEMLKHKFIQRCKTGASAMSAKIEKSRQIRASMALQAQKVAASSEDTSTLGLKSSEEMKITVPYTEALPACSLNPQHISSNTALSGEGGDFGTMIFHGEDEADEIHSGSQLAKEKESSSSVGFSGEEVTRIHERKNLPTTDVAVETSTSQSVRGTLSSIEHKKRLSNISGTQTEGGSDASGSTLKNETVGKKAFALQDKLWSIYAAGNTVPIPFLRATDISPIALLSENMIGGMQQDGNGSVAVEALQELFTSDPQSKKGRRGQNEMPLPPSVYQRLTSSPPLMNLAQVLAYHRACYEEMPLQEMQATQEQQTIQNLCDTLRTILRL*

>Bo2g068160.1

MAGSSTKRFPLYAKDYELFEEVGEGVSASVYRARCIALNENVAIKIMDLEKCRNDLDTIRKEVHIMSLIEHPNLLKAHCSFIDRNTLWIVMPYMSGGSCFHLMKTVYPQGFEQPIIATLLREVLKALVYLHRQGHIHRDVKAGNILLHSRGVVKLGDFGVSACMFDSGERMRTRSTFVGTPCWMAPEVMQQVDGYDFKADIWSFGITALELAHGHAPFSKYPPMKVLLMTLQNAPPRLDYDRDKKFSKSFRELIAACLVKDPKKRPTAAKLLKHPFFKHARSTDYLSRKILHGLSPLGERFKKLKEAEAELFKGINGEKEHEYMRGISAWNFDLQDLRKQASLNPDNEMCSSESQVVGVDVPKRNPMIQRSKTMSLEMFKISDKDLMSASNSLTIGPLLPSFRRKFLPAIGYKVGILSDESNACRQRAAEALALEEPNQLELLAVDTKQTPALEELHQLEEPLAVETKQTPALEESHQVEPLAEKPKNGYTVSSVSRCAANEVLPLLQSLLVQNDIQREKVIRLIRFFDGTVSETQNPTSKNEAVQIYPSREKELQSQVNFLEQSVEILVEEVKRRKEINDQLEEQIRSLTSSSSRRSNSRSGA*

>Bo2g109270.1

MWSGGEKKGFSVNPKDYKLMEEVGYGASAVVHRAIYLPTKQVVAIKCLDLDRCNSNLDDIRREAQTMSLIDHPNVIKSFCSFAVDHHLWVVMPFMAQGSCLHLMKAAYPDGFEESAICSMLKETLKALDYLHRQGHIHRDVKAGNILLDNTGEVKLGDFGVSACLFDSGDRQRARNTFVGTPCWMAPEVLQPGSGYNSKADIWSFGITALELAHGHAPFSKYPPMKVLLMTIQNAPPGLDYDRDKKFSKSFKEMVALCLVKDQTKRPTAEKLLKHSFFKNAKPPEICVKKLFADLPPLWTRVKTLEAKDAAQLALKGIASADQEAISQSEYQRGVSAWNFNVEDLKEQASLLDDDDSLAESREEDELCGEQLHNNNIKAKEKKLNPEVEEKTASSAEQTTPSPKCNVPQGKAEPVRRQTQSGPLSPGTLLTNSDSDKGHGYYLRPESERQPAPSAQRAPSFSGPLNLPNRASANSFSAPIKSSGGFRDSMDDKSKPNVVQIKGRFQVTSENLDLARASPLRKSASVGNWILESKMQQPTGQPIKELSNPVSPSFIMPQLQNLLQQNLIQQDLIIKLLNSLQASEATDASQNEKLPPLPRGSDSNAVIELTSSERERLLLNKMSEIRARMKELTEELEEEKSKDTRLQQKLKSVTCQL*

>Bo5g127370.1

MDDVAGLQEAAGARFSQVELIGRGSFGDVYKAFDKELNKEVAIKVIDLEESEDEIEDIQKEISVLSQCRCPYITEYYGSYLHQTKLWIIMEYMAGGSVADLLQSGHPLDETSIACITRDLLHAVDYLHSEGKIHRDIKAANILLTENGDVKVADFGVSAQLTRTISRRKTFVGTPFWMAPEVIQNSEGYNEKADIWSLGITVIEMAKGEPPLADLHPMRVLFIIPRENPPQLDEHFSRPLKEFVSLCLKKVPAERPSAKELIKHRFIKNAKKSPKLLERIRERPKYQVKEDEETPRNGPKAPFESSGTVRVARDERSQGTPGSSFQGKTVKNAGWDFSIGASQGIGTVRALKPPHARERRQEVPSNQTSQRTSRAGGSQLSPTSGITVNDHEDGFHDEEDSSVSGSGTVVIRTPKRSQSSSIFREQISASSSGYASLDDASASGTVVVRGQHDDYASPRTPKSRLGNQDRTSSASEDSIANLAEAKAALDAGFRRGNARERLGMRNNNKDGNVNRRREQMTEDSDYSRNSGDKSGKQKALPKSQQASDEEEEEEEEEEDPIWDSLPASLSVLLIPSLKEALGDDSKGSIGRALSRSLVAMERENPGSCEAFVAKLIELLGSSKEASVKELQDMAVRVFSKTAPADAENKRKPANKEFSSNTNVSPLGRFLLSRWISQSSRDL*

>Bo6g029300.1

MDGVAGLQEAAGARFSQVELIGRGSFGDVYKAFDSELNKEVAIKVIDLEESEDEIEDIQKEISVLSQCRCPYITEYYGSYLHQTKLWIIMEYMAGGSVADLLQPGHPLDEISIACITRDLLHAVEYLHTEGKIHRDIKAANILLSENGDVKVADFGVSAQLTRTISRRKTFVGTPFWMAPEVIQNSEGYNEKADIWSLGITMIEMAKGEPPLADLHPMRVLFIIPRESPPQLDEHFSRPLKEFVSFCLKKAPAERPSAKELLKHRFIKSARKSPKLLERIRERPKYQVKEDEDSPTRDPKSPAESSGTVRVARDDRGHGTSGTSFQGRTIKNAGWDFSIGGSQSAGTVRALKPPQSRERRQEVTSDQSFQKSSRASASQLSSTSGAVVPEISEGGFLKRDSYQNDGQEEDDSSLSGSGTVVIRSPRSSQSSSVFRDLSSGSTSRYTSFDDASTSGTVVVRGQNDDSGSPRTPKSRLGLQERSSSASEDSIANLAEAKVALEAGFRRGNARERLGNGKVNKRREQEKDSSDHLRSSRDDSEKQKPLIRSQQVSDDEDESELASLSASLSLLLLPSLKEAVGGDTSKGSVGHRVSRALVKMEREKPGSSEAFIAKLIEQLGSSKEVSVKEVQDMAIRVFGKTVNNDAENKRKQASKEFASNTNVSPLGRFLFSRWLGQTSRDLNPS*

>Bo6g080900.1

MMLVDHLNLLKSHCSFVSDHNLWVVMPYMSGGSCLHILKAAYPDGFEEVIIATILREALKGLDYLHQHGHIHRDVKAGNILLGARGAIKLGDFGVSACLFDSGDRQRTRNTFVGTPCWMAPEVMEQLHGYDFKADIWSFGITGLELAHGHAPFSKYPPMKVLLMTLQNAPPGLDYERDRKFSRSFKQMIASCLVKDPSKRPSAKKLLKHSFFKQARSSDYIARKLLDGLPDLVNRVQAIKKKEEDMLAQEKMADGEKEELSQNEYKRGISGWNFNLDDMKAQASLIQDIDCGLSESSLSESTTSLQALDSQDMQLETQEDTCQLPNKYLQPLIHRTLSIARDKSDDDISLASPSYDNYVYSSPRHEDLSLNNTTVASAHAINEKSTDSTSITANQPTEILAGSCVSKGESDKTQDQLQNGSTCNGAHPTVGGDEVPTELAVKPPRALSASLDESDDKSKPPVVQQRGRFKVTSENLDIDKVVPSPILQKSHSMQVLGHHSAASLPPSVSGADVPLPNLTSSYVYPLVYPVLQTNILERDNILHMMKVLTNRELTEGRSAEPGSVQQPNVAPTEKSMLEAAHEREKDLLHDITDLQWRLICAEEELQKYKTEHAHV*

>Bo6g108650.1

MAHNSPRSRRSRKPDPTPDLYSTFVVHSDSDSDQGRDPDKPDEDETVDLYATMVYKSDSDEDSMLPPLLKRLPKDFGGGGASYDGDGGFGTVIVKKDSKNSPRVPVVNPADEESSDEGEEYGTFVVVKPCSSKKGKEKEKETDLSTMGRAVASMQESSFGGKNNRKSTPSLQQQKQKQSSKMSTTSLPDCVREDPTTKYEFLNELGKGSYGSVYKARDLKTSEIVAIKVISLTEGEEGYEEIRGEIEMLQQCNHPNVVRYLGSFQGDDYLWIVMEYCGGGSVTDLMNVTEEPLEEYQIAYICREALKGLAYLHSIFKVHRDIKGGNILLTEQGEVKLGDFGVAAQLTRTMSKRNTFIGTPHWMAPEVIQENRYDGKVDVWALGVSAIEMAEGLPPRSAVHPMRVLFMISIEPAPMLEDKEKWSLVFHDFVAKCLTKEPRLRPTADEMLKHKFVQRCKMGASAMSPKIEKSRQIRASMALQAQNVVASSEDTSTLGPKSSDEMGITVPYKPPNNGYQNTTQAPPTSTGEGGDFGTMIVHGEDETEESYAGAQLAKEKESSASQVDGVSVGFSGDQVAGSWIHDKNNRSAADVPVDESTSQSVRGTPPSVSISLEHKTKLNSISRTQTEGGSEASGGTLKSETVGKKAFALQDKLWSIYAAGNTVPIPFLRATDISPIALLSENMIGGMQQDGNGTVAVEALQELFTSDPQSKKGRRGQNEMPLPPSVYQRLTSSPPLMNLAQVLAYHRACYEEMPLQEMQATQEQQTIQNLCDTLRTILRL*

>Bo6g111030.1

MAGTSTKRFPLYAKDYELFEEVGEGVSATVYRARCIALNENVAIKIMDLEKCRNDLDTIRKEVHIMSLIDHPNLLKAHCSFIDRNSLWIVMPYMSGGSCFHLMKSVYPEGLEQPIIATLLREVLKALVYLHRQGHIHRDVKAGNILVHSRGVVKLGDFGVSACMFDSGERMRTRNTFVGTPCWMAPEVMQQVDGYDFKADIWSFGITALELAHGHAPFSKYPPMKVLLMTLQNAPPRLDYERDKKFSKSFRELIAACLVKDPKKRPTSAKLLKHPFFKHARSTDYLSRKILHGLFPLGDRFKKLKEAEAELFKGINGDKEQLSQHEYMRGISAWNFDLEDLRKQASLNPDSEMCSSEVGDEVPKRKPMDLMMSSSNSLTYGAVLPSFHRKFLPAVGYKVGTFSEERKARRGRTAESLALEEPHQPKEPLEDTKQVGTAGSGQEKQKNGYTVSPVNQASCTATEILPLLHSLLVQNDIQREKFIRLIRFFDPTAETENPISKTTQGVQIYISSRERELQSQVDFLEKSVEILVEEVKRRKEINDQLEGQIRSLTSSTSNSLP*

>Bo6g124030.1

MEKKKYPIGPEHYTIYEVIGQGCSALVHRALCIPFDEVVAIKILDFERDNCDLNNISREAQTMMLVDHPNVLKSHCSFVSDHNLWVVMPYMSGGSCLHILKAAYPDGFEEVIIATMLREALKGLDYLHQHGHIHRDVKAGNILLGARGAIKLGDFGVSACLFDSGDRQRTRNTFVGTPCWMAPEVMEQLHGYDFKADIWSFGITGLELAHGHAPFSKYPPMKVLLMTLQNAPPGLDYERDRKFSRSFKQMIASCLVKDPSKRPSAKKLLKHSFFKQARSSDYIARKLLDGLPDLVNRVQAIKKKEEDMLAQEKMADGEKEELSQNEYKRGISGWNFNLDDMKAQASLIQDIDCGLSESSLSESTTSLQALDSHDMQPETQEDIGQLANKHTQPLIHRTLSIARDKSDDESVASPSYDNYIYSSPRHEDLSLNNTTVASMHASNGKPMDSTSVATNQPTEIPAGNCVNKGDSDKIQDQLQNGAHPGDEVPTEIAVKPPKAAASLDESEDKSKPPVVQQRGRFKVTSENLDIDKVVVPSPILQKSHSMQVLSQHSAASLPPSVSGSDVALPNLTSSYVYPLVYPVLQTNILERENILHMMKVLTYRELTDGRSGEPGGFHQPSVAPTEKSMLEAAHEREKELLHDITDLQWRLICAEEELQKYKTEHAQV*

>Bo8g038040.1

MARNKLEFPLDAEAYEIICKIGVGVSASVYKAVCIPMNSTVVAIKAIDLDQSRADFDSLRRETKTMSLLSHPNILNAYCSFTVDRCLWVVMPFMSCGSLHSIVSSSFPEGLPENCISVFLKETLNAISYLHDQGHLHRDIKAGNILVDSDGSVKLADFGVSASIYEPVTTSSGTTSSSLRLTDIAGTPYWMAPEVVHSHTGYGFKADIWSFGITALELAHGRPPLSHLPPLKSLLMKITKRFHFADYEINTSGCGKKKFSKAFREMVGLCLEQDPAKRPSAEKLLKHPFFRNCKGVDFVVKNVLHGLSNTEQMFIESQALIKGVEDDEDDDEEIVKNRRISGWNFREDDLQLSPVFPTTESDTSEFSPREVDPIQDKPEGDKVVITGSEVCLGLSNRNEEAKEQEGEVCGFDRDLVLEKLKLLKKSLEHQRARVLVIIEALSGEKEERNREEELLEMVEKLKIELEAEKMKTLRAEKESVLS*

>Bo9g089620.1

MVSPLRQVIQAFISRRRRKKMASGGGTSSKEKKGFSVNPKDYKLMEEVGNGASAVVHLAIYLPTKEVIAIKCLNLDRCNSNLDDIRREAQTMSLIDHPNVIKSYCSFAVDHKLWVVMPFMAQGSCFHLMKAAYPDGFVEPAICCILKETLKALDYLHRHGHIHRDVKSGNILLDDTGEIKLGDFGVSACLFENGDWQRSRNTFVGTPCWMAPEIMQPGSGYDSK*

>Bo9g165690.1

MSEKKFPLKAKDYKLQEEIGDGVSATVHKALCIPLSEVVAIKVLDLEKCNNDLDGIRREVQTMSLINHPNVLQAHCSFTAGHQLWVVMPYMAGGSCLHIIKSSYQDGFEEPVIATLLRETLRALVYLHAHGHIHRDVKAGNILLDSNGAVKLADFGVSACMFDTGDRQRSRNTFVGTPCWMAPEVMQQLHGYDFKADVWSFGITALELAHGHAPFSKYPPMKVLLMTLQNAPPGLDYERDKRFSKAFKEMVGTCLVKDPKKRPTSEKLLKHPFFKQARPPDYLAKTILNGLPPLGERYRTIKSKEADLLMQNKSEYEAHLSQQEYIRGISAWNFNLEDLKSQAALISDDDSSHAEEPDFNRKQCERQDESALSPERASSSETTPSQDDELNDIQDLESSFASFPIKPLQALKGCFDVGEDEDNATTPDWKDASLMSSGQQHLTKASSIGSLAHTTKEEDTAAQNSYLPRHVISEQKKYSSGSLIPESTYSPKRISMEADREFQLRRYQSERSYSGSLQRTKRDTVDEMSDSPHVEHKGRFKVTSADLSPKGSTNSTFTPFSGGSSSPSSLTTASILPSVQSMLQHNTMQREEILRVIKHLEQTSVKQQPGSPETSVDELLQMTPTTARERELQTQLMLMQQSFLSLTEEVKKLKQRNGQVENQLNALTQRND*

>Cre07.g317300.t1.1

MESEAARSRFQLQSVLGKGAYGTVYAAIDTASGERVAIKVIPVTEQDREEFKQIQREVAFLADCNHPNVVRYLGSYRLSGELWIVMEYCGGGSVSDLLSAGRAPLPEELIAHICCESLKGLAYLHGLGKVHRDIKCGNILLTSSGDVKIADFGVSAQLTNTMSKRQTFIGTPHWMAPEVIQESRYDGKVDVWALGISAIEMAEMTPPRWNVHPLRVIFMISRDPPPRLSQPEKWSPVFIDFVSQALIKDPKQRPAARYLLQHRFAVAQRPGVAAGLAPLIQRAAQYLQDTAAAATQQAQAGAPELKDLAGLATGRFSWRGGTVRAAADKGGGGGGAAAGADPAAATMLAPSRFPGMAGGGGGAAAGPQSPLPVTPGPPAGGAVRRISSSAGFDNTVVVKEDYDYGGTVVVHDGYGTVVVHPEAPAASGGGAAAAAAGGGGGGGGGAGAHYGGHPLLRSPATPAAAAAGAGGGGGGADYGSTMVVAGATLVERYGTVAVRHSDDTVAAPEGGGGGGGGAERSGGSDGDGGGYLAAVRAAAAEAHHRDKHARGSDSPATAAPAPALSEMERVRERLHAIHDGGLVLPLPFLKAEQAQPLALLNPYGAGPVPVGLGGSSGPAGQSGQAQARQGAGRGAAASSARLGPSTAAGVLPTSGFTAPVPADTHGVDPEAYGVVLDLVRQSAAAMVHQQRGEMGGGGGADAAAAAAAIDTLPPTVLTQLLYHSSIQNLARTLAYNRRCLALLPLDRGAAAELADSCESLSAALQCVLSL*

>Cre12.g544400.t1.2

MTAAAADRYERGCCIGRGSFGDVYEGLDTVTGQQVAIKVIDLDDVEDDIQDIHREIQALAGCKCRNITEYYGSVLRPGSAELHIIMELMACSVADLVHHGPLDEASIAYVLAQVLNALVYLHNEHRIHRDVKAANILLSRGGDVKITDFGVSGQLSGTLGYRRKTFVGTPFWMAPEVIDTSEEGYSEKADVWSLGITAIEMATGSPPHSSLHPMRVLFLIPKGPPPALEGDAFSPELKDFVATCLRKDPAARPAARDLLAHPFVAAATQAPEHLPAMVAELARHKKPLSSRRDADEALIAAGGTMPAWDFGTAGAAAAARGKAAAAAAAAVAAAPPAGTVRAMAAAISAASSGGTIRGGAGAEALRETLRASGSAAAGAAAARVETISRTAGVALKEALAAANGSGSAGGSGGGSAPSTGTLRHTNGSAAGPMPPAVPGQYDSLVLPAPPASLPPASAGGAPALFMSGNGGGGAVGAVSSPPRGLPRGAPPPAAKFATMSTAEARMYRDLMGRGAGAGAGATNTSSVGVSSGSVGGGGGAGGAAGTSMGGDGHSGTVVAAGLARNSLSVGTGATVGPAASKAANRYAAAPAFSISSDDSSQQQQPSLPGAEGSGGGSVTSPSAAAAAATASAGALADGYGTVQSRPAGAGAGAGAGAGSDSASASAAPSGSLRSTSATTTTTSGNGAGAAGPSAYGTVQSRKQVEAGPSSGAPSGSAGAAAAHTGTLLLDSSALAGLVAREGSPAESGAVLSRLLGPCARSAFNSADKASQAALAGVLSGLGQLEKMMPGASYRLVQELLVRLSCSAEPTLEPLRASAIGLYATAASASAPASAAATAVRGEGAAAQPPAAPAAAGAALGGVPRAVPDLGPLGEFLLGRWREEEAHEVALLARSQTAGAAAAAGLGMGAPSGTGGSVRR*

>Cre12.g550300.t1.1

MFKSRSAAALHELRAAAAARTYPTDPAQYELLEECGAGVTSTVHRARCVPLGGEVVAIKRCDLTDLADADLQVVIEEVAQMRRYQHPAVLPLLCSFVTAAGELWLVMPYMEGGSVAHVMRYAHPDGLEEAVVATIAREVLRALDYLHKQGAIHRDVKAGNVLLGGDGAARLGDLGVAATMERSGTWGRGRVGRRTLAGTPCWMAPEVLQDCVYDDKADMWSFGITLLEMAHGSAPFANQPPLKVLLNTLQNPPPQLEDRIGARTFSKSMREVVALCLQKEPAARPPARQLLEHRFFRHHARDRAYLVKHLLAGLPPLTERVRQLRAGHGGRVPTRQAHSRELKSRASYMTGVLGWDFNLPCAQPQQQLPQLQPVTELQPSGSPAPPLHQAASGLSCVSSSTTGASLATVTGGGGGSSATSSYSPGANTAAGTGGSSPTAATAPTTPAAAPPSPAGSASTTPPGGGSSIPGGGSSTPGGGSPTSSAGPVLGSRAQQSPSHFHPQPRHWPFLSPGPPSVSPTRQPRPGGGGAASPFGAAVSQAAAASAAAAAGGGSGTDTDHSSMPGTGGARGAAPDEGEEDEEEEWAAAEGVPRRPSLRSSVSSPDLHRRRTQQPPPHALPPPPDLPSVALPQPYQSPKQQQPLLQPQPLLPSLSPAAQPLTPAPAAGAAPAVASPTAVSRSPFSGAGGGGGGPGGGGGCHHSGHHHRVSFAPDVAGLGSSSSGPPPPTPLLGSGGSSGALSVQRKGRFQVIETTQPAAKPQHPPPPWLTSPGAAAASAHARGGGASTASTSGTGAAACGGAGRSSSGGHVFTSPTSTSPLTSPRSHSGQHGQQHQHQHQHQQLTQLPQPQQPLQLARAHSGGKPPLPPRPPPPPAATAAANHPTAAAGASNHPHQLLPMAVGSHGHGHGHGHSSHLLARTPSLRTSTSTSGSSTRSPPPSPPGSVAAAVAASGGGSAAAPPSPPPLSFPSVTSPLLPLPQPPAAPTLPPSSMLPSAPASPFASAVGGMAAAAPPPAVSVGAARSDWLKGLYKRWSDAAVHSLLAAVHRPDESHQLSLALTSAAAPRHVPTIRVTAAPPELAAALVAAAALKPPQSSLTAPRAAPSSAAQSLTSAGTSTEASAAAAAGAPPAKAPSPRLPIITLAHLVPSQPHHWFTPLLDRLMPHHPAAGTPATATSAPATLATDSTGGTGGATVTAAAMERAQQRRLQQAELRGGGGGRHRRLLVPSIYPPLEMDSGWCGCGSEADASASSDSSSGEGGAVDTAGGAMGGAGGEGLWGGHTRGHTDGSTQHTLTGSSTAPSTSTAPSTATGATATGTSGTGSQASGSDSAGHRGNREARAAAAAAAAPSTDVVPAADASASVTTSRSEALQTKHRRPHHHHHHHHHDLHDRGAAGGSMYGSMYGGGGGGGLVVVEVDPSHPDYNALAECYAGAGGTCWAPTARLLQPLLRTLPVELERAQHTCGDGNSGGGSGAAGGGGEAQGAATAAAAALDAENASTTTPVVTPGLATSPTPPLSLPPPPEPLLLPHRPLQPPAASLMPGAPRRTPHAQPSLPTLQEKPAAAPGGTFPTAHDGGAAATIVPSRVPFHGVVPEAAAAAAEAQRRRRQAELQRHLEAALMTFGVAPEEIEELERVQSGGGGDGGAGLWSPKLVPAAAAAAEGKREVAGKAAMERVEEGEEGEEGDSSESTVSDADEEAAEDEPPDGVSPAAPATLASQGRGQGQGDGLDRGGGARSSTTAAAVSRRTTANTAGAPQQAWVPAGPAQHGPQAQAAGPAPAANAAAAVAAPPQTPAQVTLGGSSTRTAGGLGGSSSNSSSSNSTSGSAAASALAQGTGAGAAVAACALQAAATGALSPAAAAQLLPIVSATSLVPSLPSPPHAQAQAQALGAPGEAPPPLQPFQSYWFAVSPPRSAVLPQPPRTGPGGGSSTCWRATAAAPAATAGASPATAAQAASAFEAWRQQGDLAGIPPLAPLAPSSQLQPLQPPPPLPMGLGGAAPLLPRHSSSSANSGSRRSSLNLPPGSHTPHGAGLEGSSPMLEGTGSGGCSNAAVAAAVAAAAAATAATVLGSSGGSGSSGGGGGAHSPGLDSAGVPRPDAAVSAGEDSGGAGPVQLRGGAGVAVTGAADHPGGSGPRGIADGPMTRWHAGGMAPTTPASPNSAMGAGSGVSAPGRLVLPAGPRAPGAAGRLQPPQRHRQPPQTAQERAVAAAAAAATAAAMAAAAAGRGVARGRFVVVNETMQQPQAGRGRG*

>Cre03.g164900.t1.1

MERAISGITSKLSSRKSTKDFSALAGGKEWPTDANQYQILDDCGRGVSATVHRAKCLTNGEIIAVKLMNLESMNCDLDEIIHEAQTMRMYNHPNILPLYTSFVHGQELWMVMPFVAGGSVLHIMKYAYPEGLDEVVIATIGREVLKGLDYVHRNGSIHRDVKAGNILVDGDGIVKLGDFGVAASLERGGSWGHDKQARMTFVGTPCWMAPEVMEQTMGYDFSADIWSFGITLLEMCHGHAPFAKFPPMKVLLMTLQNPAPTLEDKGKRHFSKALKDLVARCLQKESDKRPTAAQLLEHKFFKIARDSKYLKEFLVGNLPALADRVNRIRNGMAATNVTDNDRNLEKSQVGAPPDAASLHADELEEIKAREEYRKGVSSWNFDLAALKAQAALEPDDDSSAHGAASMLPTISESDEREDTLTGTSAAAAQAFLVAQAEEQAAAAAGAGAGGTGAGSAAAAGEAARSFMSAFATVSGQESTARTGSVLPLAAPSPTPAPMTVDVSAPVAAPLRAVASGEGLAGGDRGAMPGYGDMPPPSPGGGVSPAAGLSREGSVGGNMGGMATTPTAVTKQKKGRFEVSEHPAVPVASAAAPHGVVASGSAVALPSLATTSVAGAPHMVSASSAGSLSTYNTFSGPSISGGTVPDLSALRGSATGLADMAGAGGGAALLGGAAAHGEEGLMMGAAAAVAPGPQPTLRTEVEESVTSVEPKQRGRFKIVAEQGAESRPLSKTSSLANLSDAGKSRSDGGGGALLGKPPTGPSGPSSITPPISIMLPKLQELLDHANAHQAALQKLLGAVQECDKGRVPLLLSRAQSTRSLFDGTPGMVLSPAAAGEGAEELRTAMAELRARLATLEDENARLRERNKVLETLHDASQQSAAVAASAGAPAPSGPSYNTSLGGTSVRFNLPDSSGGALQSPTLSVSPKLPAADLPPAPGSGAQPSAL*

>Glyma.01G061500.1

MDRSSFSPGTGKTRTKPDIYSTFVVHDDDDEDSSLPPLLRRLPKDFGGGAPLDYDDDEDEGAGDFGTMIVKSDRSRQRDRSSSGVASPAWKARNPLNRFGAEDDGDEEDDDDGGGFSTFVVRSTVKSGERESVSGTVVRRTSGGSGGLGVGSTMERAVASMQGMGEFGFGKQRKGSGSSQNDEGRHQSITTKVSTSSIPDSVTREDPTTKYELLNELGKGSYGAVYKARDLRTSEMVAIKVISLSEGEEGYEEIRGEIEMLQQCNHPNVVRYLASYQGEEYLWIVMEYCGGGSVADLMSVTDEPLDEGQIAYICREALKGLDYLHSIFKVHRDIKGGNILLTEQGDVKLGDFGVAAQLTRTMSKRNTFIGTPHWMAPEVIQESRYDGKVDVWALGVSAIEMAEGVPPRSSVHPMRVLFMISIEPAPMLEDKEKWSLYFHDFVAKCLTKELRLRPTASEMLKHKFFEKWKSGSAAMLPKLEKARQIRASMASQVQALPVTSEDQELDSKLNDEYGDTVPSRPHNIGVEGAADLSSHGTTRKLHKVEDVDMSEGNFGTVIIHGDELHKTTQDADSAVSVSALTSGTRGRLTDSGIESQKVGIMNTASFRGYSATTNTIQSSLPYVSNSAEQSLKTKGTHRAQAGIGGDISNSIFKNETVSHKAFALQDKLWSIYAAGNTVPIPFLRATDISPIALLSDNVLGGIQQDTGGTGTVEALQELFSGDGQSKKGRRGLNEMPFPPSIYQRLTSSSTLMNLAQALAYHKMCYEDMPLQELQATQEQRTIQNLSDTLRTILRL*

>Glyma.02G119700.1

MDRSSFSPGTGKTRTKPDIYSTFVVHDDEDDDGGGTFRRRKSGGGAQPQDDPYATMVFKDNGHDDEDDEDSSLPPLLKRLPKDFGGGAPLDYDDEDDDAGDFGTMIVKSDRSRQRDRPSSGVASPAWKARSPLNRFGGGDDDGDDDDDDGGGFSTFVVRSTVKSGERESVSGTVVRRTSGGGSVGVGVGVGSTMERAVASMQGMGEFGFGKQRKGSGSSQNDEGRHQSITTKVSTSSIPDSVTREDPTTKYELLNELGKGSYGAVYKARDLRTSEMVAIKVISLSEGEEGYEEIRGEIEMLQQCNHPNVVRYLASYQGEEYLWIVMEYCGGGSVADLMSVTDEPLDEGQIAYICREALKGLDYLHSIFKVHRDIKGGNILLTEQGDVKLGDFGVAAQLTRTMSKRNTFIGTPHWMAPEVIQESRYDGKVDVWALGVSAIEMAEGVPPRSSVHPMRVLFMISIEPAPMLEDKEKWSLYFHDFVAKCLTKEPRLRPTASEMLKHKFFEKWKSGSAAMLPKLEKARQIRASMALQAQALPAASEDQELDSKLNDEYGGTVPSRPHNIGVEGAADLSSHGTTRKLHKVEDVDTSEGNFGTVIIHGDELHKTTQDADSAASVSALTSGSGDRLADSGIESQKVGIMNTASFRGYSATTNTVQSSLPYVSNSTEQSLKTRGTHRAQAGIGGDISNSIFKNETVSRKAFALQDKLWSIYAAGNTVPIPFLRATDISPIALLSDNVLGGIQQDTGGTGTVEALQELFSGDGQSKKGRRGLNEMPLPPSIYQRLTSSSTLMNLAQALAYHKMCYEDMPLQELQATQEQRTIQNLSDTLRTILRL*

>Glyma.05G006700.1

MEHVLEKRFPLNAEDYTLYEEVGEGVSASVYRALCVPLNEIVAIKVLDLEKCNNDLDGIRREVQTMNLIDYPNVLRAHCSFTAGHNLWVVMPYMAGGSCLHIMKSNYPEGFEEPVIATLLHEVLKALVYLHAHGHIHRDVKAGNILLDSNGAVKLADFGVSACMFDTGDRQRSRNTFVGTPCWMAPEVMQQLHGYDFKADIWSFGITALELAHGHAPFSKYPPMKVLLMTLQNAPPGLDYERDKKFSKAFKELVATCLVKDPKKRPSSEKLLKHHFFKQARASKYLARTILEGLAPLGDRFRMLKAKEADLLVQNKALYEDKDQLSQKEYIRGISAWNFNLEDLKSQAALIQDDDIPNAEEPQRDKKQKDRLDNFKVSAERLSAGAANHSDDAPTQDKEDLHLLFQDGFNNLQDLEGSLVSFPTKPLQALKGCFDMCEDDINNSSPRDLDHDGRIDNESSRPSTSLQQNTTSQQKKFPSGSLLPDNFLFPKMVVTDGDRDYLQTKYSSERNHSGPLQYRQKRDTNNLPLVDDTSDGAFFRRRGRFTLTDLSPMGPSNSTSGPVVSPTSPPNQNFMSTAILPSLQCILQHNGLQREEIIKLIKYAEQSSGKNTESVEAGTGDMLQAPPATTRERELHFQVIQLQQSIGSLVEELQRQKMKNVQLEKQLNSMANRVEK*

>Glyma.06G238000.1

MADIAGLAEAAGARFSSLELIGQGSFGDVYKGFDRELNKEVAIKVIDLEESEDEIEDIQKEISVLSQCRSPYITEYYGSFLNQTKLWIIMEYMAGGSVADLLQSGPPLDEMSIACILRDLLHAIDYLHNEGKIHRDIKAANILLTDNGDVKVADFGVSAQLTRTISRRKTFVGTPFWMAPEVIQNSEGYNVKADIWSLGITAIEMAKGEPPLADLHPMRVLFIIPRENPPQLDEHFSRYMKEFVSLCLKKVPAERPSAKELLRHRFIRNARKSPKLLERIRERPKYQIKEDQMTPRNAPRGMGEASDTIKVAKDLRGDETNQPSGQGKSLRSSGWDFSIGGSQGTGTFRSVSRPPQFRDKKIELSNHQLNQRKSPESGYQVGSDNKSAHNESLETYFGKDLGVAYHDEHPDNHLEDQDEFSGNGSGTVVIRSPKGPQPSLFRDQSSRSSSSYASFEDVSTSGTVVLRSQYNDSDSPQTPRSRLGLNSRNSNASLEDSATNLAEAKVAIQGGLRKVNIRERFALGKLNNDVQDSRRGQISSSSDSSRPSREYFDAQKGFSRSHYAIDDEESAKIISSSAPLSVLLIPSLKEAIADDPDGSIVQIVIDALVNMESTKPQSCDVLVKKLLQQLASSKESSLKDLQELAGQIFSKTKSAEENRNAESDNKKKQNKEVHSNSNLSPLARFLLSRWQGQTSRDLNPA*

>Glyma.07G002900.1

MKEKKKYPIGSEHYLLYEEIGQGVSASVHRALCVPFNEVVAIKILDFERDNCDLNNVSREAQTMFLVDHPNVLKSLCSFVSEHNLWVVMPFMSGGSCLHILKSSHPDGFVEVVISTILKEVLKALEYLHHHGHIHRDVKAGNILIDSRGTVKLGDFGVSACLFDSGDRQRTRNTFVGTPCWMAPEVMEQLHGYNFKADIWSFGITALELAHGHAPFSKFPPMKVLLMTLQNAPPGLDYERDRKFSKSFKQMIASCLVKDPSKRPSASKLLKHSFFKQARSSDIIVKKLLEGLPALGDRMEALKRKEEDMLAQKKMPDVKMEELSQNEYKRGISGWNFNLDDMKAQASLIHDFDDAMSDTNHAGSSISLSTLDSQDKQLPSAIHKPSRSADMEENDEMQNLSASVLVVDSAVNDAKFRFEKSDDHSSITCSSHEPQTSSSCLDDHVDNNLGEKPDMENGGRSVEGMATHYYHRSGCSSSILPEVTLPPIPPESEKLQNLHPNVSSCNATSFPQTGEDVLTELPSRVSKSSANSDDTDEKSKVPVVQQRGRFKVTSENVDPEKVAPPPVLQKSHSVQVFSQHNAASTHPTLPLLPASDATPSNLSGCSVFPVLHSVLQINILQRESILSLMKQITAGDSSADGTCNPAQIAITEKSLLEAAHEREKELLHEITELQWRLICTQEELQKLKTENAQV*

>Glyma.08G223700.1

MEKKKYPIGEEHYLLYEEIGQGVSASVHRALCLPFNEVVAIKILDFERDNCDLNNVSREAQTMILVDHPNVLKSHCSFVSDHNLWVVMPFMSGGSCLHILKAAHPDGFEEVVIATVLKEVLKGLEYLHHHGHIHRDVKAGNILIDSRGAVKLGDFGVSACLFDSGDRQRTRNTFVGTPCWMAPEVMEQLHGYNFKADIWSFGITALELAHGHAPFSKFPPMKVLLMTLQNAPPGLDYERDRKFSKSFKQMIASCLVKDPSKRPSASKLLKHSFFKQARSSDTIVKKLLEGLPALGDRMEALKRKEEDMLAQKKMPDGKMEELSQNEYKRGISGWNFNLDDMKAQASLIHDFDDAMSDINHAGSSSSLSTLDSLDKQLPSAIHKPSRSADMEENDEMRRQLASALLVDSAVNDAKTRFEKSDDDSSIASSSHEPQTSSSCPDDHVNNHLGEKSDMENGGRSVEGMATHYYHRRGCSSSILPEATLLPIRAESNTNDSEKPQNLPHNVSSCNATSVPQTGEDVLTELPSRVSKSSANSDDTDEKSKVPVVQQRGRFKVTSENVDPEKVAPSPLLQKSHSMQVFSQHNTASIHPTLPLLPASDATSSNLSGYSLFPVLHSVLQTNILQREGILSLMKQITVGDSSADGTCNPAQVAATEKSLLEAAHEREKELLHEITDLQWRLICTQEELQKLKTENAQV*

>Glyma.10G173000.1

MGSGSRSYSANPADYKLLEEVGYGASATVYRAIYLPYNEEVAVKCLDLDRCNSNLDDIRREAQTMSLIEHPNVVRAFCSFVVERSLWVVMGFMAQGSCLHLMKAAYPEGFEEAAIGSILKETLKALEYLHRHGHIHRDVKAGNILLDDNGLVKLADFGVSACMFDTGDRQRSRNTFVGTPCWMAPEVLQPGTGYNFKADIWSFGITALELAHGHAPFSKYPPMKVLLMTIQNAPPGLDYDRDRKFSKSFKEMVAMCLVKDQTKRPSVEKLLKHSFFKQAKPPELSVKKLFADLPPLWNRVKSLQHKDAAELALKKMPSAEQEAISQSEYHRGVSAWNFDIDDLKAQAALMQDDDDIAEMREEDENKFFSSYKGTTDSQFIVDKKNSNNLQQDEFTSQVGSNDIPKSEKRNGSVAEATPSTLENDVGTSKVKTQSVKLGKTQSGPLMPGLVLGHSSSERGRTFERFENENQLLGEKSNRDIRRAPSFSGPLMLPNRASANSLSAPIKSSGGFRDSLDDKSKANLVQIKGRFSVTSENLDLVKDIPVSSVSRRSSQGSPMRKSASVGDWMVDFKQMQSSNDSANINIPASLLVPHLHNLFQQTSIQQDLIMNLLNSLQTAEAIDTSQNGKLPPLPRNSENNGSVDTAVSEREQLLLGRISELQSRMINLTEELTYEKLRYVQLQQQLALYSQEQNGEREEFA*

>Glyma.12G132600.1

MNSAAVAIKSIKLNRSRPDLDDVRCEAKTPSLLSYPNILKAHCSFTVDRCLWVVMSFMAAGSLQSIIYHSHPNGLMEPYITVVLRDTLNALSYLHCQHLHRDIKVGNILIYTNGQVKLADFGVSASIYESTTTTTTSSSSSLKFTNVVGTPYWMAPEVIHSHTGYSFEADIWSFGITALELAHGRPPLSHLPPSKFMMLKITKRFPFSDDFDDKENMITQLKNAQRH*

>Glyma.12G226300.1

MGNELFVSLLFNLISRFDRELNKLVAIKVIDLEESEDEIDDIQKEISVLSQCRCPYITEYYGSYLNQTKLWIIMEYMAGGSVADLIQSGPPLDEMSIACILRDLLHAVDYLHSEGKIHRDIKAANILLSENGDVKVADFGVSAQLTRTISRRKTFVGTPFWMAPEVIQNTDGYNEKADIWSLGITAIEMAKGEPPLADLHPMRVLFIIPRENPPQLDDHFSRPLKEFVSLCLKKVPAERPSAKELLKDRFIRNARKSSKLSERIRERPKYQIKEDEETPRNGPSGMGEASGTVKVARDLRGEENNRPSDQGKTLKSAGWDFSIGGSQGTGTFRNVSRPPQFRDKITEVSHNQLTQRKAPQSGYQGGSVNRSALNESLESSFGRDLRVPHHDEHLDNHLEDDELSGNGSGTVVIRSPKGSRSSAFRDQSSQSSSSYASFEDASSSGTVVLRGQHDESDSPQTPRSRLGLNDRNSNASMEDSAANLAEAKAAILRKSNAREKLARGKINNDRQESKRDQKASSSDSSRPHGEYDAHKGMLRSHHASDGEESAKIMSSSVPLSVLLIPSLKEAIADDPELVRAVINSLINMEGTKPKSCDVFVKKLLQRLASSKEDSLKDLQGLADQLFSKTKSAQETRNAEADNRKKQQNKEHNSNSNLSPLARFLLSRWQGQTSRDLNPS*

>Glyma.13G001900.1

MGGYSTNPADYKLLEEVGYGATGTVYRAIYLPFNQLVAIKSFDLDRCNVNLDDLRREAQTMSLIDHPNVVRALCSFAVDRSLWVVMPFMDQGSCLHLIKIAYSHGFHEDVIGSILKETLKALHYLHRHGHIHGDVKAGNILLDTSASVRLADFGVSACLYDNAGDRHRSRNTFVGTPCWMAPEMLQPGSGYNSKADIWSFGITALELAHGHAPFSKYPPMKVLLMTIQNAPPGLDYDRDKKFSKYFKEMVAMCLVKDQTKRPSAEKLLKHSFFKHAKPPELSVKKLFADLPPLWNRVKALQLKDAAQLAVKKMPSTEEEAISRSQYQQGVSAWNFDIDDLKAQASLVRDDDDIAEMREEAEKKFFTNYKAIAIDSQSGTVKMNSEKSPQNKITSQVGAFDIKQTEQDEYLNKKEKNPKSDLQEPGLPKNIIWKRNGSITEATTSTLEKDIGMCKIKGQSRKIRQTQSGPLLPGTVLSHSALERGRTLERLENGNQLLGEKNNHEARRAPSFSGPLMLPMRASANSLSAPIKSSGGFIDSLDDKSKANLVQIKGRFSVTSENLDLVKDIPVSSVSRRSSQGSVSPLQKSASVSDWMLDSKQMSTKDSITNGISASFLTTHLQNLLQQTSIQQDLIMNLLNSLQSAEAIEVSQNGKLPPLPRSSEINGSVDTAASERERLLLLKISELQTQMITLTGELTAEKLKHMQLQQQLILYSQEQNRDKRDEVA*

>Glyma.13G274500.1

MADVAGLVEASGSRFSSLELIGQGSFGDVYKAFDRELNKLVAIKVIDLEESEDEIDDIQKEISVLSQCRCPYITEYYGSYLNQTKLWIIMEYMAGGSVADLIQSGPPLDEMSIACILRDLLHAVDYLHSEGKIHRDIKAANILLSENGDVKVADFGVSAQLTRTISRRKTFVGTPFWMAPEVIQNTDGYNEKADIWSLGITAIEMAKGEPPLADLHPMRVLFIIPRENPPQLDDHFSRPLKEFVSLCLKKVPAERPSAKELLKDRFIRNARKSSKLSERIRERPKYQIKEDEETPRNGPSGMGEASGTVKVARDSRGEENNRPSDQGKTLKSAGWDFSIGGSQGTGTFRNVSRPPQFRDKKTEVSHNQLTQRKAPERGYQGVSVNRSALNESLESSFGRDLRVPHHDEHLDNHLEDDELSGNGSGTVVIRSPKGSRSSAFRDQSSLSSSSYASFEDASSSGTVVLRGQHDESDSPQTPRSRLGLNDRNSNASMEDSAANLAEAKAAILRKSNARERLARGKINNDRQESKRDQKASSSDSSRPHGEYDAHKSMLRSHHASDGEESAKIMSSSVPLSVLLIPSLKEAIADDPELMRAVINSLINMEGTKPKSCDVFVKKLLQRLASSKEDSLKDMQGLAGQLFSKNKSAEETQNAEADNRKKQQNKEHNSNSNLSPLARFLLSRWQGQTSRDLNPS*

>Glyma.13G349800.1

MAATNEAEEAAGGKRVQYPLDSSSYKLLNEIGEGVSAVVYKALCIPMNSAAVAIKSIDLDRSRPDLDDVRREAKTLSLLSHPNILKAHCSFTVDRRLWVVMPFMAAGSLQSIISHSHPNGLTEPCIAVVLRDTLNALSYLHGQGHLHRDIKAGNILVDTNGQVKLADFGVSASIYESTTTTSSSSSLKFTDVAGTPYWMAPEVIHSHTGYSFKADIWSFGITALELAHGRPPLSHLPPSKSMMLKITKRFRFSDDFDDKYRKGNGKKFSKAFKDMVASCLDQDPSKRPTADKLLKHPFFKNCKGTDFLVKNVLQGLPSVEKRYRESKGNLHEDDDDGDDDDDDPSMQVKQRRISGWNFNQDGLELDPVFPNDVKEVRFGGETVIQQAAGMEKVNNNSSSREGMLATLNVLKGSLEQELWQVKFLVNTIQGDHHDTQVPEEQEISRLRAQLENERMKNLQLELQLQTSKLHQKSDSN*

>Glyma.19G007300.1

MEHVLEKRFPLNSEDYKLYEEVGEGVSASVYRALCVPLNEIVAIKVLDLEKCNNDLDGIRREVQTMNLIDHPNVLRAHCSFTAGHNLWVVMPYMAGGSCLHIMKSNYPEGFEEPVIATLLHEVLKALVYLHAHGHIHRDVKSGNILLDSNGAVKLADFGVSACMFDAGDRQRSRNTFVGTPCWMAPEVMQQLHGYDFKADIWSFGITALELAHGHAPFSKYPPMKVLLMTLQNAPPGLDYERDKRFSKAFKELVATCLVKDPKKRPSSEKLLKHHFFKQARASKYLARTILEGLAPLGDRFRLLKAKQADLLVQNKALYEDKDQLSQKEYIRGISAWNFNLEDLKSQAALIQDDDIPNAEESQRDKKQKDRLDDFKVSAERLSPGAANHSDDAPTQDKEDGFNNLPDLESSLASFPSKPLQALKGCFDMCEDDVNNSSPRNLDHDGRIDNESSGTSTSLQQNATSHQKKFPSGSLLPDNFLFPKKIVTDGDRDYLQTKYSSDRNHSGPLQYRQKRDTNNLPLVDDTSDGAFVQFRGRFKVTPADLSPMGPSNSTSGPLVSPTSPPNPNFLSVAILPSLQCILQQNGLQREEIIKLIKYAEQSSGKNTESMEAGIVDILQAPPATTRERELHFQVIQLQQSNGILFEELQKQKMKNVQLEKQLSSMINKVEK*

>Glyma.20G067600.1

MGGYSTNPADYKLLEEIGYGATATVYRAMYLPFNQLVAIKSLDLDRCNINLDDLRREAQTMSLIDHPNVVRAHCSFAVERSLWVVMPFMDQGSCLHLIKIALSHGFQEDAIGSILKETLKALHYLHRHGHIHRDVKAGNILLDTSGAVKLSDFGVATCLYDAVDRQRCRNTFVGTPCWMAPEVLQPAGSGYNSKADIWSFGITALELAHGHAPFSKYPPMKVLLMTMQNAPPGLDDRDKKFSKSFKEMVAMCLVKDQTKRPSAEKLLKHSFFKHAKPPELSVKKLFADLPPLWNCVKSLKLKDAAQLAVKKMPSADEEAISQSQYQRGVSAWNFDIDDLKAQASLVMDDNDNAEMREDENKFFTNYKASAIDSQSGTVKMNTEKSPQNKITSLVGAFDIKQTEQNEYLNKKEKNLESDLQEPGLPRNIIWKRNGSIMEATTSTIEKDIGMSHQTQSGLPGTVLSHSASERRRTLERLENGNQLLGEKNNREARQPPSFSGPLMLPTRASANSLSAPIKSSGGFRDSLDDKSKATLVQIKGRFSVTSENLDLVKDIPVSSVSRQSSQGSVSPLRKSASVSDWMLDSKQMATEDSATDSISASLLTTHLQNLLQQTSIQQDLIMNLLNSVQSAEAIEVSQNGKLPPLPRSSEINGSVDTAASERERLLLLKILELQTRIITLTDELTAEKLKYMQLQQQLTLYSQEQNMDKREEIA*

>Glyma.20G217200.1

MGNGSRSYSANRSDYKLLEEVGYGASATVYRAIYLPYNEEVAVKCLDLDRCNINLDDIRREAQTMSLIEHPNVVRAYCSFVVERSLWVVMAFMAQGSCLHLMKAAYPEGFEEAAIGSILKETLKALEYLHRHGHIHRDVKAGNILLDDNGQVKLADFGVSACMFDTGDRQRSRNTFVGTPCWIAPEVLQPGTGYNFKADIWSFGITALELAHGHAPFSKYPPMKVLLMTIQNAPPGLDYDRDRKFSKSFKEMVAMCLVKDQTKRPSVEKLLKHSFFKQAKPPELSVKKLFADLPPLWNRVKSLQHKDAAQLALKKMPSAEQEAISQSEYHRGVSAWNFDIDDLKAQAALMQDGDDIAEMREEDENKFFSSYKGTADSQFIVDEKNSNNLQQYEFTSQVGSNDIPQCEKRNGSVAEATPSTLENDVGTSKVKTQSVKLGKTQSGPLMPGLVLGHSSSERGRTFERFENENQLAGEKSNRDIRRAPSFSGPLMLPNRASANSLSAPIKSSGGFRDSLDDKSKANLVQIKGRFSVTSENLDLVKDIPVSSVSRRSSQGSPMRKSASVGDWMVDYKQMPIGQSSNDSANINIPASLLVPHLHNLFQQTSIQQDLIMNLLNSLQTAEAIDTSQNGKLPPLPRNSENNGSADTAVSEREQLLLGKISELQSRMINLTDELTYEKLRYVQVSFCTSFYISLSVQDFASAILFSCLDYIHVTSNLESI*

>Glyma.U025600.1

MADIAGLAEAAGARFSSLELIGQGSFGDVYKGFDKELNKEVAIKVIDLEESEDEIEDIQKEISVLSQCRSPYITEYYGSFLNQTKLWIIMEYMAGGSVADLLQSGPPLDEMSIACILRDLLHAIDYLHNEGKIHRDIKAANILLTDNGDVKVADFGVSAQLTRTISRRKTFVGTPFWMAPEVIQNSEGYNEKADIWSLGITAIEMAKGEPPLADLHPMRVLFIIPRENPPQLDEHFSRYMKEFVSLCLKKVPAERPSAKELLRHRFIRNARKSPKLLERIRERPKYQIKEDQTTPRNAPRGMGEASDTIKVAKDLRGDETNQPSGQGKTLRSSGWDFSIGGSQGTGTFRSVSRPPQFRDKKTEVSDHQLNQRKIPESGYQGESGNKSALNELLETSFGKDLGVPYHDEHPDNHLENQDEFSGNGSGTVVIRSPKGPQPSMFRDQSSQSSSSYASFEDVSTSGTVVVLHSQHDDSDSPQTPRSRLGLNSRNSNASLEDSATNLVEAKAAIQGGLRKVNVRERFALGKLNNDVQDSKRDQMSSSSDSSSIFFRPSREYFDAPKAFSRSHYSIDDEESAKIISSSVPLSVLLIPSLKEAIADDPDGSIVQIVINALVNMESTKPQSCDVFVKKLLQQLASSKESSFKDLQELAGQIFSKTKSSEETRNAESDNKKQNKEVHSNSNLSPLARFLLSRWQGQTSRDLNPA*

>HORVU2Hr1G047960.1

MQADIWSLGITAIEMAKGEPPLADIHPMRVLFMIPRENPPQLDEHFSKPMKEFVSLCLRKNPAERPSAKELLKHRFIKNARKTPKLLERIRERPKFTVKGSINATQNGQTHIEEDDYGGTIKVERNTKHAASPSSQGTVRKAAGWDFPDRSEGTGTVRAGLRPPQITSTKDGRFDMPQNPSTLKRATDRENQWRTSGTGSEESSSTNMSKKEAQTDHGRLESSTEYNDQSVSGSGTVVLRSPRAPQVYSAGPNHSSKPPSRFSSYEDMSISGTVVRNQSEEAETPRSSRSRLGTQEKTSNASLEDSATNLAEAKAALQAGFRKGNARERPATNKHEKESQEPRSSVVNSHEVRSENVDSQKGRKSRQPSDGQSAPRASAASPALSSLIIPSLKEATGDKYEGPVVHAVLSSLMDLEHEIPGSCEVLVGRILHRLGSSKDSSLQSLNETAISIFTKKPEPPLEAESNKKQASTPPLAAPTVSPLARFLLTRWQNQVSQDLNSV*

>HORVU4Hr1G088910.1

MVRGGSMRRPSLAAAAEPAVPEFTVSPDDYRLMEEVGFGANAVVYRAIFLPANRTIAVKCLDLDRVNSNLDDVRKEAQIMSLIDHPNVIRAYCSFVVDHNLWVIMPFMAEGSCLHLMKVAHPDGLEEPVICSILKETLKALAYLHGQGHIHRDVKAGNILIDSPGVVKLGDFGVSACLFDRGDRQRSRNTFVGTPCWMAPEVLQPGTGYNFKADIWSFGITALELAHGHAPFSKYPPMKVLLMTLQNAPPGLDYDRDRKFSKSFKEMVAMCLVKDQTKRPTAEKLLKHSFFKNTKAPQLTVKSILTDLPPLWDRVKALQQKDAAHLASSEQEALSMSEYQRGVSAWHFDIEDLKAQALLINDDDPPELKEDDDSARVTEIDKGTSFESHFGQSALLNGNNHRLNHERTCTTAVNPGGNGPETSDEFASDLGNADSPRMVDGRITQGTENDSLSSTSKQGSEAGNPRSEVRQRQRTFSGPVMYSGTRSSSLIERGHIIDKDAGGRSLSNKQKSDTGRIDDLSGPLSLSTRASANSLSAPIRSSGGYVGSLGDKPRVEIKGRFSVTSENVDLAKVQEIPVIKISHKPQEVRTQVSTMKKSASVGAWPVKSKSMSNSHHRKEFRDSSVSASILIPHLENLVQQTTFQQDIITNLMSNLQQNEKPNGPQTRVQNMEGDTGVETGSAERERKLLAKVFELQSRMISLTDELIASKLKHVQLQEELNTLYIQEEIADTREDGNGEA*

>HORVU5Hr1G059030.1

MHCYSGPHLSCHLSTFLLITSDHGHPQSSRFSSPALSSSVCCIRNATYVSPVYHYHYHYLHPFLSFPGRPPTDMADDAGAGGEAKYPLNPECYRLLCKIGSGVSAVVYKAACLPLGSVPVAIKAIDLERSRANLEDVWREAKAMALLSHANVLRAHCSFTVGSHLWVVMPFMAAGSLHSILAHGFPDGLPEPCVAVVLKETLRALCYLHEQGRIHRDIKAGNVLVDSDGSVKLADFGVSASIYETPPPASSFSGPLTHVPQVVLSSSSYFSEMAGTPYWMAPEVIHSHVGYGIKADIWSFGITALELAHGRPPLSHLPPSKSMLMRITSRVRMEDAEISKNKKLSKAFKDMVSSCLCQEPAKRPSAEKLLRHPFFKGCRSKDYLVRSVLGTVPSIEERCKDVTSLCGCAAGGARCVSPCHGGQASVVKNRRMSGWNFGADCPRKEDTDSFEELDRTQTAARLFLPLDDEDIVPERACDGAGEDGDKGIMEQQGDREENEEFGVKGVVVPHLVTILESLEVQKRMLAQELEGGCCYHHDGNCCRETTAREEMLLAYVRQLEQRVEVLTLEVEEEITRNXXNHH*

>HORVU6Hr1G029780.1

MGSKGKRGDGGVSSPRSRKGEFPIRAEDYELMEPIGDGATAVVRRARCLPLGGEVVAVKIMNLALRSEADVNNASEEVKTMILTDHPNLLSAYCSFTQDENLWIVMPYMAGGSCFHLMKSSFPKGFEEERFIAFVLRETLRGLEYLHGKGHIHRDVKAGNILLDQHKGVKLADFGISASVYDSMINRNGKRHTLVGTPCWMAPEVMEQKEYDFKADIWSFGITALELANGHAPFSSQPPAKVFLMTLQHAPPSLHNTKDKKFSTSFKRMIGACLIKDPSKRPTARMLLELPFFKKVKSEDNHVKCMLNKVPSLVARVQTIKENEAKLQAEKKPHDKIKEKTSHDEYWRGISQWHFDIEDLKAQAKLYSEENDSDGEEYLHFLFELDTVDETVPLKDVHRQKHIVGNEITEMPSSTTPVPIPQSGKQLENGGPNGLVRHESFERHSKVPTKQLSRAVSNATGIDEYLEKTTIQKGRFKVTMEETEVSTPREKELLERIACLERMLQVTQDEIVKLKEKEAKGAVPCIQQNSKVQAM*

>HORVU6Hr1G084460.1

MAPEVMEQLHGYDFKADIWSFGITALELAHGHAPFSKYPPMKVLLMTLQNAPPGLDYERDKKFSRHFKQMVAMCLVKEPSKRPTAAKLLKQSFFKQARSHDYIARKLVDGLPGLGARYQALKEKDEHLLAQKKMPDGRKEEISQDEYKRGISSWNFDIDDLKSQASLISECEDTISSKDTDISSIYDFDTSLQEQGHEGSLFSMKYDTDIENDVMANDKSAVSSPEQSVCLSRASLCGTSNGVLVNGHVGKLNSMESCDLDLQEKDLDAIPTSSFQERKFSFSSCSSDGFLSSKESSKPQINIHNRDKGSGGVLQVADEPSPEAISKAPKSSVSNVDEHDDRPKPPLIQQRGRFKVTPGNVELDKAHSPGLHKSHSTQTISHLPALSIPSSAEAASSIIGGSLYVQLYSVLQTNMLQREQILHAMKQLYISDSISPVRMPSLSRSPSQSSALSVDRSMLEAAQDKEKELINEVLELQWRLLCAQEEVQRLKAKAAQI*

>HORVU1Hr1G048230.1

MGRNGSVKRGAAAAPPPSFTLNPADYRLMEEVGYGAHAVVYRALFLPRNHTVAVKCLDLDQLNNNIDEVQREAQIMSLIDHPNVIRAYCSFVVEHSLWVIMPFMTEGSCLHLMKIAYPEGFEEPVIASILKETLKALEYLHRQGQIHRDIKAGNILIDSAGVVKLGDFGVSACMFDRGDRQRSRNTFVGTPCWMAPEVLQPGTGYNFKADIWSFGITALELAHGHAPFSKYPPMKVLLMTLQNAPPGLDYDRDRRFSKAFKEMVAMCLVKDQTKRPTAEKLLKHSFFKTAKPPESTMKGILTDLPPLWERVKALQVTTILLLFCLNNRSNVFSYLMLLTYFQLKDAAQLALKKMPSSEQEALSLSEYQRGVSAWHFDIEDLKAQASLIYEDEPSEMKEDDVAARITESEKSLYSRNPSGQSPSANENACSEETCTTNPDCRRMPNGHANSRSENDSLPSTSKEPESKYWRTNIGQKQQTSGAPVEGGVNSSTTERSHNFERDATGDKHGSDTRRAANLSGPLSLPTRASANSYSAPIRSSGGYVDSLGDKSKRSVVHIKGRFSVTSENVDLAKVQEIPLSSISRKSPHGIQLRKSASVGDWIVNAKPTSNSHHLKELCNSSVSSSILIPHLENLVQQTTLQQDLIVNLLSSMQQNEKADGAQSGTSSQVRNMQSGTVVETSNTEKERSLLVKISELQSRMITLTDELISAKQKHVQLQQELNALYCREEIEDIRD

>HORVU5Hr1G095970.1

STFPVLLRSHPPPMAFSPRSPWSRSKKPDIYSTVVVHDDEDDAHGGGAARAEDDDDDDPSALPPLLQRLPKDFGGASFDDDDDPYSSDLDDASLSATVVIKRGAPASTSSSSRSPFLDLRRSSPRAAEADPYSTFVVHGTARSGGASSPRESVSGTFIRHSGGPPSPRESVSGTFIRHTRGSSSPRESYSGTFIHHTSGASSPRDPASGAGAGFGSSFMTPSTGQAEEDRQPSLLMQQQQSRRQASMSSVPDSVTREDPSTKYELLHELGKGSYGAVYKARDLRTQELVAVKIISLTEGEEGYEDIRGEIEMLQQCSHPNVVRYFGSYQGEEYLWIVMEYCGGGSVADLIGITEEPLDEPQIAYICRETLKGLAYLHTIFKVHRDIKGGNILLTEQGEVKLGDFGVAAQLTRTMSKRNTFIGTPHWMAPEVIQESRYDGKVDVWALGVSAIEMAEGMPPRSTVHPMRVIFMISSEPAPMLEDKEKWSLLFHDFIAKCLTKDARLRPAAIEMLKHKFIEKCNTGASKMLAKIKEAKIIRETAVQNQLPDSDDAMDATVRINEDYGETVPTNSQSTHETKNDGSGGDFGTMIVHPEDGDEAVESSIFPRAEFIPGLGSINSFTHDPKRAELISKFWAENTADSDATKGRDLDGLPDTQEPKAMPRSIGTFKHHKGVEGTVLRHDNTASPGVASTMTKLSSSPSRKAFSVQDKLWSIYAAGNTVPIPFLKAIDISPLALVSDSVAGNGPAGSSTTDALEAVRELFSGDGQAKKGRKGQNEVCCRLFLSSVAKRHYFSCPDVCLILDLIALGPSSSRCARQIDDKPYIDEPGAGSRLP*

>HORVU7Hr1G047720.1

MAHAPRNRGFPTDPKEYKLYEEVGEGVSATVYRALCVPLNTFVAIKVLDLEKCSSDMDGIRREVQTMSLINHPNLLRACCSFANDHHLWVVMPFMAAGSALHIIKTNFPDGFEEAVIATLLREVLKALVYLHSQGHIHRDVKAGNILIDTNGDVKLGDFGVSACMFDTGNRQRARNTFVGTPCWMAPEVMQQLHGYDYKADIWSFGITALELAHGHAPFSKYPPMKVLLMTLQNAPPGLDYERDKRFSKYFKDLVSTCLVKDPQKRPSSEKLLKHSFFKQARSADFLAKNIVEGLPPLGDRFRALKVQNSV*

>MCO05G399.1

MALTSERFELGPRIGRGSAGDVHTARDKLTNDVVAVKLVDLEEAEDEVEDIQREISYLVQCSSPFVTKYLGCWLDPGSTRLAIAMEYMAGGSVADLIRDDLGGPLSESACAVICRDLLYALDYLHGEGKIHRDVKCANVLLTATGEIRLADFGVAGTLTQTLGGNKRRTFTGTPFWMAPEVIQAHESDGYNSKCDIWSLGITAMEAANGTPPYSDLHPMRVLFFIPKNPPPRLEGGNFSDAFKEFVAACLQKDPDRRPRANQLVNHRFIAHAPERCDELTDRVNKRMHGGVDRMTSSSVGGDDVGVETTETRHEAAPSWDF

>MCO12G367.1

MEPAGRPEDLFELLEQLGKGSYGAVYKARHRPSGTIVAVKVIPLSGEDEEGLEDIRREIAVLRECVHPNVVRYFGSFTGTEYLWIVMEHCGGGSVRDILSASNRPLREAQIAYLCGETLKGLVYLHSIFKVHRDIKCSNILLTESGGVKLADFGVAAQLTRTMSKRNTFIGTPHWMAPEVIQESRYDGKVDVWALGISAIEMAEVQPPRHNVHPMRVIFMITREPSPRLDESPFVDKSGERTEWSPAFHDFVAQCLRKETSRRPTATELLPHRFLQSSAGSGAGLVPMIVAAKRWKSKEEEDGEGGGGGGGGEEAREGEEKRREEEKRRAAEAGASGAKPAASSSAPVRSTDLSSRTDQGSTMSTDTGSVIVKDIAASSAPVGGETNGPATTNGPAGPPGRPGHARKHSSATIRFDPSMAAAAMAAAEAALDSMSPPQSPAGTQTKHPPLPAPEVFVSLEDDDEKKKNGNRTDDDGGDGAVVGSIPGDGDDDGAWASPRVSDAGTVVIAPSPRGSDGSAADRTSQSSGKIDGNFPDFPDESHEPASRRDSEYFESVESFDVNATMIERGDDGEDEPATGTREQPPPSPPPPPPPARPAPSSPPSHRPAGPSPRSPPPEPARESTTVLPGGAESESGLDPTLLMRMFVGRELTAEEEAGARDGGGIPTTAYAPPAPPDEPEPSRRSEPEPSRPAPEPPEPPEPQPAPEERRGSDPMAGRSFFAENPDIRRLLPDGTIDVAGLLKAYAGIEIVERRPEDVTYDERSSPWHPANAMRFALQNFLVDHETGEPLNPNADHSLSLDGYLRCALRADEPPSLPTSETELREECEEYHAYLDELANEESMKIFMDPTLDSESEANESDDDEWSARYEVRAVPGGGWATEEALEWRARDLARRHRELMRDGVIEPELRESDLDIRGYEPTIAQICAANANDEPLVSPDNIPLDHLMEEDPMALAVAYCDAAHLPRDVARSMLDDRSGACERLVRCLAWKTGRGGGASALRFDRRACRRLRRALVWYLGTLEEEAEGGEGG*

>MCO16G480.1

MLSSSSAALRRNNSFFESTPKVQYPTQASDYRLMEEIGRGVSAKVYRAECVPLNNEQVAVKKLDLEDQDPGHLEEIRREVASMSMLSHPNLVMAHCSFVEGQYLWIVMPFCGGGSALNIMKWSHPKGLDETSIATILKEVLKALDYFHRNGNIHRDVKAGNILIDDNGSVKIGDFGVSAASWGSGAKPHATFVGTPCWMAPEVMEQVNGYDWHADIWSLGITVLELCHGHAPFAKYPPMKVLLMTLQNPPPQLEAEQAESGHHFSRALRDFVSICLQKDPSKRPSAAKLLEHKFLKEAKKPDFLVKHLLDGIPTLGDRTAKLNEREKARQAQRAAAVAAGGLSSAPSTEAAEEKRSNAEYLKGVSRWDFDMDAIRAEAAAMTLDDD

>Mapoly0008s0135.1

MDAPKGAGAGGGDERRSYPVTAADYKILEEVGQGVSATVYRAQCTPFNETVAIKSLDLEKCISNLDEIRKEAQMMSLIAHPNVVKAYCSFVVDRSLWVVMPYMAGGSCLHIMKSAFSDGFEEPVIATVLKEVLKALDYLHRHGHIHRDVKAGNILVDSNGQVKLGDFGVSACMFDTGDRQRSRNTFVGTPCWMAPEVMEQLHGYDFKADIWSFGITALELAHGHAPFSKYPPMKVLLMTLQNAPPGLDYERDKRFSKAFKEMIAMCLVKDPAKRPSAEKLLKHSFFKHAKTFEYIARHVLDGLPPLGERVKNLKAKDAARLAQKKMPYGEQEERSQNEYKRGVSSWNFNVEDLKAQAALLDDEDATQPSSKDLAADSPRSRPKDSSSEDGRDAGSDSQSGWSLPSDTRDFLPASGPLYSPRADSGPPPERVPRARSGPLPSGAQVAKASNGGKPGPGPNLGRKDSSQRHSVGRFEVEDALDLDRAEWHDDSRVRAGEDRDQSRRSDEPDWERDGGGGGSFKSRDDGREGARGGEELPRINVFRDRERISSMSMPVMCGSGGGGSKDVPDEKLKGTFVQKKVAAIFPHLQNLLHHCVSQQELVLNLINSMTVAGDPPPPATGASRLSHMGSKSFMSSSNSLSISTSEASLDVSTEKERDLLDQVSGLQSRVASLVDELQVVKLRNVQLERQLNAIYNKEEEERIRKEEAAKEEG*

>Mapoly0102s0039.1

MAAALKRPESPASDIFSTVVFKSEDDTPYATVVHKSDDTYDTVVHRVGGGGTANASFNGEAGGSGTVKMAAIAKPQIATGAAKYEPRERTRTRSMRSPVHEKVMQEDPSVKYDLLNELGKGSYGAVYKARDLRTSELVAIKVISLCEGEEGYEEIRGEIEMLQQCNHPNVVRYLGSYQGEDYLWIVMEYCGGGSVADLMSITDEALEEHQIAYICKESLKGLEYLHSIFKVHRDIKGGNILLTEQGEVKLGDFGVAAQLTRTMSKRNTFIGTPHWMAPEVIQESRYDGKVDVWALGVSAIEMAEGLPPRSNVHPMRVLFMISREPAPMLEDKEKWSLVFHDFVAKCLTKEPRLRPTATAMLQHKFIDKCKGTAMSMLPRIERARAIKVEMVAQQLAYNQEQGTASSGGGQWSWEKGQTVKMNESFGGTMLIRHDADTLQKGASLDSEQEDGDFGTIVVHGTSKKHDSASGTPVTAEDGQEPNSENGSPLLSPVQTPDISSEVGSTVRGRNLNVPPIVPPSPRSPSTSPVSSPKQPAIDGILGSTLRRIAGDGLATATGTPKGTPTVNRQGFALQDKLLSVYAAGNTVPIPFLKATDISPIALISDNISGSGQPDHSGVVALEAIQELYNGGGLGDGQQRRGRKPLGNEMPLPPSVYQRLATSTTLPNLARALAYHKLCYEEVPLQGWQVAQEQQTIHNLSDTLRTILRL*

>Mapoly0133s0001.1

MASSSSNVGMSNSIESRFTNLQLIGKGSFGDVFKGFDKELNKEVAIKVIDLEEAEDEVEDIQKEISVLSQCRSPYVTEYYGSYLHSTKLWIVMEYMAGGSVLDLLETGPPLDEMSIACILRDLLHSLEYLHGEGKIHRDIKAANILLTANGDVKVADFGVSAQLTRTVSKRKTFVGTPFWMAPEVIQNSDGYNEKADIWSLGITAIEMAKGEPPHAELHPMRVLFNIPKNPPPQLDEHFSRQMKEFVSLCLKKNPAERPSAKELLKYRFVKNARKSPRLLERIRERPKTHIGKSKDLPRPDQQYEKEAAEKKKVIKSTGEKDAQYRRKAIRNASWDFGTTTIQSTGTIRSALKPTYSTSHNEGNTSTEALPSLLVETEKEWNIYEGLSELRGSSRENGDQGELPRMQEEDEGEDPIEDGRMSVSNSGTMVIRSPKERTSLMSLASQLSSLVGSPQERASLMSLASQLSSLSTSGDASKEEASEMFSSGTIVTRNLPEDTSSSTVTTPKLRPSSQEGPFAASREDSATNLAEAKAALQAASRKNMRGVPSFKGAGVPLGSSQNLNQSGEEAGVERKVTRAPSRAAARRSTEEEDAARAAAAGASPALSLLLIPALKETAAEQSEGPALRAAADAADSLMDLERLAPGACEVLLRRLLQRLGSTDEAALKGLQDLARKVLIIPTEEKAQSQEEAAVGGQFGFGKSGFGRNRANPDAAENSGLSPVAAFLLSRWQVQVARDITPHRK*

>Medtr1g077800.1

MGSGSRSYSANPQDYKLLEEVGYGASATVYHAVYLPYNEEVAVKSLDLDRCNSNFDDIRKEAQIMSLIDHRNVVKSYCSFVVDRKLWVIMPFMAQGSCLHLMKSAYPDGFEEDAIGSILKETLKALDYLHQHGHIHRDVKAGNILLDSNGEVKLSDFGVSASMFDTGDRQRSRNTFVGTPCWMAPEVLQPGTGYNFKADIWSFGITALELAHGHAPFSKYPPMKVLLMTIQNAPPGLDYDRDRKFSKSFKEMVAMCLVKDQTKRPSVDKLLKHSFFKQAKPPELSVKKLFADLPPLWTRVKALQLKDAAQLALKRMPSAEQEAISQSEYKRGVSAWNFDVDDLKAQASLVQEEDDDIAEIKEEDENKFFNSNKDTSDSQFGVDKRNSDNSQENEFALQVGGSNVPALHVGGNDILQSDKRNGSIAEATTSTSEKDMGTGKIKTQAVKVGKTQSGPLVPGTVLGHYLPEKGRLFERFENENQLPGEKINRDMRRAPSFSGPLMLPNRASANSLSAPIKSSGGFRDSPDDKSKANLVQIKGRFSVTSENLDLVKDIPASSVSRRSSQDSSPLRKSASVGDWMLDFKQQMPTGQSSNDSANLNVPASVIVPHLHNLYQQMSIQQDLIMSLLSSLQTAEPNDASQNGKLPPMPRSSENNGSVDTATSEREQLLLAKISELQSRMVSLTDELTSEKLKHMHLQQQVAAFYSQQQNGEREEGA*

>Medtr2g100030.1

MATNIAERVQYPLDSSSYKIVDEIGAGNSAVVYKAICIPINSTPVAIKSIDLDRSRPDLDDVRREAKTLSLLSHPNILKAHCSFTVDNRLWVVMPFMAGGSLQSIISHSFQNGLTEQSIAVILKDTLNALSYLHGQGHLHRDIKSGNILVDSNGLVKLADFGVSASIYESNNSVGACSSYSSSSSNSSSSHIFTDFAGTPYWMAPEVIHSHNGYSFKADIWSFGITALELAHGRPPLSHLPPSKSLMLNITKRFKFSDFDKHSYKGHGGSNKFSKAFKDMVALCLNQDPTKRPSAEKLLKHSFFKNCKGSDVLVKNVLNGLPSVEKRYKEIKAIMDPDSKCKDDGDDDDDESVKNVKQRRISGWNFNEDGLELVPVFPKDQSKDDEVVKQVRFEEEKVIQEDAVVTASGTVMEPKTNTSDVVDHENVGGVVKNREATLATLNVLKESLEQELGQVKFLMNLIGGEEIHVAESDEKMVQEISKLRTELENEKKKNLQLEMQLENIKLHLISSAANSPTS*

>Medtr5g045190.1

MDRGSFSPRTRNTRPKSDIYSTFVIHNDDDDDGETNRRRKTASAEAQEDPYATMKFKDNGHDDNDDDEDEDSFLPPLLKRLPKDFGGGASMDYDDDEDESGDFGTMIVKNDRSRQRDRSSSGVASPAGLTWKTGNSSQVTALNDEDDDDGGGFSTFVMRSTVRNSERESVSGTMVRRTSGNDGGGGSTMERAIASMQGVGEFGKQRKGSGSSQNEAITTKVSTSSIPDSVIREDPTTKYELLNELGKGSYGAVYKARDYRTSEMVAIKVISLSEGEEGYEEIRGEIEMLQQCNHPNVVRYLGSYQGEEYLWIVMEYCGGGSVADLMNVTDEALDEGQIAYICREALKGLDYLHSIFKVHRDIKGGNILLTEQGDVKLGDFGVAAQLTRTMSKRNTFIGTPHWMAPEVIQESRYDGKVDVWALGVSAIEMAEGVPPRSSVHPMRVLFMISIEPAPMLEDKEKWSLYFHDFVAKCLTKEPRLRPAASEMLKHKFFEKWKVGSAAMFPKLEKARQVRASMALQVQTLAPAAAGDQEPMIASILNDEYGDTVPSRPQNMGVEEAAYISSHGTTRKLQRVDEVDNSEGNFGTMIVHGDEFKTTQDTYSASYKTAFTSSTGGRLSDPGVGSLKVGDMNTASFRGSGPTADTIQPALPSVSNSTEQNLKTKGTYRAQAGIGSDIGNSTLKNETVNRKFALQDKLWSIYAAGNTVPIPFLRATDISPIALLSDNVLGGMQQDTGGTGTVEALQELFGGDGQSKKGRRGINEQMPLPPSICQRLTSSSTLMNLAQALAYHKMCYEDMPLQELQATQEQRTIQNLSDTLRTILRL*

>Medtr6g006770.1

MERISEKRYPLSAKDYKLYEEVGEGVSASVYRALCIPLNEIVAIKVLDLEKCNNDLDGIRREVQTMSLIDHPNLLRAHCSFTAGHSLWVVMPFMSGGSCLHIMKSSFPEGFDEPVIATVLREVLKALVYLHAHGHIHRDVKAGNILLDANGSVKMADFGVSACMFDTGDRQRSRNTFVGTPCWMAPEVMQQLHGYDFKADIWSFGITALELAHGHAPFSKYPPMKVLLMTLQNAPPGLDYERDKRFSKSFKELVATCLVKDPKKRPSSEKLLKHHFFKHARATEYLSRTILDGLAPLGDRFRQLKAKEADLLMQNKALYEDMEQLSQKEYIRGISAWNFNLEDLKSQAALIQDEDMSNAEEPDMAKKNEDTSNDLKVPVENLSASNHSDGTSTLDKEDGFNNLQDLESSLASFPMKPLQALKGCFDVGDDDGNNTSPRDLDHSYGRNDNESAGPSNLSPQNAQPNKFLSGSLQSDNFAKKVTDGDRDYLQTKYPSERNHSGPLYRQRREINNFPSVDDTSEGAIVQRRGRFKVTSADPSSMGLSNNTSGPVAVSPTPTSPPNQNSMAASILPSLQCILQQNGLQREEIVKLIKYAEQSYGKNTESIEAGASDALQASPVSTREKELHLQVIQLQQSIGSLVEELQRQKLKNVQLERQLSSMVNKVEK*

>Medtr2g078740.1

MEDLAGLVEATGTRFTSLELIGQGSFGDVYKGFDKELNKEVAIKVIDLEESEDDIDDIQKEISVLSQCRCPYITEYYGSFLNQTKLWIIMEYMAGGSVADLLQSGPPLDEMSIAYILRDLLHAVDYLHNEGKIHRDIKAANILLSENGDVKVADFGVSAQLTRTISRRKTFVGTPFWMAPEVIQNSEGYNEKADIWSLGITAIEMAKGEPPLADLHPMRVLFIIPRENPPQLDEHFSRPMKEFVSSCLKKVPAERPSAKELLKDRFIRNARKSPKLLERIRERPKYQIKEDLEASRNGARGMSEASNTMKVARDSRVEEISRPSQVKTLKSSEWDFSIGESEGTGTIRHASRPPQSRDKKTDVSYNQLTQRKALDAGYQGGYANRSAPNQSLESSLGKDPRAPYHHEHPDNQFEDDELSGNGSGTVVVRSPKGSRPSVFRDHSSQDELSENGSGTVVIRSPKGSKPSVFRDQSSQSSSSYASFDDSMSGTVVLRSQHDDSDSPRTPRSRLGLHDRNSNASLEDSAANLAEAKAAIQGGRKVNARERHSRGKINSDIQESKRDQMTSSTDSSRSYREYIDAQRGMSKSHYASDDEESARILSSSAPLSVLLIPSLKEAIADDPEGPIMRAVINSLINMEGTKPRSSDVLVKKLLQRLASSKEDSLKDLQGLASQLFSKAKLTEETQNAEADNRRKQHSKEPHPNSNLSPLARFLLSRWQGQTSRDLNQS*

>Medtr4g005730.1

MKDKDRDKEKETEKKKYPIGAEHYQLYEEIGQGVSASVHRALCVSFNEIVAIKILDFERDNCDLNNISREAQTMVLVDHPNVLKSHCSFVSDHNLWVVMPFMSGGSCLHILKAAHPDGFEEVVIATVLREVLKGLEYLHHHGHIHRDVKAGNVLIDSRGAVKLGDFGVSACLFDSGDRQRSRNTFVGTPCWMAPEVMEQLHGYNFKADIWSFGITALELAHGHAPFSKYPPLKVLLMTLQNAPPGLDYERDKKFSKSFKQMIACCLVKDPSKRPSASKLLKHSFFKQARSSDYITRTLLEGLPALGDRMEILKRKEDMLAQKKMPDGQMEELSQNEYKRGISGWNFNLEDMKAQASLINDFDDAMSDISHVSSACSLTNLDAQDKQLPSSSHSRSQTADMEESDEMHNQLASVPEVDSTINDVKTRIEKSDDDSSITSSSHEPQTSSCLDDHVDHSLGDMENVGRAAEVVVATHPPLHRRGCSSSILPEVTLPPIRAESEKLQNLSTNVSSANAILVTHTGDDVLTELPSRASKTSANSDDTDDKAKVPVVQQRGRFKVTSENVDPEKATPSPVLQKSHSMQVGCLEVMSQHNATPLHSPLPLLSPISDATPSNISCCSLFPVLHSVLQTNILQRETILTLMKQITVGESAADNTNAPAQIAAMEKSLLESAHEREKELLHEITDLQWRLICTQEELQKLKTDNAQV*

>LOC_Os02g08240.1

MEKGKRRGGSSSSSGPQAVARKRDQFPSRAKDYELLEPVGDGATAVVRRARCLPLGGEVVAVKIMNMSQRSEDDVNHASEEVKMMSTIDHDNLLGAYCSFTEGETLWIIMPYMAGGSCFHLMKSSYPKGFDEKFIAFVLRETLEGLAYLHRYALVHRDVKAGNILLDQHKGVKLADFGASASLYDPMINRHGKRKTLVGTPCWMAPEVMEQKEYDAKADIWSFGITALELAHGHAPFSTQPPAKVFLLTLQHAPPSLHNTKDKKFSKSFKQMIATCLMKDPSKRPTAQHLLELPFFKKVKFEDNVLKSVLNKLPSLGDRMQSIQENEAKLQAEKKPLDKCKEKASQDEYMRGVSEWNFDIEELKAQAALYPDENDGGEDEYLRFLFELDTICESAPIHDVQSRDYSKNENEKVFQICIL*

>LOC_Os02g54900.1

MAKAWEKVATAAGLGGSGERRKYPIRVEDYELYEEIGQGVSAIVYRSLCKPLDEIVAVKVLDFERTNSDLNNIMREAQTMILIDQPNVMKAHCSFTNNHSLWVVMPYMAGGSCLHIMKSVYPDGFEEAVIATVLREVLKGLEYLHHHGHIHRDVKAGNILVDSRGVVKLGDFGVSACLFDSGDRQRARNTFVGTPCWMAPEVMEQLHGYDFKADIWSFGITALELAHGHAPFSKFPPMKVLLMTLQNAPPGLDYERDKKFSRHFKQMVAMCLVKDPSKRPTAKKLLKQPFFKQARSSDFISRKLLEGLPGLGARYLALKEKDEVLLSQKKMPDGQKEEISQDEYKRGISSWNFDMDDLKSQASLITECDDSISCKDSDASCFYDLDTILPERATGPHMSRVFSIKYDTDTEYINAQDYKRGTCKWPGQETQLHRNSKHQINIHNRDKCNGGPLQVADEPSPEAVPKVPKSSAANVEDHDDRSKPPLIQQRGRFKVTPGHVELDKAHSPGLQKSHSMQAISHLPSLSIPSSIEAASTIIGGSLYMQLYNVLQTNMLQREQILHAMKQLSGCDMAMTSPACIAPASRASSPSSALSIDRSLLEAAHEKEKELVNEITELQWRLVCSQDEIQRLKAKAAQI*

>LOC_Os03g47470.1

MAAAAGSVGGDDHHHHQQARYPLDAGSYRLLCKIGSGVSAVVYKAACVPLGSAVVAIKAIDLERSRANLDEVWREAKAMALLSHRNVLRAHCSFTVGSHLWVVMPFMAAGSLHSILSHGFPDGLPEQCIAVVLRDTLRALCYLHEQGRIHRDIKAGNILVDSDGSVKLADFGVSASIYETAPSTSSAFSGPINHAPPPSGAALSSSCFNDMAGTPYWMAPEVIHSHVGYGIKADIWSFGITALELAHGRPPLSHLPPSKSMLMRITSRVRLEVDASSSSSEGSSSAARKKKKFSKAFKDMVSSCLCQEPAKRPSAEKLLRHPFFKGCRSRDYDYLVRNVLDAVPTVEERCRDSTQLCGCARGARCVSPCRHASSGSNVVAAKNRRISGWNFNEESFELDPTDKPPEQQQQQPCFPFHHDNDDDMVEHEQEQRRRQDGNDGSSDVAVPHLVTILGSLEMQRDMVMQVLEGDGGGGGETAGREEMLVGYVRELEKRVQELSTEVEEEMARNAHLQELLHERACENHTDSSHTSGSR*

>LOC_Os03g54780.1

MAFSPRSPWSRSRKPDVYSTVVVHGDDDDDDARGGGGGGRHALAEDDEEEDPSSLPPLLQRLPKDFGGGASFDEYDDPYSSDLDDASLSATVVVKRGAPASTSASSRSPFLDLRRSSPRAAEVDPYSTFVVHGTARSGGASSPRESASGTFVRRSGGSSSPRESVSGTFIRRTGSPSSPHESISGTFIHHTSGASSPRDTAQGGGGFGSSFWSPAVGQSEELRQPALLVQQQHQQQQNSRRKPSVSSVPESVTREDPSTKYELLHELGKGSYGAVYKARDLRTQELVAIKIISLTEGEEGYEDIRGEIEMLQQCSHPNVVRYFGSYQGEEYLWIVMEYCGGGSVADLIGITEEPLDESQIAYICREALKGLAYLHSIFKVHRDIKGGNILLTEQGEVKLGDFGVAAQLTRTMSKRNTFIGTPHWMAPEVIQESRYDGKVDVWALGVSAIEMAEGMPPRSTVHPMRVIFMISSEPAPMLEDKEKWSLLFHDFIAKCLTKDPRLRPAASEMLKHKFIEKCNPGASKMLAKIKEAKKIRAKVAAETELSGPDSDATVRINDDFGETVPTNPQQQTNHETYDGGAGDFGTMIVHPEDGDEVDESPIFPSSEFIPGLGSINSFTHDPKRAELISNFWAENTADIEANKEQYLDEHPDMQEAKTMPPSTGTVKKLKVAEGTMPRHGNQVSSASPGVASTMTKLNSSPSRKAFSVQDKLWSIYAAGNTVPIPFLKAIDISPLALVSDSVVGNGLAGSNRTDALEAVRELFSGDGQTKKGRKGQNEVPLPPGVHHRLTTSPTLMNLAQALAYHKTCYEDMPLQDSQATQEQQTIQNLCDTLRTILRL*

>LOC_Os06g29120.1

MEHARRFPTDPKEYKLCEEVGDGVSATVYKALCIPLNIEVAIKVLDLEKCSNDLDGIRREVQTMSLIDHPNLLRAYCSFTNGHQLWVIMPYMAAGSALHIMKTSFPDGFEEPVIATLLREVLKALVYLHSQGHIHRDVKAGNILIDTNGAVKLGDFGVSACMFDTGNRQRARNTFVGTPCWMAPEVMQQLHGYDYKADIWSFGITALELAHGHAPFSKYPPMKVLLMTLQNAPPGLDYERDKRFSKSFKDLVATCLVKDPRKRPSSEKLLKHSFFKHARTAEFLARSILDGLPPLGERFRTLKGKEADLLLSNKLGSESKEQLSQKEYIRGISGWNFNLEDLKNAAALIDNTNGTCHLDGVNSKFKDGLQEANEPENIYQGRANLVASARPEDEIQEVEDLDGALASSFPSRPLEALKSCFDVCGDDDPPTATDLREQPNMESTSPMQQFQQIENHKSANCNGESLERSASVPSNLVNSGSHKFLSGSLIPEHVLSPYRNVGNDPARNECHQKNTCNRNRSGPLFRQMKDPRAHLPVEPEEQSEGKVIQRRGRFQVTSDSIAQKVASSASSSRCSNLPIGVTRSTVHPSTILPTLQFMIQQNTMQKEVISRLISSIEEISDAADASTTGSSQPSGVHFREKELQSYIANLQQSVTELAEEVQRLKLKNTQLEEQINALPKKDERLRREDTRQQ*

>LOC_Os10g37480.1

MGRNGSVKRTSSSGAAAAFTANPRDYQLMEEVGYGAHAVVYRALFVPRNDVVAVKCLDLDQLNNNIDEIQREAQIMSLIEHPNVIRAYCSFVVEHSLWVVMPFMTEGSCLHLMKIAYPDGFEEPVIGSILKETLKALEYLHRQGQIHRDVKAGNILVDNAGIVKLGDFGVSACMFDRGDRQRSRNTFVGTPCWMAPEVLQPGTGYNFKADIWSFGITALELAHGHAPFSKYPPMKVLLMTLQNAPPGLDYDRDRRFSKSFKEMVAMCLVKDQTKRPTAEKLLKHSFFKNAKPPELTMKGILTDLPPLWDRVKALQLKDAAQLALKKMPSSEQEALSMSEYQRGVSAWNFDVEDLKAQASLIRDDEPPEIKEDDDTARTIEVEKDSFSRNHLGKSSSTIENFFSGRTSTTAANSDGKGDFSFEAFDFGENNVDTKIMPNGYENARSENSSSPSTSKQDPESKYWRSTSGQKQQTSGTPAVHSGGVNSSTTEKGHGVERDATVQLASDKLRTETRRATNLSGPLSLPTRASANSLSAPIRSSGGYVDSLGDKSKRNVVEIKGRFSVTSENVDLAKVQEVPLSSLSRKSPQASPLKKSASVGDWLVNTKPMSNSHHVKELCNSSVSSSILIPHLENLVKQTMFQQDLIMNVLSSLQQNEKVDGVLSGISPQLRNTDSDTMVGSVNSEKERSLLVKISELQSRMITLTDELIAAKLKHVQLQQELNALYCREEIEDIRDDDNEET*

>LOC_Os03g02320.1

MVRSGSVRRTAASSSPAAAAVPTAFTASPGDYRLLEEVGYGANAVVYRAVFLPSNRTVAVKCLDLDRVNSNLDDIRKEAQTMSLIDHPNVIRAYCSFVVDHNLWVIMPFMSEGSCLHLMKVAYPDGFEEPVIASILKETLKALEYLHRQGHIHRDVKAGNILMDSPGIVKLGDFGVSACMFDRGDRQRSRNTFVGTPCWMAPEVLQPGAGYNFKADIWSFGITALELAHGHAPFSKYPPMKVLLMTLQNAPPGLDYDRDKRFSKSFKEMVAMCLVKDQTKRPTAEKLLKHSFFKNAKPPELTVKSILTDLPPLWDRVKALQLKDAAQLALKKMPSSEQEALSMSEYQRGVSAWNFDIEDLKAQASLIHDDDPPEIKEDVDNDRINEADKEPFSGNHFGQPKILSGKHFSEQTCVTAVSPGGNMHETSRGLVSEPGDADSERKVDGYRKQGSENESLPSTSKHDSEGQNSSSEVKQKERTCSGPILCSGVHNKSITESSRIFDREAAVKLASDKQKSCTKRTTNLSGPLALPTRASANSLSAPIRSSGGYVGSLGDKSKRSVVEIKGRFSVTSENVDLAKVQEVPTSGISRKLQEGSSLRKSASVGHWPVDAKPMSNSHQRKELCNGSVSASVLIPHLRNLVQQTTFQQDLITNLLSSLQQNEKADATQYRLGNMDGDTEVETSISEGERSLLVKIFELQSRMISLTDELITTKLQHVQLQEELKILYCHEEIIDTREVDNA*

>PAB00014937.1

MDIYEDEIRREAQTLSLTSHPNLLRAHCSFVANQNLWVVMPYMAGGSCLHIMKSVYPEGFEEVIIATILRDVLKALEYLHNHGHIHRDVKAGNILVDESGAVKLGDFGVSACMFDTGDRQRSRNTFVGTPCWMAPEVMEQLHGYDFKADIWSFGITALELAHGHAPFSKYPPMKVLLMTLQNAPPGLDYERDKRFSKSFKEMIAMCLVKDPTKRPSAERLLKHSFFKHARSNEYVARTLLDGLPPLGDRLRALKLKEEDRLAQKKMPYGEKEERSQNEYKRGISAWNFDLEDLKSQAALIKDYDELPAAKEDLEPQKSTKGSEDSKDTPSYFQSPINIGSLRVETLEKDGDHVVRVQPGPKPYFPLQCLNGLKGRFDVFEDDIHCESPTWRECTKSEFCRAQETQIYKGNAENYRNDERKQSFQEDSNPAPVHSHKVCSGPLAPEHILACHRLRTGDASREKLQSRPQRERNFSGPLTAHVASDIRSTDSFLGSPKSSAYNLEEKSKGLVVQQKGRFKVTSGDVIPSSSPRRTQGLQVSILKYI*

>PAB00026846.1

MCVLWNQFEFMATGKSIPKILHHSHFSLLKARPRKVQDVSLESHRCYLVEHEKYQILQEIGRGGSGTAYKALCLEFNELVAIKCIDIEISKPNMENLEKESRTLMLLHHPNMLGGHCSFVAGQFLWEVIPYMAEGSLYNILRSSTHEGLEESVVATVLKETLKALDYLHKAGHIHWDVKSSNILIDSDRSIKMAEYGVSACIYEFGDRDKMMRHTLTGSLLWMAPEVILCETTRVYDCKDYIWSLGITAMELAQGHSPYSGSQPMKVIMNLAKGTVPKITDAKFSKSFQDMVASCLEYDPAKRPSTEKLLKHPFIKKNSHSPHHLAKHLLRGLPELPQHLKIVGEEECHLLSVKMQYLTPKIEKEKQRIEWDFM*

>PAB00040610.1

MDHHHQKSYSLISSEYRMVDEIGHGVSATVYEAKCLVLDETVTIKSIDLELLNATLDDVRREAQMMSLVDHPNVLRAHCSFIVQQSLWVVMPYMAGGSCQSIMKKSFPNGFEELVIALILKETLKALEYLHQQGHLHRDVKAGNILIDSRGGVKVGDFGVSACVFDSRDGQRLKQNTFVGTPCWMAPEVIDHGNGLITRPIYGRLGLLHSSSRMAMPRSLSSRQSKPSSCSSRAPRQVWTMKPIRSFLEVKDTQLYYESNFVPEPKNTQIDYESNCVLKPKNTQVASEANSIPSPENTQVVSQTNCVLDPKNTQVDSQSNCVPGPKNSQVASEAYCMPEPESIIEFGHKPQSDAESNTMPRH*

>PAB00040641.1

MGNFMQDDIRREAQTMSLIDHPNVIRAYCSFVVDRCLWVVMPFMAEGSCLHIMKIAYPDGFEEYAIATILKETLKALEYLHRQGHIHRDVKAGNILVDTSGAVKLGDFGVSACMFDTGDRQRSRNTFVGTPCWMAPEVLQPGSGYDFKADIWSLGITALELAHGHAPFSKYPPMKVKSTSTHPMSENQFVCIPRLLKFSSYNVMIFLIVSFISSGCILLTSIFNI*

>Pp3c11_21850V3.1

MSSSDPRGSPGLIRSESRRREQLGVDNLDSSKSPSSPPSIFATVITKPDEVSSSSQFSTVIQHISGTDPYSTVVHKAAESFRKDGGGSVQRRDGGGRPRGKSDWAGGIEGSSGALSGDVRDRTSLLQENVAFEDPSLKYELLNELGKGSYGAVYKARDLLTSELVAVKVISLTEGEEGYEEIRGEIGMLQQCNHPNVVRYLGSFQGEDYLWIVMEYCGGGSVADLMNITDEALEEQQIAYICREALKGLTYLHSIFKVHRDIKGGNILLTEQGEVKLGDFGVAAQLTRTMSKRNTFIGTPHWMAPEVIQENRYDGKVDVWALGVSAIEMAEGLPPRSNVHPMRVLFMISREPAPMLEDKERWSLVFHDYVAKCLTKEPRLRPTASALLSHKFIEKCKGTASSMLPQIEKARRIRAEMAAQAANYVQEQGTTSSGGQYGSWEKGQTVKMHDTSAGTMLVRDTVVKSLSDTFKEGTGTIRGPVSAELDSRRSMYEATMSMETSPNREDSKSSLDTRDQDTDQGRNSTPESTVGSRKHFKDGVRLTLETPTTYSSSYAVFSPIPHQPVGVHSSISRRSLSSMEATLGPGTPVGGGNRPGFGIQDKLLSVYAAGNTVPIPFLKATDLSPIALTFDRIFADGRPDRTRNVALEAIQDLYVGEGHVRRGRKAPNSEMPLPPSVYQRTATSTTLPNLARSLAYHKRCYEELPSQGWQAAQEQQIIHNLSDTLRTILRL*

>Pp3c12_5150V3.1

MEGQGKEQKNGVGEEGGDEEGRRGYPLSASDYRIMEELGRGASCTVYSALCLPYNELVAIKVLQLDEGTRISSDFWTASKSRIQHPNVLTAYCSFTTSHNVWVVMPFMAGGSCLDIMKAAFSNGFDESVVCTFLKESLMGLNYLHHHGRIHQDVKAGNIFVDGNGRVKLADFGISTCILNAGDQQQSRSSLLRSCYWMAPEMIEQVHLYDSKADIWSFGITALELVHGIVPFSKYPMKVFFNTLQNKISEMNCACFKMFSKSFKDMIAICLAKDPSRRPTTEQLLRHPFFKQARSPEYIYQHVLDGLSSLGERSKNLKIIEAAQLALKKSPHSKEKLSQGEYLRGLHNWICNAHDIRKTETEVAIFKGGPADLAAQMDVDWESSCLHSKSASSIEIMDIIAERDAALMEKSTALAEKMAAYAERDAAILQRDIASADRDAAILTRDAALAALARLERKSSGRGRRPSQTALDESMHGSKLLQRMDLAEHSAFSSEFHPTAANPSHSGIMLLDPDSAHLQGVHRFSRDKESQIEIFVRTKRKATEMAEATHRGKHPRAAPKKSRNWPVTHQGQQPVQERESQAQVANEEEGGQIREPELVSSRIFRSQTVPTPIPYCSCTGMNQQCYRWGNGGWQSACCTTLISMFPLPLNPKKRGSRLAGRKMSAGAFDKLLEKLVSEGVNINLPVDLREHWAKHGTNRYVTLR*

>Pp3c17_14200V3.1

MEHDEDVDDEIHGAVDSAMEARFENLRLIGKGSFGDVFSGFDKELNKEVAIKVIDLEEAEDEVEDIQKEISVLSQCRSPHITEYYGSYLHATKLWIVMEYMAGGSVLDLLETGPPLDEVSIACILRELLLSLDYLHSEGKIHRDIKAANILLTANGDVKVADFGVSAQLTRTMSKRKTFVGTPFWMAPEVIQNSGDGYDEKADIWSLGITAIEMAKGEPPYADLHPMRVLFLIPKNNPPQLDDHYSRPFKEFVSFCLKKNPAERPSAKELLRHRFVKNARKSTRLLDRIRERPKSHISKSKDVPRPAEAFENESSSKEMGAKGDNGVYPPLSSRRKPLRDASWDFGTGTIHTTGTLRSGSTNLYTDQDRTGGNENTVLSGSEALPSLLSKAELDYQIDAGLRDASMDGNGFRRNSFGIQRKLDTEQYSPSDYGSYASASGTVKAASTLERPSLKSLSTSLANTGGNRNEEASEIFASGTIVRSSTPDEGGSVPGTPKAKQEPREDSATNLAEAKAALQAGMRKGGPRGPAPVKLRKEALEYASDPRETPRETPKATPRASARASEEDVARAAAAGASAALALLLIPALKETAAEQSEGPALRAAADAADALMDLERLAPGACEVLVSKLLRQLARKDQAPVKGLQNLARRLLSSSDNGSEVGHIPSSRDQPGNVRHRFQRDKTDDSGLSPVASFLLHRWQNQVAKDLNARR*

>Pp3c1_23810V3.1

MEYYVYVDEEVHGLIDGAMEARFENLELIGKGSFGDVFRGFDKELNKEVAIKVIDLEEAEDEVEDIQKEISVLGQCRSPHITEYYGSYLHTTKLWIVMEYMAGGSVSDLLETGPPLDEVSIASILRELLLSLDYLHSEGKIHRDIKAANILLTANGDVKVADFGVSAQLTRTMSKRKTFVGTPFWMAPEVIQNSGDGYNEKADIWSLGITAIEMAKGEPPYADLHPMRVLFLIPKNNPPQLDDHYSRPFKEFVSFCLKKNPSERPSAKELLRHRFVKNARKSSRLVDRIRERPKSHVSKAKDTPRPSEAFKNESDSKGVRMRSDNGVYPPLSSRRRDASWDFGTGSIHTTGTLRSSSKTFGQIDQDRPRGTDNSLHQGSDVLPTLLSKAELDYQIDAGLRDASVDINALRRNSFGTQKRLDAEPYFSRDPGPHASGDSSMEGRSQARPFLGSLSATTVNTTGGSRNEEASEGFSSGTIVRSSTPDEGGSIPGTPKINLEAREDSATNLAEAKAALQAGMRKGGPKGPTPVKLRKDAHEHLSDPRETPKAASRQSRRASEEDVARAAAAGASAALTLLLIPALKETAAEQSEGPALRAAADAADALMDLERLAPGACEVLVSKLLRQLIRKDEAPVKGLQSLARRLLSDNGGEKDGHVPSSHIRSGTARLRSQRDKSYDSGLSPVAAFLLHRWQGQVSKDLNFRR*

>Pp3c11_1950V3.1

MPSGGGTMVEAEKRSYPVLASEYKLYEEIGQGVSAIVYRAHCVTYNEIVAIKSLDLEKCNSNLDNIRREAQTMSLINHQNVVKAHCSFVVGQNLWVVMPHLAGGSCLHIMKAAYPDGFEEPVIATILKESLKALEYLHRHGHIHRDIKAGNILVDSNGSVKLGDFGVSACMFDTGDRQRSRNTFAGTPCWMAPEVMEQLHGYDFKADIWSFGITALELAHGHAPFSKYPPLKVLLMTLQNAPPGLDYERDKKFSKSFKEMIAMCLVKDPAKRPTAEKLLRHSFFKQARSFDYIARHILEGLPPLGETVNNLKIKDANRLAQQIQPYDEQEAQSQNEYKRGVSAWDFNVEDLKAQAALIQDDEEVVPKLLKVALNTEAEEISQAPVTPVKEEITEGDLQCSSPLSQPPLCRESSFNRVQMPVTIHSIIAETTNDAGERVTRIRSGPLPNPVQPKSANISRTALPSGRKEPKHIGRFDVWDEHDGESLSWHGSPRENRKSEARRERDDREQRRGDDRDRKEEREQRRSDERSERDEIRRVGSERVLSSIRGLTTMDERERDRDRDYRQGGRGSDYSGRMMQRERSFSGPVNTISDRSSFDQKSTNGLQAVSRQTPTQVNLNKEISEDKVKAPVQKGPVLHKGRFSVTSDDTQFEESSQLNPRKTSNTQASLHKSASSGDFMSGERRLPVSQHSSSSQHALLSQHSHSSLHVSSPNGLISHQNSQVTTPTAAVLSQLQQIFQQAQKQQDSILHLIQSLNPQDAASVISTSSMSRFSRTSSTNSGVEHSRNSVGARLRVNQVEVSTDRERELLHQVSDLQFSLRGS*

>Pp3c21_3370V3.1

MGGGVASEGQMEKRKYPYGASDYKLFEEVGQGVTATVYRAHCIPYNETVAIKSLDLEKCNTNLDDIRREAQTMSLIDHPNVVKAYCSFVVEQSLWVVMPFMAGGSFLHIMKSAFPDGFDEPVIATVLKETLKALEYLHRHGHIHRDVKAGNILVDAAGAVKLGDFGVSACLFDTGDRQRTRNTFVGTPCWMAPEVMEQLHGYDFKADIWSFGITALELAHGHAPFSKYPPIKVLLMTLQNAPPGLDHERDKKFSKSFKEMIAMCLVKDPAKRPSADKLLRHSFFKQARSGDYIARNVLYGLPPLGERVKKLKVNDANRLAQNEQEVQSQNEYKRGVSGWNFDLDDLKAEAALIQDGDEDLSMVVGKDVIVVEEQEVTKLQVLPFMETDGDVPSHCLTPSPPPSLSRQPSIDRFQQPVTIHSIIAETTSDAGERVTRVRSGPLPNPVQTKPTNGMRGRFEVWEDNNPESPVSHGSPRESRRSAEDKEPSRRERDSREFRKADDRERKEDREQRRSDERGDRDAFRRVGSFGSERVLSSIRGLTMDSREQDRDRDIRQNGRNGSKETLEEKTKYLGQKGSVLQKGRFSVTSDDMNLEDSPHVSSRKYSNVPTPLHKSASASEWSNDRKVLGLQNPASPSQQSIASSQNISGAISLCAGIMPQLHQILQHGQRQQEGILNLIQSLSPTDAANLQALNSASRYLRSLSANSVDSSSGCGSPRSPQCGSRNRPSQVEASHKGYYNCQLEVSTDKERELLHQVSELQARMANLVDELQTVKLRNVSLERQLNAIYNKEEEERIRKEDAARESG*

>Pp3c4_10780V3.1

MEKQSERKGPYPVTATQYQVLEEVGHGLGATVHRAICLPFNEVVAIKKLDLESRNVNVDDIRREAVTMSLTNHPNLVKSYCSFVVDQSVWIVMPFMAGGSCLHIMKAAFPDGFEEPVIATLLKESLKALEYLHRQGHIHRDVKAGNILLDGDGSVKLGDFGVAASMFDKGDRQRSRITVKGTPCWMAPEVIEKTHGYDFKADIWSFGITALELAHGHAPFSKYPPLKVLLMTLQNAPPRLDNERDKKFSKSFKEMISMCLVKEPTKRPSAERLLRHSFFKQARSSDYILRHVLDGLPPLGERVKHLRMLDAAQIAEKKMPFEEQEEKSKTEYKRGVSNWNFNIEDLKAEAALISGDGGYAEAVKEEDDTPKQQTQKGLDDYLPPKNAEPSARDRELRQRGMSAEDLSRCRERAFSGLLPSDERATSDQGPASGFQGVPRPSSGSKEGSEEKSKGPAQKFPIVQRGRFSVTSNDLDLQDPPQGSTRRRSASSQALQLQAPSVASSLSNVSGSSVSLAALLPHLQNALNHAVMQQDTLMNLLNSINASEASSSLRRSSRSSSSHSMTEISSDREKELLHQIAEFQSKISVLVDELQALKLKNMSLERQLNAHHNREKEERIRKQTNIGDG*

>Pp3c4_15760V3.1

MEGGQSERRIYPVTAAEYKLLEEIGQGVSATVYRAICLPFKEVVAIKALDLEKCNSNLDDVRREAVTMRLINHPNVVKAFCSFVVEQTLWVVMPYMAGGSCLHIMKSAFIDGFEEPVIATFLKEVLKALDYLHRHGHIHRDVKAGNILVDEKGAVKLADFGVSACMFDTGDRQRSRKTFVGTPCWMAPEVMEQLHGYDFKADIWSFGITALELAHGHAPFSKYPPMKVLLMTLQNAPPGLDYERDKRFSKSFKEMIAMCLVKEPSKRPTAEKLLRHSFFKHARTPDYICRHILDGLPSLGERIKNLKNADAARMTQQKNLDEQEQRSQTEYNRGVSAWNFNLEDLKAQSALIPDDSDDSPPQSTKAESPEPSHFHSPQSRHGDFGSSPDRVQPSISMHSSQVEATSDNGEFLAAPRNSYSSKSAVRSAGVSVRKEPKHIGRFDVFEDDNDMESPGWHYSPSRRGFERDQTRKEREDTGSYEDRGRDDSRRGGSGPLVPERVLCGGRGRDDERDREHRHGRVSERGSFSGPLLPTGDHRLSNGVSRPPTVSKDALEEKLKGPTLKAPIVQKGRFSVTSNDGGVEDLPHVSSRRSTCSQGSQQHLFASASSSHLNGLSVPAAAIVPHLQTALQLAVMQQDALVNILSGINPSDSSNSMKLHYASKRNSSFNCGSECSMIEILNDRERDYLQQISDLQSKVSQLLQEQQVMKMKNITLERQLNAIYNREEEERIRREEAAEGDGLPL*

>Pp3c7_24190V3.1

MPTGGGTLPEVEKRTYPLVASEYKLYEEVGQGVSAIVYRAHCVTYNEIVAIKSLDLEKCNSNLDDIRREAQTMSLINHQNVVKAYCSFVVGQNLWVVMPYMAGGSCLHIMKAAYPDGFDEPVIATVLKESLKALEYLHRQGHIHRDVKAGNILVDSNGSVKLGDFGVSACMFDTGDRQRSRNTFVGTPCWMAPEVMEQLNGYDFKADIWSFGITALELAHGHAPFSKYPPMKVLLMTLQNAPPGLDYERDKKFSKSFKEMIAMCLVKDPAKRPTAEKLLRHSFFKQARSLDYISRHILEGLPPLGERVKNLKIKDANRLAQKIQPYDEQEAQSQNEYKRGVSAWNFNVEDLKAQAALIQDDEEIVVAKVLKIAVDKEVEEISQAPVTPSKEEITEADLHCLSPLAQPPLCHESSSDRIQLPVTIHSIIAETTNDAGERVTRIRSGPLPNPGQSKSANISRTAPLSSRKEPKHIGRFDVWDDHDGESPSWHGSPRESRKSDETRKEREDREQRRGDDKDRKEERDPRRPDERSEKDEIRRVGSERILSSIRGLTTLDERDRDSRDKDHRQGGRSGEDSSRVVQRERPFTGSGNIISDRSNFDQKAINGSQTSSMSRQSCGTKEVSDEKVKAPVQKGPVLQKGRFSVTSDDTDFEESPHLSSRKSSNTQASLPKSASTGDFMSAERRLPHTPVLQHSPSSQHISSTSGQASQQNSHVTTPIATILPQLQQILHHGQKQQDSLLHLIQSLSPQDAASVCSVSSMSRFSRTSSTNSAIEHSMEVSTDRERELLHQVSELQSRIATLVDELQAVKLRNVQLERQLNAIYNKEEEERIRKEEAAKGDR*

>Potri.001G084700.1

MGRMGKMYTVNPNDYKLLEEVGYGASAVVYRAIYIPFNEVVAIKCLDLDRCNSNLDDIRREAQTMSLIDHPNVIRAYCSFVVDQNLWVVMPFMAEGSCLHLMKIAYQEGFEESAIGSILKETLKALEYLHRQGHIHRDVKAGNILLDTNGIVKLADFGVSACMFDTGDRQRSRNTFVGTPCWMAPEVLQPGSGYNSKADIWSFGITALELAHGHAPFSKYPPMKVLLMTIQNAPPGLDYDRDKKFSKSFKEMVAMCLVKDQTKRPTAEKLIKHSFFKNAKPPELSVKKLFADLPPLWNRVKAIQLKDAAQLALKKMPSAEQEALSQSEYQRGVSAWNFDLEDLKAQASLVRDDDDIPETREEDESIKFGGGKAAIGSQSSSVKVNSNSEIQLVEYSRQLSGGELPQADNFIRKGKLPESDLLETSSLEKVGWKRNGSSSEAKASTSESVMAQAKAKTVKSRQTHSGPLMPGTVFSHSLSERGRTSERFENEIHPTAERATREVRKAPSFSGPLMLPNRASANSLSAPIKSSGGFRDSLDEKSKTNLVQIKGRFSVTSENLDLVKPVNQPPKEVSNNSVHALLFPHLQNLFQQTSIQQDIIMSLLNSLQPAEAIEAAQNGKLPPLPHGSENNGSVEAASSEREKSLLIKITELQNRMVNLTDELNAEKLKYEQLQQQLKAISGREENGERSEVDA*

>Potri.003G146000.1

MGRMGKTYTVNPIDYKLLEEVGYGASAVVYRAIYIPFNEVVAIKCLDLDRCNINLDDIRREAQTMSLIDHPNLIRAYCSFVVDHNLWVVMPFMAEGSCLHLMKIAYSDGFEEPAIGSILKETLKALEYLHQQGHIHRDVKAGNILLDTNGDVKLADFGVSACMFDAGDRQRSRNTFVGTPCWMAPEVLQPGSGYNSKADIWSFGITALELAHGHAPFSKYPPMKVLLMTIQNAPPGLDYDRDKKFSKSFKEMVAMCLVKDQTKRPTAEKLLKHSFFKHVKPPELSVKKLFADLPPLWNRVKAIQLKDAAQLALKKMPSAEQEALSQSEYHRGVSAWNFDLDDLKAQASLVQDDDDDILETREEDESVKFGGDKVTTGSQSSSLNVNSKSEMQQAEYSRQLSGGKLPRVDNLNRKGRLPESDLLETSSQEKVGWKRNGSSTEAKASTSENDVMQAKAKTVKSRQTLSGPLMPGTVLSHSLSERGRTSERFENERQPTAERATREIRKAPSFSGPLILPNRASANSFSAPIKSSGGFRDSLDEKPKTNLVQIKGRFSVTSENLDLVKDIPLSTVPRRSSQSPLKKSASVGEWMFEPKQMPVNNQPPKEVNNNSVPALLLPHLQNLFQQTSIQQDIIMNLLNSLQPAEAVEAAQNGKLPPLPRGSENNGSVESASSERERSLLIKITELQNRMMNLTNELNSEKLKYEQLQQQLKAITGGEENGERSDVDS*

>Potri.004G194700.1

MSASSSSSRADGKVQIQYPTGADAYRILEQIGGGARATVHKATCVNNDIRYSGLVSIKIIDLEQYSAADLDGLRRESKAMSLHSHPNFLGSLCSFTVDHHLWLVMPYMAAGSLQSIVSSFFPDGLLEPCIAIVLKETLKGLSYLHRLDYLHTDIKAGNILLNHTDNGSIKLEDSGMSVWIYDSNSIEKSSSLSSSSKMRLTDVAGTPYWMAPEVIQDSNTGYSFKSDIWSFGVTALELAHGGPPFSYLPPSKSLMLKIKKRFGLSDYDYDEKSKKDFKNNHFSQAFKDMVASCLDQDPSRRPSADQLLQYSLFKNCEGLDLLFNEFFRGLPNVEERFKEPNASSDGTSSQITSGTDTDSAGSSVKTTRISGWKFNESKFELEPEFHAESKDDVVKTVHFGGETIIDTDTNIGFSESSSGSGDLEGLVGDHAGANMSGIEGAVETLNQETVAEKASSRYDCSVTTRV*

>Potri.008G163800.1

MAHFQDQRTKSQRVQYPVDSNAYKILDEIGVGVSATVYKAICVPMNSTLVAIKCIDLDQSRADFDSVRRETKTMSLLSHPNILESHCSFTVDRHLWMVMPFMSAGSLQSIISSSFPDGLPEPCIAVVLKEILNALSYLHDQGHLHRDVKAGNIVIDSNGKVKLADFGVSASIYEFNTLERSSSLSCSSRMRLTDLAGTPYWMAPEVIHSHTGYSFKADIWSFGITALELAHGRPPLSHLPPSKSLIMKITKRFRFSDCHEENRKKSCRNKKFSKAFKDMVASCLDQDPSKRPSAAKLLKHSFFKNCKGLDYFVKNVLHGLPSVEERFKEAKVLSGISSQSGTDVEEEEKGDIDGDSVIQRVKTRRISGWNFNEEGFELDPVFPTDSKNDSVVKQVRFGGESIIQDKKIEFSESDGSGDLVDSAKPSILNSLAPVKEEMSQVGDHIGVNMSGVGGIVEGLNQVTMLEGLVALKRSLEEQRRHVAIIIGLLGGETDGEDQMVQMSENLKEELDIEKQKNLKLEMELEFIKIVISGAFAAASFPN*

>Potri.010G157400.1

MWRMDHPSPSRRTRTSKTPNKSELYSTVVIHNSDSDSEPESKSKTDDNNIYATMLYKGGGENNSKDDDVDVEEEDEESLPPLLKRLPKDFGGGDDDDDADFGTMIVKASRGRHQNQSWSSSSSVAPPRKPYSAPFTEFESRINDIGDNSDGDDDGRGEFGTFLVKSTVVRRSGSGGGGSTMGKAVASMQASGELGFGKERKGSGLLGEEGKQHQQKQSKMSSSSIPESVTREDPTTKYELLNELGKGSYGAVYKARDLRSSELVAIKVISLTEGEEGYEEIRGEIEMLQQCSHPNVVRYLGSYQGEEYLWIVMEYCGGGSVSDLMNVAEEPLEEYQIAYICREALKGLAYLHSIFKVHRDIKGGNILLTEQGEVKLGDFGVAAQLTRTMSKRNTFIGTPHWMAPEVIQESRYDGKVDVWALGVSAIEMAEGLPPRSTVHPMRVLFMISIEPAPMLEDKEKWSLVFHDFVAKCLTKEPRSRPMASEMLKHKFIDRCKVGASAMLPKIEKARQIRTAMSLQAQNLAPAESEPTEGPQLNEVYGDTVPSNRLPMVNEVHSSSDGVDMAGGDYGTFVVHGGEETDKTGLQTALYDVGGILQDHPGNIEGLSVSGTGGKSADPWLDNATGVAANNPLVGESLPALQTIQTSTPEVSGYSEQNLKKNTVSKVHVEGGGGLGSSTLKNETVSRKAFALQDKLWSIYAAGNTVPIPFLRATDISPIALLSDNVLGGIQCDNSGTVAAEALQELFSGDGPSKKGRRIQNEMPLPPGVYQRLTSSSTLLNLAQALAYHKMCYEEMPLQELQATQEKQTIQNLCDTLRTILRL*

>Potri.016G049500.1

MADELDQESEAQLQYPTDPNAYRLVDEIGGTGARAKVHKAICIHNIWKSTFVAIRIFDLEQYPADFDGLRRETKTMSLHSHPNVLASHLSFAVDSYLWVVMPYMAAGSLQPIISTYFPEGLPEPSIAIILKETLQGLCYIHDQGRLHTDIKAGNILIDTENGSIKLADRGKSVSIYDLRSVVGSSPLSPSSRMRLTDVAGTPYWMAPEVIHDLDAGYSLKADIWSFGITALEIAYGGPPFSDLPPSKSLIMKIKKRLGFSNYHDEKHKKDFKNKKFSKEFKDMVASCLDQDPSKRPSADQLLEYSFFKNCRGLEFLFKKVFDGLPNVEETFKELKALQGTPSQITAGTDVEEEEERPESVGPSEKTRWISGWKLNEYEFNLVPEFSSDSEDDSVVKLVRFGGETIIPDTNIGFSVSSIGSSDLEGSVEDHTGENMSGIEGIVEEFNQVTVLEGIMALMRSLDEQRRQVARMIALLGGEADGEEQLVQRIENLMRELDLEKEKNLKLEMELENINIVISGAYNDASAAAAADDDDAAIDDID*

>Potri.001G037900.2

MEKKKYPIGSENYLLYEEVGQGVSASVHRALCVPFDEIVAIKILDFERDNADLSNISREVQTMILVDHPNVLKSQCSFVSDHNLWVVMPFMAGGSCLHILKAAYPDGFEELVIATILREVLKGIEYLHQQGHIHRDVKAGNILVDGRGAVKLGDFGVSACLFDSGDRQRMRNTFVGTPCWMAPEVMEQLHGYDFKADIWSFGITALELAHGHAPFSKYPPMKVLLMTLQNAPPGLDLERDKKFSKSFKQMIASCLVKDPLKRPSANKLLKHSFFKQARSNDYIVRTLLDGLPDLGDRIKDLKRKEEDMLAQKKMPDGEMEELSQNEYKRGISGWNFNLEDVKAQASLIPDAEDHTTDSNPGGSSNSLSTLDAVEKQSEPRNSSLGQVTEMMEDKDMQNRAAPLPSVNSAINITKVRSVKSDDDSINASPCHERHVSQNSSSLSDRVEGNATERPASDINGKPSDKLQNQPPNNSNINGAIINQDGDDVPSENPSKPFKSSGASSEELDEKAKPPVVQQRGRFKVTSENVDIEKAVSPLVLQKSHSMQVRNFEVLTQHPGTPSPSPSETIPSTVLDHSVFPLLLSLLQTNITQRDGILHLMRQLYGGDTAGNRTTDGGWATAQGGSTEKSLIEAAHDREKELLHEITELQWRTNSKNLTSYTGEAHRGDSP*

>Potri.001G349300.1

MEYVSEKRYPVNAKDYKLYEEIGEGVSATVYRALCIPFNQIVAIKVLDLEKCNNDLDGIRREVQTMSLIDHPNVLRAHGSFTAGYSLWVVMPYMAGGSCLHIMKSAYPEGFEEPVIATLLRETLKALVYIHEHGHIHRDVKAGNILIDSDGTVKLADFGVSACMFDTGDRQRSRNTFVGTPCWMAPEVMQQLHGYDFKADIWSFGITALELAHGHAPFSKYPPMKVLLMTLQNAPPGLDYERDKRFSKSFKEMVAACLVKDPKKRPTSEKLLKHHFFKNARSHDYLVRAILDGLSPLGERFKILKAKEADLLVQNKALYGDKEQISQQEYIRGISAWNFNLEDLKNQAALIQDYDCMSNAEDPDLSGKQMDRYNIVGFPAEKLPPKIANHSISAPSQEDGFNDLHDLETSLPSFPIKPLQALKGCFDVGEEAVGATSPNWKVTSQTECEQQVLTELSSSAMDQESERNEDNALSPKKVIGDENRDLLQPKYQSERNYSGPMLHRQKRDTNNLSSVEDTSEGAVVQRKGRFKVTSADLSPKGPTNCCFNPVGGGSACATISNPAASSVLPSLQCILQQNTLQREEILKLIKYVEQTSGKLVESGEAATNDLLQISPTWTREKELQAQFIGLQQSVGSLFEELQRQKMKNVQLERQLNAFINKERERS*

>Potri.001G397600.2

MADAAGLMEAAGARFSSLELIGRGSFGDVYKAFDKELDKEVAIKVIDLEESEDEIEDIQKEISVLRQCRSPYITEYYGSYLHQTKLWIIMEYMAGGSVADLLQSGPPLDEMSIACILRDLLHAIEYLHNEGKIHRDIKAANILLSENGDVKVADFGVSAQLTGTVSRRKTFVGTPFWMAPEVIQNSEGYNVKADIWSLGITAIEMAKGEPPLADLHPMRVLFIIPRENPPQLDEHFSRPMKEFVSFCLKKVPAERPTAKELLRHRFIRNARKSPRLLERIRERPMYQIKDAETPRNGPIGIGEGFDTVKVVRDLRADGTVRASGQGKPFKNAGWDFSIGGSQTTGTIRSAARPPQESLELSYGKDARDPYHDDHQDNSYDDDDLSVSGSGTVVIRTPKGYQSSALFRDQNNASSSTSTSFEDASTSGTVVFRGQHDESDSPRTHKSRLGMQERTSSSSLEDSALNLAEARAALQGGLRKVNARERFVPSNNNRYGLENRRREQLTNSSDSSRSSREYFDAPKAFPRSQQASNVEESARIASASLSVLLIPSLKEAVADDSERALFHAVTNSLVNMERVKPGSCDIFVRSLLQQLASSKESSLRDLQELAAHLLSKGKTTPEETQNGNTDVDSRKKQPTKEFNSNANLSPLLIQMPI*

>Potri.003G186700.1

MHNPLSFLFTPRHHHHHHREGKNKDLRKTMEKKKYPIGPENYVLYEEIGQGATASVHRALCVPFNEIVAIKILDFERDNADLRTISREVQTMILVDHPNVLKSHCSFVSDHNLWVVMPYMSGGSCLHILKAAYPDGFEELVIATILREVLKGIDYLHHQGYIHRDVKAGNILVDGRGAVKLGDFGVSACLFDSGDRQRTRNTFVGTPCWMAPEVMEQLNGYDFKADIWSFGITALELAHGHAPFSKYPPMKVLLMTLQNAPPGLDLERDKKFSKSFKQMIASCLVKDPSKRPSANKLLKHSFFKQARSNDYIVRKLLDGLPDLGDRIRALKRKEEDMLAQQKMPDGEKEELSQNEYKRGLSDWNFNLEDVKAQASLIPDSEDPMTDNNLGESLNSLPTLDGVEKQSDTQNSSLGQVTEMMDDNDVSQNRAARRLPLVDSSLNIAKVRFVKSDDDSSTASPCHERHVSQNSSPGCDSLEGNAAERPAFNINGKPRFLVYIYIVKSCKINHQIVQISMEQQSFKMEMMELLKILPKHYINHQEQAVMNLMKKQSRQLFSKEDDSKLLQKTLI*

>Potri.011G116300.2

MADAAGLMEAAGARFSSLELIGRGSFGDVYKAFDKELNKEVAIKVIDLEESEDEIEDIQKEISVLQQCRSPYITEYYGSYLHQTKLWIIMEYMAGGSVADLLQSGPPLDEMSIACILRDLLHAIEYLHNEQKIHRDIKAANILLSENGDVKVADFGVSAQLTSTISRRKTFVGTPFWMAPEVIQNSEGYDAKADIWSLGITAIEMAKGEPPLADLHPMRVLFIIPRDNPPQLDEHFSRPMKDFVSSCLKKVPDERPNAKDLLKHRFIRNARKSPRLLERIRERPKYQIKDAETPSNGRKGIGEGSDTVKVARDIKPDGTVRISGQGKPFKNAGWDFSIGGSQATGTVRSAARPPQESLDLSFGKDARDSYHDDRQNNSLDDDDLSVSGSGTVVIHTPKGSQSSAIFRDQSNASSSSFASFEDASTSGTVVFYGQHDESDSPRTPKSRLGMQERTSRASLEDSALNLAEARAAFQGGLRKGNARERFVPNKNNSDGLESRRRETLTNSSDSSRSSREYSAVPKAFSRSRQASDDEESARIASSSAPLSVLLIPSLKEAVADDSEGSVVHAVTNSLVTMERLKPGSCDVLVRSLLQRLASSKESSLKDLQELAARLLSKGKPASEETQNANTEADNRKKQPTKEFNSNANLSPLARFLLSRWQGQSS*

>Sobic.001G085700.1

MAFSPRSPWSRPKRNDVYSTFVVHGDDDEDDARGSRRGRGPSTRAEEDEEEDPSSLPPLLQRLPKDFGGPSFDEDEDAYSSDPDDASLSATVVFKSGAPASARSPFLDLRRSSPRATDDDPYSTFVVHSTARSGGTSSSPRGSASGTFIRRSGGSSSPHESVSGTFIRRTSTPSSPRDSVSGTFIRRPGSPSSPRGSFSGTFIRHTSGGSSSYESASGGGGGFGSSFWSPAVEQTEELRQPSPLMQQQQQQHSRRKPSVSSAPDSVTREDPSTKYELLHELGKGSYGAVYKARDLRTQELVAVKIISLTEGEEGYEDIRGEIEMLQQCSHPNVVRYFGSYQGEDYLWIVMEYCGGGSVADLIGITEEPLDESQIAYICREALKGLAYLHSIFKVHRDIKGGNILLTEQGEVKLGDFGVAAQLTRTMSKRNTFIGTPHWMAPEVIQESRYDGKVDVWALGVSAIEMAEGMPPRSTVHPMRVIFMISSEPAPMLEDKEKWSLLFHDFIAKCLTKDPRLRPAASEMVKHKFIEKCNSGASKMLAKIKEAKKIRATLAVQNELSDPDNTMQDVPVRINEDYGETVPTNSQQHMNHGTYNDCQAGDFGTMIVHTEDGDEVAESPIFPRTEFIPGLGSINSFTHDPKRAELISSFWAENTGDSDANKDRDLDDRPDIQESKAIPPLTGTVKKHIGAEGTIRRHDNQVNLSPGFANTTKLNSSPSRKAFSVQDKLWSIYAAGNTVPIPFLKAIDISPLALVSENEAGNGLAGSSTNDALEAVRELFSGDGQAKKGRKGQNEVPLPPGVHHRLMTSPTLMNLAQALAYHKTCYEDMPLQDSQATQEQQTIQNLCDTLRTILRL*

>Sobic.002G313100.1

MSDSASMAAAIEARFSSRDLIGRGNFGDVYKGFDKELNKEVAIKVIDLEEAEDDIEDIQKEISVLSQCRCPYITDYYGSYLHQTKLWIVMEYMAGGSVADLLQAGPPLDEMSISCILRDLLHAIEYLHSEGKIHRDIKAANILLTESGDVKVADFGVSAQLTKTMSRRKTFVGTPFWMAPEVIQNSEGYNEKADIWSLGITAIEMAKGEPPLADIHPMRVLFMIPRENPPQLDEHFSKPMKEFVSLCLKKNPAERPSAKELLKHRFIKNARKTPKLLERIRERPRFAGKSMDDTQNGQTHDEEEDFGTGTIKVNRTKDTAPSLSQGTVRKATVRDFPDRSEGTGTVRVVSRPPQIASTKDGRSDMPQSPKAPIRTTDRENQRKSSWTGSEDSLSQRDTQSERGRVESSTDDNDQSVSGSGTVVLRSPRASQLYSTASNHSTKPPSRFSSYEDMSNSGTVVRTQNEDPETPRSSRSRLGMQEKAANTSLEDSAINLAEAKAALQAGLRKGNARERPIISRHEKGSHEHRTSGVNSQEVQSEDVDTQKGRKLRQLPDGQSASRASSSVAPPAVSSLLLPSLKEATGDKFDRPAVHAFLDSLMDLEHEIPGSCEVLVGRLLHRLGSSKDSSLQGLQETAMSIFTKKPESPSEKAGDKKLANMPPLAAPTVSPLARFLLTRWQNQVSQDLNSV*

>Sobic.004G064200.1

MWTKGKKSGVLAPRGIIRKREFPIRAADYELLEPVGDGATAVVRRARCLPLGGEVVAVKIMNMAHRTESDVNNASEEVKTMIMINHPNLLSAYCSFTEGEALWIVMPYMAGGSCYHLMKSSYPKGFDDENFIAFVLRETLKGLEYLHENGHIHRDVKAGNILLDQDKGVKLSDFGVTASLYDSIINRHGKRKTLVGTPCWMAPEVMEQKDYDFKADIWSFGITALELAIGHAPFSSQPPAKVFLMTLQHAPPSLHNTKEKKFTDSFKSMIATCLIKDPTKRPPAKKLLKHPFFKRAKSDHNVVKCMLNKLPSLAERMQFIKENEALQADKKPLDNCKEKASQEEYRRGVSEWNFDIADLKAQAALYPDENEAEDFLRFLFELDIVDETTQLKDIRAQSHSINDDKMNVGDDGSGKSNSTSPMSLSQSVKQLDKGSPNDLVRSESFEIHSISPAKQLTRAVSTCKDVDEYLEKTAFQKGRFKVIHDYSKIEGATPREKELLERISSLEQMLLATQDEVERLKAKESKGAEGCSQQQ*

>Sobic.004G327000.1

MAGPGEKAAVGGGGTERRKYPIHVEDYELYEEIGQGVSAIVYRALCKPLDEIVAVKVLDFERTNSDLNNIVREAQTMILIDHPNVVKAHCSFAKDQTLWVVMPYMAGGSCLHIMKSVHPTGFEEPIIATILREVLKGLEYLHHHGSIHRDVKAGNILVDARGGIKLGDFGVSACMFDSGDRQRARNTFVGTPCWMAPEVMEQLHGYDFRADIWSFGITALELAHGHAPFSKYPPMKVLLMTLQNAPPGLDYERDKKFSRNFKQMVAMCLVKDPSRRPSAKRLLKQAFFKQARSTDFIARKLLEGLPGLGVRYQALKEKDKDLMAQKKMSDGKKEEISQDEYKRGISSWTFDMDDLRSQASLGTECEDSISCKDSDMSFYDLDSLQDQASEGPHLSRDISMKYDADIENDMTAKDKSVVSSPACLLRNASMHGTPINGSIRKDNSTESFDMEYQEKHPDIIPNSSSHERKFSLSSCSSDGLLSSKESSRQQSSIYNRDKCNGAPLYVSDETSSEAAPKAHKSAEDHDDRSKPPLIRGRFKVIPGHVDFDKAQPPGLQKCHSMQTISRLPSLSIPSSAEVASNIIGGSFYMQLYSILQTNLLQRDQILNAMKQVSGCDMASPGIPSMASQCIPSASRSSSPSGAVSVDRSMLEAAYEREKELLNEVLELQWRLLCTQDEVQRLKAKTAQI*

>Sobic.008G011200.1

MLRAAGLFAPAASPPSSAMAPANNVSNGGSEEEARYPLNAESYRLLCKIGSGVSAVVYKAVCLPLGSSSSSSAVVAIKAIDLERSRVDLDGVRREAKAMALLSHRNVLRAHCSFTVGSHLWVVMPFMGAGSLHSILRHGFPGGLPEPCVAVVLAETLRALCYLHGQGRIHRDIKAGNILVDSDGAVKLADFGVSASIYETMHASASASAATAALGLGSSASCCFNDVAGTPYWMAPEVIHSHVGYGIKADIWSFGITALELAHGRPPLSHLPPSKSMLMRITSRVRLEDEESAAASSSSNKFSRAFKDMVSACLCQEPAKRPSAEKLLRHPFFKACSRRSKDFLVRNVLAAVPSIEERCSKDDDAGNDLCGCVVGARGGGARCVSPCRRHAADDDVVGVKNRRISGWNFNEDNLELDPTTTEEPAAEKRCVPFENPVVELDDSDSTGDGDRRRRPSHGDEDQDHGKAAEVVGFKEQQEVVTRKLMAVLQSLEMQRDMVTNVLERTAGSFDGGGNGGACGESVTGPRQEEREEMLLGYVRQLEHRVGDLRMEVEEEMAWNARLEKMMLQEKEINSSQASAGNSFS*

>Sobic.010G139600.1

MEHALISRRFPTDPNEYKLYEEIGEGVSATVYRALCVPVDILVAIKVLDLEKCNNDLDGIRREVQTMSLIDHPNLLRAYCSFTNGHQLWVVMPYMAAGSALHIMKTSFPEGFDEPVIATLLREVLKALVYLHSEGHIHRDVKAGNILIDTNGAVKLADFGVSACMFDTGNRQRARNTFVGTPCWMAPEVMQQLHGYDYKADIWSFGITALELAHGHAPFSKYPPMKVLLMTLQNAPPGLDYERDKRFSKSFKDLVATCLVKDPRKRPPSEKLLKHSFFKHARSAEYLARSILDGLPPLGERFRELKCKEAELLLNNKLGQESKEQLSQKEYIRGISGWNFNLEDLKNAAALIDNLNGTCHLDVRENKVKDDSQDAYNGPGHIYQERLNHVASRRPEEDEIQEVEALNNALSSSFPNHPLEALKSCFDVCGADDLDPTATDSRAQPSVRTLPFQQLQKMEHCKSANCNGESLERSLSVPKNLVTSGYHRHSSGSLIPEQVLSPYSSSDLERDGFRQKNLSSRNRSGPLLFRQLKDSRTHQSVAPEESSEGNIIRRRGRFQVTSDSISQKVATSACSSSSRINLPIEAAQSNPKSPAILPTLQFLMQQNTMQKEVLSRLISSIEETSDDSLARTSSSYQSSGGPAREKELHSYVLQLQRSVTELAEEVQRLKLQNNQLEQQINVLSRKDERSQTQDNRQ*

>Sobic.001G193000.1

MGRNGSVKRAASSGAAAGPPSFSVNPADYRLMEEVGYGAHAVVYRAIFLPTKGVVAVKCLDLDQLNNNIDEIQREAQIMSLIDHPNVIRAYCSFVVEHSLWVIMPFMTEGSCLHLMKISYQEGFDEPVIGSILKETLKALDYLHRQGQIHRDVKAGNILIDGAGVVKLGDFGVSACMFDRGDRQRSRNTFVGTPCWMAPEVLQPGTGYNFKADIWSFGITALELAHGHAPFSKYPPMKVLLMTLQNAPPGLDYERDRRFSKSFKEMVAMCLVKDQTKRPTAEKLLKHSFFKNAKPPELTIKSILSGLPPLWDRVKALQLKDAAQLALKKMPSSEQEALSLSEYQRGVSAWNFDIEDLKAQASLIHDDEPPEIKEDEDIARNIEVEKDLSSRNHLGKSSANECNSRQRAFASTLNSDGNSPTTNEAFDFDFDFSDADTTRRADGYESNIRENDSLPSTSKRDPESNHWTNDGGQRQLTSGGSNSSATERGYGFERDAAVQMISDKQRSEMRKTASLSGPLSLPTRASANSLSAPIRSSGGYVDSSGDKSKRSVVEIKGRFSVTSENVDLAKVQEGPLSSLSRKSPEGSLLRKSASTGDCLVNPKLMCNANQLKELCNSSVSSSVLIPHLNNLVQQTMFQQDLIMNLLSNLQQNEKVDGTQPGSSQVRTVENDKVADTANSEKERSLLVNISELQSRMITLTDELIAAKLKHVQLQQELNALYCREEIEDFRDEDNEET*

>Sobic.001G533700.1

MVRTGSMRRSSAPTPTRVAAPAFTVSPADYRLLEEVGYGANAVVYRAEFIPTGRTVAVKCLDLDRVNSNLDDVRKETQTMSLIDHPNVIRSYCSFVVGHNLWVVMPFMSEGSCLHLMKVAYPDGFEEPVIASILKETLKALDYLHRQGHIHRDVKAGNILIDNPGVVKLGDFGVSACMFDRGDRQRARNTFVGTPCWMAPEVLQPGTGYNFKADIWSFGITALELAHGHAPFSKYPPMKVLLMTLQNAPPGLDYDRDKRFSKSFKEMVAMCLVKDQTKRPTAEKLLKHSFFKNVKPPELTVKSILTDLPPLWDRVKALQLKDAAQLALKRMPSSEQEALSMSEYQRGVSAWNFDIEDLKAQASLICDDDPPEIKEDDDTGRITDIDKDTSSDGYFGKPTPSNGNNCSKRSSAAANPCPNGPETSEVLSSNNGSTYSERKADGPKNPGPENDSLPSTSKHDPDGKDYRSEVGQKQRTYSGPVLQSGPHDSSMTERGHIIERDAGVRSVSDKQKNGTRRANNLSGPLSLSTRASANSLSLSRTNNLSGPLSLSTRASANSLSAPIRSSAGYVGSLGDKPKRTMVEIKGRFSVTSENVDLAKVQEIPGSSTPRKLQEGPSLRKSASVGDWSVNDKTTSTNHQRKELCNSSVSTSILIPHLQNLVKQTAFQQDLITNLLSSLQQNERVDAAQSRVQSTGSDTVVEAATAEREHSLLVKIFELQSRMISLTDELIATKLKHVQLQEELNALYCQEEIVDMREDENEEA*

>Solyc02g086790.2.1

MEQLSEKKFPVNAKDYTLYEEVGDGVSATVYRALCIPLNEIVAIKVLDLEKCNNDLDGIRREVQTMILIDHPNVLRAHCSFTAGHSLWVVMPFMAGGSCLHIMKSSYPDGFEEPVIATLLREVLKALVYLHYHGHIHRDVKAGNILIDSNGAIKLADFGVAACMFDTGDRQRSRNTFVGTPCWMAPEVMQQLHGYDFKADIWSFGITALELAHGHAPFSKYPPMKVLLMTLQNAPPGLDYERDKRFSKSFKEMVAACLVKDPKKRPSSEKLLKHPFFKQARGHDYLARTILDGLPPLGDRFRMLKAREADYLLQNQAMYEDKDHLSQQEYIRGISAWNFNLEDLKNQAALLPDFDDIPDAEDSSNRGKLREGNGDVGSVVERHNQSSVLSHEDEMNGNHDLDGSLAAFPIKPLQALKGCFDMCEDDITASSPSWEDTMQSESNQQNDMLSLAKVEDQDGGKDDGENLGSLLQDNALSPKKVVTDGEREYQHPKYQSERNYSGPLQYRHKKDLGEDSSEGAIVQRKGRFQVTSADLSPKEPTSYFLNPVQGGSTSAINLGLAAASLLPTLQCILQQNTLQREELVKLIKFAERGSVNPTDLAEAGTSDLPQMPATSVRERELQSMVIQLQQSIGSLVEELQRQKMKNVQLEKKLNR*

>Solyc03g117790.1.1

MDLKLIPLEVDFVVWIFLRDRAGDIEIMEKKKYPIGPEHYTLFEEVGQGVSASVHRALCVSLNEVVAVKILDFERDNSDLNNISREAQTMVLVDHPNVLKSHCSFVSDHNLWVIMPYMAGGSCLHILKAAHPDGFEETVIATVLREVLKGLEYLHHHGFIHRDVKAGNILIDSRGGIKLGDFGVSAYLFDSGDRQRMRNTFVGTPCWMAPEVMEQLHGYDFKADIWSFGITALELAHGHAPFSKYPPMKVLLMTLQNAPPGLDYERDKKFSKSFKQMIASCLVKDPSKRPSAKKLLKHPFFKQARSNDYIGRTLLEGLPALGDRMKALKRKEEDMLAQKKIPDGQKEEISQNEYKRGISSWNFNLEDLKAQATLIPDEEILGYKDLGGSSNSLSGLDIPGKQLNKFQHQFSFSSQYSDATEFDSNNPSAPPSPATQNVAYNITKCEKSDDDLSIASSFHDHQISQNSSPCYDNRMELNSAGKGDQVADAKLFEGMPTNSRQSDKSQFQNVSSCNGTSVLQTIDDVPTEVISKHCRTSASSEDFDEKTKGHVVQQRGRFKVTSENVDLEKVGASPMLHKSQSMLVMPQTLAASQPLPLDVTPPNLLTPSHFPALQSILEANILQRESILRLMRQVAVGDNTVDAGCMPLNSLGVEKSLLEVAHDKEKELISENTELQWRLIRAQEELQKYKAENAQNNS*

>Solyc05g012130.2.1

MEFRPSSWRSRKPPPPAKQSDIYSTFVIHDNDRKTDEKDNSDLYATMVCKDDDDDDVVDDLNDDESLPPLLKRLPKDFGGGGGAIDSVSDDDMASISGTMIVKTDRSSKFTTPKQPQQQTARYMSYWDRDEKSPVRRRYEEDEDEDEEEEEEDGRFSTFVVKDNEFDSGTMVRRTVRSGSNEGAGSTMSRAVASMQAAGEIGIGRQRNRSSRAPSDEEGGTLRPQGSKVSSSSIPDSVTREDPCTKYELLHELGKGSYGAVYKARDLRTSEMVAIKVISLSEGEEGYEEIRGEIEMLQQCSHPNVVRYLGSYQGEEYLWIVMEYCGGGSVADLMNVTDEALEEYQIAFICREALKGLSYLHSIFKVHRDIKGGNILLTDQGEVKLGDFGVAAQLTRTMSKRNTFIGTPHWMAPEVIQESRYDGKVDVWALGVSAIEMAEGLPPRATVHPMRVLFMISIEPAPMLEDKEKWSLVFHDFVAKCLTKDPRLRPTASEMLKHKFIEKFKAGASVMMPKIEKAKQIRASMALEAQNIASETPEVLGGPKVNDEFGDTVPSKPKNDDAPSTSLEPVGEGDFGTMIVRDGPDIDKTASQIRNAEASSTLRRTGIPSIPTIAGKSNDPWLLNDIDVSSPVGMSQRQSMQVSSPGTLPSPDQGLKGSTTSQATVSSGGGGYNTGTLPNETVSRRALDKLRSIYSAGNTVPIPFLRATDISPIALLSEDVLGDWQRDNSGKTAVEAMQELFSGDSQSKKGRSRQNEVPLPPSVYQRLTSSPTLMNLAQALAYHKMCYEEMPLQEMQASQEQQTIQNLCDTLRTILRL*

>Solyc06g053730.1.1

MAYNQEEQNQEDHKRIQYPLDSTCYRILDEIGRGVSAIVYKAICIPMNSSVVAIKAIDLDQSRADLDNIRREAKTMSLLSHPNILKAHCSFTVDRCLWVIMPFMSAGSLQSIISSAFPDGLSEPCIALVLKETLNALAYLHNQGHLHRDIKAGNILIDSDGTIKLADFGVSASIFEPISGYGSSFSSSSSCLMFTDVAGTPYWMAPEVIHSHTGYSFKADIWSFGITALELAHGRPPLSHLPPSKSLFMKITKRFRFSDYEKTKNSKKFSKGFKDMVGLCLDQDPFRRPTAEKLLKHPFFKSCNKGPDFLVKHVLQGLPSVEQRFKQVKIHRLLSSKKSDGDDDDDDPENGEISKQRRISGWNFNVDGFELDPVLSTTEKDQDISSLKPNYVDDVVKQVDVSELFSDTSTISSPGGSRQSSEVEGVAMNCSGDRRIGGESVSREVMLASLLFLKKSLDDQRQNVMNLISIFHGEQHVVDVNKKDNLIEVIDKLRNEVENEKKKNSTLQLEIEFLKSHYSNE*

>Solyc06g068590.2.1

MEKKTNLPKQPSAKRTQYPIGSEQYTVYEEIGKGVSALVHRALCIPLNEIVAIKILDFERDKCDLNSVSREAQIMVLVDHPNVLKSYCSFVCDYNLWVVMPYMAQGSCLHILKSAYPDGFGEVVIATILREVLKGLEYLHDHGYIHRDVKAGNILIDSRGGIKLGDFGVSAYMFDSGDRQHMRNTFAGTPCWMAPEVMEQVHGYDFKADIWSFGITALELAHGHAPFSKYPPMKVLLMTLKNEPPRLDSERDRKFSKSFRQMIASCLVKDPSKRPTAKKLLKHPFFKKARSNDYVARTLLEGLPSLGDRMQELKRKEEELLAQKKMPDGQKEEMSQNEYKRGISCWSFDVEDLKAQASLIPDEEIIGENDHGGSSDALAGVDNQGNRLQYQLSSPELPKLQYQVSFGSQSSDATGFDGNNPSAPPSPAERTMDYSRHKTEKFDDDLSIASPLHDAQISQNSSPCQEEMNFVGKGEQKIHAETFEGIPINTCQRLYLAGSGEDCEEKTKYQVVQQKGRFKVTSEEKATPENLTATQPLPSDPTPANHSSQSIFPVLQNALQATIIGRESILSAMRQVADSTANLAMDAGCNPSNSAGVEKSLVWICTNLVATSSANSTITRNYYPIISLVSLFLGGSNILYNTLLSFSVVGGSS*

>Solyc07g062940.2.1

MADAAALIEASGSRFSDLELIGRGSFGDVYKGFDKELNKEVAIKVIDLEESEDEVEDIQKEIAVLSQCRSPYITEYYGSYLHQTKLWIIMEYMAGGSVADLIQPNQPLDEMSIACILRDLLHAIEYLHSEGKIHRDIKAANILLSENGDVKVADFGVSAQLTRTISRRKTFVGTPFWMAPEVIQNSDGYNEKADIWSLGITAIEMAKGEPPLADLHPMRVLFIIPRENPPQLEEHFSRPLKEIVSLCLKKSPAERPSAKELLKHRFIRNARKSPRLLERIRERPKFQIKDDMDSPRNGQKPVGEASGTVRVTRDVGTEGTVKVSGQGKTLKTAGWDFSIGGSSSTGTVRSVKPPQVRDRKPEVPLNQPASRKNLDSASNWSSASGTVHYTSSEGFNQKDGGDANTEKGDYSHEDEELSVSGTGTVVVRSPRGSPRGIQSTSLFSDQSSLSSSTLASFEDASSSGTVVYRGRHDDPDSPRTPKSRLGIQERSSSASLEDSSANLAEAKAAMLAGLKKENVRDRSKLGKVQRDGLENKTEQPTMSSDSSRHSRDYLDAQKVFSRSRHTSDEEDGARTYPIPSSATLSVLLIPSLKEAATSETDGSLMQAMVSSFMDMERMKAGSCDLFMTRLLQRLASAKDPSLKDLQDLAGRIFSKGKIETESTTTESDSKKKQQSKELNSNANLSPLARFLLSRACFSRSKFLNIELGRIWGTHLG*

>Solyc08g008550.2.1

MTGGQKSYSVNPSDYKLLEEVGYGASATVYRTIYIPSNEVVAVKCLDLDRCNSNLDDIRREAQTMSLIGHPNLIKAFCSFVVDHYLWVVMPFMAEGSCLHLMKISYPDGFEEAVIGSILKETLKALEYLHSHGHIHRDVKAGNILLDTNGVVKLADFGVSACMFDSGDRQRSRNTFTGTPCWMAPEVLQPGTGYDFKADIWSFGITALELAHGHAPFSKYPPMKVLLMTINSAPPGLDYDRDKKFSKSFKEMVAMCLVKDQTKRPTAEKLLKHIFFKKVKPPELSVKKLFADLPSLGSRVKMLQLKDAAQLASKKMPSSEQEAISQSEYQRGVSAWNFDLEDLKFQASMVQDDDEIPEIREEDESIKSYMNYKENSVSALNAGKSSLKQDIISSEHGSVGEVTLAECQRKKGEDLECNKLDSDHQEEGGLKKNSSKTELPPLTSDKEAVQAKSKCQTAKTCRSQSGPLMPGVELSHSASERSANFERSKIENQQAEKAHQVRRAPSFSGPLMLPNRASANSKSAPINSSGGFKDSSEDKSTANLVQIKGRFSVTSENVDLVKGSQLRKTASVGDWIMESKLMPPSRIPKELGLDNIPASVLMPHLQNLFQQTSIQQDLIVNLLSSLQPPEAGDAPQYGKSSSQCVAENNGSVDAAVSERERMLLVKISELQARMSSLTDELTTEKLKYMQLQQRLNSMSNQGEDWDRRESES*

>Solyc08g083040.2.1

MGGGGPRNYSANPNDYKLLEEVGYGASATVYRAIYLPYNEVIAVKCLDLDRCNSNLDDIRREAQTMSLIDHPNVIKSFCSFVVENYLWVVMPFMAEGSCLHLMKIAYPDGFEESAICSMLKETLKALEYLHRHGHIHRDVKAGNILLDTNGAVKLGDFGVSACMFDSGDRQRSRNTFVGTPCWMAPEVLQPGTGYDFKADIWSFGITALELAHGHAPFSKYPPMKVLLMTIQNAPPGLDYDRDKKFSKSFKEMVAMCLVKDQTKRPTAEKLLKHSFFKYAKPPELSVKKLFADLPPLWNRVKALQLKDAAQLALKRMPSSEQEALSQSEYQRGVSAWNFDLEDLKLQASLVQDDDEIQEIKEEDDTIKAYMNYKEKSVALQYAGKSTPRKDSIASEQESIGEVPVAEYEIKKGKDLESSTPDSDHWEKNGLKKNASKTELPPLTSDRDVLPAKSRTQTPKARQSQSGPLMAGAVLSHSASERVRSSERSEIENQQPGDKAHPVRRAPSFSGPLMLPNRASGNSLSAPIKSSGGFKDSLDDKSKPNLVQIKGRFSVTSENVDLVKDIPLCTVPRRSSQGSPLRKSASVGEWLVESKQMPPSQPPKELGVNNVPASVLMPHLQNLFQQTSIQQDLIVNLLSSLQLSEAGDSSQNGKLSPLQRPESNGIVEAAVSEREKLLLVKISELQARMINLTDELTAEKLKYWQLQQRLNAMSSCGEDGDRRELES*

>SMO116G0224.1

MEERKDKGKYPVVPADYKLLEEIGQGVSGLVFRAFCVPLGEYVAVKQLDLEKCSGTLDTIRKEAQTMSLINHSNVVKAHCSFVVSQHLWVVMPYMGGGSCLHIMKATSPDGFDEPVIATILRDTLKALEYLHRQGHIHRDVKAGNILIHENGAVKLGDFGVAAFMFDNGDRQWSRKTFVGTPCWMAPEVMEQINGYDFKADVWSFGITALELAHGHAPFSKYPPLKVLLMTLQNAAPGLTYERDKRFSKSFKEMIAMCLVKDPTKRPTAEKLLKHSFFKSAKSSDYIVRHVLDGLPPLWERVRDVKLKDAARLAEKKTSTFEEQEEKSQNAYKRGVSGWNFDVEDLKAQAAMVT*

>SMO134G0223.1

AKRRGSSSPVHENVAREDPSLKYDLLNQLGKGSYGAVYKARDRRTSEFVAIKVISLTEGEEGYDEIRGEIEMLQQCNHPNVVRYLGSYQGEEYLWIVMEYCGGGSVGDLMSITDEPLEEVQIAYICREALKGLAYLHAIFKVHRDIKGGNILLTEQGEVKLGDFGVAAQLTRTMSKRNTFIGTPHWMAPEVIQESRYDGKVDVWALGVSAIEMAEGLPPRSNVHPMRVLFMISREPAPMLEDKEKWSLVFHDFVAKCLTKEPRLRPTATALLQHKFIEKCKGSATSMLPRIERARALKELSARTFGPEQATRGQTVKMHESVAATVLVRPDKEKADIDNEDTDVGDFGTMVVHRTSSLTAQSSDLLQLNQSSTDLQGDNPTVKSNAKVESEKVVGEERHDEPVIAAPIDLPPFSPATPPSLSPNQQLSNESQTLRRRKSPGDGASTVGGTTPRNLGGFALHDKLVSIYAAGNTVPIPFLRATDISAIALLSDDVLGNGFADHSGAVALQTVEELYNAAGVADSTFKKGKKPGTSETPLPASVYQSLATSSTLQNLARALAYHKRCYEEMPLQGWQAAQEERVIGNLSDTLRTILRL

>SMO169G0133.1

ARKPYPVAASDYRVLEEIGHGRNATVHRALCVPRGEIVSIKSIDLEKCRSDLDEVRREAQTLSLIDHPNVVAALALFIVGQRLWVVMPYMAAGSCLTIMRVARPYGLDELLVATVLRECLKALDYLHFHGHIHRDVKAGNILVDQHGGVKLGDFGVSACLFDCYNRQIARRTTFAGTPCWMAPEVLDPVCGYDCSADIWSLGITALELAQGHAPLSDLPPMKMVLVELSSPPPTLEPERAKVFSKSFKDFVACCLQKEASKRPTAGKLLKHGFFKHAQSGEYLVEHLLRELPPLWEQVRELRNRD

>SMO351G0517.1

MSNVGAATAAATAGTSSSSSAAAAAAVAAAAAAGIDSRFDNLQLIGKGSFGDVFKGFDKELNKEVAIKVIDLEEAEDEIDEIQKEISVLSQCRSPYITEYYGSFLHGTKLWIVMEYMAGGSVSDLLETGNPLDELSIACIIRDLLHALDYLHSEGKIHRDIKAANILLTANGDVKVADFGVSAQLTRTVSKRKTFVGTPFWMAPEVIQNSDGYNEKADIWSLGITTIEMAKGEPPFADLHPMRVLFLIPKNNPPQLEDHFSRPMKEFVSLCLKKNPAERASAKELLKHRFVRNARKSPRLLERIRERPKVHIRKPRSEHMYEQDTVEKPKGLKAAKDGESHLLSLTKAMRNASWDFGTGTIQGISQGTGTVRSGLRTPFGFDDRVESFEGDMSPRISSRSHDWHRTSASSSTEQQAAAQEPETSLDSQGEAYDPDEASESSSPIFDSGTFVAREKSSESTSNSETPRYQPQLERSESSLQEDSAKNLAEAKAALQSGSRKGMGREKQSSSKRREDPVVTTDPSTSADPGRKSSYLDVPKVRLSSGEEEHARTLITEGSPALSLLLIPALKETAAEQSEGSALRAAADAADALIDLERMAPGACEVLVTRLLKRLGSAEDPSVKTLQDLARKALVVDHPPGAGDGHLQSTTQQFDFAKSGQQRGQPNLESSESSGLSPLASFLLSRWQSQLPRDMNCYN*

>SMO356G0010.1

GGGVAMEDKRQYPALASDYQLLEEVGQGVSATVYRAMCISFKEIVAIKSLDLEKCSSNLDEIRKEAKTMSLINHPNVVRAYCSFVVEHSLWVVMPYMAGGSCLHIMKAAYPDGFEEAVIATVLKDTLKALEYLHRHGHIHRDVKAGNILIDSNGAIKLGDFGVSACLFDTGDRQRSRNTFVGTPCWCLPCECRMAPEVMEQIHGYDFKADIWSFGITALELAHGHAPFSKYPPMKVLLMTLQNAPPGLDYERDRRFSKSFKEMIAMCLVKDPTKRPTAEKLLKHSFFKGAKSTEYLVRHVLEGLPPLWERVRTLKINDAARLAQKKIPYGEQEEQSQKEYKRGVSSWNFDVEDLKAQAALIQDDDDVYPEKNDENSSF

>PGSC0003DMT400042095

MAYNQEEQNEEEHKKIQYPLDSTCYRILDEIGRGVSAIVYKAICIPMNSSVVAIKAIDLDQSRADLDNIRREAKTMSLLSHPNILKAHCSFTVDRCLWVIMPFMSAGSLQSIISSAFPDGLSEPCIAIVLKETLSALAYLHNQGHLHRDIKAGNILIDSHGTIKLADFGVSASIFEPISGYGSSFSSSSSCLMFTDVAGTPYWMAPEVIHSHTGYSFKADIWSFGITALELAHGRPPLSHLPPSKSLFMKITKRFRFSDYEKTKNSKNYKKFSKGFKDMVGLCLDQDPFRRPTAEKLLKHPFFKSCNKGPDFLVKHVLQGLPSVEQRFKQVKIQRLLSLKKSDGDDDDDPEHGEISKQRRISGWNFNVDGFELDPVLFPTEKDQEISSLKPNYVDDVVKQVDVSELFSDTSTISSPGGSRQSSEVEGVAMNCSGDRRIGGESVSREVMLASLLFLKKSLDDQRQNVMNLISIFHGEQYVVEVNKKDNLIEVIDKLRNEVENEKKKNSALQLEIEFLKSHYSNE*

>PGSC0003DMT400074400

MEKKTNLPKKPIEKRTQYPIGSEQYTVYEEIGKGVSALVHRALCIPLNEIVAIKILDFERDKCDLNSVSREAQIMVLVDHPNVLKSYCSFVCDYNLWVVMPYMAQGSCLHILKSAYPDGVGEVVIATVLREVLKGLEYLHDHGYIHRDVKAGNILIDSRGGIKLGDFGVSAYMFDSGDRQHMRNTFAGTPCWMAPEVMEQVHGYDFKADIWSFGITALELAHGHAPFSKYPPMKVLLMTLKNEPPRLDSERDRKFSKSFRQMIASCLVKDPSKRPSAKKLLKHPFFKKARSNDYVARTLLEGLPSLGDRMQELKRKEEEMLAQKKMPDGQKEEMSQLEAALDREKDLHREVADLRWRYYDYTLILPVNFGYYK*

>PGSC0003DMT400036874

MDKKKYPIGPEHYTLFEEVGQGVSASVHRALCISLNEVVAIKILDFERDNSDLNNISREAQTMVLVDHPNVLKSHCSFVSDHNLWVIMPYMAGGSCLHILKATHPDGFEETVIATVLREVLKGLEYLHHHGFIHRDVKAGNILIDSRGGIKLGDFGVSAYLFDSGDRQRMRNTFVGTPCWMAPEVMEQLHGYDFKADIWSFGITALELAHGHAPFSKYPPMKVLLMTLQNAPPGLDYERDKKFSKSFKQMIASCLVKDPSKRPSAKKLLKHPFFKQARSNDYIGRTLLEGLPALGDRMKALKRKEEDMLAQKKIPDGQKEEISQNEYKRGISSWNFNLEDLKAQATLIPDEEILGDKDLGGSSNSLSGLDIPGKQLNKFQHQFSFSSQYSDATEFDSNNPSAPPSPATQNVAYNITKCEKSDDDLSIAGSFHDPQLSQNSSPCYDNRMELNLVGKGEQVADAKLFEGMPVNSRQSDKSQFQNVSSCNGTSVLQTIDDVPTEVISKHCRTSASSEDFDEKTKGHVVQQRGRFKVTSENVDLEKVGASPMLHKSQSLLVMPQTLAAAQPLPLDATPPNLLTPSHFPALQSILEANILQRESILRLMRQVALGDTAVDAGCMPLNSLGVEKSLLEVAHDKEKELISENTELQWRLIRAQEELQKYKAENAQNNS*

>PGSC0003DMT400036872

MDKKKYPIGPEHYTLFEEVGQGVSASVHRALCISLNEVVAIKILDFERDNSDLNNISREAQTMVLVDHPNVLKSHCSFVSDHNLWVIMPYMAGGSCLHILKATHPDGFEETVIATVLREVLKGLEYLHHHGFIHRDVKAGNILIDSRGGIKLGDFGVSAYLFDSGDRQRMRNTFVGTPCWMAPEVMEQLHGYDFKADIWSFGITALELAHGHAPFSKYPPMKVLLMTLQNAPPGLDYERDKKFSKSFKQMIASCLVKDPSKRPSAKKLLKHPFFKQARSNDYIGRTLLEGLPALGDRMKALKRKEEDMLAQKKIPDGQKEEISQNEYKRGISSWNFNLEDLKAQATLIPDEEILGDKDLGGSSNSLSGLDIPGKQLNKFQHQFSFSSQYSDATEFDSNNPSAPPSPATQNVAYNITKCEKSDDDLSIAGSFHDPQLSQNSSPCYDNRMELNLVGKGEQVADAKLFEGMPVNSRQSCNGTSVLQTIDDVPTEVISKHCRTSASSEDFDEKTKGHVVQQRGRFKVTSENVDLEKVGASPMLHKSQSLLVMPQTLAAAQPLPLDATPPNLLTPSHFPALQSILEANILQRESILRLMRQVALGDTAVDAGCMPLNSLGVEKSLLEVAHDKEKELISENTELQWRLIRAQEELQKYKAENAQNNS*

>PGSC0003DMT400036873

MDKKKYPIGPEHYTLFEEVGQGVSASVHRALCISLNEVVAIKILDFERDNSDLNNISREAQTMVLVDHPNVLKSHCSFVSDHNLWVIMPYMAGGSCLHILKATHPDGFEETVIATVLREVLKGLEYLHHHGFIHRDVKAGNILIDSRGGIKLGDFGVSAYLFDSGDRQRMRNTFVGTPCWMAPEVMEQLHGYDFKADIWSFGITALELAHGHAPFSKYPPMKVLLMTLQNAPPGLDYERDKKFSKSFKQMIASCLVKDPSKRPSAKKLLKHPFFKQARSNDYIGRTLLEGLPALGDRMKALKRKEEDMLAQKKIPDGQKEEISQNEYKRGISSWNFNLEDLKAQATLIPDEEILGDKDLGGSSNSLSGLDIPGKQLNKFQHQFSFSSQYSDATEFDSNNPSAPPSPATQNVAYNITKCEKSDDDLSIAGSFHDPQLSQNSSPCYDNRMELNLVGKGEQVADAKLFEGMPVNSRQSDKSQFQNVSSCNGTSVLQTIDDVPTEVISKHCRTSASSEDFDEKTKGHVVQQRGRFKVTSENVDLEKVMPQTLAAAQPLPLDATPPNLLTPSHFPALQSILEANILQRESILRLMRQVALGDTAVDAGCMPLNSLGVEKSLLEVAHDKEKELISENTELQWRLIRAQEELQKYKAENAQNNS*

>TraesCS1A02G181900.1

MGRNGSVKRGAAAAAPPSFTVNPADYRLMEEVGYGAHAVVYRALFLPRNQTVAVKCLDLDQLNNNIDEVQREAQIMSLIDHPNVIRAYCSFVVEHSLWVIMPFMTEGSCLHLMKIAYPEGFEEPVIASILKETLKALEYLHRQGQIHRDIKAGNILIDSAGVVKLGDFGVSACMFDRGDRQRSRNTFVGTPCWMAPEVLQPGTGYNFKADIWSFGITALELAHGHAPFSKYPPMKVLLMTLQNAPPGLDYDRDRRFSKAFKEMVAMCLVKDQTKRPTAEKLLKHSFFKTAKPPESTMKGMLTDLPPLWERVKALQLKDAAQLALKKMPSSEQEALSLSEYQRGVSAWHFDIEDLKAQASLIYEDEPSETKEDDVAARIIESEKSLYSRTPSGQSSSANENTCSEETSTTNPDCRRMPNGHENSRSENDSLPSTSKEPESKYWRTNVGQKQQTTGGPVEGGVNSSTTERSHNFERDATADKHGSDTRRAANLSGPLSLPTRASANSYSAPIRSSGGYVDSLGDKSKRSVVHIKGRFSVTSENVDLAKVQEIPLSSISRKSPQGIQLRKSASVGDWIVNAKPTSNSHHLKELCNSSVSSSVLIPHLENLVQQTTLQQDLIVNLLSSMQQNEKADGAQSGTSSQVRNMQSETVVETSNTEKERSLLVKISELQSRMITLTDELISAKQKHVQLQQELNALYCREEIEDIRDEDNEET*

>TraesCS1B02G199100.1

MGRNGSIKRGAAAAAPPSFTVNPADYRLMEEVGYGAHAVVYRALFLPRNQTVAVKCLDLDQLNNNIDEVQREAQIMSLIDHPNVIRAYCSFVVEHSLWVIMPFMTEGSCLHLMKIAYPEGFEEPVIASILKETLKALEYLHRQGQIHRDIKAGNILIDSAGVVKLGDFGVSACMFDRGDRQRSRNTFVGTPCWMAPEVLQPGTGYNFKADIWSFGITALELAHGHAPFSKYPPMKVLLMTLQNAPPGLDYDRDRRFSKAFKEMVAMCLVKDQTKRPTAEKLLKHSFFKTAKPPESTMKGMLTDLPPLWERVKALQLKDAAQLALKKMPSSEQEALSLSEYQRGVSAWHFDIEDLKAQASLIYEDESSETKEDDVAARIIESEKSLYSRTPSGQSSSANENTCSEETSTTNPDCRRMPNGHENSRSENDSLPSTSKEPESKYWRTNVGQKQQTSGGPVEGGVNSSTAERSHNFERDATADKHGSDTRRASNLSGPLSLPTRASANSYSAPIRSSGGYVDSLGDKSKRSVVHIKGRFSVTSENVDLAKVQEIPLSSISRKSPQGIQLRKSASVGDWIVNAKPMSNSHHLKELCNSSVSSSVLIPHLENLVQQTTLQQDLIVNLLSSMQQNEKADGAQSGTASQVRNMQSETVVETSNTEKERSLLVKISELQSRMITLTDELISAKQKHVQLQQELNALYCREEIEDIRDEDNEET*

>TraesCS2A02G233400.1

MSDSASMAAATEARFSNRDLIGRGSFGDVYRGFDKELSKEVAIKVIDLEEAEDDIEDIQKEISVLSQCRCPYITDYYGSYLHQTKLWIVMEYMAGGSVADLLQAGPPLDEISIACILRDLLHAVEYLHSEGKIHRDIKAANILLTESGDVKVADFGVSAQLTKTMSRRKTFVGTPFWMAPEVIQNSDGYNEKADIWSLGITAIEMAKGEPPLADIHPMRVLFMIPRENPPQLDEHFSKPMKEFVSLCLRKNPAERPSAKELLKHRFIKNARKTPKLLERIRERPKFTVKGSIDATQNGQTHIEEDDYGGTIKVDRNTRHAASPSSQGTVRKAAGWDFPDRSEGTGTVRGGLRPPQITSTKDGRFDMPQNPSTLKRTVDRENQWRTSGTGSEESSSTNMSKKEAQTDHGRLESSTEYNDQSVSGSGTVVLRSPRASQVYPAGPNHSSKPPSRFSSYEDMSISGTVVRNQTEEAETPRSSRSRLGTLEKTSNASLEDSATNLAEAKAALQAGFRKGNARERPATNKHEKESHEPRFSGVNSHEVRSENVDSQKGRKSRQPSDGQSAPRASAASPALSSLIIPSLKEASGDKYEGPVVHAVLSSLMDLEQEIPGSCEVLVGRILQRLGSSKDSSLQSLNETAISIFTKKPEPPSEAGSNKKQASTPPLAAPTVSPLARFLLTRWQNQVSQDLNSV*

>TraesCS2B02G249900.1

MSDSASMAAATEARFSNRDLIGRGSFGDVYRGFDKELSKEVAIKVIDLEEAEDDIEDIQKEISVLSQCRCPYITDYYGSYLHQTKLWIVMEYMAGGSVADLLQAGPPLDEISIACILRDLLHAVEYLHSEGKIHRDIKAANILLTESGDVKVADFGVSAQLTKTMSRRKTFVGTPFWMAPEVIQNSDGYNEKADIWSLGITAIEMAKGEPPLADIHPMRVLFMIPRENPPQLDEHFSKPMKEFVSLCLRKNPAERPSAKELLKHRFIKNARKTPKLLERIRERPKFTVKGSIDATQNGQTHIEEDDYGGTIKVDRNTRHAASPSSQGTVRKAAGWDFPDRSEGTGTVRGGLRPPQITSTKDGRFDMPQNPSTVKRTAERENQWRTSGTGSEESFSTNMSKKEAQTDHGRLESSTEYNDQSVSGSGTVVLRSPRASQVYPAGPNHSSKPPSRFSSYEDMSISGTVVRNQTEEAETPRSSRSRLGTQEKTSNASLEDSATNLAEAKAALQAGFRKGNARERPATNKHEKESHEPRFSGVNSHEVRSENVDSQKGRKSRQPSDGQSAPRASAASPALSSLIIPSLKEASGDKYEGPVVHAVLSSLMDLEQEIPGSCEVLVGRILHRLGSSKDSSLQSLNETAISIFTKKPEPPSEAGSNKKQASTPPLAAPTVSPLARFLLTRWQNQVSQDLNSV*

>TraesCS2D02G232200.1

MSDSASMAAATEARFSNRDLIGRGSFGDVYRGFDKELSKEVAIKVIDLEEAEDDIEDIQKEISVLSQCRCPYITDYYGSYLHQTKLWIVMEYMAGGSVADLLQAGPPLDEISIACILRDLLHAVEYLHSEGKIHRDIKAANILLTESGDVKVADFGVSAQLTKTMSRRKTFVGTPFWMAPEVIQNSDGYNEKADIWSLGITAIEMAKGEPPLADIHPMRVLFMIPRENPPQLDEHFSKPMKEFVSLCLRKNPAERPSAKELLKHRFIKNARKTPKLLERIRERPKFTVKGSIDATQNGQTHIEEDDYGGTIKVDRNTRHAASPSSQGTVRKAAGWDFPDRSEGTGTVRGGLRPPQITSTKDGRFDMPQNPSTLKRTADRENQWRTSGTGSEESSSTNMSKKEAQTDHGRLESSTEYNDQSVSGSGTVVLRSPRASQVYPAGPNHSSKPPSRFSSYEDMSISGTVVRNQTEEAEMPRSSRSRLGTLEKTSNASLEDSATNLAEAKAALQAGFRKGNARERPATNKHEKESHEPRFSGVNSHEVRSENIDSQKGRKSRQPSDGQSAPRASAASPALSSLIIPSLKEASGDKYEGPVVHAVLSSLMDLEQEIPGSCEVLVGRILHRLGSSKDLSLQSLNETAISIFTKKPEPPSEAASNKKQASTPPLAAPTVSPLARFLLTRWQNQVSQDLNSV*

>TraesCS4B02G395600.1

MRRPSLAVAEPAAPEFTLSAGDYRLMEEVGFGANAVVYRAIFLPANCTIAVKCLDLDRVNSNLDDVRKEAQIMKSIDHPNVIKAYCSFVVDHNLWVIMPFMAEGSCLHLMKVAHPDGLEEPVICSILKETLKALAYLHGQGHIHRDVKAGNILVDSPGVVKLGDFGVSACLFDRGDRQRSRNTFVGTPCWMAPEVLQPRTGYNFKMHHQVFDYDRDRKFSKSFKEMVAMCLVKDQTKRPTAEKLLKHSFFKNAPQLTVKSILTFAPPLWDRVKALQQKDAAHLASSEQEALSMYQRGVRASPARRTCTTAVNPGGNGPETSDEFACDLGNADSPRMVDGHIKQGTENDSLSSTSKQGSEGGNRRSEVRQRQRTFSGPVMDAGARSPNKQKSDTGRIDDLSGPLSLSTRASANSLSAPIRSSGGYVGSLGVKPRVEIKGRFSVTSENVDLAKNSGLYNERSASVGAWPVKAKSILQSNSHHRKEFRDSSVSASILIPHLENLVQQTTFQQDIITNLMSNLQQNEKPNATEAPPQPLPPQRRHTRTLALTPTLALTEKEGKELLVKKVVPPQGHAGAALFSLTETEKKRKKKGKGKGREEINKVHDLFFQKIFSVKNKKFMNFYSCFFAAK*

>TraesCS4B02G398400.1

MVRGGSMRRPSLAAVSAAAAEPAAPEFTLSADDYRLMEEVGFGANAVVYRAIFLPANRTIAVKCLDLDRINSNLDDVRKEAQIMKLIDHPNVIKAYCSFVVDHNLWVIMPFMAEGSCLHLMKVAHPDGLEEPVICSILKETLKALAYLHGQGHIHRDVKAGNILVDSPGVVKLGDFGVSACLFDRGDRQRSRNTFVGTPCWMAPEVLQPGTGYNFKADIWSFGITALELAHGHAPFSKYPPMKVLLMTLQNAPPGLDYDRDRKFSKSFKEMVAMCLVKDQTKRPTAEKLLKHSFFKNTKAPQLTVKSILTDLPPLWDRVKALQQKDAAHLASSEQEALSMSEYQRGVSAWHFDIEDLKAQALLINDDDPPELKEDDDIARITEVDKGTSFESHFGQSTLLNGNNHRLNHERTCTTAVNPGGNGPETSDVFTSDLGNADSPRMVDGHTKQGSENGSLSSTSKQGSEGGNHRSEVRQRQRTFSGPVMYSGTRSSSLIERGYIIDKDAGARSPNKQKSDTGRIDDLSGPLSLSTRASANSLSAPIRSSGGYVGSLGDKPRVEIKGRFSVTSENVDLAKSNSHHRREFRDSSVSASILIPHLENLVQQTTFQQDIITNLMSNLQQNEKPNGPQTRVQTMVGDTGVETGSAERERKLLAKVFELQSRMISLTDELIASKLKHVELQEELNRLYCQEETGETREDDYSGDL*

>TraesCS5A02G187400.1

MADDAGPGGEAKYPLNPDCYRLLCKIGSGVSAVVYKAACLPLGSVPVAIKAIDLERSRANLEDVWREAKAMALLSHANVLRAHCSFTVGSHLWVVMPFMAAGSLHSILAHGFPDGLPEPCIAVVLKETLRALCYLHEQGRIHRDIKAGNVLVDSDGSVKLADFGVSASIYETPPPASSFSGPLTHAPQVVLSSSSYFSEMAGTPYWMAPEVIHSHVGYGIKADIWSFGITALELAHGRPPLSHLPPSKSMLMRITSRVRMEDAEISKNKKLSKAFKDMVSSCLCQEPAKRPSAEKLLRHPFFKGCRSKDYLVRNVLSVVPSIEERCKDVTGLCGCAAGGARCVSPCHGQASASIVKNRRMSGWNFGADCPRKEDADSFEDLDQTETVARLFLPLDDEDTVPERACDGAGEDGDKGTMEQQGDREENEGSFGVKGVVVPHLMTILGSLEVQKRMLAQELEGGCCYHHDGNCCRETTAREEMLLAYVRQLEQRVEVLTLEVEEEIARNAHLEELLRERAG*

>TraesCS5A02G392500.1

MAFSPRSPWSRSKKPDIYSTVVVHDDEDDARGGGAARAEDDDDDDPSALPPLLQRLPKDFGGASFDDDDDPYSSDLDDASLSATVVIKRGAPASTSSSSRSPFLDLRRSSPRAAEADPFSTFVVHGTARSGGASSPRESVSGTFIRHSGGPPSPRESVSGTFIRHTRGSSSPHESFSGTFIHHTSSASSPRDSASGAGAGFGSSFITPSSGQAEEDRQPSLLMQQQQSRRKASMSSLPDSVTREDPSTKYELLHELGKGSYGAVYKARDLRTQELVAVKIISLTEGEEGYEDIRGEIEMLQQCSHPNVVRYFGSYQGEEYLWIVMEYCGGGSVADLIGITEEPLDEPQIAYICRETLKGLAYLHTIFKVHRDIKGGNILLTEQGEVKLGDFGVAAQLTRTMSKRNTFIGTPHWMAPEVIQESRYDGKVDVWALGVSAIEMAEGMPPRSTVHPMRVIFMISSEPAPMLEDKEKWSLLFHDFIAKCLTKDARLRPPAIEMLKHKFIEKCNTGASKMLAKIKEAKIIRETAVQNQLPDSDDAMDATVRINEDYGETVPTNSQSTHETKNDGSGGDFGTMIVHPEDGDEAAESSIFPRAEFIPGLGSINSFTHDPKRAELISKFWAESTAESDASKERDLYGLPDIQEPKTMPRSTGTVKHHKGVEGTVLRHDITASPGVASTMNKLSSSPSRKAFSVQDKLWSIYAAGNTVPIPFLKAIDISPLALVSDSVAGNGPAGSSTTDALEAVRELFSGDGQAKKGRKGQNEAPLPPGVHDRLTTSPTLMNLAQALAYHKTCYEDMPLQDSQATEEQQTIQNLCDTLRTILRL*

>TraesCS5B02G196400.1

MADEAGAGGEAKYPLNPECYRLLCKIGSGVSAVVYKAACLPLGSVPVAIKAIDLERSRANLEDVWREAKAMALLSHANVLRAHCSFTVGSHLWVVMPFMAAGSLHSILAHGFPDGLPEPCIAVVLKETLRALCYLHEQGRIHRDIKAGNLLVDSDGSVKLADFGVSASIYETPPPASSFSGPLTHAPQVVLSSSSYFSEMAGTPYWMAPEVIHSHVGYGIKADIWSFGITALELAHGRPPLSHLPPSKSMLMRITSRVRMEDAEISKNKKLSKAFKAMVSSCLCQEPAKRPSAEKLLRHPFFKGCRSKDYLVRNVLSIVPSIEERCKDVTGLCGCAAGGARCVSPCHGQASASIVKNRRMSGWNFGADCPRKEDADSFEELDRTETVARLFLPLDDEDTVPERACEGAGEDGDKGVTEEQCDREENDGSFGVKGVVVPHLVTILGSLEVQKRMLAQELEGGCCYHHNGNCCRETTAREEMLLAYVRQLEQRVEVLTLEVEEEITRNAHLEELLRGRAG*

>TraesCS5B02G397300.1

MLPDSSELAPTKLIPHSPTSEYLPRPPPHLPPMAFSPRSPWSRSKKPDIYSTVVVHDDEDDARGGGGAARAEDDDDDDPSALPPLLQRLPKDFGGASFDDDDDPYSSDLDDASLSATVVIKRGAPASTSSSSRSPFLDLRRSSPRAAEADPYSTFVVHGTGRSGGASSPRESVSGTFIRHSGGPPSPRESVSGTFIRHTRGSSSPHESFSGTFIHHTSSASSPRDSASGAGAGFGSSFITPSAGQAEEDRQPSLLMQQQQSRRKASMSSLPDSVTREDPSTKYELLHELGKGSYGAVYKARDLRTQELVAVKIISLTEGEEGYEDIRGEIEMLQQCSHPNVVRYFGSYQGEEYLWIVMEYCGGGSVADLIGITEEPLDEPQIAYICRETLKGLAYLHTIFKVHRDIKGGNILLTDQGEVKLGDFGVAAQLTRTMSKRNTFIGTPHWMAPEVIQESRYDGKVDVWALGVSAIEMAEGMPPRSTVHPMRVIFMISSEPAPMLEDKEKWSLLFHDFIAKCLTKDARLRPPAIEMLKHKFIEKCNTGASKMLAKIKEAKIIRETAAQNQLPDSDDAMDATVRINEDYGETVPTNSQSTHETKNDGSGGDFGTMIVHPEDGDEAAESSIFPRTEFIPGLGSINSFTHDPKRAELISKFWAESTADSDASKERDLYGLPDIQEPKTMPRSTGTVKQHKGAEGTVLRHDITASPGVASTMNKLSSSPSRKAFSVQDKLWSIYAAGNTVPIPFLKAIDISPLALVSDSVAGNGPAGSSTTDALEAVRELFSGDGQAKKGRKGQNEAPLPPGVHDRLTTSPTLMNLAQALAYHKTCYEDMPLQDSQATEEQQTIQNLCDTLRTILRL*

>TraesCS5D02G203600.1

MADDAGAGGEAKYPLNPECYRLLCKIGSGVSAVVYKAACLPLGSVPVAIKAIDLERSRANLEDVWREAKAMALLSHANVLRAHCSFTVGSHLWVVMPFMAAGSLHSILAHGFPDGLPEPCIAVVLKETLRALCYLHEQGRIHRDIKAGNVLVDSDGSVKLADFGVSASIYETPPPASSFSGPLTHAPQIVLSSSSYFSEMAGTPYWMAPEVIHSHVGYGIKADIWSFGITALELAHGRPPLSHLPPSKSMLMRITSRVRMEDAEISKNKKLSKAFKDMVSSCLCQEPAKRPSAEKLLRHPFFKGCRSKDHLVRNVLSVVPSIEERCKDVTGLCGCAAGGARCVSPCHGQASASIVKNRRMSGWNFGADCPRKEDADSFEELDRTETTARLFLPLDDEDTVPERTCEGAGEDGDKGVTEEQGDREENEGSFGVKGVVVPHLMTILGSLEVQKRMLAQELEGGCCYHHDGNCCGETTAREEMLLAYVRQLEQRVEMLTLEVEEEITRNAQLEELLRERAG*

>TraesCS5D02G402300.1

MAFSPRSPWSRSKKPDIYSTVVVHDDEDDARGGDARAEDDDDDDPSALPPLLQRLPKDFGGASFDDDDDPYSSDLDDASLSATVVIKRGAPASTSSSSRSPFLDLRRSSPRAAEADPFSTFVVHGTARSGGASSPRESVSGTFIRHSGGPPSPRESVSGTFIRHTRGSSSPHESFSGTFIHHTSGASSPRDSASGAGFGSSFITPSAGQAEEDRQPSLLMQQQQSRRKASMSSVPDSVTREDPSTKYELLHELGKGSYGAVYKARDLRTQELVAVKIISLTEGEEGYEDIRGEIEMLQQCSHPNVVRYFGSYQGEEYLWIVMEYCGGGSVADLIGITEEPLDEPQIAYICRETLKGLAYLHTIFKVHRDIKGGNILLTDQGEVKLGDFGVAAQLTRTMSKRNTFIGTPHWMAPEVIQESRYDGKVDVWALGVSAIEMAEGMPPRSTVHPMRVIFMISSEPAPMLEDKEKWSLLFHDFIAKCLTKDARLRPPAIEMLKHKFIEKCNTGASKMLAKIKEAKIIRETAAQNQLPDSDDAMDATVRINEDYGETVPTNSQSTHETKNDGSGGDFGTMIVHPEDGDEAAESSIFPRTEFIPGLGSINSFTHDPKRAELISKFWAESTADSDASKERDLYGLPDIQEPKTMPRSTGTVKQHKGAEGTVLRHDITASPGVASTMNKLSSSPSRKAFSVQDKLWSIYAAGNTVPIPFLKAIDISPLALVSDSVAGNGPAGSSTTDALEAVRELFSGDGQAKKGRKGQNEAPLPPGVHDRLTTSPTLMNLAQALAYHKTCYEDMPLQDSQATEEQQTIQNLCDTLRTILRL*

>TraesCS6A02G353400.1

MVGSGSKHAAGGSGLGGGGERRKYPIRAEDYELYEEIGQGVSAIVYRALCRPLGETVAVKVLDFERTNSNLNNIMREAQTMILIDHPNVVKAICSFANNQTLWVVMPYMAGGSCLHIMKSVYPDGFEEAVIATLLREVLRGLEYLHHHGHIHRDVKAGNILVDSRGGVKLGDFGVSACLFESGDRQRARNTFVGTPCWMAPEVMEQLHGYDFKADIWSFGITALELAHGHAPFSKYPPMKVLLMTLQNAPPGLDYERDKKFSRHFKQMVAMCLVKEPSKRPTATKLLKQSFFKQARSHDYIVRKLLEGLPGLGARYQALKEKDEHLLAQKKMPDGRKEEISQVLILNL*

>TraesCS6A02G353500.1

MVGSGSKHAAGGSGLGGGGERRKYPIRAEDYELYEEIGQGVSAIVYRALCRPLGETVAVKVLDFERTNSNLNNIMREAQTMILIDHPNVVKAICSFANNQTLWVVMPYMAGGSCLHIMKSVYPDGFEEAVIATLLREVLRGLEYLHHHGHIHRDVKAGNILVDSRGGVKLGDFGVSACLFESGDRQRARNTFVGTPCWADIWSFGITALELAHGHAPFSKYPPMKVLLMTLQNAPPGLDYERDKKFSRHFKQMVAMCLVKEPSKRPTATKLLKQSFFKQARSHDYIVRKLLEGLPGLGARYQALKEKDEHLLAQKKMPDGRKEEISQDEYKRGISSWNFDIDDLKSQASLISECEDTISSKDTDISSIYDFDTSLQEQALEGSLFSMKYDTDIENDVMANDKSAVSSPEQSVCLSRKHSSMESCDLDLQEKDLDAIPTSSFQERKCSFSSCSSDGFLSSKESSFSSKPQINIHNRDKGSGGVLQVADEPSPEAISRAPKSLVSNVDEHDDRSKPPLIQQRGRFKVTPGNVELDKAHSPGLQKSHSMQTISQLSALSIPSSAEAASSIIGGSLYIQLYNVLQTNLLQREQILHAMKQLYISDSISPVRMHSLSRSPSPSSALSVDRSMLLSTDSAAAQQNLNQRANPAESDSRNPLPKSEPKAEAAQDVLSKI*

>TraesCS6B02G177800.1

MGSKEKGKRGEGGASPRARKGEFPIRAEDYELMEPIGDGATAVVRRARCLPLGGEVVAVKIMNLSLRSEADVNNASEEVKTMILTDHPNLLSAYCSFTQDENLWIVMPYMAGGSCFHLMKSSFPKGFEEERFIAFVLRETLRGLEYLHGKGHIHRDVKAGNILLDQHKGVKLADFGISASVYDSMINRNGKRHTLVGTPCWMAPEVMEQKEYDFKADIWSFGITALELANGHAPFSSQPPAKVFLMTLQHAPPSLHNTKDKKFSNSFKRMIGACLIKDPSKRPTAQMLLQLPFFKKVKSEDNHVRCMLNKVPSLVARVQTIKENEAKLQAEKKPHDKIKEKTSHDEYWRGISQWHFDIEDLKAQAKLYSEENDSDEEEYLRFLFELDTVDEIVPIQDVHPQNHASDDKKIVGTEIVETPSSTTPISPPQSGKQLENGPPNGLLRHESFERHSKVPTKQLSRAVSNVTCTDEYLEKTAIQKGRFKVTMEETEASTRREKELLERIACLERMLQVTQDEVVRLKEKEAKGAGPCVQQNSKVQGL*

>TraesCS6D02G335800.1

MVGSGSKHAAGGSGGGGGGERRKYPIRAEDYELYEEIGQGVSAIVYRALCRPLGETVAVKVLDFEHTNSNLNNIMREAQTMILIDHPNVVKAICSFANNQTLWVVMPYMAGGSCLHIMKSVYPDGFEEAVIATLLREVLRGLEYLHHHGHIHRDVKAGNILVDSRGGVKLGDFGVSACLFESGDRQRARNTFVGTPCWMAPEVMEQLHGYDFKADIWSFGITALELAHGHAPFSKYPPMKVLLMTLQNAPPGLDYERDKKFSRHFKQMVAMCLVKEPSKRPTATKLLKQSFFKQARSHDYIVRKLLEGLPGLGARYQALKEKDEHLLAQKKMPDGRKEEISQDEYKRGISSWNFDIDDLKSQASLISECEDTISSKDTDISSIYDFDTSLQEQALEGSLFSMKYDTDIENDVMANDKSAVSSPEQSVCLSRASLCGTSNGVLVNGHVRKHSSMESSDLDLQEKDLDAIPTSSFQERKCSFSSCSSDGFLSSKESSKPQINIHNRDKGSGGVLQVADEPSPEAISRAHKSLVSNVDEHDDRSKPPLIQQRGRFKVTPGNVELDKAHSPGLQKSHSMQTISQLSALSIPSSAEAASSIIGGSLYIQLYNVLQTNLLQREQILHAMKQLYISDSISPVRMHSLSRSPSPSSALSVDRSMLEAAQEKEKELVNEVLELQWRLLCAQDEVQRLKAKAAQI*

>TraesCS7A02G232300.1

MERAPRNRGFPTDPKEYKLYEEVGEGVSATVYRALCVPLNTFVAIKVLDLEKCSSDLDGIRREVQTMSLLDHPNLLRACCSFANDHQLWVVMPFMAAGSALHIIKTNFPDGFEEAVIATLLWEVLKALVYLHSQGHIHRDVKAGNILIDTNGAVKLGDFGVSACMFDTGNRQRARNTFVGTPCWMAPEVMQQLHGYDYKADIWSFGITALELAHGHAPFSKYPPMKVLLMTLQNAPPGLDYERDKRFSKSFRDLVAVCLVKDPQKRPSSEKLLKHSFFKQARSADFLAKSILEGLTPLGDRFRALKAKEADLLLNNKLGPESKEQLSQKEYIRGISGWNFNLEDLKTAAALLDSSNGTYHFDGANNKDRNGLQDVYNESENIYQERVNHGASARHDEHEIQEVEDLDGDLASSFPTRPLEALKSCFDVGGDDDPDPTATNLPVQPSMESISPVQQFSEMDHSRSDNCNGENLERSVSVPSNLGNSVYPKFSSGSLIPEHVLSPYKNVGSDSRRNEFHQKNPSSRNRSGPLFFRQMKDTRPHLSVAPDEASEGNVVQRRGRFQVTSDNPGQKVASSASSNSRPNLPSGVTRPASNSSTILPTLQFLMQQNSMQKEVLSRLISSIEETSDASDASTVGLSQSFGSLAREKGLESYVVQLQRSVTELSEEVQRLKLRNNQLEQQINGLSKKDERLRREGSTKQ*

>TraesCS7B02G130700.1

MERAPRERGFPTDPKEYKLYEEVGEGVSATVYRALCVPLNTFVAIKVLDLEKCSSDLDGIRREVQTMSLIDHPNLLRACCSFANDHQLWVVMPFMAAGSALHIIKTNFPDGFEEAVIATLLWEVLKALVYLHSQGHIHRDVKAGNILIDTNGAVKLGDFGVSACMFDTGNRQRARNTFVGTPCWMAPEVMQQLHGYDYKADIWSFGITALELAHGHAPFSKYPPMKVLLMTLQNAPPGLDYERDKRFSKSFRDLVAACLVKDPQKRPSSEKLLKHSFFKQARSADFLAKSILEGLTPLGDRFRALKAKEADLLLNNKLGPESKEQLSQKEYIRGISGWNFNLEDLKTAAALLDSSNGTYHFDGANNKDRDGLQDVYNESENIYQERVNHGASARHDEHEIQEVEDLDGDLASSFPTRPLEALKSCFDVGGDDDPDPTATNLRAQPSMESISPVQQFSEMDHSRSDNCNGENLERSVSVPSNLGNSVYPKFSSGSLIPEHVLSPYKNVGSDSRRNEFHQRNPSSRNRSGPLFFRQMKDTRPHLSVAPDEASEGNVVQRRGRFQVTSDNPGQKVASSASSNSRPNLPSGVTRPASNSSTILPTLQFLMQQNSMQKEVLSRLISSIEETSDASDASTVGSSQSSGSLAREKGLESYVVQLQRSVTELAEEVQRLKLRNNQLEQQINGLSKKDERLRREGSTKQ*

>TraesCS7D02G232400.1

MERAPRKRGFPTDPKEYKLYEEVGEGVSATVYRALCVPLNTFVAIKVLDLEKCSSDLDGIRREVQTMSLIDHPNLLRACCSFANDHQLWVVMPFMAAGSALHIIKTNFPDGFEEAVIATLLWEVLKALVYLHSQGHIHRDVKAGNILIDTNGAVKLGDFGVSACMFDTGNRQRARNTFVGTPCWMAPEVMQQLHGYDYKADIWSFGITALELAHGHAPFSKYPPMKVLLMTLQNAPPGLDYERDKRFSKSFRDLVAVCLVKDPQKRPSSEKLLKHSFFKQARSADFLAKSILEGLTPLGDRFRALKAKEADLLLNNKLGPESKEQLSQKEYIRGISGWNFNLEDLKTAAALLDSSNGTYHFDGANNKDRNGLQDVYNESENIYQERVNHGASARHDEHEIQEVEDLDGDLASSFPTRPLEALKSCFDVGGDDDPDPTATNLRVQPSMESISPVQQFSEMDHSRSDNCNGENLERSVSVPSNLGNSVYPKFSSGSLIPEHVLSPYKNVGSDSRRNDFHQKNPSSRNRSGPLFFRQMKDIRPHLSVAPDEASEGNVVQRRGRFQVTSDNPGQKVASSASSNSRPNLPSGVTRPASNSSTILPTLQFLMQQNSMQKEVLSRLISSIEETSDVSDASTVGLSQSSGSLAREKGLESYVVQLQRSVTELAEEVQRLKLRNNQVVVSIVIVSILASSHYSIIFDLYSMLCVIGED*

>TraesCS1D02G185000.1

MGRNGSVKRGAAAAAPPSFTVNPADYRLMEEVGYGAHAVVYRALFLPRNQTVAVKCLDLDQLNNNIDEVQREAQIMSLIDHPNVIRAYCSFVVEHSLWVIMPFMTEGSCLHLMKIAYPEGFEEPVIASILKETLKALEYLHRQGQIHRDIKAGNILIDSAGVVKLGDFGVSACMFDRGDRQRSRNTFVGTPCWMAPEVLQPGTGYNFKADIWSFGITALELAHGHAPFSKYPPMKVLLMTLQNAPPGLDYDRDRRFSKAFKEMVAMCLVKDQTKRPTAEKLLKHSFFKTAKPPESTMKGMLTDLPPLWERVKALQLKDAAQLALKKMPSSEQEALSLSEYQRGVSAWHFDIEDLKAQASLIYEDEPSETKEDDVAARIIESEKSLYSRTPSGQSSSANENTCSEETSTTNPDCRRMPNGHENSRSENDSLPSTSREPESKYWRTNVGQKQQTSGGPVEGGVNSSTTERSHNFERDATADKHGSDTRRAANLSGPLSLPTRASANSYSAPIRSSGGYVDSLGDKSKRSVVHIKGRFSVTSENVDLAKVQEIPLSSISRKSPQGIQLRKSASVGDWIVNAKPMSNSHHLKELCNSSVSSSVLIPHLENLVQQTTLQQVWRKLEHV*

>TraesCS5A02G556400.1

MVRGGSMRRPSLAAVSEFTLSADDYRLMEEVGFGANAVVYRAIFLPANRTIAVKCLHLDRISSNLDDVRKEAQIMKLIDHPNVIKAYCSFVVDHNLWVIMPFMAEGSCLHLMKVAHPDGLEEPVICSILKETLKALAYLHGQGHIHRDVKAGNILVDSPGVVKLGDFGVSACLFDRGDRQRSRNTFVGTPCWMAPEVLQPGTGYNFKADIWSFGITALELAHGHAPFSKYPPMKVLLMTLQNAPPGLDYDRDRKFSKSFKEMVAMCLVKDQTKRPTAEKLLKHSFFKNTKAPQLTVKSILTDLPPLWDRVKALQQKDAAHLASSEQEALSMSEYQRGVSAWHFDIEDLKAQALLINDDDPPELKEDDDSVRINEVDKGTSFESHFGQSTLLNGNTHRLNHERTCTTAVNPGGNGPETSDEFASDLGNADSPRMVDGHIKQGTENDSLSSTSKQGSEGGNRRSEVRQRQRTFSGPVMYSGTRSSSLIERGYIIDKDAGVRSPNKQKSDTGRIDDLSGPLSLSTRASANSLSAPIRSSGGYVGSLGDKPRVEIKGRFSVTSENVDLAKVQEIPVVKISPKPQEVRTQVSTMKRSASVGAWPVKAKSMSNSHHRKEFRDSSVSASILIPHLENLVQQTTFQQDIITNLMSNLQQNEKPNGPQTRVQTMVGDTGVEMGSAERERKLLAKVFELQSRMISLTDELIASKLKHVQLQEELNTLYCQEETGDTGEA*

>TraesCS6A02G149900.1

MGSKEKGKRGDGGASPRARKGEFPIRAEDYELMEPIGDGATAVVRRARCLPLGGEVVAVKIMNLSLRSEADVNNASEEVKTMILTDHPNLLSAYCSFTQDENLWIVMPYMAGGSCFHLMKSSFPKGFEEERFIAFVLRETLRGLEYLHGKGHIHRDVKAGNILLDQHKGVKLADFGISASVYDSMINRNGKRHTLVGTPCWMAPEVMEQKEYDFKADIWSFGITALELANGHAPFSSQPPAKVFLMTLQHAPPSLHNTKDKKFSNSFKRMIGACLIKDPSKRPTAQMLLQLPFFKKVKSEDNHVKCMLNKVPSLVARVQTINENEAKLQAEKKPHDKIKEKTSHDEYWRGISQWHFDIEDLKAQAKLYSEENDSDEEEYLRFLFELDTVDEIVPLQDVRPQNHASDDKKIVGTEIVETPSSTTPILIPQSGKQLENGPPNGLARHESFERHSKVPTKQLSRAVSNVTCMDEYVEKTAIQKGRFKVTTEETEASTHREKELLERIACLERMLQVTQDEVVRLKEKGEAKGAVPCVQQNSKVQGL*

>TraesCS6B02G386100.1

MVGSGSKHAAGGSGGAGGGERRKYPIRAEDYELYEEIGQGVSAIVYRALCRPLGETVAVKVLDFERTNSNLNNIMREAQTMILIDHPNVVKAICSFANNQTLWVVMPYMAGGSCLHIMKSVYPDGFEEAVIATLLREVLRGLEYLHHHGHIHRDVKAGNILVDSRGGVKLGDFGVSACLFESGDRQRARNTFVGTPCWMAPEVMEQLHGYDFKADIWSFGITALELAHGHAPFSKYPPMKVLLMTLQNAPPGLDYERDKKFSRHFKQMVAMCLVKEPSKRPTATKLLKQSFFKQARSHDYIVRKLLEGLPGLGARYQALKEKDEHLLAQKKMPDGRKEEISQDEYKRGISSWNFDIDDLKSQASLISECEDTISSKDTDISSIYDFDTSLQEQAHEGSLFSMKYDTDIENDVMANDKSAVSSPEQSVCLSRASLCGTSNGVLVNGHVRKHSSMESCDLDLQEKDLDAIPTSSFQERKCSFSSCSSDGFLSSKESSKPQINIHNREKGSGGVLQVADEPSPEAISRAPKSLVSNVDEHDDRSKPPLIQQRGRFKVTPGNVELDKAHSPGLQKSHSMQTISQLSALSIPSSAEAASSIIGGSLYIQLYNVLQTNLLQREQILHAMKQLYISDSISPVRMHSLSRSPSPSSALSVDRSMLEAAQEKEKELVNEVLELQWRLLCAQDEVQRLKAKAAQI*

>TraesCS6D02G139200.1

MEPIGDGATAVVRRARCLPLGGEVVAVKIMNLSLRSEADVNNASEEVKTMILTDHPNLLSAYCSFTQDENLWIVMPYMAGGSCFHLMKSSFPKGFEEERFIAFVLRETLRGLEYLHGKGHIHRDVKAGNILLDQHKGVKLADFGISASVYDSMINRNGKRHTLVGTPCWMAPEVMEQKEYDFKADIWSFGITALELANGHAPFSSQPPAKVFLMTLQHAPPSLHNTKDKKFSNSFKRMIGACLIKDPSKRPTAQMLLQLPFFKKVKSEDNHVRCMLNKVPSLVARVQTIKENEAKLQAEKKPHDKIKEKTSHDEYWRGISQWHFDIEDLKAQAKLYSEENDSDEEEYLRFLFELDTVDEIVPLQDVRPQNHASDDKKIVGTEIVETPSSTTPILIPQSGKQLENGPPNGLARHESFERHSKVPTKQLSRAVSNVTCMDEHIEKTAIQKGRFKVTTEETEASTHREKELLERIACLERMLQVTQDEVVRLKEKEAKGAVPCVQQNKVHGL*

>TraesCSU02G115300.1

MVRGGSMRRPSLALAAVSEPAAPEFTLSADDYRLMEEVGFGANAVVYRAIFLPANRTIAVKCLDLDRINSNLDDVRKEAQIMKLIDHPNVIKAYCSFVVDHNLWVIMPFMAEGSCLHLMKVAHPDGLEEPVICSILKETLKALAYLHGQGHIHRDVKAGNILVDSPGVVKLGDFGVSACLFDRGDRQRSRNTFVGTPCWMAPEVLQPGTGYNFKADIWSFGITALELAHGHAPFSKYPPMKVLLMTLQNAPPGLDYDRDRKFSKSFKEMVAMCLVKDQTKRPTAEKLLKHSFFKNTKAPQLTVKSILTDLPPLWDRVKALQQKDAAHLASSEQEALSMSEYQRGVSAWHFDIEDLKAQALLINDDDPTELKEDDDSARINEVDKGTSFESHFGQSMLLNGNNHRLNHERTCTTAVNPGGNGPETSDEFASDLGNADSPRMVDGHIKQGTENDSLSSTSKQGSEGGNRRSEVRQRQRTFSGPVMYSGTRSSSLIERGYIIDKDAGARSPNKQKSDTGRIDDLSGPLSLSTRASANSLSAPIRSSGGYVGSLGDKPRVEIKGRFSVTSENVDLAKVQEIPVVKISPKPQEVRTQVSTMKRSASVGAWPVKAKSMSNSHHRKEFRDSSVSASILIPHLENLVQQTTFQQDIITNLMSNLQQNEKPNGPQTRVQTMVGDTGVETGSAERERKLLAKVFELQSRMISLTDELIASKLKHVQLQEELNTHYCQEETGDTGEDDSGEA*

>TCA.XM_007045769.1

MDYPPRRNRKAAVKSELYSTVVIHSGSESESDSDSTRSKRKPRPPREQDPYATMLYKDGDEEDEDDSSLPPLLKRLPKDFGGGGGGPTDFDVDDDEDAGGFGTMIVKTDRRRNTRGQTSSSFKPPEAAVSPMMARREEMDDDDEEDVDGDGEGFGTFVVRSTVRSDREGSGTVVSRAVASMGELGFGKQKRSTSSASLQGEENRFSQNSKVSSSSIPDSLTREDPSTKYELLNELGKGSYGAVYKARDIRTSELVAIKVISLSEGEEGYEEIRGEIEMLQQCSHPNVVRYLGSYQGEEYLWIVMEYCGGGSVADLMNVTEEPLEEYQIAYICREALKGLEYLHSIFKVHRDIKGGNILLTEQGEVKLGDFGVAAQLTRTMSKRNTFIGTPHWMAPEVIQESRYDGKVDVWALGVSAVEMAEGLPPRSAVHPMRVLFMISIEPAPMLEDKEKWSLVFHDFVAKSLTKDPRLRPTASEMLKHKFIEKCKCGASVMFPKIEKAKQIRAAMVQEAQTLAPTISRVNPPEGSKLNEDYGDTVPSRPQNMGLEVANEAPATGTLKKHHILDGVKVTGEGEFGTVIVHGGDEVQKSFAQSQLQSGKAASTALEHVESTLINGTGRQLAESWVDNRRGGSANNTTMASISVPPEQKLRSDSVLQAQAEGGSEISGSTLKNETVSKKAFALQDKLSSIYAAGNTVPIPFLRATDISPIALLSDNVLGGMHQDSSGTVAVEAVQELFAGDGQLKKGRRAQNEFNMLQMPLPPSVYQRLTSSSTLMNLAHALAYHKMCYDEMPLQELKATQEQQTIQNLCDTLRTILRL*

>TCA.XM_007039347.1

MRRMGGSQRSYSANPSDYRLLEEVGYGASATVYRAIYLPFNDVVAVKCLDLDRCSSNLDDIRREAQTMSLIDHPNVIRALCSFVVDRNLWVVMPFMSEGSCLHLMKIAYPDGFEEPAIGSILKETLKALDYLHRQGHIHRDVKAGNILLDNNGTVKLADFGVSACMFDAGDRQRSRNTFVGTPCWMAPEVLQPGSGYNSKADIWSFGITALELAHGHAPFSKYPPMKVLLMTIQNAPPGLDYDRDKKFSKSFKEMVAMCLVKDQTKRPTAEKLLKHSFFKHAKPPELSVKKLFADLPPLWNRVKALQLKDAAQLALKKMPSAEQEAISQSEYQRGVSAWNFDIGDLKAQASLVRDDDDIHECKDDDASMKSSLGHKAAAYCESSLGKLNLNREVSQAETGGPRNVDLSQSDCLNGKGKNLECDIVEAGCQETVGLRKNGSSIDVMASTSERDVVLTRAKTVKPRQTQSGPLTPGAVLNHSSSERVRNSERFENEILPANEKVCQVRKAPSFSGPLMLPNRASANSLSAPIKSSGGFRDSLDDKSKANLVQIKGRFSVTSENLDLVKDIPLSTVSRRSSQQTSPLRKSASVGDWIFESKQVPTNHSSKDLTNGNMPTSIFMNHLQNLFQQTSLQQDLIVNLLNIFQPAEFVDATQNGKLPPLPRCSESNGNVETAASERERLLLCKISELQSRMMNLTDELTAEKLKYEQLQQQLRSMSGAEENGIRLEGDA*

>TCA.XM_007031422.1

MEHPSEKRYPVNAIDYKLYEEVGEGVSATVCRALCIPLNEIVAIKVLDLEKCNNDLDGIRREVQTMSLIDHPNVLRAHCSFTAGHNLWVVMPYMAGGSCLHIMKSVYSEGFEEPVIATLLREVLKALVYLHAHGHIHRDVKAGNILIDSNGSVKLADFGVSACMFDTGDRQRSRNTFVGTPCWMAPEVMQQLHGYDFKADIWSFGITALELAHGHAPFSKYPPMKVLLMTLQNAPPGLDYERDKRFSKSFKELVATCLVKDPKKRPTSEKLLKHHFFKHARPHDYLARSILDGLAPLGERFRVLKAKEADLLVQNKALYEDKEQLSQQEYIRGISAWNFNLEDLKSQAALIQDYDDVSSAEDRDGSRKQRDRHDDVGLPAERMSPEMASNSIAATSQEDGLSDLHDLESSLVSFPIKPLQALKGCFDIGEDDEGANSPNWKGATRSESEQIITKSSRAADQDAGRNEGENSGQSSSLPRQVIPEHKKFLSGSLIPDNAFSPKKVTGDGDRDFPQPKFPSDRNYSGPLSYRHRRETNNISSEDASEGAVVQRGRFKVTSADLSPKGPTNCNFNPAIGGSTCPSSLNLTASAVLPSLQCILQQNTVQREEIIRLIKYLEQTYGKPGDLTEVGTNDLLQIPHSSLRERELQSQVIQLQQSIGNLVEELQRQKMRNMQLEKQLSALANNKE*

>TCA.XM_007034971.1

MAHEQEHHPKLQFPLESEAYKIIDEIGVGVSAVVYKAECTPMNSTVVAIKSIDLDQSKDFDNIRRETKILSLLSHPNILNAHCSFTVDQRLWVVMPFMSGGSLQSIISSSFPDGLPEQCIAIVLKETLNALSYLHNQGHLHRDIKAGNILMDSNGCVKLADFGVSASIYESNPGYGSGSSTSSSPLMLNDVTGTPYWMAPEVIHSHTGYSFKADIWSFGITALELAHGRPPLSHLPLSKSLIMKITKRFRFSDYESNSKEGKSKKFSKAFKDMVASCLDQDPAKRPSAEKLLKHSFFKSCKGSEFLVKNVLHGLSSVEERFRVSKILGKGVHVEADDDDVDGESVSQLVKYRRISGWNFNEDGFELEPVFPDESKEDSIVKQVRFGGEMIIPGTVGEADGLSNSGRINLNSPETESSLLSSRGEGGDEASSPSSWRVGGKEESGIEGIVGDEAGIEGTVNTDIMVGGLTALIRSLDDQRQRVTDLITLFGGDVTSREDQLLQLIERLRLDLENERQKNFQLEMELEFLKIHISGASNTDENN*

>TCA.XM_007024752.1

MEKKKYPIGPEYYTLHEEVGQGVSASVHRAVCIPFNEIVAIKILDFERDNCDLNNISREAQTMILVDHPNVLKSHCSFVSDHNLWVVMPYMAGGSCLHILKAAYPDGFEEVVIATVLREVLKGLEYLHHHGHIHRDVKAGNILIDSRGAIKLGDFGVSACIFDSGDRQRMRNTFVGTPCWMAPEVMEQLHGYDFKADIWSFGITALELAHGHAPFSKYPPMKVLLMTLQNAPPGLDYERDRKFSKSFKQMIASCLVKDPLKRPSAKKLLKHSFFKQARSNDYIARTLLDGLPALGDRIQALKRKEEDMLAQKKMPDGEKEELSQNEYKRGISGWNFNLEDMKAQASLIQDEDLVSDTNQGGSSSSLSTLDGQDKQSECQTSSQPLDKEDNDPVQNQPTPVAAVEPTINIAKVRFERSDDDSSVASPSHEHHAISPHHDDHVESNLGEKSVLEINGKSSDNMSKPFYQRTTSFSGSTSIPETIVPPIKGESDKQNQPQNIFVGNGAAVPAGGEDSISDLHSKASKSSAVNSDDLDEKAKPPVVQQRGRFKVTSENVDLEKVAPAPILQKSHSMQVGTLEVLTPHPVVSLAPPPSDAASSTLAAHHLFPLLQSVLQTNILQRENILNLIKHISAVDSTVNRAFEGVCTPANVAVTEKSLLEAAHDRERELLHEITELQWRLICAQEELQKYKTENAQV*

>TCA.XM_007021490.1

MADIAGLQEAAGSRFSQLELIGRGSFGDVYKAFDKELNKEVAIKVIDLEESEDEIEDIQKEISVLSQCRSQYITEYYGSYLHQTKLWIIMEYMAGGSVADLLQSGPPLDEMSIACISRDLLHAIEYLHNEGKIHRDIKAANILLTENGDVKVADFGVSAQLTRTISRRKTFVGTPFWMAPEVIQNSEGYNEKADIWSLGITVIEMAKGEPPLADLHPMRVLFIIPRENPPQLDEHFSRPMKEFVSLCLKKVPAERPSAKELLKHRFIRNARKSQRLLERIRERPKYQLKEDAETPRNGPKAVGESTDTVKVTRDIRGEETVRASKQGKTFKNAGWDFSIGGEQSTGTVRNAVRPPQVRERKLEAVYNQATPRTPESVNALNEFPEVSFGKDTKKSYYDEHQDNYQEDDDTSVSGSGTVVIRSPRGSQSSALFRDPSSLSSSAYASFEDASTSGTVVFRGQHDDSDSPRTPRSRLGIQERTSSASAEDSAANLAEAKAAIQAGLRKSNVRDRPALSKFNSPGHENRRRDQMSNSSDSSRGSREYFDAQRVFPRSRQPSDDEENAKIASSSVSLSMLLIPSLKEVVADDSEGSVARAVTNSLINMERTKPGSCETLVRRLLERLASLKEPSMKDIQELAARMFNKGKATPEDTQNANMEPDSRKKQQHKELNSNSNLSPLARFLLSRWQSQTSRDLNPT*

>TCA.XM_007021757.1

MGRMQGTRSYPANPSDYQLLEEIGHGATATVYKAIYLPSKDVVAVKCLDLDRCSGTNLDDVRREAQTLRLIDHPNVLRAYSSFVVDRNLWVVMPFMSEGSCLHRMKTGYPDGFEEAAIASILKETLKALDYLHRQGHIHRDVKAGNILLDNNGTVKLADFGVSACMFDSGDRQRSRNTFVGTPCWMAPEVMQPGTGYNSKADIWSFGITALELAHGHAPFSNYPPMKVLLMTIQNAPPRLDQDCDRKFSKSFKDMVAMCLVKDHTKRPTAEKLLKHSFFKHAKPPELSVKKLFAPLQPLSNPAKPLQLKDAAQLALKNMPSAEQEAISRSQYQRGVSAWNFDIEDLKAQASLVHDDDDDDDDIHECKDDDRSMKSSLGDKTAASCISSSSIVLSNDLFFSDDDFFIMPATCASSETRENALGRKQLNKAETEKRAIGMRVLNVDLLN*

>TCA.XM_007021772.1

MGKMQGTRSHSANPSDYQLLEEIGHGATATVYKAIYLPSKDVVAVKCLDLDRCSGTNLDDVRREAQTLRLIDHPNVLRAYSSFVVDRNLWVVMPFMSKGSCLHRMKTAYPDGFEEAAIASILKETLKALDYLHRQGHIHRDVKAGNILLDNNGTVKLADFGVSACMFDSGDRQRSRNTFVGTPCWMAPEVMQPGTGYNSKADIWSFGITALELAHGHAPFSNYPPMKVLLMTIQNAPPRLDQDCDKKFSKFFKDMVAMCLVKDQTKRPTAEKLLKHSFFKHAKPPELSVKKLFAPMPPLCNPVKPLQLQEAAQQEATSQSQYQRGISAWNFDIEDLKAQASLVHDDDDIHGGKDDDRSMKASLGDKKLIQPPHDPMLVL*

>TCA.XM_007021774.1

MGRMGGTRSYSANPSDYQLLEEIGHGATATVYKAIYLPSKDVLAVKCLDLDRCSGTNLDDVRREAQTLRLIDHPNVLRAYSSFVVDRNLWVVMPFMSEGSCLHRMKTAYPDGFEEAAIASILKETLKALDYLHRQGHIHRDVKAGNILLDNKGTVKLADFGVSACMFDSGDRQRSRNTFVGTPCWMAPEVMQSGTGYNSKADIWSFGITALELAHGHAPFSKYPPMKVLLMTIQNAPPRLDQDCDKKFSKSFKDMVAMCLVKDQTKRPTAEKLLKHSFFKHAKPPELSVKKLFAPLEPLSNPAKPLQLKDAAQLALKNMPSADQEATSRSQYQRGVSAWNFDIEDLKAQASLVHDDDDDGDIHECKDDDRSTKSSLGDKTAASCISSSIVLSNDLCKSWVIIPA*

>TCA.XM_007021777.1

MSFGSGCYSLVGNNLETLLLTNNLLTGIIPASLSNCKISLGCHFLSIRIPPELGSCQSLLWLDLNNNHLTGNIPSDLANQVGLVFENNLKLYKPHPLKSQSSSSRSVTLIMGGTRSYSANPRDYQLLEEIGHGATATVYKAIYLPSKDVVAVKCLDLDRCSGTNLDDVRREAQTMRLIDHPNVLRAYSSFVVDRNLWVVMPFMSEGSCLHRMKTAYPDGFEEAAIASILKETLKALDYLHRQGHIHRDVKAGNILLDNNGTVKLADFGVSACMFDSGDRQRSRNTFVGTPCWMAPEVMQSGTGYNSKADIWSFGITALELAHGHAPFSKYPPMKVLLMTIQNAPPRLDQDRDKKFSKSFKDMVAMCLVKDQTKRPTAEKLLKHSFFKHAKRPELSVKKLFTSLPRLSNPAKPLQLNDAGQLALKNMPSAEQEATSRSQYQRGVSAWNFDIEDLKAQASLVHDDDDIHECKDADRSMKSSLGDKTAASCISSSCIVLSNDQCKSWVIIPA*

>TCA.XM_007021791.1

MQGTRSYSANPSDYQLLEEIGHGATATVYKAIYLPSKDVVAVKCLDLDRCSGTNLDDVRREAQTLRLIDHPNVLRAYASFVVDRNLWVVMPFMSEGSCLHRMKTAYPDGFEEAAIASILKETLKALDYLHRQGHIHRDVKAGNILLDNNGTVKLADFGVSACMFDSGDRQRSRNTFVGTPCWMAPEVMQPGTGYNSKADIWSFGITALELAHGHAPFSKYPPMKVLLMTIQNAPPRLDQDCDKKFSKSFKDMVAMCLVKDQTKRPTAEKLLKHSFFKHAKPPELFVKKLFAPFPRLSNPVKPVQLKDAAQLALKKMPSAEQAATSQSQYQRGISAWNFDIEDLKAQASLVHDDDDIHECEDDDRSMKSNLGDKTAAYCSSSSPIVLSKMMYVRAGA*

>TCA.XM_007021794.1

MGRMEGTRSYSANPSDYQLLEEIGHGATATVYKAIYLPSKDVVAVKSLDLERCNGNNLDDVRREAQTMSLIDHPNVLRAYSSFVVDHNLWVVMPFMSEGSCLHRMKTAYPDGFEEAAIASILKEILKALDYLHRQGHIHRDVKAGNILLDNNGTVKLADFGVSACMFDSGDRQRSRNTFVGTPCWMAPEVLQSGTGYNSKADIWSFGITALELAHGHAPFSKYPPMKVLLMTIQNAPPRLDQDRDKKFSKSFKDMVAMCLVKDQTKRPTAEKLLKHSFFKHANPSELSVKKLFAPMPPLCNPVKPLQLKDAAEQEAASQSQYQRGISAWNFDIDDLKAQASLVHDDDDIHEVQR*

>TCA.XM_007045768.1

MDYPPRRNRKAAVKSELYSTVVIHSGSESESDSDSTRSKRKPRPPREQDPYATMLYKDGDEEDEDDSSLPPLLKRLPKDFGGGGGGPTDFDVDDDEDAGGFGTMIVKTDRRRNTRGQTSSSFKPPEAAVSPMMARREEMDDDDEEDVDGDGEGFGTFVVRSTVRSDREGSGTVVSRAVASMGELGFGKQKRSTSSASLQGEENRFSQNSKVSSSSIPDSLTREDPSTKYELLNELGKGSYGAVYKARDIRTSELVAIKVISLSEGEEGYEEIRGEIEMLQQCSHPNVVRYLGSYQGEEYLWIVMEYCGGGSVADLMNVTEEPLEEYQIAYICREALKGLEYLHSIFKVHRDIKGGNILLTEQGEVKLGDFGVAAQLTRTMSKRNTFIGTPHWMAPEVIQESRYDGKVDVWALGVSAVEMAEGLPPRSAVHPMRVLFMISIEPAPMLEDKEKWSLVFHDFVAKSLTKDPRLRPTASEMLKHKFIEKCKCGASVMFPKIEKAKQIRAAMVQEAQTLAPTISRVNPPEGSKLNEDYGDTVPSRPQNMGLEVANEAPATGTLKKHHILDGVKVTGEGEFGTVIVHGGDEVQKSFAQSQLQSGKAASTALEHVESTLINGTGRQLAESWVDNRRGGSANNTTMASISVPPEQKLRSDSVLQAQAEGGSEISGSTLKNETVSKKAFALQDKLSSIYAAGNTVPIPFLRATDISPIALLSDNVLGGMHQDSSGTVAVEAVQELFAGDGQLKKGRRAQNEMPLPPSVYQRLTSSSTLMNLAHALAYHKMCYDEMPLQELKATQEQQTIQNLCDTLRTILRL*

>TCA.XM_007039346.1

MRRMGGSQRSYSANPSDYRLLEEVGYGASATVYRAIYLPFNDVVAVKCLDLDRCSSNLDDIRREAQTMSLIDHPNVIRALCSFVVDRNLWVVMPFMSEGSCLHLMKIAYPDGFEEPAIGSILKETLKALDYLHRQGHIHRDVKAGNILLDNNGTVKLADFGVSACMFDAGDRQRSRNTFVGTPCWMAPEVLQPGSGYNSKADIWSFGITALELAHGHAPFSKYPPMKVLLMTIQNAPPGLDYDRDKKFSKSFKEMVAMCLVKDQTKRPTAEKLLKHSFFKHAKPPELSVKKLFADLPPLWNRVKALQLKDAAQLALKKMPSAEQEAISQSEYQRGVSAWNFDIGDLKAQASLVRDDDDIHECKDDDASMKSSLGHKAAAYCESSLGKLNLNREVSQAETGGPRNVDLSQSDCLNGKGKNLECDIVEAGCQETVGLRKNGSSIDVMASTSERDVVLTRAKTVKPRQTQSGPLTPGAVLNHSSSERVRNSERFENEILPANEKVCQVRKAPSFSGPLMLPNRASANSLSAPIKSSGGFRDSLDDKSKANLVQIKGRFSVTSENLDLVKDIPLSTVSRRSSQTSPLRKSASVGDWIFESKQVPTNHSSKDLTNGNMPTSIFMNHLQNLFQQTSLQQDLIVNLLNIFQPAEFVDATQNGKLPPLPRCSESNGNVETAASERERLLLCKISELQSRMMNLTDELTAEKLKYEQLQQQLRSMSGAEENGIRLEGDA*

>TCA.XM_007039348.1

MRRMGGSQRSYSANPSDYRLLEEVGYGASATVYRAIYLPFNDVVAVKCLDLDRCSSNLDDIRREAQTMSLIDHPNVIRALCSFVVDRNLWVVMPFMSEGSCLHLMKIAYPDGFEEPAIGSILKETLKALDYLHRQGHIHRDVKAGNILLDNNGTVKLADFGVSACMFDAGDRQRSRNTFVGTPCWMAPEVLQPGSGYNSKADIWSFGITALELAHGHAPFSKYPPMKVLLMTIQNAPPGLDYDRDKKFSKSFKEMVAMCLVKDQTKRPTAEKLLKHSFFKHAKPPELSVKKLFADLPPLWNRVKALQLKDAAQLALKKMPSAEQEAISQSEYQRGVSAWNFDIGDLKAQASLVRDDDDIHECKDDDASMKSSLGHKAAAYCESSLGKLNLNREVSQAETGGPRNVDLSQSDCLNGKGKNLECDIVEAGCQETVGLRKNGSSIDVMASTSERDVVLTRAKTVKPRQTQSGPLTPGAVLNHSSSERV*

>TCA.XM_007031421.1

MEHPSEKRYPVNAIDYKLYEEVGEGVSATVCRALCIPLNEIVAIKVLDLEKCNNDLDGIRREVQTMSLIDHPNVLRAHCSFTAGHNLWVVMPYMAGGSCLHIMKSVYSEGFEEPVIATLLREVLKALVYLHAHGHIHRDVKAGNILIDSNGSVKLADFGVSACMFDTGDRQRSRNTFVGTPCWMAPEVMQQLHGYDFKADIWSFGITALELAHGHAPFSKYPPMKVLLMTLQNAPPGLDYERDKRFSKSFKELVATCLVKDPKKRPTSEKLLKHHFFKHARPHDYLARSILDGLAPLGERFRVLKAKEADLLVQNKALYEDKEQLSQQEYIRGISAWNFNLEDLKSQAALIQDYDDVSSAEDRDGSRKQRDRHDDVGLPAERMSPEMASNSIAATSQEDGLSDLHDLESSLVSFPIKPLQALKGCFDIGEDDEGANSPNWKGATRSESEQIITKSSRAADQDAGRNEGENSGQSSSLPRQVIPEHKKFLSGSLIPDNAFSPKKVTGDGDRDFPQPKFPSDRNYSGPLSYRHRRETNNISSEDASEGAVVQRGRFKVTSADLSPKGPTNCNFNPAIGGSTCPSSLNLTASAVLPSLQCILQQNTVQREEIIRLIKYLEQTYGKPGDLTEVGTNDLLQIPHSSLRERELQSQVIQLQQSIGNLVEELQRQKMRNMQLEKQLSALANNKE*

>TCA.XM_007024753.1

MEKKKYPIGPEYYTLHEEVGQGVSASVHRAVCIPFNEIVAIKILDFERDNCDLNNISREAQTMILVDHPNVLKSHCSFVSDHNLWVVMPYMAGGSCLHILKAAYPDGFEEVVIATVLREVLKGLEYLHHHGHIHRDVKAGNILIDSRGAIKLGDFGVSACIFDSGDRQRMRNTFVGTPCWMAPEVMEQLHGYDFKADIWSFGITALELAHGHAPFSKYPPMKVLLMTLQNAPPGLDYERDRKFSKSFKQMIASCLVKDPLKRPSAKKLLKHSFFKQARSNDYIARTLLDGLPALGDRIQALKRKEEDMLAQKKMPDGEKEELSQNEYKRGISGWNFNLEDMKAQASLIQDEDLVSDTNQGGSSSSLSTLDGQDKQSECQTSSQPLDKEDNDPVQNQPTPVAAVEPTINIAKVRFERSDDDSSVASPSHEHHAISPHHDDHVESNLGEKSVLEINGKSSDNMSKPFYQRTTSFSGSTSIPETIVPPIKGESDKQNQPQNIFVGNGAAVPAGGEDSISDLHSKASKSSAVNSDDLDEKAKPPVVQQRGRFKVTSENVDLEKVAPAPILQKSHSMQVLTPHPVVSLAPPPSDAASSTLAAHHLFPLLQSVLQTNILQRENILNLIKHISAVDSTVNRAFEGVCTPANVAVTEKSLLEAAHDRERELLHEITELQWRLICAQEELQKYKTENAQV*

>TCA.XM_007024754.1

MEKKKYPIGPEYYTLHEEVGQGVSASVHRAVCIPFNEIVAIKILDFERDNCDLNNISREAQTMILVDHPNVLKSHCSFVSDHNLWVVMPYMAGGSCLHILKAAYPDGFEEVVIATVLREVLKGLEYLHHHGHIHRDVKAGNILIDSRGAIKLGDFGVSACIFDSGDRQRMRNTFVGTPCWMAPEVMEQLHGYDFKADIWSFGITALELAHGHAPFSKYPPMKVLLMTLQNAPPGLDYERDRKFSKSFKQMIASCLVKDPLKRPSAKKLLKHSFFKQARSNDYIARTLLDGLPALGDRIQALKRKEEDMLAQKKMPDGEKEELSQNEYKRGISGWNFNLEDMKAQASLIQDEDLVSDTNQGGSSSSLSTLDGQDKQSECQTSSQPLDKEDNDPVQNQPTPVAAVEPTINIAKVRFERSDDDSSVASPSHEHHAISPHHDDHVESNLGEKSVLEINGKSSDNMSKPFYQRTTSFSGSTSIPETIVPPIKGESDKQNQPQNIFVGNGAAVPAGGEDSISDLHSKASKSSAVNSDDLDEKAKPPVVQQRGRFKVTSENVDLEKVAPAPILQKSHSMQVGTLEVLTPHPVVSLAPPPSDAASSTLAAHHLFPLLQSVLQTNILQRENILNLIKHISAVDSTVNRAFEGVCTPANVAVTEKSLLEAAHDRERELLHEITELQWRLICAQEELQKYKTENAQV*

>TCA.XM_007024755.1

MEKKKYPIGPEYYTLHEEVGQGVSASVHRAVCIPFNEIVAIKILDFERDNCDLNNISREAQTMILVDHPNVLKSHCSFVSDHNLWVVMPYMAGGSCLHILKAAYPDGFEEVVIATVLREVLKGLEYLHHHGHIHRDVKAGNILIDSRGAIKLGDFGVSACIFDSGDRQRMRNTFVGTPCWMAPEVMEQLHGYDFKADIWSFGITALELAHGHAPFSKYPPMKVLLMTLQNAPPGLDYERDRKFSKSFKQMIASCLVKDPLKRPSAKKLLKHSFFKQARSNDYIARTLLDGLPALGDRIQALKRKEEDMLAQKKMPDGEKEELSQNEYKRGISGWNFNLEDMKAQASLIQDEDLVSDTNQGGSSSSLSTLDGQDKQSECQTSSQPLDKEDNDPVQNQPTPVAAVEPTINIAKVRFERSDDDSSVASPSHEHHAISPHHDDHVESNLGEKSVLEINGKSSDNMSKPFYQRTTSFSGSTSIPETIVPPIKGESDKQNQPQNIFVGNGAAVPAGGEDSISDLHSKASKSSAVNSDDLDEKAKPPVVQQRGRFKVTSENVDLEKVAPAPILQKSHSMQVLTPHPVVSLAPPPSDAASSTLAAHHLFPLLQSVLQTNILQRENILNLIKHISAVDSTVNRAFEGVCTPANVAVTEKSLLEAAHDRERELLHEITELQWRLICAQEELQKYKTENAQV*

>TCA.XM_007021491.1

MADIAGLQEAAGSRFSQLELIGRGSFGDVYKAFDKELNKEVAIKVIDLEESEDEIEDIQKEISVLSQCRSQYITEYYGSYLHQTKLWIIMEYMAGGSVADLLQSGPPLDEMSIACISRDLLHAIEYLHNEGKIHRDIKAANILLTENGDVKVADFGVSAQLTRTISRRKTFVGTPFWMAPEVIQNSEGYNEKADIWSLGITVIEMAKGEPPLADLHPMRVLFIIPRENPPQLDEHFSRPMKEFVSLCLKKVPAERPSAKELLKHRFIRNARKSQRLLERIRERPKYQLKEDAETPRNGPKAVGESTDTVKVTRDIRGEETVRASKQGKTFKNAGWDFSIGGEQSTGTVRNAVRPPQVRERKLEAVYNQATPRTPESVNALNEFPEVSFGKDTKKSYYDEHQDNYQEDDDTSVSGSGTVVIRSPRGSQSSALFRDPSSLSSSAYASFEDASTSGTVVFRGQHDDSDSPRTPRSRLGIQERTSSASAEDSAANLAEAKAAIQAGLRKSNVRDRPALSKFNSPGHENRRRDQMSNSSDSSRGSREYFDAQRVFPRSRQPSDDEENAKIASSSVSLSMLLIPSLKEVVADDSEGSVARAVTNSLINMERTKPGSCETLVRRLLERLASLKEPSMKDIQELAARMFNKGKATPEDTQNANMEPDSRKKQQHKELNSNSNLSPLARFLLSRWQSQTSRDLNPT*

>GSVIVT01012233001

MDPSLTPSRRTRPAVPKSDIYSTFVVHDDEDDDETAFQEKYSRRRTKSQEKEDIYATMVYKDDPNDDDDDDSSLPPLLKRLPKDFDAAHDYYDEDSTGTGDFGTMIPRGSPYLERSTGKRTDDEDNYSTFVVRSTLGTRESGTVVRRGSGGASASSTMSRAVASMQASGELGFRKHRKGSGSSQGDEARFQASKISTSSIPESMTREDPSTKYELLNELGKGSYGAVYKARDIRTSELVAIKVISLCEGEEGYEEIRGEIEMLQQCSHPNVVRYLGSYQGEEYLWIVMEYCGGGSVADLMNTTEEPLDEYQIAYICREALKGLSYLHSIFKVHRDIKGGNILLTEQGEVKLGDFGVAAQLTRTMSKRNTFIGTPHWMAPEVIQESRYDGKVDVWALGVSAIEMAEGLPPRSAVHPMRVLFMISIEPAPMLEDKEKWSLVFHDFVAKCLTKEPRLRPTASEMLKHKFIEKCKCGASAMLPKIEKARQIRASMALQAQSLARITSISGDAPPEGPKLNEDYGDTVPSRPFNNGLQVTNEVPTASTLVKQKISDGVELEGEGEFGTVIVHGGFEMDKTANQTPVSSTKEPSAAHENVESHPVGGPGIKSTNNWVEDTVDVAANNDQVGESHPGEQTTSKSVFGSPEQNLRTSSISQVQAGGGGVSSSQLKNETVSRTAFASQDKLWSIYAAGNTVPIPFLRATDISPIALLSGNVLGGRQRESSGAVAVEAVQELFTGDSQLKKGRRGQNEIPLPPSMYQRLTSSSTLLNLAQALAYHKTSYEEMPLQDLQAVQEQQTIQNLCDTLRTILRL*

>GSVIVT01013739001

MEYLSNKKFPINSKDYKLYEEVGEGVSATVYRALCIPFNEIVAVKVMDLERCNDNLDGIRHEVQTMSLIDHPNVLRAHCSFSVGSCLWIVMPYMASGSCLHIMKSSYPEGFEQAVIATLLREVLKALVYLHSNGHIHRDVKAGNILVDSKGAIKLADFGVSACMFDAGDRQRSRNTFVGTPCWMAPEVMQQLNGYDFKADIWSFGITALELAHGHAPFSKYPPMKVLLMTLQNAPPGLDYERDRRFSKSFKEMVGACLVKDPKKRPTSEKLLKHRFFKHACSKEFLAWAILNGLAPLGDRFRMLKADFLVQKRGMDGNKEQLSQVTISKFVLKWKFSIGRKGKQNSLKYENFKKEPKDPKHDSVHNTK*

>GSVIVT01014297001

MAEAAAIMDATGSRFSSLELIGRGSFGDVYKGFDKELNKDVAIKVIDLEEAEDEIEDIQKEISVLSQCRSPYITEYYGSYLHQTKLWIIMEYMAGGSVADLIQSGQPLDEMSIACILRDLLHAIEYLHNEGKIHRDIKAANILLTENGDVKVADFGVSAQLTRTISRRKTFVGTPFWMAPEVIQNSEGYNEKADIWSLGITAIEMAKGEPPLADLHPMRVLFIIPRENPPQLDEHFSRPMKEFVSLCLKKVPAERPSAKELLKHRFIRNARKSPRLLERIRERPKYQIKDDAETPRNGPKGIGEGSDTVKVTRDSRGEETVRASSQGKTLRNAGWDFSLSGSQSTGTVRSVIRPPQARERKPEVSYQAPSRKTADSNNHLLSASGSGRYESSEIFLGKEARDAYYDEQDNSHEDDELSVSGSGTVVVRSPRGYQSSIPFSDQSSLSSNTYASLEDASTSGTVVIRSHHDDSDSPRTPKSRLGIQERTSTAPPEDSAINLAEAKAAMQGGLRKGNARERSVLGKVNKDEQEKRKTEQITSSSDSSRHSREYYDAPKAFARSRQSSDDDESARAAVLSSSKALSILFIPSLKEAGLDDSKGAIGHAVANALINMERTKPGSCEVLVSKLLQHLASSKESSLKDLQELATRVFTKGKTAPEEAENANAEADNRKRQQNKELNSNPNLSPLARFLLSRWQGQVSRDLSPA*

>GSVIVT01016074001

MEKKKYPIGPEFYTLYEEIGQGVSASVLRALCVPLNEIVAIKILDFERDNCDLNNISREAQTMNLVDHPNVLKSHCSFVSEHNLWVVMPYMAGGSCLNILKAAYPDGLEEVVIATILREVLKGLEYLHHHGHIHRDVKAGNILIDARGAIKLGDFGVSACLFDSGDRQRSRNTFVGTPCWMAPEVMEQLHGYDFKADIWSFGITALELAHGHAPFSKYPPMKVLLMTLQNAPPGLDYERDRKFSKSFKQMIASCLVKDPSKRPSAKKLLKHPFFKQARSSDYIGRTLLEGLPDLGERIKELKRKEEDMLAQKKMPDGEKEELSQILNLNSMNEYKRGISGWNFNLEDVKAQASLIPDVEDSGSDLGGSSNSLSGLDVHEKQSSMGHLSQVAEEGSDLMQNLPVPLPSVDSAINNIRVQSYKSDDESSIASSSHEHHISQGSSPRHDDQIENNLAEKPDPEISEKLLDMAIQSQKVGSSSDSTSSLEVNCPVKGESGNLQNQLRKMSSCNGTTVVTVVDEAPSEIISRTSKSSANSDEPDERAKMPVVQQRGRFKVTSENVDLEKVNL*

>GSVIVT01019643001

MARTGTSHKTYSANPSDYKLLEEVGYGASATVYRAIYLPFNEVVAVKCLDLDRCNSNLDDIRREAQTMSLIDHPNVIKAYCSFVVERNLWVVMPFMAEGSCLHLMKIAYTDGFEESAIGSILKETLKALEYLHRHGHIHRDVKAGNILLDSNGVVKLADFGVSACMFDKGDRQRSRNTFVGTPCWMAPEVLQPGSGYDFKADIWSFGITALELAHGHAPFSKYPPMKVLLMTIQNAPPGLDYDRDKKFSKSFKEMVAMCLVKDQTKRPTAEKLLKHSFFKQAKPPELSVKKLFAELPPLWQRVKALQLKDAAQLALKKMPSAEQEAISQSEYKRGVSAWNFDIEDLKVQASLVQDDDEIQEMREEDESMKSFVIDKDSSDSRSSLGKSISTNDNFFRGRITGDELAQAECLSKKGKSLRSDSLESSNQEKIGQEKDISSAEMMPSTSEKDMVNAKTKTQMVKGRQTQSGPLMPGVVLSHSLSDRGRSFERSENEIQPITEKANREVRRAPSFSGPLMLPNRASANSLSAPIKSSAGYRDSLEEKSKANLVQIKGRFSVTSENVDLVKDIPLCAVARRSSQGSPLRKSASVGDWMFDSKPMLTTPKDFSNSNVPASLLMPHLQNLFQQTSLQQDLITNLLNSLQSSEIVDASQNGKLPPLPRGSENNGNVDPGASERERLLLLKVSELQARMINLTDELTAEKFKYMQLQQQLNAVSGQEEDGDKREGDA*

>GSVIVT01027718001

MAQHEGEASPRVQYPLNSEAYRILDEIGVGVSAIVYKAECLPMNSALVAIKSIDLDQSRADLDSVLREVKTMSLLSHPNILRAHCSFAVDHRLWVVMPFMSAGSLQSIIGSSFPDGLPEPCIAIVLKETLNALSYLHDQGHLHRDIKAGNILVDSTGTVKLADFGVSALIYESNTITGTPYWMAPEVIHSHTGYSLKADIWSFGITALELAHGRPPLSHLPPSKSLVMKITKRFRFSDYEQSRSEQSPKFSKSFKDMVASCLDQDPSKRPSADKLLKHPFFKNCKNSEFLVNKVLRISGWNFKEDVLELDPVFPSETETLSPPRRQVRFAAEPIIRDPSNESSSDNPGSSSQSVEGVQELHDEHTNEHAGMRMLRGLTRSLYDQRRAVGLMLTILTGQEEVSREDQLLQENERLKMELEQEKKKTSEMEMEMEFLSLQISRGSTSAASRQSGE*

>GSVIVT01032461001

MEHEKKFPLDAKDYKLYEEVGEGVSATVYRALCIPLNEIVAIKVLDLEKCNNDLDGIRREVQTMSLIDHPNLLQAHCSFTSGHTLWVVMPYMAGGSCLHIMKSEYPEGFDEPVIATLLREVLKALVYLHNHGHIHRDVKAGNILIDSNGAVKLADFGVSACMFDAGDRQRSRNTFVGTPCWMAPEVMQQLHGYDFKADIWSFGITALELAHGHAPFSKYPPMKVLLMTLQNAPPGLDYERDKKFSKSFKEMVATCLVKDPKKRPTSEKLFKHSFFKHARSNEYLARTILEGLTPLGDRFRTLKAKEADLLVQNKALYGDKEQLSQQEYIRGISAWNFNLEDLKNQAALIQDYDVISNVDDPDCSNAAPSHEDGLNDLSNLENSLASFPIQPLQALKGYFDVCEDDGNASSLSWRDVMQSESEQQSVDQEAEKDDGENFGRSSSLPRQIIPGHKKFFSGSLLQDNALSPKKVNGDGDRENIQSRYQPERNYSGPLLHRQKRDTNNISSVEDTPEGAVVQCKGRFKVTSAELSPKGPTNCFFSQVSGGSTSPTTPSLTAASILPSLQFVLQQNTMQREGIMKLIKYVEQSCGNASHPLIVCI*

>Zm00001d018352_T001

MTGPGDKAAAGGAGGERRKYPIHVEDYELYEEIGQGVSAVVYRALCKPLDEIVAVKVLDFERTNSDLNSIVREAQTMILIDHPNVVKAHCSFAKDQTLWVVMPYMAGGSCLHIMKSVHPTGFEEPIIATILREVLKGLEYLHHHGSIHRDVKAGNILVDSRGGIKLGDFGVSACLFDSGDRQRARNTFVGTPCWMAPEVMQQIHGYNFRADIWSFGITALELAHGHAPFSKYPPMKVLLMTLQNAPPGLDYERDKKFSRNFKQMVAMCLVKDPSKRPPAKKLLKQPFFKQARSTDFIARKLLEGLPGLGFRYQALKEKDQDLMAQKKMSDGKKEEISQDEYKRGISSWNFDIDDLRSQASLGTECEDNISCKDSDVSFYDSDSLQDQAPEVPHLSKDFSTKYDADVENDMAAKDKSAVSPPDQPACLLRNASMHGMPITGSVTKENSTESLDLECQEKHPDIIPTSSSHERKFSFSSCSSDGFLSSKESTKQQSSIHNRDKCNGAPLHVSDETSYEAAPKAHKSAEDHDDRSKPPLIRGRFKVIPAHVDFDKAQQPGLQKCHSMQTISRLPSLSIPSSAEVASNIIGGSFYMQLYSILQTNILQRDQILNAMKQVSGCDIASPGVPSMASQCIPSTSRSTSPSGAISVDRSMLEAAHEREKELLNEVLELQWR*

>Zm00001d023240_T001

MAAANNAASGGGDEARYPLNAESYRLLCKIGSGVSAVVYKAVCLPLGSSAVVAIKAIDLERSRADLDGVQREAKAMALLSHGNVLRAHCSFTVGSHLWVVMPFMGAGSLHSILRHGFPGGLPEPCVAVVLGETLRALCYLHGQGRIHRDIKAGNILVDSDGAVKLADFGVSASIYETIRQHASASASTAAAVGTGSSASCCFNDVAGTPYWMAPEVIHSHVGYGIKADIWSFGITALELAHGRPPLSHLPPSKSMLMRITSRVRFEESVGTGNRRDKNNKFSRAFKDMVSACLCQEPAKRPSAEKLLRHPFFKACSRRSRDFLVRNVLAAVPSIEERCRKDDEAGNDLCGCVGGGARCVSPCPRHADNVVVAKNRRISGWNFNEDNLEFDPTTEERPVEAEKRCLPFDFDEGDDQVELHDSNSAGDGDGDRGRPPAQPQHGDGDDHVHGKAAEAVGLKQQQQVVTQQLMTVLQSLEMQRDMVTHVLQRTAGRLDDGGGNGDVSVTAPREEREEMLLGYVRQLEHRVEDLRKEVEEEMAWNARLEKMLQERVIISDDDKKMNSSKTSSSAELRAVDENI*

>Zm00001d045967_T001

MEHVLSSRRFPTDPNEYKLYEEIGDGVSATVYRALCVPLDILVAIKVLDLEKCNNDLDGIRREVQTMSLIDHPNLLRAYCSFTNGHQLWVVMPYMAAGSALHIMKTSFPEGFDEPVIATLLREVLKALVYLHSQGHIHRDVKAGNILIDTNGAVKLGDFGVSACIADIWSFGITALELAHGHAPFSKYPPMKVLLMTLQNAPPGLDYERDKRFSKSFKDLVATCLVKDPRKRPSSEKLLKHSFFKHARSAEYLARSILDGLPPLGERFRELKSKEAELLLNNKLGQESKEQLSQMQLFAFQKEYIRGISGWNFNLEDLKNAAALIDSSNGTCYLDVRENRVKDDSQEAYNGPEHIYQERLNHVASRRPEEDEIQEVEALNDAVSSSFSSRSLEALKSCFDVCGVDDPSPTATDSRAQPSVGTLPFQQLQKFEHCKSGDCNGESLERSVSVPTNLVTSGYHKHSSGSLIPEQVLSPYLSSDLERYFFLKFIFPCPTQFGLSHPFLRAVAPDESSEGKIIRRRGRFQVTSDSISEKVATSSCNSSRINLPIDAARSSPKLSAILPTLQFLMKQNTMQKEVLSRLISSIEETSGMHEYSSLF*

>Zm00001d013836_T001

MGRNGSVKRSASSSAAGAAAGPPSFSVNPADYRLMEEVGYGAHAVVYRAIFLPTEDVVAVKCLDLDQLNNNIDEIQREAQIMSLIDHPNVIRAYCSFVVEHSLWVIMPFMTEGSCLHLMKISYQEGFDEPVIGSILKETLKALEYLHRQGQIHRDVKAGNILIDGAGVVKLGDFGVSACMFDRGDRQRSRNTFVGTPCWMAPEVLQPGTGYNFKADIWSFGITALELAHGHAPFSKYPPMKVLLMTLQNAPPGLDYERDRRFSKSFKEMVAMCLVKDQTKRPTAEKLLKHSFFKNAKPPELTIKNILSGLPPLWDRVKALQLKDAAQLALKKMPSSEQEALSLSEYQRGVSAWNFDIEDLKAQASLIHDDEPPEIKEDDDIPRNIEDLSSRNHLAKSSANECNSSQRAFATTLNSDGNSPTMNEAFDFDISDADTTRRADGYESNTRQNDSLPSTSKRDPESNHWASDGQRQLTSGGNNSSTSERGYGFERDASVQMISDKQRSEMRKTASLSGPLSLPTRASANSLSAPIRSSGGYVDSSGDKSKRSVVEIKGRFSVTSENVDLAKVQEGPLSSLSRKSPEGSLLRKSASTGDYFVNPKLMCNANQLKELCNSSVSSSVLIPHLNNLVQQTMFQQDLIMNLLSNLQQSEKVDGTQPIISSQVRTMENDKVADTVNSVKERSLLVKISELQSRMIILTDELIAAKLKHVQLQQELNALYCREEIVDIRDEDNEET*

>Zm00001d021584_T001

MSRRKTFVGTPFWMAPEVIQNSEGYNEKADIWSLGITAIEMAKGEPPLADIHPMRVLFIIPRENPPQLDEHFSKPMKEFVSLCLKKNPAERPSAKELLKHRFIKNARKTPKLLERIRERPKFAAKSMDATQNGQTHDEEEDFGTGTIKVNRTKDTAPSLSQGTVRKSAVRDFSDRSEGTGTVRVVSRPPQIASTKDGRSDMPQSPKAPIRTADRENQWRSSGTGSEESLSLRDTQSGRGRVESSTDDNDHSVSGSGTVVLRSPRASQLYSTASNHSAKPPSRFSSYEDMSNSGTVVRTQNEDPETPRSSRSRLGIQEKTSNAPLEDSAINLAEAKAALQAGLRKGNARERPFINRHEKGSHEHRSSGVNSQEVQSEDVDTQKGHKLRQLPDGQSASRASSVASPAVSSLLLPSLKEATGDKFDRPAVHAFLDSLMDLERDIPGSCEVLVGRLLHRLGSSKDSSLQGLQETAMSIFTKEPEPPSEKAGDKKLANMPPLAAPTVSPLARFLLTRWQNQVSQDLNSV*

>Zm00001d027340_T001

MVRSGSIRRSAVPAPMPAPAFTVSPADYRLLEEVGYGANAVVYRAEFIPTGRTVAVKCLDLDRVNSNLDDVRKETQTMSLIDHPNVIRSYCSFVVGHNLWVVMPFMSEGSCLHLMKVAYPDGFEEPIIASILKETLKALDYLHRQGHIHRDVKAGNILIDNPGVVKLGDFGVSACMFDRGDRQRARNTFVGTPCWMAPEVLQPGTGYNFKADIWSFGITALELAHGHAPFSKYPPMKVLLMTLQNAPPGLDYDRDKRFSKSFKEMVAMCLVKDQTKRPTAEKLLKHSFFKNAKPPELTVKNILTDLPPLWDRVKALQLKDAAQLALKRMPSSEQEALSMSEYQRGVSAWNFDIEDLKAQASLICDDDPPEIKEDDDTGRITDIDKDTSSDDYFGKTAPSNGNNCSDRSSVPANPGQNGPEINEILSSNNGNAYSERKPDGRKNPESENDSLPSTSKHDADGKDYRCDFRQKQRTYSGPVLQSGPNNSSMTERGHIIERDAGVQSVSDKQKNGTRRANNLSGPLSLPTRASANSLSRANNLSGPLSLPTRASANSLSAPIRSSAGYVGSLGDKPKRTMVEIKGRFSVTSENVDLAKVQEIPGSSASRKLQEGPSLRKSASVGDWSANDKTTSTNHQRKELCNSSVSTSILIPHLQNLVKQTAFQQDLITNLLSSLQQNERVDAAQSRVQSTTSDTVVEAATAEREHSLLVKIFELQSRMISLTDELIAAKLKHVQKHAFKTIINDRLRQLCTCLTYDPLCCYQLQEELNVLYCQEEILDMREDESEEA*

>Zm00001d032440_T002

MGRNGSVRRSAFSGAAGAAAGPPSFSVNPADYRLMEEVGYGAHAVVYRAIFLPTKGVVAVKCLDLDQLNNNIDEIQREAQIMSLIDHPNVIRAYCSFVVEHSLWVIMPFMTEGSCLHLMKISYQEGFDEPIIGSILKETLKALDYLHRQGQIHRDVKAGNILIDGAGVVKLGDFGVSACMFDRGDRQRSRNTFVGTPCWMAPEVLQPGTGYNFKADIWSFGITALELAHGHAPFSKYPPMKVLLMTLQNAPPGLDYERDKRFSKSFKEMVAMCLVKDQTKRPTAEKLLKHSFFKNAKPPELTIKSILSGLPPLWDRVKALQLKDEAQLALKKMPSSEQEALSLSEYQRGVSAWNFDIEDLKAQASLIHDDEPPEIKEDEDIARNIEVEKDLSSRNHLGKSFANECNSRQRAFVTTLKSDGNRPTTNEAFDFDFDFSEAATTRRADGHERNIRENDSLPSTAKRDPESNHWTSDVGQRQLSSGGSNSSTAERGYGFERDAVVQTIPEKQRSEARKTASLSGPLSLPTRASANSLSAPIRSSGGFVDSSGDKSKRSVVEIKGRFSVTSENVDLAKVQEGPLSSLSRKSPEGSLLKKSASTGDCLINTKLMV*

>Zm00001d034055_T001

MAFSPRSPWSRAKKTDVYSTFVVHGDDDEDDARGGRGPSTRAEEDEEEDPSSLPPLLQRLPKDFGGASFDDDEDPYSSDPDDASLSATAAFKSGVPASGRSPFLDLRRSSPRATDDDPYSTFVVHSTTRSGRTSSSPRGSASGTFIRRSGGSSSPRESVSGTFIRRTGSPSSPGKSASGTFIRRPGSPSSPRGSFSGTFIRHTSGGSSSHESASGGGGGFGSSFWTPAVEQSEELRQPSPLMQQQQQQHSRRKPSVSSVPDSVTREDPSTKYELLHELGKGSYGAVYKARDLRTQELVAVKIISLTEGEEGYEDIRGEIEMLQQCSHPNVVRYFGSYQGEEYLWIIMEYCGGGSVADLIGITEEPLDESQIAYICREALKGLAYLHSIFKVHRDIKGGNILLTEQGEVKLGDFGVAAQLTRTMSKRNTFIGTPHWMAPEVIQESRYDGKVDVWALGVSAIEMAEGMPPRSTVHPMRVIFMISSEPAPMLEDKEKWSLLFHDFIAKCLTKDPRLRPAAIEMLKHKFIEKCNSGASKMLAKIKVAKKIRATLAVQNELSDPDNTTQDVPFRINEDYGETVPTSSQQHMKHGTYNDCQAGDFGTMIVHTEDGDEVTESPIFPRTEFIPGLGSINSFTHDPKRAELISSFWAENIGDSDANKDRDTDYGPDMQESKVISPLTGTVKKNIGAEGTIRCHDNQINLSPGLANTTTKLNNSPSRKAFSVQDKLWSIYAAGNTVPIPFLKAIDISPLALVSENESGNGLAGSSTNDALEAVRELFSGDGQAKKGRKGQNEVPLPPGVHHRLTTSPTLMNLAQALAYHKTCYEDMPLQDSQATQEQQTIQNLCDTLRTILRL*

>Zm00001d052051_T001

MAGPNEKAAAGGGGGERRKYPIHMEDYELYEEIGQGVSAIVYRALCKPLDEIVAVKVVDFERTNSDLNNIVREAQTMILIDHPNVVKAHCSFAKDQTLWVVMPYMAGGSCLHIMKSVHPTGFEEPIIATILREVLKGLEYLHHHGSIHRDVKAGNILVDSRGGIKLGDFGVSACLFDSGDRQRARNTFVGTPCWMAPEVMEQLHGYDFRADIWSFGITALELAHGHAPFSKYPPMKVLLMTLQNAPPGLDYERDKKFTRNFKQMVAMCLVKDPSKRPSAKKLLKQPFFKQARSTDFIARKLLEGLPGLGVRYQALKEKDQHLMAQKKMSDGKKEEISQDEYKRGISSWTFDMDDLRSQASLGTECEDSTLCKDSDISFYDLDSLQDQAPEGSHLLRDFSTKYDADIENDMTTKDKSAVSSPDQPASLVRNASMRGMPINGSFRKDNSTESFDLECQEKHLDIVPTSSSPERKFSFSSCSSNGLLLSKESSKQQTSIHNLDKCNGGHLHVSDETSSEAAPKTHKSAEDHDDRSKPPLIRGRFRVIPGHVDSKAQPPGLQKCHSMQTISRLPSLSIPSSAEVASTIIGGSFYMQLYSILQTNMLQRDQILNAMKQVSGCAMASPGVPSMASPCIPSTSRSTSPSGVPSVDRSMLEAAQEREKELLNEVLELQWRLLCTQDEVQKLKAKAAQI*

>Zm00001d053735_T001

MWKGKKSGPLAPRGRKREFPIRAADYELLEPIGDGATAVVRRARCLPHGGEIVAVKVMNMAHRTESDVNNASEEVKTMIMIDHPNLLSAYCSFTEGEALWIVMPYMAGGSCYHLMKSSYPKGFDDENFIAFVLRETLKGLEYLHENGHIHRDVKAGNILLDQDKGVKLSDFGVTASLYDSIINRHGKRKTLVGTPCWMAPEVMEQKDYDFKADIWSFGITALELAIGHAPFSSQPPAKVFLMTLQHAPPSLHNTKEKKFSDSFKSMIATCLIKDPTKRPPAKKLLKHPFFRRARSDHNAVKCMLNKLPSLAERMQFIKENEAKLQADKKPLDNCKEKASQEEYRRGVSEWNFDIADLKAQAALYPDENEAEDFLRFLFELDIVDETTQLKDIRAESHSINNDGTNVADDGLGKSNSTSPMSSSQSVKQLDKGSPNGLVRSESFEIHSISPAKQLTTAVSTCNDVDEYLEKTAFQKGRFKVIHDYSKLEGATPREKELLDRISSLEQMLLATQDEVERLKAKES*
